# Thiazol-2-ylidenes as N-Heterocyclic carbene ligands with enhanced electrophilicity for transition metal catalysis

**DOI:** 10.1038/s42004-022-00675-7

**Published:** 2022-05-06

**Authors:** Jin Zhang, Tao Li, Xiangyang Li, Anqi Lv, Xue Li, Zheng Wang, Ruihong Wang, Yangmin Ma, Ran Fang, Roman Szostak, Michal Szostak

**Affiliations:** 1 College of Chemistry and Chemical Engineering, Key Laboratory of Chemical Additives for China National Light Industry, Shaanxi University of Science and Technology, Xi’an, 710021 China; 2 Institute of Frontier Science and Technology Transfer, Shaanxi University of Science and Technology, Xi’an, 710021 China; 3 Department of Chemistry, Wroclaw University, F. Joliot-Curie 14, Wroclaw, 50-383 Poland; 4 Department of Chemistry, Rutgers University, 73 Warren Street, Newark, NJ 07102 USA

**Keywords:** Structure elucidation, Organometallic chemistry, Synthetic chemistry methodology

## Abstract

Over the last 20 years, N-heterocyclic carbenes (NHCs) have emerged as a dominant direction in ligand development in transition metal catalysis. In particular, strong σ-donation in combination with tunable steric environment make NHCs to be among the most common ligands used for C–C and C–heteroatom bond formation. Herein, we report the study on steric and electronic properties of thiazol-2-ylidenes. We demonstrate that the thiazole heterocycle and enhanced π-electrophilicity result in a class of highly active carbene ligands for electrophilic cyclization reactions to form valuable oxazoline heterocycles. The evaluation of steric, electron-donating and π-accepting properties as well as structural characterization and coordination chemistry is presented. This mode of catalysis can be applied to late-stage drug functionalization to furnish attractive building blocks for medicinal chemistry. Considering the key role of N-heterocyclic ligands, we anticipate that *N*-aryl thiazol-2-ylidenes will be of broad interest as ligands in modern chemical synthesis.

## Abstract (web summary)

*N*-heterocyclic carbenes are widely exploited as ligands in transition metal catalysis, but are largely limited to *N*-arylimidazolylidenes. Here, the authors report on the steric and electronic properties of thiazol-2-ylidenes, demonstrating that their enhanced π-electrophilicity makes them attractive as carbene ligands in transition metal-catalysed electrophilic cyclization reactions.

## Introduction

**Fig. 1:**
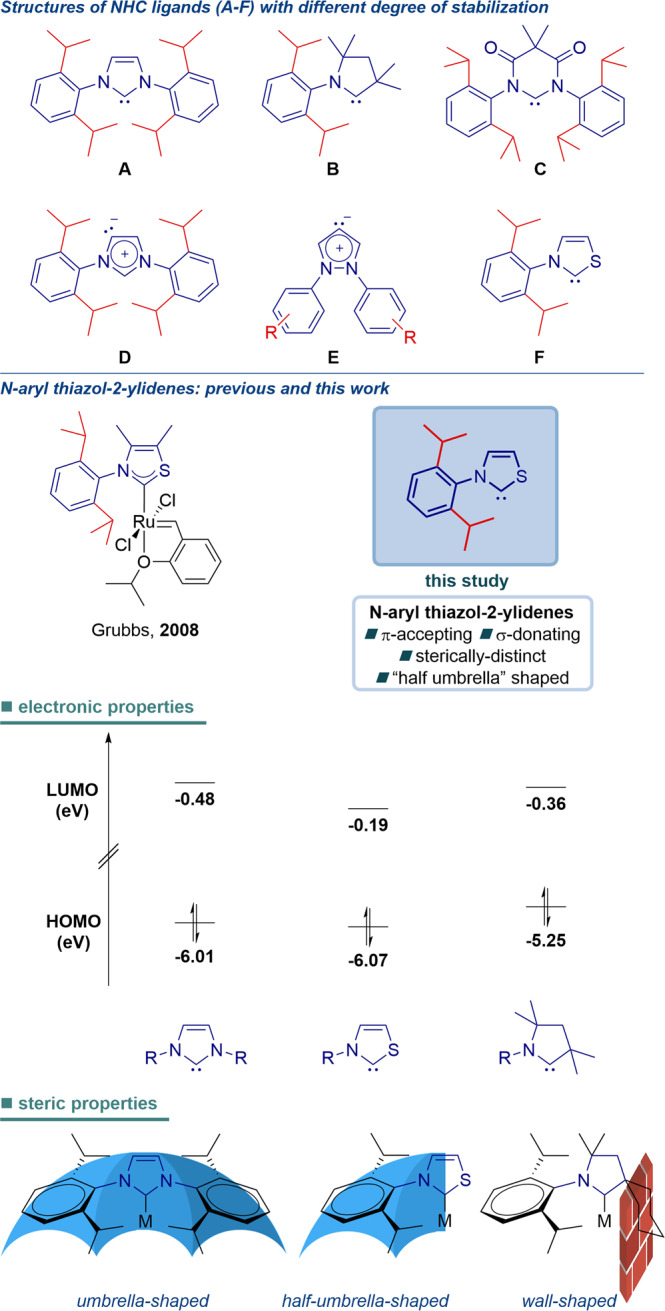
Structures of N-heterocyclic carbenes with different degrees of stabilization. Energies calculated at B3LYP 6-311 + +g(d,p) level, R = Dipp.

Since the first successful isolation in 1991^1^ and the first use in catalysis in 1995^2^, N-heterocyclic carbenes (NHCs) have emerged as a powerful class of ligands in transition metal catalysis^3–14^. The tremendous utility of NHCs hinges on strong σ-donation^15,16^ in combination with tunable steric environment^17,18^, supercharging the catalytic activity of transition metals beyond other ligands. The most remarkable impact is in the development of Ru-catalyzed olefin metathesis^19,20^ and Pd-catalyzed cross-couplings^21–23^, where the strong σ-donation and high stability of M–C_(NHC)_ bond render NHCs superior to the more ubiquitous phosphine ligands. Thus far, NHC ligand development in transition metal catalysis has been almost exclusively limited to *N*-aryl-imidazolylidenes **A**^5–23^, such as IPr^24–26^, prepared by deprotonation of symmetrical imidazolium salts (Fig. 1). This is presumably due to enhanced electronic and steric stabilization of the carbene center by two nitrogen atoms as well as two N-Ar wingtip substituents, which render *N*-aryl-imidazolylidenes more stable and easier to handle^27^. The pioneering studies by Bertrand and co-workers established that cyclic carbene systems with a marked decrease of heteroatom stabilization, such as CAACs **B** (cyclic (alkyl)amino)carbenes)^28–35^, are readily available, showing unique reactivity as supporting ligands in transition metal catalysis. More reactive and less stabilized systems, such as diamidocarbenes **C**^36–38^, mesoionic carbenes **D**^39–41^ and remote carbenes **E**^42–45^, have been developed, each class showing varying degrees of heteroatom stabilization and distinctive promise in transition metal catalysis^46–48^.

In this context, *N*-aryl thiazol-2-ylidenes **F** are an intriguing class of N-heterocyclic carbenes (Fig. 1). Following the isolation of a stable thiazol-2-ylidene by Arduengo in 1997^49^, this class of ligands stayed dormant until 2008, when Grubbs demonstrated the unique reactivity of Ru-based thiazol-2-ylidene olefin metathesis catalysts^50^. To our knowledge, this is the only application of *N*-aryl thiazol-2-ylidene ligands in transition metal catalysis reported to date^51–54^. More recently, there has been a resurgence of organocatalyzed radical relays and decarboxylative couplings made possible through the persistent radical stabilization by thiazol-2-ylidenes^55–60^. In the meantime, studies by Boydston demonstrated organocatalyzed anodic oxidation of aldehydes through in situ generation of electroauxiliaries of thiazol-2-ylidenes^61^, while the first characterization of elusive Breslow intermediates from thiazol-2-ylidenes by spectroscopic and crystallographic methods has been reported^62,63^. Thiazol-2-ylidenes are key intermediates in biochemical transformations of vitamin B1^64,65^.

Geometrically, replacement of one of the nitrogen atoms in imidazol-2-ylidene systems with sulfur in thiazol-2-ylidenes results in disrupting the ring geometry of imidazolylidenes^66–68^. At the same time, there is a strong electronic effect in decreasing stabilization of the carbene center through diminished π donation from sulfur^69^. Finally, the sulfur atom does not bear any wingtip substituents that in imidazolylidene systems often provide a significant contribution to the stabilization of the carbene center^5–23,66–68^. These geometrical and electronic factors might explain why, with exception of the report by Grubbs^50^, *N*-aryl thiazol-2-ylidenes have been unexplored as NHC ligands in transition metal catalysis.

In terms of electronics, the diminished π donation from sulfur due to ring geometry and large sulfur radius is expected to result in more electrophilic carbenes than traditional imidazol-2-ylidene systems, while maintaining strong donor ability (Fig. 1)^69^.

In terms of geometry, the effect of typical NHC ligands on M–C_(NHC)_ bond is defined as “umbrella” shaped, in contrast to cone shaped phosphines (Fig. 1)^17,18^. The combination of a nitrogen atom with a quaternary carbon in CAACs renders these ligands as “wall-shaped” in some cases with regard to the M–C_(NHC)_ bond^28–35^. The steric properties of *N*-aryl thiazol-2-ylidenes render these ligands “half umbrella” shaped with the nitrogen N-wingtip oriented toward the M–C_(NHC)_ bond and lack of substitution on the sulfur atom.

As a part of our interest in NHC catalysis^70–76^, herein, we report the study on steric and electronic properties of thiazol-2-ylidenes. Most importantly, we demonstrate that the thiazole heterocycle and enhanced π-electrophilicity result in a class of highly active carbene ligands that supersede imidazol-2-ylidenes. We present the evaluation of steric, electron-donating and π-accepting properties as well as structural characterization and coordination chemistry. Considering the key role of N-heterocyclic ligands, we envision that *N*-aryl thiazol-2-ylidenes will be of broad interest as ligands in chemical synthesis.

## Results

### Synthesis of Thiazol-2-ylidene precursors

**Fig. 2:**
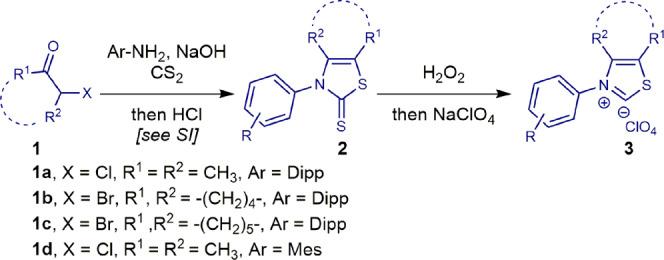
Synthesis of thiazol-2-ylidene precursors. See Supplementary Method 2 for details.

*N*-Aryl thiazol-2-ylidenes carbene precursors are readily available on multigram scale following protocols for organocatalytic transformations (see Supplementary Method 2)^55–60^. Four *N*-aryl thiazol-2-ylidenes carbene precursors were selected as a starting point (Fig. 2). For the study, we chose structural sulfur analogues of IPr and IMes on 3-aryl-4,5-dimethylthiazol-2-ylidene framework; ^**Me**^**IPrS** and ^**Me**^**IMesS**. IPr ligand (IPr = 1,3-bis(2,6-diisopropylphenyl)imidazol-2-ylidene; N-Dipp, Dipp = 2,6-diisopropylphenyl) is by far the most common NHC ligand used in transition metal catalysis, while its smaller IMes (IMes = 1,3-bis(2,4,6-trimethylphenyl)imidazol-2-ylidene, *N*-Mes, Mes = 2,4,6-trimethylphenyl) counterpart is often used for transformations requiring lower steric demand of the N-wingtip substituents. Furthermore, 3-aryl-4,5-cyclohexylthiazol-2-ylidene and 3-aryl-4,5-cycloheptylthiazol-2-ylidene, ^**6**^**IPrS** and ^**7**^**IPrS** were selected on the basis of the recent reports in organocatalytic transformations^55–60^, where the fused cyclic ring on the backbone of *N*-aryl thiazol-2-ylidenes often provided advantageous stability of the system.

### Synthesis of Thiazol-2-ylidene Complexes

**Fig. 3:**
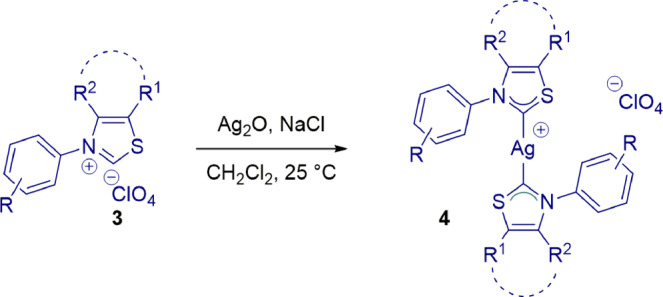
Synthesis of Ag(I) complexes. Conditions: Ag_2_O (0.5 equiv), NaCl (2.0 equiv), CH_2_Cl_2_, 25 °C, 16 h, **4a**: 96%; **4b**: 95%; **4c**: 97%; **4d**: 90%.

**Fig. 4:**
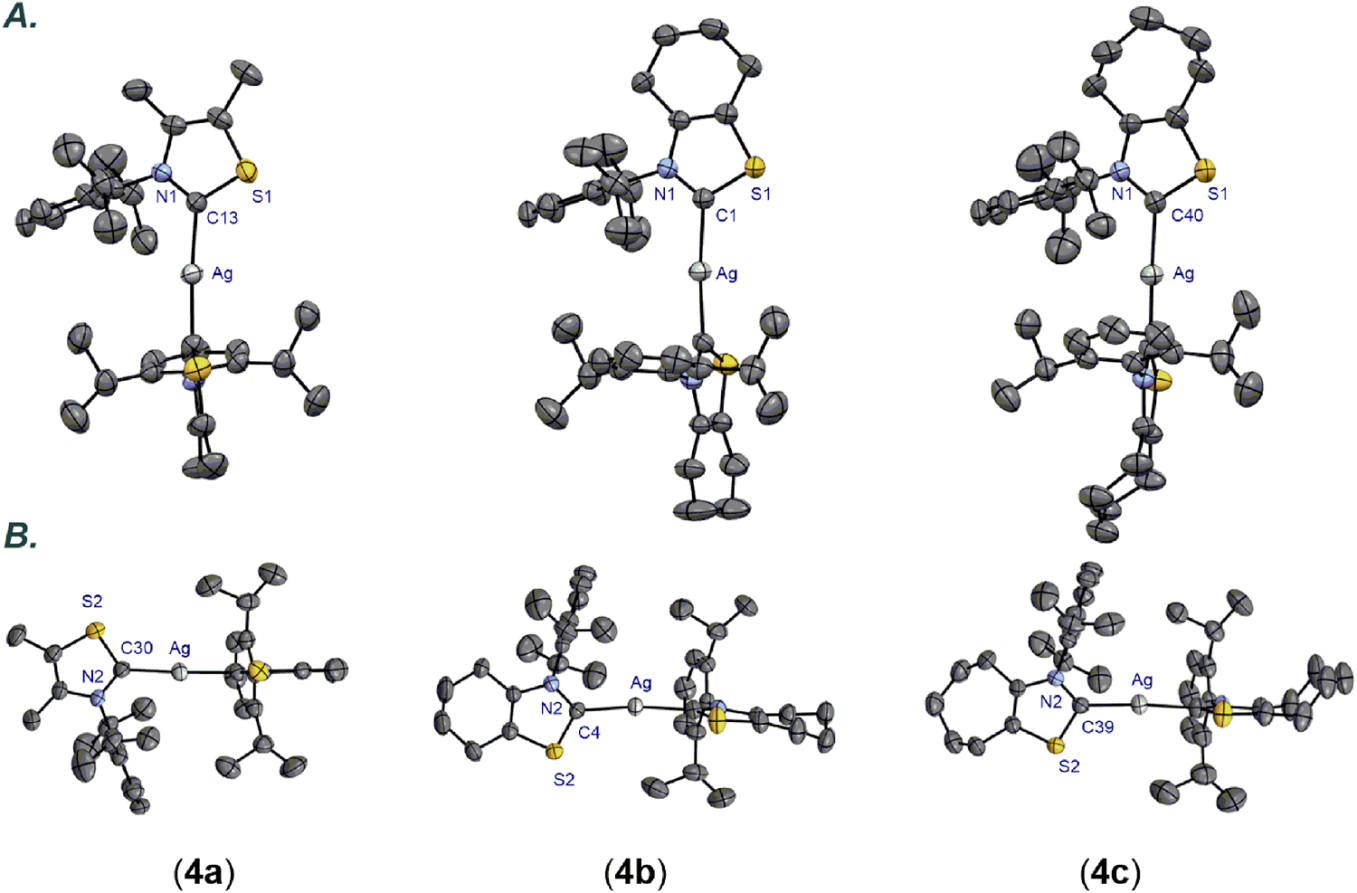
X-ray crystal structure of Ag(I) complexes 4a-4c. Two views: front (**A**. top); side (**B**. bottom). Hydrogen atoms and counterion have been omitted for clarity. Selected bond lengths [Å] and angles [°]: **4a**: Ag–C13, 2.082(3); Ag–C30, 2.086(3); N1–C13, 1.335(4); N2–C30, 1.337(4); S1–C13, 1.703(3); S2–C30, 1.697(3); C13–Ag–C30, 175.0(1). **4b**: Ag–C1, 2.075(4); Ag–C4, 2.078(4); N1–C1, 1.342(6); N2–C4, 1.331(6); S1–C1, 1.691(5); S2–C4, 1.693(6); C1–Ag–C4, 172.2(2). **4c**: Ag–C40, 2.087(2); Ag–C39, 2.081(3); N1–C40, 1.339(3); N2–C39, 1.336(3); S1–C40, 1.699(4); S2–C39, 1.704(3); C40–Ag–C39, 175.5(1). **4a**: CCDC 2117719; **4b**: CCDC 2117722; **4c**: CCDC 2117721.

With access to *N*-aryl thiazol-2-ylidene precursors, we next prepared Ag(I) complexes [Ag(**NHC**)_2_](ClO_4_) **4a-4d** by the reaction with Ag_2_O in CH_2_Cl_2_ (Fig. 3). Interestingly, complexes **4a-4c** ([Ag(^**Me**^**IPrS**)_2_](ClO_4_) (**4a**), [Ag(^**6**^**IPrS**)_2_](ClO_4_) (**4b**) and [Ag(^**7**^**IPrS**)_2_](ClO_4_) (**4c**)) were found to be stable to air and moisture and could be fully characterized by X-ray crystallography (Fig. 4, for more details, see Supplementary Note 1 and Supplementary Data 1–3). In contrast, the less sterically-hindered Ag(I) complex **4d** [Ag(^**Me**^**IMesS**)_2_](ClO_4_) was found to be significantly less stable. Arduengo reported that small N-wingtip substituents in thiazol-2-ylidenes result in unstable carbenes^49^. Unsurprisingly, the “half-umbrella” shape of *N*-aryl thiazol-2-ylidenes requires larger groups at the nitrogen atom for easy handling and isolation. Likewise, we found that the formation of bis-NHC–Ag(I) is needed to prevent decomposition of monomeric Ag(I)–NHCs in these thiazol-2-ylidene systems. From the outset, we were interested in Ag(I)–NHC complexes because of the untapped potential of Ag(I)–NHC complexes in catalysis as compared to other coinage metals^77,78^.

**Fig. 5:**
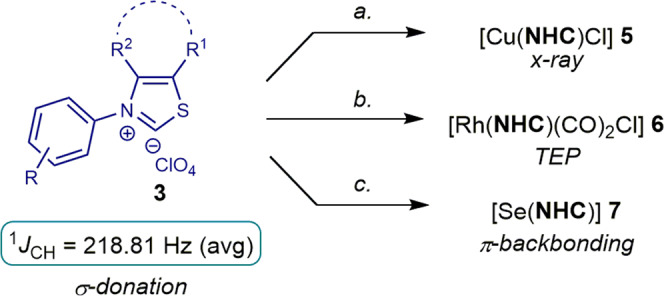
Synthesis of thiazol-2-ylidene complexes. The respective experimental conditions can be found in the Supplementary Method 4–6.

We next comprehensively evaluated steric and electronic properties of these *N*-aryl thiazol-2-ylidene ligands. As shown in Fig. 5, the linear copper(I) complex [Cu(^**Me**^**IPrS**)Cl] (**5a**) was prepared after deprotonation with an excess of KO*t*-Bu (2 equiv), while Rh(I) complexes, [Rh(^**6**^**IPrS**)(CO)_2_Cl] (**6b**) and [Rh(^**7**^**IPrS**)(CO)_2_Cl] (**6c**) were prepared by a two-step procedure via [Rh(**NHC**)(cod)Cl] and the reaction with carbon monoxide. We have also prepared the selenium adducts [Se(**NHC**)] (**5a**–**5d**) by adding the free carbene generated in situ to excess of selenium (The respective experimental conditions could be found in the Supplementary Method 4–6.).

**Fig. 6:**
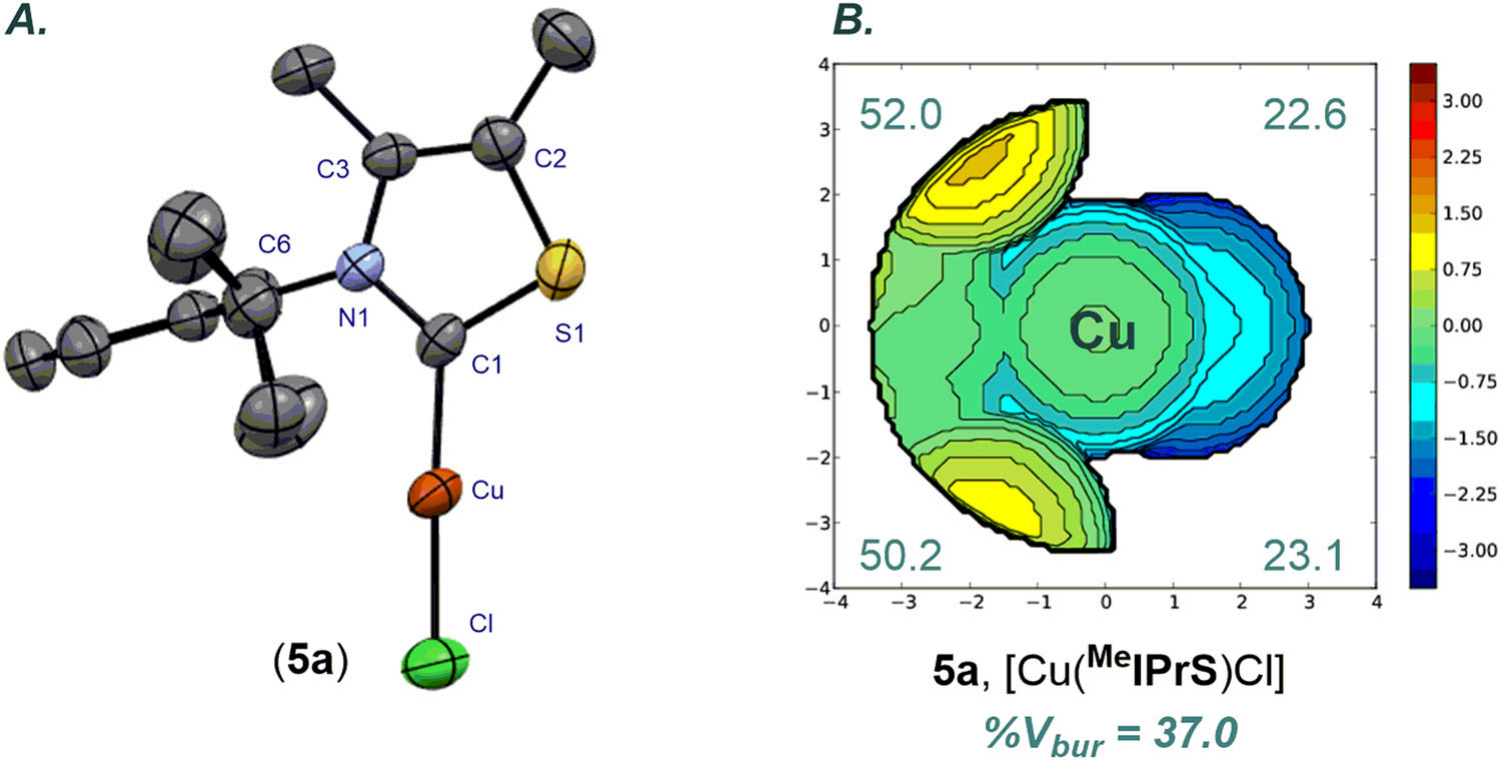
X-ray crystal structure and Topographical steric map. **A** X-ray crystal structure of complex (**5a**). Hydrogen atoms have been omitted for clarity. Selected bond lengths [Å] and angles [°]: Cu–C1, 1.871(2); Cu–Cl, 2.0947(7); C1–N1, 1.338(2); C1–S1, 1.700(2); C6–N1, 1.460(3); N1–C3, 1.402(3); S1–C2, 1.723(2); C1–Cu–Cl, 176.98(7); N1–C1–S1, 107.3(1); C6–N1–C1, 121.3(2); C3–N1–C1, 117.0(2); C2–S1–C1, 94.5(1). **B** Topographical steric map of [Cu(^**Me**^**IPrS**)Cl] (**5a**) showing % V_*bur*_ per quadrant. CCDC 2117739. Note dissymmetry of the ring.

Cu(I)–NHC complex [Cu(^**Me**^**IPrS**)Cl] (**5a**) was fully characterized by X-ray crystallography (Fig. 6, for more details, see Supplementary Note 4 and Supplementary Data 4). Studies by Cavallo and co-workers demonstrated that catalytic pockets of M–NHCs are best described by the % buried volume (%V_*bur*_) of model linear [M(NHC)Cl] complexes^79^. Complex [Cu(^**Me**^**IPrS**)Cl] (**5a**) is linear (C_(NHC)_–Cu–Cl, 177.0°; C–Cu, 1.871 Å), making it a good model for evaluating %V_*bur*_ of *N*-aryl thiazol-2-ylidene ligands. Thus, the (%V_*bur*_) of (**5a**) is 37.0%, which can be compared with the (%V_*bur*_) of 47.6% determined for [Cu(**IPr**)Cl] (C–Cu–Cl, 176.7°; C–Cu, 1.881 Å)^80^. A graphical representation of the steric mapping is shown in Fig. 3b. Importantly, the X-ray crystallographic analysis revealed the (%V_*bur*_) of 50.2%, 52.0%, 22.6%, 23.1% for each quadrant (Fig. 6). The values can be compared with the (%V_*bur*_) of 55.5%, 39.6%, 39.6%, 55.5% for each quadrant of [Cu(**IPr**)Cl], revealing a “half-umbrella” steric arrangement of *N*-aryl thiazol-2-ylidene ligands.

The Tolman electronic parameter (TEP) has been determined from the CO stretching frequencies of [Rh(^**7**^**IPrS**)(CO)_2_Cl] of *ν*_*sym*_ = 2078.0 cm^−1^ and *ν*_*asym*_ = 2001.4 cm^−1^ (CH_2_Cl_2_, 0.20 M), respectively. This corresponds to a TEP of 2051.9 cm^−1^ as a combined measure of the electronic properties of *N*-aryl thiazol-2-ylidene ligands^81^. These values can be compared with the classical imidazol-2-ylidene IPr (TEP of 2051.5 cm^−1^) and a model cyclic (alkyl)amino)carbene CAAC^Cy^ (TEP of 2048.6 cm^−1^)^28^, indicating strong donor ability of *N*-aryl thiazol-2-ylidenes.

In the same vein, selenourea adducts allow to determine π-backbonding of NHC ligands from the ^77^Se NMR spectra^82–85^. As such, the δ_Se_ values of 375.99 ppm for [Se(^**Me**^**IPrS**)] and 374.88 ppm, 366.70 ppm, 329.96 for [Se(^**6**^**IPrS**)], [Se(^**7**^**IPrS**)], [Se(^**Me**^**IMesS**)] (CDCl_3_), respectively, indicate significantly better π-acceptance of *N*-aryl thiazol-2-ylidenes than imidazol-2-ylidene, **IPr** (δ_Se_ = 90 ppm), as expected from the sulfur substitution.

Moreover, one-bond CH J coupling constants from ^13^C satellites of the ^1^H NMR spectrum give a good prediction of σ-donating properties of NHC ligands^86,87^. The values of 218.70 Hz for ^**Me**^**IPrS** and 218.34 Hz, 218.82 Hz, 219.36 Hz for ^**6**^**IPrS**, ^**7**^**IPrS**, and ^**Me**^**IMesS** (HClO_4_ salts, CDCl_3_), respectively, are consistent with *N*-aryl thiazol-2-ylidenes as strongly σ-donating NHC ligands, which can be compared with imidazol-2-ylidene **IPr** (^1^*J*_CH_ = 223.70 Hz). However, at the same time, *N*-aryl thiazol-2-ylidenes are significantly more π-accepting and feature a distinct “half-umbrella” steric impact.

### Ag–NHC-catalyzed cyclization

**Fig. 7:**
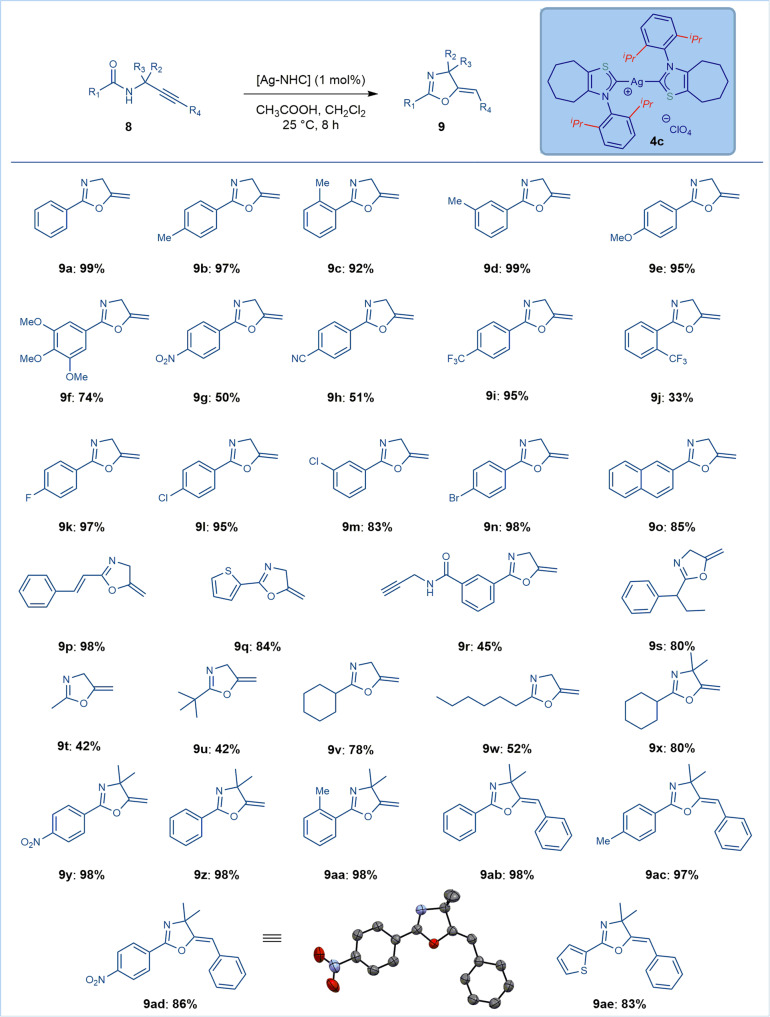
Scope of Ag–NHC-catalyzed cyclization of *N*-propargylic amides. Conditions: amide (1.0 equiv), catalyst **4c** ([Ag], 1 mol%), CH_3_CO_2_H (1.0 equiv), CH_2_Cl_2_ (1.0 M), 25 °C, 8 h. See Supplementary Method 9 for details, **9ad**: CCDC: 2125052.

**Fig. 8:**
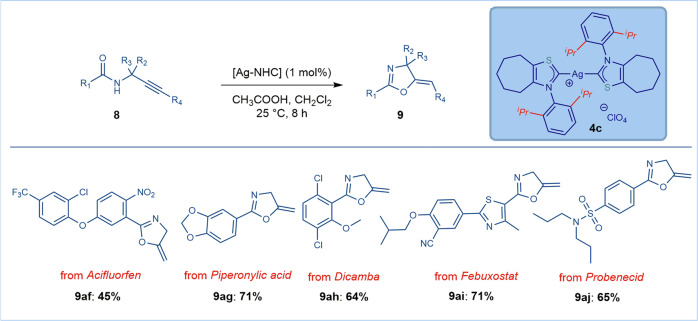
Late-stage functionalization in Ag–NHC-catalyzed cyclization of *N*-propargylic amides. Conditions: amide (1.0 equiv), catalyst **4c** ([Ag], 1 mol%), CH_3_CO_2_H (1.0 equiv), CH_2_Cl_2_ (1.0 M), 25 °C, 8 h. See Supplementary Method 9 for details.

With structural and electronic characterization of *N*-aryl thiazol-2-ylidenes, we next evaluated the activity of Ag(I)–thiazol-2-ylidene complexes in catalysis (Table 1, and Figs. 7–8). As stated above, we selected Ag(I)–NHC complexes because Ag(I) complexes have been much less explored in catalysis than other group 11 metals^77,78^ as well as to probe electrophilic π-activation of the ligands. Electrophilic *O*-cyclization of N-propargylic amides was selected as a model reaction due to the importance of the product oxazoline heterocycles in medicinal chemistry research^88^. As shown, the reaction proceeds under very mild conditions using bis-NHC–Ag(I) salts **4a-d** (5-10 mol%) in the presence of AcOH in CH_2_Cl_2_ at room temperature (Table 1, entries 1-8, see Supplementary Method 8 for details). AcOH is required as an additive (vide infra)^89,90^. Likewise, no reaction takes place in the absence of *N*-aryl thiazol-2-ylidene Ag(I) complexes (Table 1, entries 9-10). Out of the complexes **4a-d**, the cycloheptyl complex [Ag(^**7**^**IPrS**)_2_](ClO_4_) showed the highest reactivity and was selected for scope studies. The loading could be further decreased to 1 mol% with excellent efficiency (>95%) (Table 1, entries 11-14). Most importantly, the use of classical imidazol-2-ylidene complexes [Ag(IPr)Cl] and [(Ag(IMes)Cl] resulted in negligible reactivity (7-16%) (Table 1, entries 15-16), indicating superior reactivity of *N*-aryl thiazol-2-ylidenes. Further comparative studies between thiazol-2-ylidene and IMes/IPr to eliminate the effect of counterion were conducted. Specifically, we also prepared and tested thiazol-2-ylidene [NHC–Ag]_2_PF_6_ (NHC = **3c**, 90% yield), imidazol-2-ylidene [NHC–Ag]_2_ClO_4_ (NHC = IPr, <5% yield, IMes, 30% yield) and imidazol-2-ylidene [NHC–Ag]_2_PF_6_ (NHC = IPr, <5% yield, IMes, 21% yield). These results are consistent with the superior reactivity of *N*-aryl thiazol-2-ylidene Ag(I) complexes (See Supplementary Method 10). As expected, marginal reactivity was observed in the presence of soluble silver salts (AgOTf, 11%; AgSbF_6_, 5%).

**Table 1:** Optimization of Ag–NHC-Catalyzed Cyclization of N-Propargylic Amides^*a*^.


entry	catalyst	additive	mol%	yield (%)
1	**4a**	CH_3_CO_2_H	10	90
2	**4b**	CH_3_CO_2_H	10	95
3	**4c**	CH_3_CO_2_H	10	98
4	**4d**	CH_3_CO_2_H	10	96
5	**4a**	CH_3_CO_2_H	5	90
6	**4b**	CH_3_CO_2_H	5	93
7	**4c**	CH_3_CO_2_H	5	96
8	**4d**	CH_3_CO_2_H	5	91
9	**-**	CH_3_CO_2_H	-	-
10	**4a–4d**	-	10	-
11	**4a**	CH_3_CO_2_H	1	88
12	**4b**	CH_3_CO_2_H	1	85
13	**4c**	CH_3_CO_2_H	1	>95
14	**4d**	CH_3_CO_2_H	1	87
15	[Ag(IPr)Cl]	CH_3_CO_2_H	10	16
16	[Ag(IMes)Cl]	CH_3_CO_2_H	10	7

^*a*^Conditions: **8** (1.0 equiv), Ag–NHC ([Ag], 1-10 mol%), additive (1.0 equiv), CH_2_Cl_2_ (1.0 M), 25 °C, 8 h. See Supplementary Method 8 for details.

Having established the optimal conditions for electrophilic cyclization using *N*-aryl-thiazol-2-ylidene–Ag(I) complexes, next the scope was examined (Fig. 7).

As shown, the scope of the reaction is very broad and encompasses a variety of N-propargylic amides (Fig. 7). As such, aromatic amides with neutral (**9a**), electron-donating (**9b-9f**), electron-withdrawing (**9g-9n**) substituents at the para, ortho and meta positions could be successfully reacted to give diverse 2-aryl-2-oxazolines. Importantly, medicinally-relevant substituents, such as 3,4,5-trimethoxyphenyl (**9** **f**), nitro (**9** **g**), cyano (**9** **h**), trifluoromethyl (**9i-9j**) as well as halide functional handles that enable further derivatization (**9k-9n**) were well compatible with the reaction. Furthermore, π-conjugated substituents, such as naphthyl (**9o**) and cinnamyl (**9p**) furnished the products in high yields. Heterocyclic amides, such as 2-thienyl (**9q**) were well tolerated. Interestingly, highly selective mono-cyclization is possible using meta-substituted propargylic diamide (**9r**). Pleasingly, aliphatic amides with α-branching (**9** **s**) as well as 1° (**9t**, **9w**), 2° (**9** **v**), and 3° sterically-hindered aliphatic amides (**9** **u**) are tolerated in this process despite the lack of Ar→π^*^_C=O_ conjugation with the amide oxygen atom. Furthermore, substitution at the methylene carbon adjacent to the nitrogen to deliver 4-substitued oxazolines is also compatible (**9x-9aa**), increasing the overall synthetic utility of the process. Further, substitution of the alkyne is tolerated without loss in reaction efficiency (**9ab-9ad**), furnishing fully substituted oxazolines. The product **9ad** was crystalline and the structure was confirmed by x-ray crystallography (for more details, see Supplementary Note 1 and Supplementary Data 5), indicating (*Z*)-geometry of the double bond (dr > 98:2). This result is consistent with an anti-attack of the amide bond oxygen on the Ag(I)–NHC-π-activated alkyne (vide infra).

Most crucially, the mild reaction conditions enabled by the *N*-aryl thiazol-2-ylidene ligands permit this mode of catalysis to be applied to late-stage functionalization to furnish attractive heterocyclic building blocks for medicinal chemistry and agrochemistry research (Fig. 8). Thus, electrophilic cyclization of propargylic amides from acifluorfen (**9af**, protoporphyrinogen oxidase inhibitor), piperonylic acid (**9ag**, trans-cinnamate hydroxylase inhibitor), dicamba (**9ah**, broad spectrum herbicide), febuxostat (**9ai**, antigout) and probenecid (**9aj**, antihyperuricemic) delivered cyclization products in good to high yields without modification of the reaction conditions. This successful late-stage diversification highlights the mild conditions of the present protocol with tolerance to an array of sensitive functional groups (halides, cyano, nitro, sulfonamide, aryl ethers, S-heterocycles), demonstrating prospective impact on medicinal chemistry research.

### Mechanism

**Fig. 9:**
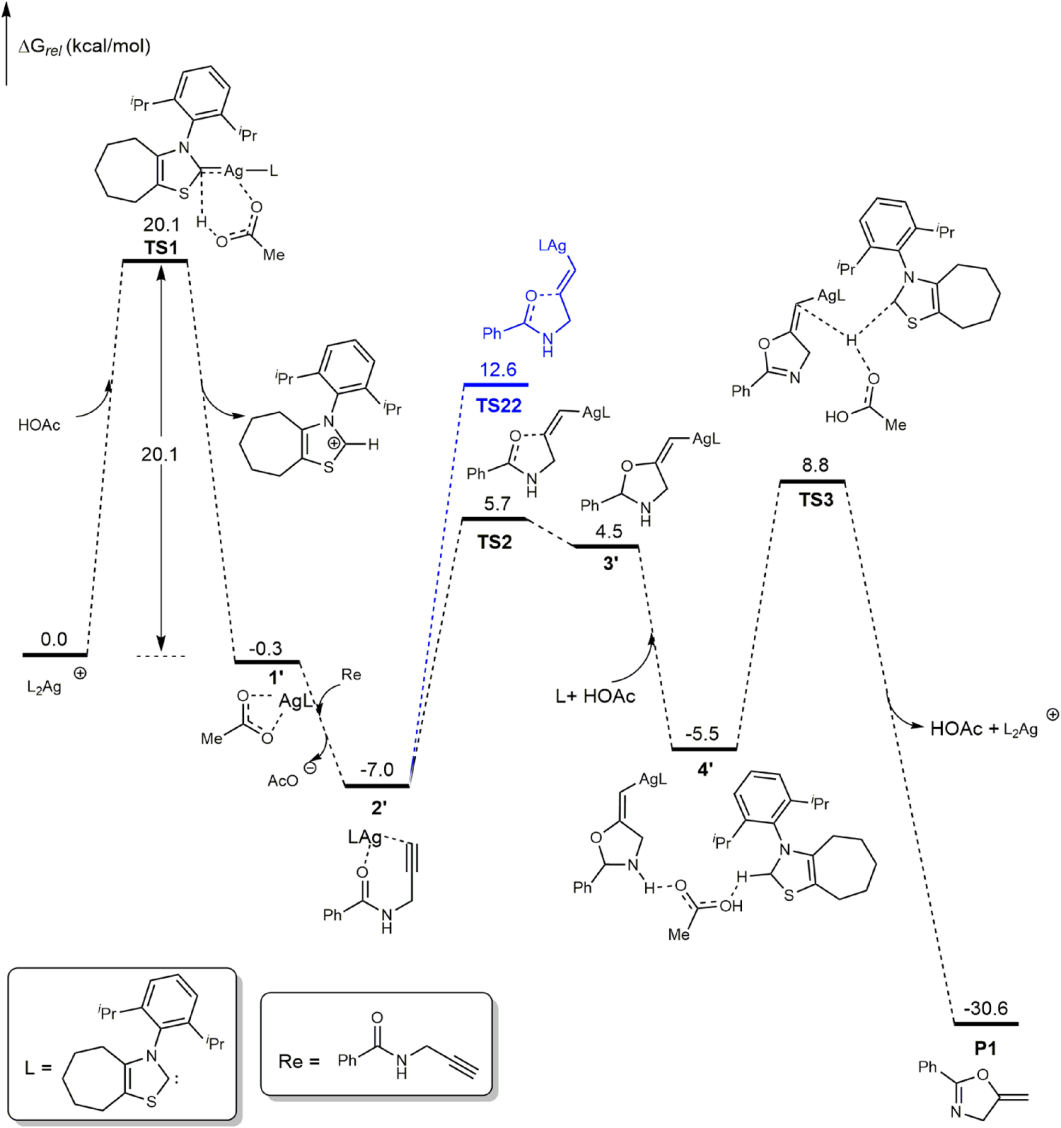
DFT-computed free energy profile of Ag–NHC catalyzed cyclization of *N*-propargylic amides. See Supplementary Note 3 for computational details.

To gain insight into the reaction mechanism of this intriguing transformation, catalytic cycle was studied by DFT computations (Fig. 9). D3 correction has been omitted in optimization. Based on the previous work^89,90^, our calculation results show that the catalytic cycles for these processes are comprised of three key steps. In the first step, L_2_Ag will give active catalyst **1′** in the presence of AcOH. The free energy of activation for this step is 20.1 kcal/mol for **TS1** (Fig. 9). This step is also the rate-determining one for this reaction, and the calculated free energy of activation is in good agreement with the kinetics of the reaction. After formation of active catalyst **1**, the ligand exchange between AcO^-^ and amide **Re** would give intermediate **2** with a free energy release of −6.7 kcal/mol. In second step, **2′** would generate putative vinyl–silver intermediate **3′** by a *5-exo-dig* cyclisation. The free energy of activation for **TS2** (*Z*) and **TS22** (*E*) is 12.7 and 19.6 kcal/mol, respectively. This calculation result for *Z/E* selectivity is fully consistent with the experiments results. The final step involves a 1,4-H shift that leads to the final product **P1**. First, two- and three-molecule HOAc-assisted 1,4-H shift (**TS31** and **TS32)** were calculated. The calculated activation free energy of **TS31** and **TS32** is high (27.4 and 24.5 kcal/mol, See Supplementary Note 3 for details) to occur under the experimental conditions. Another possible pathway involves HOAc and ligand assisted 1,4-H shift. In this pathway, coordination of HOAc and ligand with **3′** would generate intermediate **4′** with a free energy release of −10.0 kcal/mol. The protodemetallation step would form the product **P1** and regenerate the silver catalyst and HOAc. This step is exergonic by −30.6 kcal/mol and the free energy barrier is 15.8 kcal/mol.

**Fig. 10:**
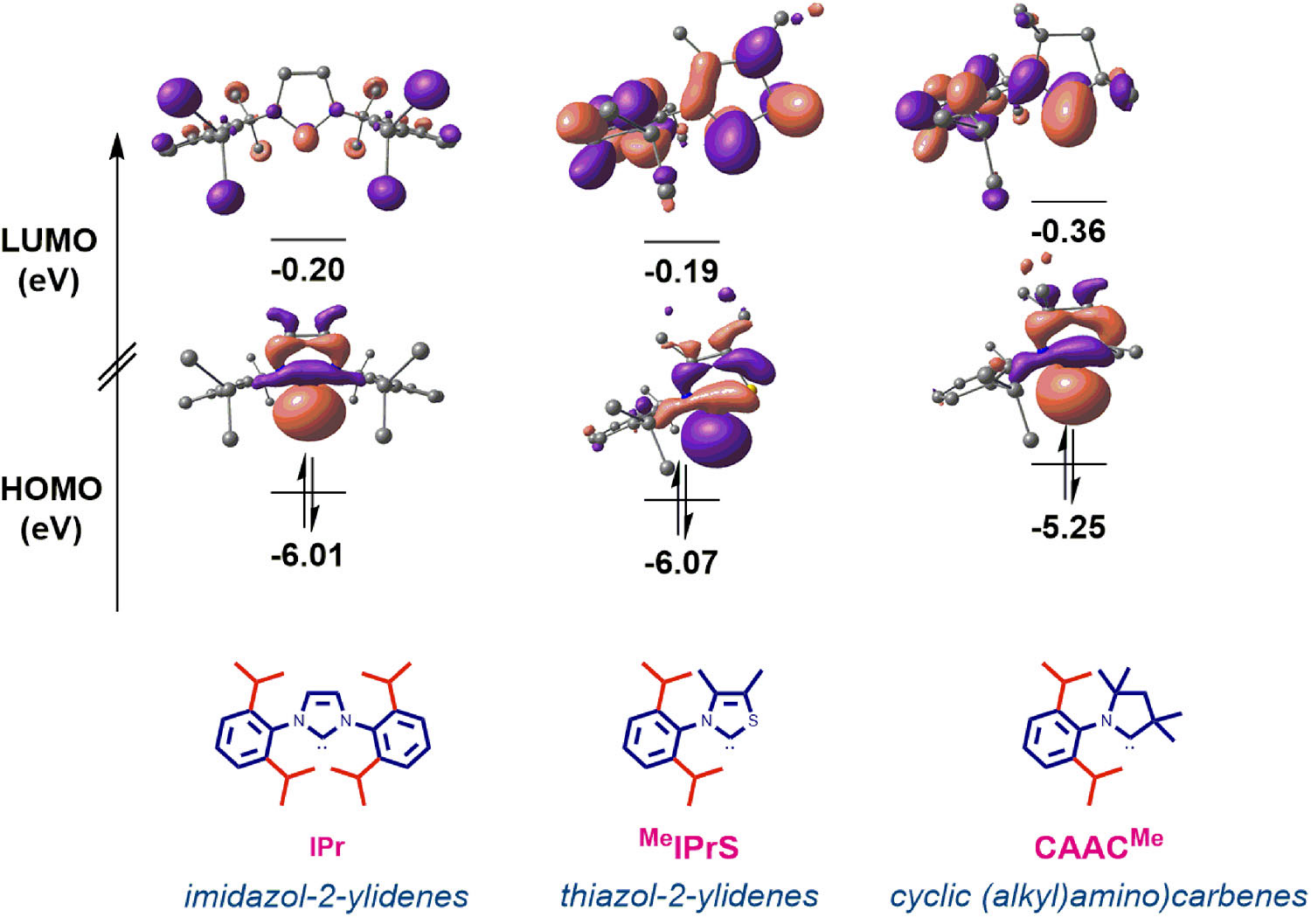
HOMO and LUMO energy levels (eV) calculated at B3LYP 6-311 + +g(d,p). See Supplementary Note 3 for details.

To further evaluate the effect of nitrogen to sulfur replacement in *N*-aryl thiazol-2-ylidenes, HOMO and LUMO energy levels of carbenes ^**Me**^**IPrS**, ^**6**^**IPrS**, ^**7**^**IPrS** and ^**Me**^**IMesS** were determined at the B3LYP 6-311 + +g(d,p) level (Fig. 10 and See Supplementary Note 3 for details). It is now recognized that the donating ability of carbenes is closely associated with the HOMO orbital, while the electron acceptance is associated with the LUMO orbital^15,16,28–35,81^. Computation of frontier orbitals represents the most accurate evaluation of nucleophilicity (higher energy of HOMO) and electrophilicity (lower energy of LUMO) of NHC ligands^15,16,28–35,81^, while the comparison must be available at the same level of theory.

The HOMO of ^**Me**^**IPrS** (−6.07 eV, σ-bonding orbital) is comparable with **IPr** (−6.01 eV), which is a routine model for σ-donating NHCs. The HOMO of ^**6**^**IPrS**, ^**7**^**IPrS** and ^**Me**^**IMesS** are −6.05 eV, −5.99 eV, −6.02 eV respectively, indicating that these *N*-aryl thiazol-2-ylidenes are similarly strongly nucleophilic as *N*-aryl-imidazol-2-ylidenes. Furthermore, the LUMO + 3 of ^**Me**^**IPrS** (−0.36 eV, π-accepting orbital) is compared with the standard imidazol-2-ylidene ligands **IMes** (−0.03 eV), **IPr** (−0.20 eV) of the corresponding π-accepting orbitals. The LUMO + 3 of ^**6**^**IPrS**, ^**7**^**IPrS** and ^**Me**^**IMesS** are −0.33 eV, −0.29 eV, −0.36 eV, respectively (LUMO + 3 due to required symmetry), indicating that *N*-aryl thiazol-2-ylidenes are as good π-acceptors as the standard imidazolylidene **IMes** and **IPr** ligands. In addition, the HOMO-1 (π-donating orbital) in the series of ^**Me**^**IPrS**, ^**6**^**IPrS**, ^**7**^**IPrS** and ^**Me**^**IMesS** is −6.57 eV, −6.49 eV, −6.44 eV, −6.51 eV, which is in the same range as for the π-donating orbital for the standard imidazolylidene **IMes** (−6.44 eV) and **IPr** (−6.55 eV) determined at the same level of theory. Overall, *N*-aryl thiazol-2-ylidenes can be characterized as π-accepting, σ-donating and sterically-distinct “half umbrella” shaped ligands that are well-poised for electrophilic catalysis^91–95^.

It should be further noted that in terms of the steric profile, %V_*bur*_ of ^**Me**^**IPrS** (37.0%, CuCl complex) is much smaller than that of **IPr** (47.6%, CuCl complex) and **CAAC**^**Et**^ (43.1%, AuCl complex)^79^.

## Conclusions

In conclusion, although N-heterocyclic carbenes represent a dominant direction in ligand development in the last 20 years, the majority of efforts in catalysis have been almost exclusively limited to *N*-aryl-imidazolylidenes, such as IPr. In this study, we reported the study on steric and electronic properties of thiazol-2-ylidenes. We presented comprehensive evaluation of steric, electron-donating and π-accepting properties as well as structural characterization of Ag(I) and Cu(I) complexes of *N*-aryl thiazol-2-ylidenes. The thiazole heterocycle and enhanced π-electrophilicity result in a class of highly active carbene ligands that supersede imidazol-2-ylidenes. We showed that this mode of catalysis can be applied to late-stage drug functionalization to furnish attractive building blocks for medicinal chemistry. The unique electronic properties in combination with steric differentiation in a “half umbrella” shape with the single nitrogen N-wingtip oriented toward the metal open a plethora of possibilities in the development of enhanced arsenal of thiazol-2-ylidene ligands of broad interest in chemical synthesis. Our ongoing studies are focused on expanding the scope of reactions catalyzed by thiazol-2-ylidenes in catalysis using electrophilic group 11 metals as well as comparative studies using other NHC ligands. We believe that the class of *N*-aryl thiazol-2-ylidenes is well poised to make an impact on catalysis via electrophilic mechanisms.

## Methods

### General information

For more details, see Supplementary Method 1.

### General procedure for the synthesis of Ag(I) complexes

An oven-dried vial equipped with a stir bar was charged with *N*-Aryl thiazol-2-ylidenes carbene precursors (1.0 equiv), Ag_2_O (typically, 0.5 equiv) and NaCl (typically, 2.0 equiv). The reaction mixture was placed under a positive pressure of argon and subjected to three evacuation/backfilling cycles under high vacuum. DCM (typically, 0.04 M) was added and the reaction mixture was stirred away from light overnight at room temperature. The reaction mixture was filtered through Celite with DCM as eluent and concentrated under reduced pressure, and dried under high vacuum to afford silver(I) complex. For more details, see Supplementary Method 3.

### General procedure for the synthesis of propargylic amides

An oven-dried 100 ml round-bottomed flask equipped with a stir bar was charged with propargylic amine (1.0 equiv), Et_3_N (typically,1.0 equiv), DMAP (typically,0.02 equiv) and DCM (typically, 0.25 M). The resulting mixture was cooled to 0 °C, and the acid chloride (1.0 equiv) was added. The mixture was stirred for 30 min at 0 °C and 3–12 h at room temperature. H_2_O (typically, 0.33 M) was added, and the aqueous layer extracted with DCM. The combined organic extracts were washed with saturated NaHCO_3_, H_2_O and brine, dried over Na_2_SO_4_ and concentrated in vacuo. The crude products were purified by column chromatography on silica gel (EtOAc/hexanes). For more details, see Supplementary Method 7.

### General procedure for the cyclisation of propargylic amides

An oven-dried vial equipped with a stir bar was charged with propargylic amides (1.0 equiv), Ag catalyst **4c** (typically, 1.0 mol%). The reaction mixture was placed under a positive pressure of argon and subjected to three evacuation/backfilling cycles under high vacuum. Then AcOH (typically, 1.0 equiv) and DCM (typically, 1.0 M) was added and the reaction mixture was stirred at room temperature for 8 h. The volatiles were removed in vacuo and the products were purified by column chromatography on silica gel (EtOAc/hexanes). For more details, see Supplementary Method 9.

### Compound characterization

See supplementary note 2 for NMR spectra.

## Supplementary information

Description of Additional Supplementary Files

Supplementary Information

Supplementary Data 1

Supplementary Data 2

Supplementary Data 3

Supplementary Data 4

Supplementary Data 5

## Data Availability

The authors declare that all data supporting the findings of this study, including Experimental procedures, characterization data, computational details, coordinates and energies are available within this article and its Supplementary Information. Data are also available from the corresponding author on request. The X-ray crystallographic coordinates for structures of **4a**-**4c**, **5a** and **9ad** reported in this study have been deposited at the Cambridge Crystallographic Data Centre (CCDC), under deposition numbers 2117719, 2117722, 2117721, 2117739, 2125052. These data can be obtained free of charge from The Cambridge Crystallographic Data Centre via www.ccdc.cam.ac.uk/data_request/cif. The CIF files of CCDC 2117719, CCDC 2117722, CCDC 2117721, CCDC 2117739 and CCDC 2125052 are also included as Supplementary Data 1–5.

## References

[1] Arduengo AJ, Harlow RL, Kline M (1991). A stable crystalline carbene. J. Am. Chem. Soc..

[2] Herrmann WA, Elison M, Fischer J, Köcher C, Artus GRJ (1995). Metal Complexes of N-Heterocyclic Carbenes—A New Structural Principle for Catalysts in Homogeneous Catalysis. Angew. Chem. Int. Ed..

[3] Igau A, Grutzmacher H, Baceiredo A, Bertrand G (1988). Analogous α,α‘-bis-carbenoid, triply bonded species: synthesis of a stable λ^3^-phosphino carbene-λ^5^-phosphaacetylene. J. Am. Chem. Soc..

[4] Martin D, Melaimi M, Soleilhavoup M, Bertrand G (2011). A Brief Survey of Our Contribution to Stable Carbene Chemistry. Organometallics.

[5] Bellotti P, Koy M, Hopkinson MN, Glorius F (2021). Recent advances in the chemistry and applications of N-heterocyclic carbenes. Nat. Rev. Chem..

[6] Hopkinson MN, Richter C, Schedler M, Glorius F (2014). An overview of N-heterocyclic carbenes. Nature.

[7] Cazin, C. S. J., Ed. *N-Heterocyclic Carbenes in Transition Metal Catalysis* (Springer: New York, 2011).

[8] Diez-Gonzalez, S., Ed. *N-Heterocyclic Carbenes: From Laboratory Curiosities to Efficient Synthetic Tools* (RSC: Cambridge, 2016).

[9] Huynh, H. V. *The Organometallic Chemistry of N-Heterocyclic Carbenes* (Wiley: Hoboken, 2017).

[10] Hermann WA (2002). N-Heterocyclic carbenes: A new concept in organometallic catalysis. Angew. Chem. Int. Ed..

[11] Peris E (2018). Smart N-Heterocyclic carbene ligands in catalysis. Chem. Rev..

[12] Iglesias M, Oro LA (2018). A leap forward in iridium–NHC catalysis: New horizons and mechanistic insights. Chem. Soc. Rev..

[13] Danopoulos AA, Simler T, Braunstein P (2019). N-Heterocyclic Carbene Complexes of Copper, Nickel, and Cobalt. Chem. Rev..

[14] Zhao Q, Meng G, Nolan SP, Szostak M (2020). N-Heterocyclic Carbene Complexes in C–H Activation Reactions. Chem. Rev..

[15] Jacobsen H, Correa A, Poater A, Costabile C, Cavallo L (2009). Understanding the M-(NHC) (NHC = N-heterocyclic carbene) bond. Coord. Chem. Rev..

[16] Dröge T, Glorius F (2010). The Measure of All Rings: N-Heterocyclic Carbenes. Angew. Chem. Int. Ed..

[17] Clavier H, Nolan SP (2010). Percent buried volume for phosphine and N-heterocyclic carbene ligands: steric properties in organometallic chemistry. Chem. Commun..

[18] Gomez-Suarez A, Nelson DJ, Nolan SP (2017). Quantifying and understanding the steric properties of N-heterocyclic carbenes. Chem. Commun..

[19] Vougioukalakis GC, Grubbs RH (2010). Ruthenium-Based Heterocyclic Carbene-Coordinated Olefin Metathesis Catalysts. Chem. Rev..

[20] Ogba OM, Warner NC, O’Leary DJ, Grubbs RH (2018). Recent advances in ruthenium-based olefin metathesis. Chem. Soc. Rev..

[21] Meijere, A.; Bräse, S.; Oestreich, M., Eds. *Metal-Catalyzed Cross-Coupling Reactions and More* de (Wiley: New York, 2014).

[22] Molander, G. A.; Wolfe, J. P.; Larhed, M., Eds. *Science of Synthesis: Cross-Coupling and Heck-Type Reactions* (Thieme: Stuttgart, 2013).

[23] Colacot, T. J. *New Trends in Cross-Coupling: Theory and Applications* (RSC: Cambridge, 2015).

[24] Arduengo AJ (1999). Looking for stable carbenes: The difficulty of starting anew. Acc. Chem. Res..

[25] Huang J, Nolan SP (1999). Efficient cross coupling of Aryl Chlorides with Aryl Grignard Reagents (Kumada-Corriu Reaction) Mediated by a Palladium/Imidazolium Chloride System. J. Am. Chem. Soc..

[26] Arduengo AJ (1999). Imidazolylidenes, imidazolinylidenes and imidazolidines. Tetrahedron.

[27] Munz D (2018). Pushing Electrons—Which Carbene Ligand for Which Application?. Organometallics.

[28] Lavallo V, Canac Y, Präsang C, Donnadieu B, Bertrand G (2005). Stable Cyclic (Alkyl)Amino)Carbenes as Rigid or Flexible, Bulky, Electron-Rich Ligands for Transition-Metal Catalysis: A Quaternary Carbon Atom Makes the Difference. Angew. Chem. Int. Ed..

[29] Martin D, Lassauque N, Donnadieu B, Bertrand G (2012). A Cyclic Diaminocarbene with a Pyramidalized Nitrogen Atom: A Stable N-Heterocyclic Carbene with Enhanced Electrophilicity. Angew. Chem..

[30] Weinstein C (2018). Highly Ambiphilic Room Temperature Stable Six-Membered Cyclic (Alkyl)(amino)carbenes. J. Am. Chem. Soc..

[31] Melaimi M, Soleihavoup M, Bertrand G (2010). Angew. Chem. Int. Ed..

[32] Soleilhavoup M, Bertrand G (2015). Cyclic (Alkyl)(Amino)Carbenes (CAACs): Stable Carbenes on the Rise. Acc. Chem. Res..

[33] Melaimi M, Jazzar R, Soleilhavoup M, Bertrand G (2017). Cyclic (Alkyl)(amino)carbenes (CAACs): Recent Developments. Angew. Chem. Int. Ed..

[34] Jazzar R, Soleilhavoup M, Bertrand G (2020). Cyclic (Alkyl)- and (Aryl)-(amino)carbene Coinage Metal Complexes and Their Applications. Chem. Rev..

[35] Morvan J, Mauduit M, Bertrand G, Jazzar R (2021). Cyclic (Alkyl)(amino)carbenes (CAACs) in Ruthenium Olefin Metathesis. ACS Catal..

[36] Hudnall TW, Bielawski CW (2009). An N,N′-Diamidocarbene: Studies in C−H Insertion, Reversible Carbonylation, and Transition-Metal Coordination Chemistry. J. Am. Chem. Soc..

[37] Moerdyk JP, Bielawski CW (2012). Diamidocarbenes as versatile and reversible [2 + 1] cycloaddition reagents. Nat. Chem..

[38] Moerdyk JP, Schilter D, Bielawski CW (2016). N,N′-Diamidocarbenes: Isolable Divalent Carbons with Bona Fide Carbene Reactivity. Acc. Chem. Res..

[39] Mathew P, Neels A, Albrecht M (2008). 1,2,3-Triazolylidenes as Versatile Abnormal Carbene Ligands for Late Transition Metals. J. Am. Chem. Soc..

[40] Donnelly KF, Petronilho A, Albrecht M (2013). Application of 1,2,3-triazolylidenes as versatile NHC-type ligands: synthesis, properties, and application in catalysis and beyond. Chem. Commun..

[41] Guisado-Barrios G, Soleilhavoup M, Bertrand G (2018). 1H-1,2,3-Triazol-5-ylidenes: Readily available Mesoionic Carbenes. Acc. Chem. Res..

[42] Schuster O, Yang LR, Raubenheimer HG, Albrecht M (2009). Beyond Conventional N-Heterocyclic Carbenes: Abnormal, Remote, and Other Classes of NHC Ligands with Reduced Heteroatom Stabilization. Chem. Rev..

[43] Crabtree RH (2013). Abnormal, Mesoionic and Remote N-Heterocyclic Carbene Complexes. Coord. Chem. Rev..

[44] Vivancos Á, Segarra C, Albrecht M (2018). Mesoionic and Related Less Heteroatom-Stabilized N-Heterocyclic Carbene Complexes: Synthesis, Catalysis, and Other Applications. Chem. Rev..

[45] Sau SC, Hota PK, Mandal SK, Soleilhavoupb M, Bertrand G (2020). Stable Abnormal N-Heterocyclic Carbenes and Their Applications. Chem. Soc. Rev..

[46] Krahulic KE, Enright GD, Parvez M, Roesler R (2005). A Stable N-Heterocyclic Carbene with a Diboron Backbone. J. Am. Chem. Soc..

[47] Despagnet-Ayoub E, Grubbs RH (2004). A stable four-membered N-Heterocyclic Carbene. J. Am. Chem. Soc..

[48] Chen WC (2017). Carbodicarbenes: Unexpected π-accepting ability during reactivity with small molecules. J. Am. Chem. Soc..

[49] III Arduengo AJ, Goerlich JR, Marshall WJ (1997). A Stable Thiazol-2-ylidene and Its Dimer. Liebigs Ann.

[50] Vougioukalakis GC, Grubbs RH (2008). Synthesis and activity of ruthenium olefin metathesis catalysts coordinated with Thiazol-2-ylidene Ligands. J. Am. Chem. Soc..

[51] Huynh HV, Meier N, Pape T, Hahn FE (2006). Benzothiazolin-2-ylidene Complexes of Iridium(I). Organometallics.

[52] Ding N, Hor TSA (2010). Ruthenium(ii) N,S-heterocyclic carbene complexes and transfer hydrogenation of ketones. Dalton Trans..

[53] Ding N, Zhang W, Hor TSA (2012). One-step entry to olefin-tethered N,S-heterocyclic carbene complexes of ruthenium with mixed ligands. Dalton Trans..

[54] Schöffler AL, Makarem A, Rominger F, Straub BF (2016). Dinuclear thiazolylidene copper complex as highly active catalyst for azid-alkyne cycloadditons. Beilstein J. Org. Chem..

[55] Ishii T, Ota K, Nagao K, Ohmiya H (2019). N-Heterocyclic Carbene-Catalyzed Radical Relay Enabling Vicinal Alkylacylation of Alkenes. J. Am. Chem. Soc..

[56] Ishii T, Kakeno Y, Nagao K, Ohmiya H (2019). N-Heterocyclic Carbene-catalyzed decarboxylative alkylation of aldehydes. J. Am. Chem. Soc..

[57] Hirano K, Biju AT, Piel I, Glorius F (2009). N-Heterocyclic Carbene-catalyzed hydroacylation of unactivated double bonds. J. Am. Chem. Soc..

[58] Biju AT, Wurz NE, Glorius F (2010). N-Heterocyclic Carbene-catalyzed cascade reaction involving the hydroacylation of unactivated alkynes. J. Am. Chem. Soc..

[59] Biju AT, Glorius F (2010). Intermolecular N-heterocyclic carbene catalyzed hydroacylation of arynes. Angew. Chem. Int. Ed..

[60] Piel I, Pawelczyk MD, Hirano K, Fröhlich R, Glorius F (2011). A Family of Thiazolium Salt Derived N-Heterocyclic Carbenes (NHCs) for Organocatalysis: Synthesis, Investigation and Application in Cross-Benzoin Condensation. Eur. J. Org. Chem.

[61] Finney EE, Ogawa KA, Boydston AJ (2012). Organocatalyzed anodic oxidation of aldehydes. J. Am. Chem. Soc..

[62] Paul M (2018). Breslow Intermediates from Aromatic N-Heterocyclic Carbenes (Benzimidazolin-2-ylidenes, Thiazolin-2-ylidenes). Angew. Chem. Int. Ed..

[63] Paul M, Neudörfl JM, Berkessel A (2019). Breslow Intermediates from a Thiazolin-2-ylidene and Fluorinated Aldehydes: XRD and Solution-Phase NMR Spectroscopic Characterization. Angew. Chem. Int. Ed..

[64] Kluger R, Tittmann K (2008). Thiamin diphosphate catalysis: Enzymic and nonenzymic covalent intermediates. Chem. Rev..

[65] Breslow R (1958). On the Mechanism of Thiamine Action. IV. Evidence from studies on model systems. J. Am. Chem. Soc..

[66] Bourissou D, Guerret O, Gabbaï FP, Bertrand G (2000). Stable Carbenes. Chem. Rev..

[67] Hahn FE, Jahnke MC (2008). Heterocyclic Carbenes: Synthesis and coordination chemistry. Angew. Chem. Int. Ed..

[68] Soleilhavoup M, Bertrand G (2020). Stable Carbenes, Nitrenes, Phosphinidenes, and Borylenes. Chem.

[69] Gusev DG (2009). Donor properties of a series of two-electron ligands. Organometallics.

[70] Shi S, Nolan SP, Szostak M (2018). Well-Defined Palladium(II)-NHC (NHC = N-Heterocyclic Carbene) Precatalysts for Cross-Coupling Reactions of Amides and Esters by Selective Acyl CO–X (X = N, O) Cleavage. Acc. Chem. Res..

[71] Zhao Q (2021). IPr#—highly hindered, broadly applicable N-heterocyclic carbenes. Chem. Sci..

[72] Zhou T (2020). [Pd(NHC)(µ-Cl)Cl]2: Versatile and highly reactive complexes for cross-coupling reactions that avoid formation of inactive Pd(I) off-cycle products. iScience.

[73] Xia Q (2021). [(NHC)PdCl_2_(Aniline)] Complexes: Easily Synthesized, Highly Active Pd(II)−NHC Precatalysts for Cross-Coupling Reactions. J. Org. Chem..

[74] Lei P, Meng G, Szostak M (2017). General method for the Suzuki-Miyaura cross-coupling of amides using commercially available, air- and moisture-stable Palladium/NHC (NHC = N-Heterocyclic Carbene) Complexes. ACS Catal..

[75] Lei P (2017). Suzuki-Miyaura cross-coupling of amides and esters at room temperature: Correlation with barriers to rotation around C–N and C–O Bonds. Chem. Sci..

[76] Chen C, Liu FS, Szostak M (2021). BIAN‐NHC ligands in transition‐metal‐catalysis: A perfect union of sterically encumbered, electronically tunable N‐Heterocyclic Carbenes?. Chem. Eur. J..

[77] Wang Z, Tzouras NV, Nolan SP, Bi X (2021). Silver N-Heterocyclic Carbenes: Emerging Powerful Catalysts. Trends Chem..

[78] Bi, X. “Silver Complexes in Organic Transformations” In *Silver Catalysis in Organic Synthesis*; Li, C. J.; Bi, X., Eds., Wiley-VCH: Weinheim, pp. 661–722 (2019).

[79] Falivene L (2019). Towards the Online Computer-Aided Design of Catalytic Pockets. Nat. Chem..

[80] Mankad NP, Gray TG, Laitar DS, Sadighi JP (2004). Synthesis, Structure, and CO_2_ Reactivity of a Two-Coordinate (Carbene)copper(I) Methyl Complex. Organometallics.

[81] Huynh HV (2018). Electronic properties of N-Heterocyclic carbenes and their experimental determination. Chem. Rev..

[82] Vummaleti SVC (2015). What can NMR spectroscopy of selenoureas and phosphinidenes teach us about the π-accepting abilities of N-heterocyclic carbenes?. Chem. Sci..

[83] Liske A, Verlinden K, Buhl H, Schaper K, Ganter C (2013). Determining the π-Acceptor Properties of N-Heterocyclic Carbenes by Measuring the ^77^Se NMR chemical shifts of their selenium adducts. Organometallics.

[84] Junor GP (2020). The Influence of C(sp^3^)H–Selenium Interactions on the ^77^Se NMR quantification of the π-accepting properties of carbenes. Angew. Chem. Int. Ed..

[85] Back O, Henry-Ellinger M, Martin CD, Martin D, Bertrand G (2013). ^31^P NMR chemical shifts of carbene-phosphinidene adducts as an indicator of the π-accepting properties of carbenes. Angew. Chem. Int. Ed..

[86] Meng G, Kakalis L, Nolan SP, Szostak M (2019). A Simple ^1^H NMR method for determining the σ-Donor properties of N-heterocyclic carbenes. Tetrahedron Lett..

[87] Verlinden K, Buhl H, Frank W, Ganter C (2015). Determining the ligand properties of N-Heterocyclic Carbenes from 77Se NMR parameters. Eur. J. Inorg. Chem.

[88] Zhang HZ, Zhao ZL, Zhou CH (2018). Recent advances in oxazole-based medicinal chemistry. Eur. J. Med. Chem..

[89] Wong VHL (2016). Synthesis, structure and catalytic activity of NHC-Ag(I) carboxylate complexes. Chem. Eur. J..

[90] Su HL (2012). Studies of ligand exchange in N-Heterocyclic Carbene Silver(I) Complexes. Organometallics.

[91] Itoh T, Shimizu Y, Kanai M (2016). Ligand-Enabled, Copper-Catalyzed Regio- and stereoselective synthesis of Trialkylsubstituted Alkenylboronates from Unactivated Internal Alkynes. J. Am. Chem. Soc..

[92] Lee MT, Goodstein MB, Lalic G (2019). Synthesis of Isomerically Pure (*Z*)-Alkenes from Terminal Alkynes and Terminal Alkenes: Silver-Catalyzed Hydroalkylation of Alkynes. J. Am. Chem. Soc..

[93] Cheng L-J, Mankad NP (2021). Copper-catalyzed carbonylative coupling of alkyl halides. Acc. Chem. Res..

[94] Ozawa Y, Endo K, Ito H (2021). Regio- and stereoselective synthesis of Multi-Alkylated Allylic Boronates through three-component coupling reactions between allenes, alkyl halides, and a diboron reagent. J. Am. Chem. Soc..

[95] García-Fernández PD (2020). AuI-Catalyzed Hydroalkynylation of Haloalkynes. J. Am. Chem. Soc..

