# Supplementary material for: Thiazol-2-ylidenes as N-Heterocyclic carbene ligands with enhanced electrophilicity for transition metal catalysis
Source: Commun Chem. 2022 May 6;5:60. doi: 10.1038/s42004-022-00675-7 (PMC9814509; doi:10.1038/s42004-022-00675-7)
Supplement: Supplementary file 1 — Description of Additional Supplementary Files [file 42004_2022_675_MOESM1_ESM.pdf]

Description of Additional Supplementary Files

File Name: Supplementary Data 1

Description: cif of 4a

File Name: Supplementary Data 2

Description: cif of 4b

File Name: Supplementary Data 3

Description: cif of 4c

File Name: Supplementary Data 4

Description: cif of 5a

File Name: Supplementary Data 5

Description: cif of 9ad
