# Supplementary material for: Thiazol-2-ylidenes as N-Heterocyclic carbene ligands with enhanced electrophilicity for transition metal catalysis
Source: Commun Chem. 2022 May 6;5:60. doi: 10.1038/s42004-022-00675-7 (PMC9814509; doi:10.1038/s42004-022-00675-7)
Supplement: Supplementary file 2 — Supplementary Information [file 42004_2022_675_MOESM2_ESM.pdf]

# Supporting Information

# Thiazol-2-ylidenes as N-Heterocyclic Carbene Ligands with Enhanced Electrophilicity for Transition Metal Catalysis

Jin Zhang,[*,†] Tao Li,[†] Xiangyang Li,[†] Anqi Lv,[†] Xue Li,[†] Zheng Wang,[†] Ruihong Wang,[§] Yangmin Ma,[†] Ran Fang,[*,†] Roman Szostak,[Δ] and Michal Szostak[*,‡]

**[†]College of Chemistry and Chemical Engineering, Key Laboratory of Chemical Additives for China National Light Industry, Shaanxi University of Science and Technology, Xi'an 710021, China**

**[§]Institute of Frontier Science and Technology Transfer, Shaanxi University of Science and Technology, Xi'an 710021, China**

**[Δ]Department of Chemistry, Wroclaw University, F. Joliot-Curie 14, Wroclaw 50-383, Poland**

**[‡]Department of Chemistry, Rutgers University, 73 Warren Street, Newark, New Jersey 07102, United States**

## Table of Contents

**Supplementary Methods**

Supplementary Method 1: General information S2

Supplementary Method 2: General Procedure for the Synthesis of Thiazolium Salts S3

Supplementary Method 3: General Procedure for the Synthesis of NHC-Silver Complexes S8

Supplementary Method 4: General Procedure for the Synthesis of NHC-Copper Complexes S11

Supplementary Method 5: General Procedure for the Synthesis of NHC-Rhodium Complexes S12

Supplementary Method 6: General Procedure for the Synthesis of NHC-Selenium Complexes S14

Supplementary Method 7: General Procedure for the Synthesis of Starting Materials S16

Supplementary Method 8: Optimization of the Reaction Conditions S29

Supplementary Method 9: General Procedure for the Cyclisation of Propargylic Amides S30

Supplementary Method 10: Mechanism Studies S41

**Supplementary Notes**

Supplementary Note 1: Crystallographic Studies S42

Supplementary Note 2: $^{1}H$ and $^{13}C$ Spectra S67

Supplementary Note 3: Computational Details S139

Supplementary References S175

# Supplementary Methods

## Supplementary Method 1: General Information

All starting materials reported in the manuscript have been previously described in literature or prepared by the method reported previously unless indicated otherwise. All experiments were performed using standard Schlenk techniques under nitrogen or argon unless stated otherwise. All solvents were purchased at the highest commercial grade and used as received or after purification by passing through activated alumina columns or distillation from sodium/benzophenone under nitrogen. All other chemicals were purchased at the highest commercial grade and used as received. All products were identified using $^{1}$H NMR and $^{13}$C NMR analysis and comparison with authentic samples. Reaction glassware was oven-dried at 140 °C for at least 24 h or flame-dried prior to use, allowed to cool under vacuum and purged with argon (three cycles). All yields refer to yields determined by $^{1}$H NMR using an internal standard (optimization) and isolated yields (scope) unless stated otherwise. $^{1}$H NMR and $^{13}$C NMR spectra were recorded in $CDCl_3$ or DMSO-$d_6$ on a Bruker Ascend spectrometers at 400 ($^{1}$H NMR) and 100 MHz ($^{13}$C NMR) or 600 ($^{1}$H NMR) and 150 MHz ($^{13}$C NMR). All shifts are reported in parts per million (ppm) relative to residual $CHCl_3$ peak (7.26 and 77.16 ppm, $^{1}$H NMR and $^{13}$C NMR, respectively), DMSO-$d_6$ peak (2.50 and 39.52 ppm, $^{1}$H NMR and $^{13}$C NMR, respectively). All coupling constants (*J*) are reported in hertz (Hz). Abbreviations are: s = singlet, brs = broad singlet, d = doublet, t = triplet, q = quartet. Dibromomethane was used as an internal standard to determine NMR yields. Infrared spectra (IR) data were recorded on a Bruker INVENIO spectrometer and recorded in wavenumbers (cm'). High resolution mass spectra were acquired on a Q-Exactive Focus Hybrid Quadrupole-Orbitrap Mass Spectrometer (Thermo Fisher). Powder diffraction data were recorded on a Rigaku SmartLab diffractometer with Cu-K radiation and D/teX Ultra detector covering 3-60° (2$\theta$). All flash chromatography was performed using silica gel, 60 Å, 300 mesh. TLC analysis was carried out on glass plates coated with silica gel 60 F254, 0.2 mm thickness. The plates were visualized with KMnO4 solution or exposure to UV light ($\lambda$ = 254 nm). $^{1}$H NMR, $^{13}$C NMR and MS data are given for all compounds in the Supporting Experimental for characterization purposes.

**Supplementary Method 2: General Procedure for the Synthesis of Thiazolium Salts.**

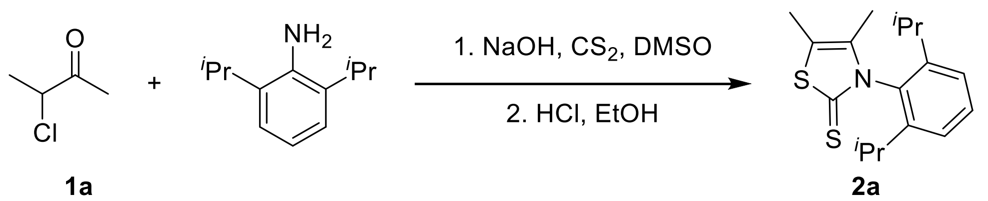


**3-(2,6-Diisopropylphenyl)-4,5-dimethylthiazole-2(3*H*)-thione (2a).** An oven-dried 250 ml round-bottomed flask equipped with a stir bar was charged with 2,6-diisopropylaniline (7.09 g, 40 mmol, 1.0 eq.), 20 N aq. NaOH solution (2 ml, 40 mmol, 1.0 eq.) and DMSO (20 ml). The mixture was cooled to 0 °C and the $CS_2$ (3.05 g, 40 mmol, 1.0 eq) was added dropwise and stirred for 1 h at room temperature. Then the 3-chlorobutan-2-one (4.26 g, 40 mmol, 1.0 eq.) was added at 0 °C and the mixture was stirred for 12 h at room temperature. $H_2O$ (40 ml) was added, the mixture was stirred for 10 min at 0 °C and the supernatant solution was decanted. The resulting slurry was suspended in EtOH (40 ml), concd. HCl (2 ml) was added and the mixture was heated to reflux for 1 h. After cooling to room temperature, the mixture was placed in the fridge, where crystals precipitated. The solid was collected by suction filtration and was washed with a small amount of $H_2O$. The crystallisation afforded the product **2a** as a white solid (8.55 g, 70%). $^1$H NMR (400 MHz, $CDCl_3$) $\delta$ 7.47 (t, $J$ = 7.8 Hz, 1H), 7.30 (d, $J$ = 7.7 Hz, 2H), 2.49 (hept, $J$ = 7.0 Hz, 2H), 2.21 (s, 3H), 1.77 (s, 3H), 1.28 (d, $J$ = 6.8 Hz, 6H), 1.16 (d, $J$ = 6.8 Hz, 6H). $^{13}$C NMR (100 MHz, $CDCl_3$) $\delta$ 187.4, 146.1, 134.9, 133.8, 130.3, 124.6, 117.8, 28.7, 24.5, 23.8, 13.2, 12.0. This compound showed identical spectroscopic properties to those reported previously.[1]

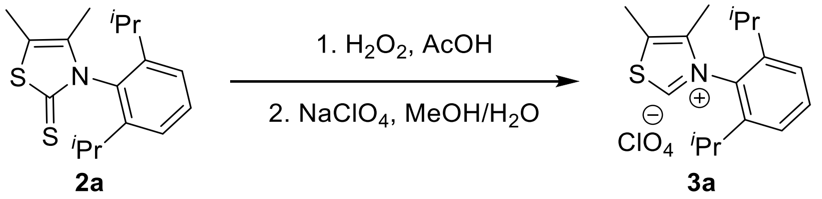


**3-(2,6-Diisopropylphenyl)-4,5-dimethylthiazol-3-ium perchlorate (3a).** An oven-dried 250 ml round-bottomed flask equipped with a stir bar was charged with the thione **2a** (6.11 g, 20 mmol, 1.0 eq.) and glacial acetic acid (82.4 ml) . The mixture was cooled to 0 °C and the $H_2O_2$ (7.48 g, 66 mmol, 3.3 eq.) was added dropwise and stirred for 1 h at 0 °C. Then the volatile components were removed under reduced pressure and the residue was dissolved in MeOH (13.8 ml). At 0 °C a mixture of sodium perchlorate monohydrate (11.57 g, 82.4 mmol, 4.12 eq) in a mixture of MeOH/$H_2O$ = 2/1 (69 ml) was added. After stirring for 30 min, the solid was collected by suction filtration and was washed with $H_2O$ (100 ml). The following recrystallisation with MeOH afforded the product **3a** as a white solid (6.87 g, 83%). $^1$H NMR (400 MHz, DMSO-$d_6$) $\delta$ 10.47 (s, 1H), 7.71 (t, $J$ = 7.8 Hz, 1H), 7.55 (d, $J$ = 7.8 Hz, 2H), 2.65 (s, 3H), 2.09 (s, 3H), 2.07 – 1.99 (m, 2H), 1.18 (d, $J$ = 6.8 Hz, 6H), 1.12 (d, $J$ = 6.8 Hz, 6H). $^{13}$C NMR (100 MHz, DMSO-$d_6$) $\delta$ 157.2, 144.1, 142.2, 134.9, 132.2, 132.1, 125.1, 28.1, 24.4, 22.6, 12.3, 11.2. This compound showed identical spectroscopic properties to those reported

previously.[1]

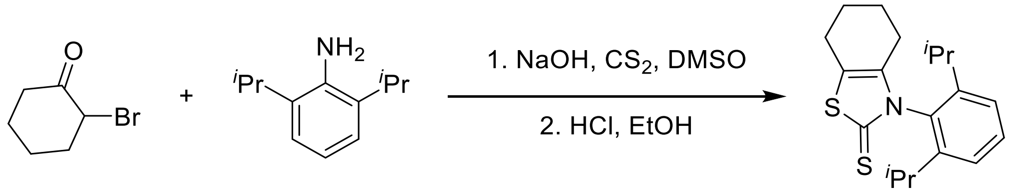


**1b** **2b**

**3-(2,6-Diisopropylphenyl)-4,5,6,7-tetrahydrobenzo[*d*]thiazole-2(3*H*)-thione (2b).** An oven-dried 250 ml round-bottomed flask equipped with a stir bar was charged with 2,6-diisopropylaniline (7.09 g, 40 mmol, 1.0 eq.), 20 N aq. NaOH solution (2 ml, 40 mmol, 1.0 eq.) and DMSO (20 ml). The mixture was cooled to 0 °C and the $CS_2$ (3.05 g, 40 mmol, 1.0 eq) was added dropwise and stirred for 1 h at room temperature. Then the 2-bromocyclohexanone (7.08 g, 40 mmol, 1.0 eq.) was added at 0 °C and the mixture was stirred for 12 h at room temperature. $H_2O$ (40 ml) was added, the mixture was stirred for 10 min at 0 °C and the supernatant solution was decanted. The resulting slurry was suspended in EtOH (40 ml), concd. HCl (2 ml) was added and the mixture was heated to reflux for 1 h. After cooling to room temperature, the solid was collected by suction filtration and was recrystallized from EtOH to afford the product **2b** as yellowish crystals (7.46 g, 75%). $^{1}H$ NMR (400 MHz, $CDCl_3$) $\delta$ 7.47 (t, $J$ = 7.7 Hz, 1H), 7.29 (d, $J$ = 7.7 Hz, 2H), 2.52 (dq, $J$ = 13.6, 6.8, 6.2 Hz, 4H), 1.96 (t, $J$ = 5.9 Hz, 2H), 1.85 (p, $J$ = 5.7 Hz, 2H), 1.74 (p, $J$ = 5.7 Hz, 2H), 1.28 (d, $J$ = 6.8 Hz, 6H), 1.16 (d, $J$ = 6.9 Hz, 6H). $^{13}C$ NMR (100 MHz, $CDCl_3$) $\delta$ 187.8, 146.1, 137.7, 132.8, 130.3, 124.6, 121.0, 28.8, 25.1, 24.6, 23.8, 23.3, 22.9, 21.8. This compound showed identical spectroscopic properties to those reported previously.[1]

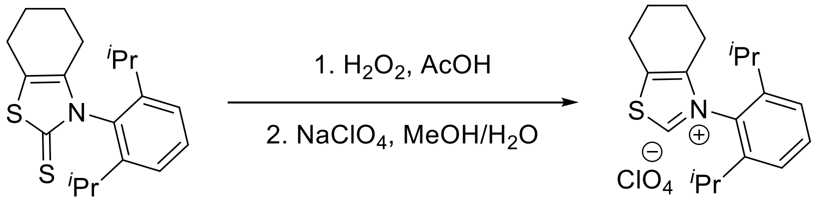


**2b** **3b**

**3-(2,6-Diisopropylphenyl)-4,5,6,7-tetrahydrobenzo[*d*]thiazol-3-ium perchlorate (3b).** An oven-dried 250 ml round-bottomed flask equipped with a stir bar was charged with the thione **2b** (6.63 g, 20 mmol, 1.0 eq.) and glacial acetic acid (82.4 ml) . The mixture was cooled to 0 °C and the $H_2O_2$ (7.48 g, 66 mmol, 3.3 eq.) was added dropwise and stirred for 1 h at 0 °C. Then the volatile components were removed under reduced pressure and the residue was dissolved in MeOH (13.8 ml). At 0 °C a mixture of sodium perchlorate monohydrate (11.57 g, 82.4 mmol, 4.12 eq) in a mixture of MeOH/$H_2O$ = 2/1 (69 ml) was added. After stirring for 30 min, the solid was collected by suction filtration and was washed with $H_2O$ (50 ml). The following recrystallisation with MeOH afforded the product **3b** as a white solid (4.81 g, 80%). $^{1}H$ NMR (400 MHz, $CDCl_3$) $\delta$ 9.87 (s, 1H), 7.61 (t, $J$ = 7.8 Hz, 1H), 7.43 – 7.34 (m, 2H), 3.13 (d, $J$ = 5.5 Hz, 2H), 2.34 – 2.25 (m, 2H), 2.10 (dq, $J$ = 13.2, 6.6 Hz, 2H), 2.05 – 1.97 (m, 2H), 1.97 – 1.89 (m, 2H), 1.21 (d, $J$ = 6.7 Hz, 6H), 1.16 (d, $J$ = 6.7 Hz, 6H). $^{13}C$ NMR (100 MHz, $CDCl_3$) $\delta$ 157.0, 144.6, 144.4, 137.7, 132.5, 131.2, 125.3, 28.8, 23.9, 23.5, 23.1, 21.6,

20.8. This compound showed identical spectroscopic properties to those reported previously.[1]

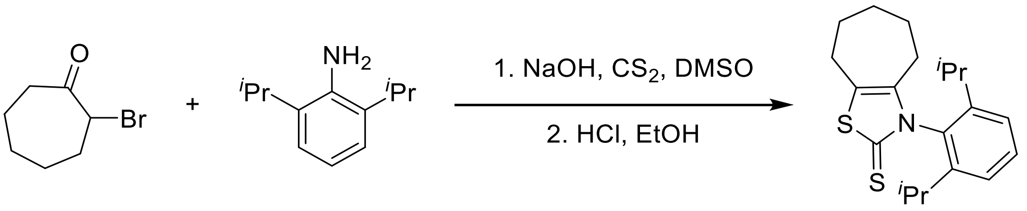


**1c** **2c**

**3-(2,6-Diisopropylphenyl)-3,4,5,6,7,8-hexahydro-2*H*-cyclohepta[*d*]thiazole-2-thione (2c).** An oven-dried 250 ml round-bottomed flask equipped with a stir bar was charged with 2,6-diisopropylaniline (7.09 g, 40 mmol, 1.0 eq.), 20 N aq. NaOH solution (2 ml, 40 mmol, 1.0 eq.) and DMSO (20 ml). The mixture was cooled to 0 °C and the $CS_2$ (3.05 g, 40 mmol, 1.0 eq) was added dropwise and stirred for 1 h at room temperature. Then the 2-bromocycloheptanone (7.64 g, 40 mmol, 1.0 eq.) was added at 0 °C and the mixture was stirred for 12 h at room temperature. $H_2O$ (40 ml) was added, the mixture was stirred for 10 min at 0 °C and the supernatant solution was decanted. The resulting slurry was suspended in EtOH (40 ml), concd. HCl (2 ml) was added and the mixture was heated to reflux for 1 h. After cooling to room temperature, the resulting precipitate was collected by suction filtration and was washed with cold EtOH (20 ml). The crystallisation afforded the product **2c** as a white solid (10.37 g, 75%). $^1$H NMR (400 MHz, $CDCl_3$) $\delta$ 7.46 (t, $J$ = 7.7 Hz, 1H), 7.29 (d, $J$ = 7.4 Hz, 2H), 2.63 (dd, $J$ = 6.4, 3.8 Hz, 2H), 2.46 (hept, $J$ = 7.2 Hz, 2H), 2.24 – 2.17 (m, 2H), 1.78 (q, $J$ = 9.2, 7.9 Hz, 4H), 1.55 (p, $J$ = 5.2 Hz, 2H), 1.28 (dd, $J$ = 7.0, 1.9 Hz, 6H), 1.13 (dd, $J$ = 7.1, 1.9 Hz, 6H). $^{13}$C NMR (100 MHz, $CDCl_3$) $\delta$ 186.6, 146.4, 141.7, 133.7, 130.2, 124.5, 124.0, 30.8, 29.3, 28.7, 27.4, 27.1, 26.3, 24.3, 23.8. This compound showed identical spectroscopic properties to those reported previously.[1]

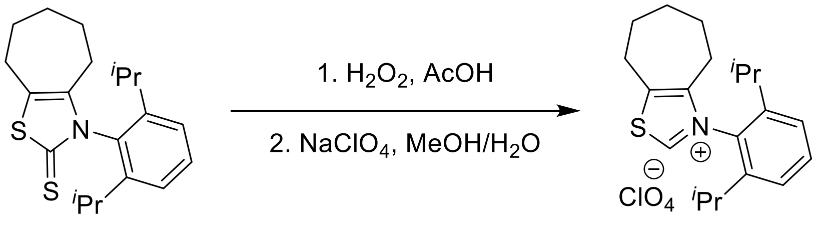


**2c** **3c**

**3-(2,6-Diisopropylphenyl)-5,6,7,8-tetrahydro-4*H*-cyclohepta[*d*]thiazol-3-ium perchlorate (3c).** An oven-dried 250 ml round-bottomed flask equipped with a stir bar was charged with the thione **2c** (6.91 g, 20 mmol, 1.0 eq.) and glacial acetic acid (82.4 ml) . The mixture was cooled to 0 °C and the $H_2O_2$ (7.48 g, 66 mmol, 3.3 eq.) was added dropwise and stirred for 1 h at 0 °C. Then the volatile components were removed under reduced pressure and the residue was dissolved in MeOH (13.8 ml). At 0 °C a mixture of sodium perchlorate monohydrate (11.57 g, 82.4 mmol, 4.12 eq) in a mixture of MeOH/$H_2O$ = 2/1 (69 ml) was added. After stirring for 30 min, the solid was collected by suction filtration and was washed with $H_2O$ (50 ml). The following recrystallisation with MeOH afforded the product **3c** as a white solid (7.14 g, 81%). $^1$H NMR (600 MHz,

DMSO-$d_6$) $\delta$ 10.34 (s, 1H), 7.70 (t, $J$ = 7.8 Hz, 1H), 7.55 (d, $J$ = 7.8 Hz, 2H), 3.23 – 3.13 (m, 2H), 2.57 – 2.51 (m, 2H), 2.05 (hept, $J$ = 6.7 Hz, 2H), 1.92 – 1.83 (m, 2H), 1.82 – 1.73 (m, 2H), 1.62 – 1.52 (m, 2H), 1.16 (d, $J$ = 6.8 Hz, 6H), 1.14 (d, $J$ = 6.8 Hz, 6H). $^{13}$C NMR (150 MHz, DMSO-$d_6$) $\delta$ 155.9, 148.3, 144.5, 140.5, 132.1, 125.1, 28.2, 27.0, 26.4, 26.0, 24.9, 24.3, 22.8. This compound showed identical spectroscopic properties to those reported previously.[1]

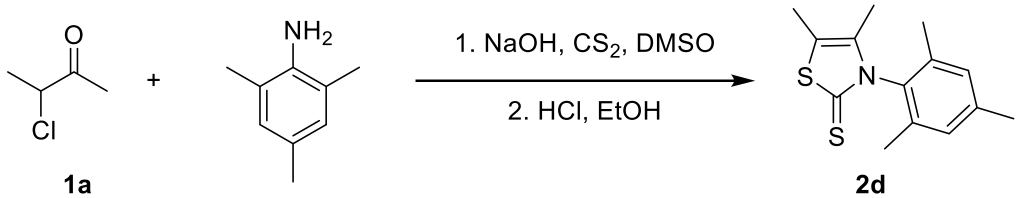


1a 2d

**3-Mesityl-4,5-dimethylthiazole-2(3*H*)-thione (2d).** An oven-dried 250 ml round-bottomed flask equipped with a stir bar was charged with 2,4,6-trimethylaniline (5.41 g, 40 mmol, 1.0 eq.), 20 N aq. NaOH solution (2 ml, 40 mmol, 1.0 eq.) and DMSO (20 ml). The mixture was cooled to 0 °C and the $CS_2$ (3.05 g, 40 mmol, 1.0 eq) was added dropwise and stirred for 1 h at room temperature. Then the 3-chlorobutan-2-one (4.26 g, 40 mmol, 1.0 eq.) was added at 0 °C and the mixture was stirred for 12 h at room temperature. $H_2O$ (40 ml) was added, the mixture was stirred for 10 min at 0 °C and the supernatant solution was decanted. The resulting slurry was suspended in EtOH (40 ml), concd. HCl (2 ml) was added and the mixture was heated to reflux for 1 h. After cooling to room temperature, the mixture was placed in the fridge, where crystals precipitated. The solid was collected by suction filtration and was washed with a small amount of $H_2O$. The crystallisation afforded the product **2d** as a white solid (5.27 g, 55%). $^{1}$H NMR (400 MHz, $CDCl_3$) $\delta$ 7.00 (s, 2H), 2.33 (s, 3H), 2.22 (s, 3H), 2.03 (s, 6H), 1.76 (s, 3H). $^{13}$C NMR (100 MHz, $CDCl_3$) $\delta$ 186.1, 139.5, 135.3, 134.1, 134.1, 129.6, 118.1, 21.3, 17.6, 12.8, 12.0. This compound showed identical spectroscopic properties to those reported previously.[1]

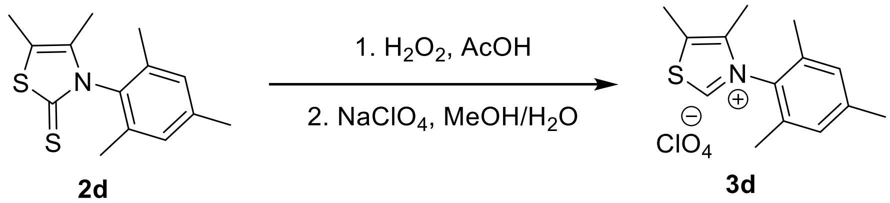


2d 3d

**3-Mesityl-4,5-dimethylthiazol-3-ium perchlorate (3d).** An oven-dried 250 ml round-bottomed flask equipped with a stir bar was charged with the thione **2d** (5.27 g, 20 mmol, 1.0 eq.) and glacial acetic acid (82.4 ml) . The mixture was cooled to 0 °C and the $H_2O_2$ (7.48 g, 66 mmol, 3.3 eq.) was added dropwise and stirred for 1 h at 0 °C. Then the volatile components were removed under reduced pressure and the residue was dissolved in MeOH (13.8 ml). At 0 °C a mixture of sodium perchlorate monohydrate (11.57 g, 82.4 mmol, 4.12 eq) in a mixture of MeOH/$H_2O$ = 2/1 (69 ml) was added. After stirring for 30 min, $H_2O$ (100 mL) was added and the mixture was extracted with DCM (3×100 mL). The combined organic layers were dried over $MgSO_4$ and the solvent was removed under reduced pressure. The column chromatography (Silica Gel, n-hexane/ethyl acetate 1:1) afforded the product **3d** as a less yellow solid

(4.65 g, 70%). $^1$H NMR (400 MHz, $CDCl_3$) $\delta$ 9.69 (s, 1H), 7.07 (s, 2H), 2.69 (s, 3H), 2.37 (s, 3H), 2.14 (s, 3H), 1.96 (s, 6H). $^{13}$C NMR (100 MHz, $CDCl_3$) $\delta$ 156.0, 142.1, 135.3, 133.8, 132.8, 130.3, 21.2, 17.2, 13.0, 11.4. This compound showed identical spectroscopic properties to those reported previously.[1]

**Supplementary Method 3: General Procedure for the Synthesis of NHC-Silver Complexes.**

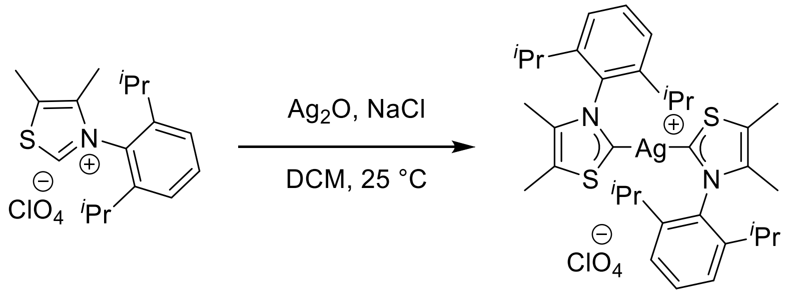


**3a** **4a**

**Bis(3-(2,6-diisopropylphenyl)-4,5-dimethylthiazol-2(3*H*)-ylidene)silver(I) perchlorate (4a).** An oven-dried vial equipped with a stir bar was charged with 3-(2,6-Diisopropylphenyl)-4,5-dimethylthiazol-3-ium perchlorate **(3a)** (74.8 mg, 0.2 mmol, 1.0 eq.), $Ag_2O$ (23.2 mg, 0.1 mmol, 0.5 eq.) and NaCl (23.4 mg, 0.4 mmol, 2.0 eq.). The reaction mixture was placed under a positive pressure of argon and subjected to three evacuation/backfilling cycles under high vacuum. DCM (5 ml, 0.04 M) was added and the reaction mixture was stirred away from light overnight at room temperature. The reaction mixture was filtered through Celite with DCM as eluent and concentrated under reduced pressure, and dried under high vacuum to afford silver(I) complex **4a** as a white solid (72.4 mg, 96%). $^1$H NMR (600 MHz, $CDCl_3$) $\delta$ 7.48 (t, $J$ = 7.8 Hz, 2H), 7.23 (d, $J$ = 7.8 Hz, 4H), 2.49 (s, 6H), 1.97 – 1.89 (m, 10H), 1.11 (d, $J$ = 6.9 Hz, 12H), 0.86 (d, $J$ = 6.8 Hz, 12H). $^{13}$C NMR (150 MHz, $CDCl_3$) $\delta$ 204.1, 204.0, 202.8, 144.4, 142.7, 142.6, 133.5, 133.4, 131.0, 124.8, 28.1, 25.2, 23.0, 12.6, 12.3. HRMS calcd for $C_{34}H_{46}AgN_2S_2^+$ $[M]^+$ 653.2148, found 653.2130.

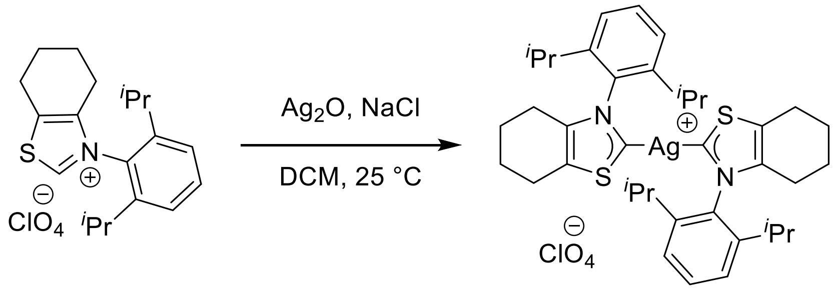


**3b** **4b**

**Bis(3-(2,6-diisopropylphenyl)-4,5,6,7-tetrahydrobenzo[*d*]thiazol-2(3*H*)-ylidene)silver(I) perchlorate (4b).** An oven-dried vial equipped with a stir bar was charged with 3-(2,6-Diisopropylphenyl)-4,5,6,7-tetrahydrobenzo[*d*]thiazol-3-ium perchlorate **(3b)** (80.1 mg, 0.2 mmol, 1.0 eq.), $Ag_2O$ (23.2 mg, 0.1 mmol, 0.5 eq.) and NaCl (23.4 mg, 0.4 mmol, 2.0 eq.). The reaction mixture was placed under a positive pressure of argon and subjected to three evacuation/backfilling cycles under high vacuum. DCM (5 ml, 0.04 M) was added and the reaction mixture was stirred away from light overnight at room temperature. The reaction mixture was filtered through Celite with DCM as eluent and concentrated under reduced pressure, and dried under high vacuum to afford silver(I) complex **4b** as a white solid (76.6 mg, 95%). $^1$H NMR

(400 MHz, $CDCl_3$) $\delta$ 7.47 (t, $J$ = 7.8 Hz, 2H), 7.22 (d, $J$ = 7.8 Hz, 4H), 2.89 (d, $J$ = 5.9 Hz, 4H), 2.12 (t, $J$ = 5.5 Hz, 4H), 1.95 (dq, $J$ = 13.6, 6.8 Hz, 4H), 1.89 – 1.82 (m, 4H), 1.82 – 1.74 (m, 4H), 1.10 (d, $J$ = 6.8 Hz, 12H), 0.84 (d, $J$ = 6.8 Hz, 12H). $^{13}$C NMR (100 MHz, $CDCl_3$) $\delta$ 204.9, 202.9, 144.9, 144.8, 144.5, 136.4, 135.8, 135.7, 131.0, 124.8, 28.2, 25.3, 24.3, 23.7, 23.0, 21.9, 21.4. HRMS calcd for $C_{38}H_{50}AgN_2S_2^+$ $[M]^+$ 705.2461, found 705.2459.

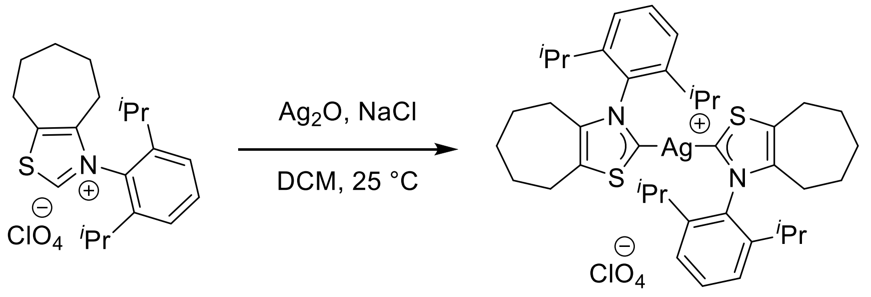


**3c** **4c**

**Bis(3-(2,6-diisopropylphenyl)-3,4,5,6,7,8-hexahydro-2*H*-cyclohepta[*d*]thiazol-2-ylidene)silver(I) perchlorate (4c).** An oven-dried vial equipped with a stir bar was charged with 3-(2,6-Diisopropylphenyl)-5,6,7,8-tetrahydro-4*H*-cyclohepta[*d*]thiazol-3-ium perchlorate **(3c)** (82.8 mg, 0.2 mmol, 1.0 eq.), $Ag_2O$ (23.2 mg, 0.1 mmol, 0.5 eq.) and NaCl (23.4 mg, 0.4 mmol, 2.0 eq.). The reaction mixture was placed under a positive pressure of argon and subjected to three evacuation/backfilling cycles under high vacuum. DCM (5 ml, 0.04 M) was added and the reaction mixture was stirred away from light overnight at room temperature. The reaction mixture was filtered through Celite with DCM as eluent and concentrated under reduced pressure, and dried under high vacuum to afford silver(I) complex **4c** as a white solid (80.9 mg, 97%). $^1$H NMR (400 MHz, $CDCl_3$) $\delta$ 7.50 (t, $J$ = 7.8 Hz, 2H), 7.25 (d, $J$ = 7.8 Hz, 4H), 3.03 – 2.94 (m, 4H), 2.47 – 2.37 (m, 4H), 1.99 (p, $J$ = 6.8 Hz, 4H), 1.91 (p, $J$ = 5.7 Hz, 4H), 1.82 (q, $J$ = 6.5, 5.7 Hz, 4H), 1.61 – 1.52 (m, 4H), 1.12 (d, $J$ = 6.8 Hz, 12H), 0.91 (d, $J$ = 6.8 Hz, 12H). $^{13}$C NMR (100 MHz, $CDCl_3$) $\delta$ 203.24, 203.15, 201.95, 201.86, 149.34, 149.29, 144.64, 139.43, 139.39, 137.77, 130.94, 124.73, 30.99, 28.20, 27.94, 27.69, 26.90, 25.80, 25.04, 23.11. HRMS calcd for $C_{40}H_{54}AgN_2S_2^+$ $[M]^+$ 733.2774, found 733.2754.

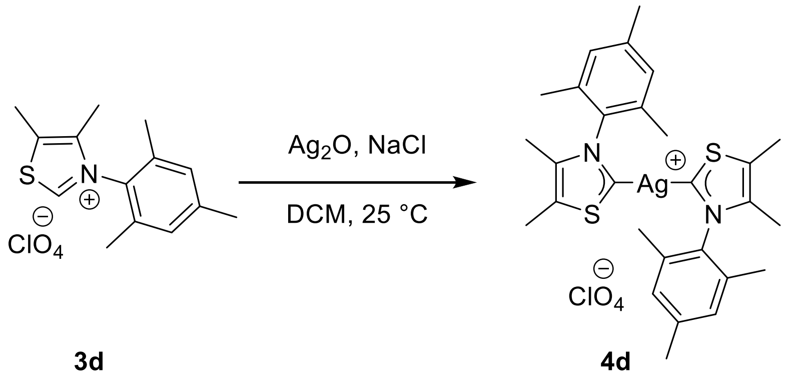


**3d** **4d**

**Bis(3-mesityl-4,5-dimethylthiazol-2(3*H*)-ylidene)silver(I) perchlorate (4d).** An oven-dried vial equipped with a stir bar was charged with 3-mesityl-4,5-

dimethylthiazol-3-ium perchlorate **(3d)** (66.4 mg, 0.2 mmol, 1.0 eq.), $Ag_2O$ (23.2 mg, 0.1 mmol, 0.5 eq.) and NaCl (23.4 mg, 0.4 mmol, 2.0 eq.). The reaction mixture was placed under a positive pressure of argon and subjected to three evacuation/backfilling cycles under high vacuum. DCM (5 ml, 0.04 M) was added and the reaction mixture was stirred away from light overnight at room temperature. The reaction mixture was filtered through Celite with DCM as eluent and concentrated under reduced pressure, and dried under high vacuum to afford silver(I) complex **4d** as a white solid (60.3 mg, 90%). $^1$H NMR (600 MHz, $CDCl_3$) $\delta$ 6.96 (s, 4H), 2.46 (s, 6H), 2.42 (s, 6H), 1.94 (s, 6H), 1.65 (s, 12H). $^{13}$C NMR (150 MHz, $CDCl_3$) $\delta$ 204.6, 203.3, 141.7, 139.7, 138.5, 133.6, 133.4, 129.7, 21.2, 17.2, 12.5, 11.7. HRMS calcd for $C_{38}H_{34}AgN_2S_2^+$ $[M]^+$ 569.1209, found 569.1206.

**Supplementary Method 4: General Procedure for the Synthesis of NHC-Copper Complexes.**

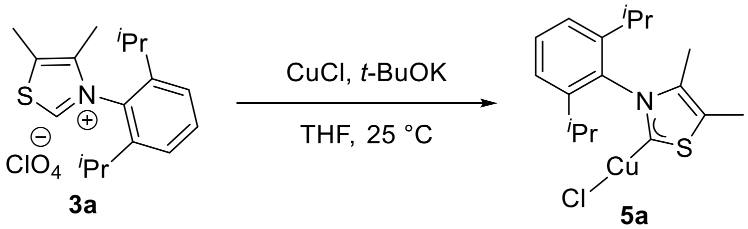


3a 5a

**(3-(2,6-Diisopropylphenyl)-4,5-dimethylthiazol-2(3*H*)-ylidene)copper(I) chloride (5a).** An oven-dried vial equipped with a stir bar was charged with 3-(2,6-Diisopropylphenyl)-4,5-dimethylthiazol-3-ium perchlorate **(3a)** (74.8 mg, 0.2 mmol, 1.0 eq.), CuCl (21.8 mg, 0.22 mmol, 1.1 eq.) and *t*-BuOK (22.4 mg, 0.2 mmol, 1.0 eq.). The reaction mixture was placed under a positive pressure of argon and subjected to three evacuation/backfilling cycles under high vacuum. THF (5 ml, 0.04 M) was added and the reaction mixture was stirred at room temperature for 16 h. The reaction mixture was filtered through Celite with DCM as eluent and concentrated under reduced pressure to afford a brown solid. The crude product was triturated for 1 h in 2.5 ml of DCM, and then 2.5 ml of pentane was added. The supernatant was removed, and the resulting white solid was triturated with pentane (3 × 2 mL) and dried under high vacuum to afford the NHC-Copper(I) complex **5a** as a white solid (43.9 mg, 59%). $^1$H NMR (600 MHz, $CDCl_3$) $\delta$ 7.50 (t, $J$ = 7.7 Hz, 1H), 7.31 (d, $J$ = 7.8 Hz, 2H), 2.43 (s, 3H), 2.11 (dt, $J$ = 13.4, 6.6 Hz, 2H), 1.95 (s, 3H), 1.25 (d, $J$ = 6.6 Hz, 6H), 1.16 (d, $J$ = 6.8 Hz, 6H). $^{13}$C NMR (150 MHz, $CDCl_3$) $\delta$ 201.0, 144.6, 141.4, 137.6, 130.9, 130.7, 124.8, 28.4, 25.3, 23.2, 12.6, 12.2. HRMS calcd for $C_{17}H_{24}NS^+$ [M-CuCl]$^+$ 274.1624, found 274.1614.

**Supplementary Method 5: General Procedure for the Synthesis of NHC-Rhodium Complexes.**

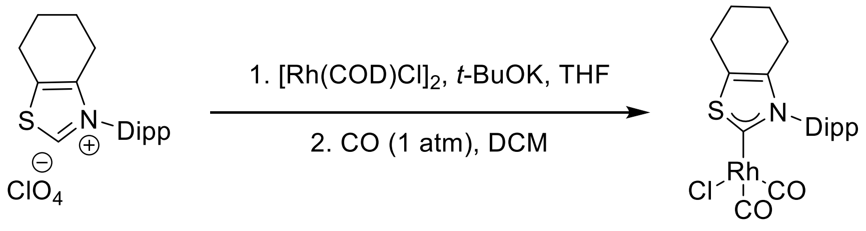


3b 6b

**[(NHC)Rh(CO)$_2$Cl] (6b).** A 25 ml round-bottomed flask was equipped with a septum and a stir bar and flame-dried under a stream of argon. The flask was charged with *t*-BuOK (44.9 mg, 0.4 mmol, 2.0 eq.) and THF (2 ml) and cooled with an ice bath. [Rh(COD)Cl]$_2$ (98.6 mg, 0.2 mmol, 1.0 eq.) was introduced, and the resulting orange solution was stirred for 15 min in the ice bath and then for 45 min at room temperature. The 3-(2,6-Diisopropylphenyl)-4,5,6,7-tetrahydrobenzo[*d*]thiazol-3-ium perchlorate **(3b)** (144.0 mg, 0.36 mmol, 1.8 eq.) was added, and the reaction mixture evolved progressively from orange to yellow. After 8 h, the homogeneous solution was filtered through Celite with DCM as eluent and concentrated under reduced pressure to afford a yellow solid. Then the yellow solid was dissolved in DCM (5 ml, 0.04 M). The solution was cooled to 0 °C and exposed to an atmosphere of CO (flask was equipped with a CO balloon connected via a syringe and a septum). After bubbling CO through the solution for 1 h the solvent was removed and the crude product was washed with pentane twice to afford the pure complex **6b** as an orange solid (93.8 mg, 95%). $^1$H NMR (600 MHz, CDCl$_3$) $\delta$ 7.53 (t, *J* = 7.8 Hz, 1H), 7.32 (d, *J* = 7.8 Hz, 2H), 2.82 (t, *J* = 5.7 Hz, 2H), 2.30 (hept, *J* = 6.5 Hz, 2H), 2.17 (t, *J* = 5.6 Hz, 2H), 1.87 – 1.80 (m, 2H), 1.80 – 1.74 (m, 2H), 1.33 (d, *J* = 6.8 Hz, 6H), 1.13 (d, *J* = 6.8 Hz, 6H). $^{13}$C NMR (150 MHz, CDCl$_3$) $\delta$ 207.2, 206.8, 185.0, 184.6, 181.6, 181.1, 145.2, 144.3, 137.4, 135.1, 131.2, 125.1, 28.4, 25.0, 24.6, 23.9, 23.6, 22.1, 21.8. IR νCO (CH$_2$Cl$_2$, cm$^{-1}$): 2079.0 (vs), 2001.8 (vs).

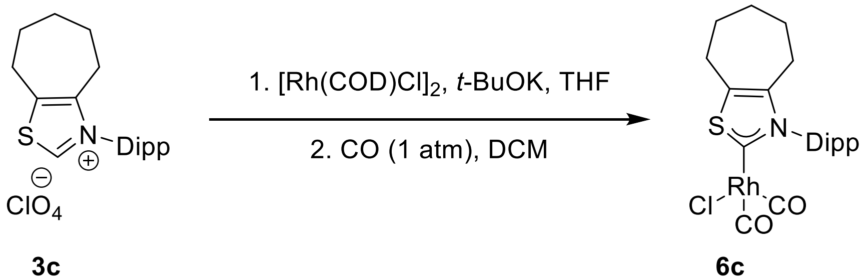


3c 6c

**[(NHC)Rh(CO)$_2$Cl] (6c).** A 25 ml round-bottomed flask was equipped with a septum and a stir bar and flame-dried under a stream of argon. The flask was charged with *t*-BuOK (44.9 mg, 0.4 mmol, 2.0 eq.) and THF (2 ml) and cooled with an ice bath. [Rh(COD)Cl]$_2$ (98.6 mg, 0.2 mmol, 1 eq.) was introduced, and the resulting orange solution was stirred for 15 min in the ice bath and then for 45 min at room temperature. The 3-(2,6-Diisopropylphenyl)-5,6,7,8-tetrahydro-4*H*-cyclohepta[*d*]thiazol-3-ium perchlorate **(3c)** (149.0 mg, 0.36 mmol, 1.8 eq.) was added, and the reaction mixture evolved progressively from orange to yellow. After 8 h, the homogeneous solution was

filtered through Celite with DCM as eluent and concentrated under reduced pressure to afford a yellow solid. Then the yellow solid was dissolved in DCM (5 ml, 0.04 M). The solution was cooled to 0 °C and exposed to an atmosphere of CO (flask was equipped with a CO balloon connected via a syringe and a septum). After bubbling CO through the solution for 1 h the solvent was removed and the crude product was washed with pentane twice to afford the pure complex **6c** as an orange solid (97.5 mg, 96%). $^{1}$H NMR (600 MHz, $CDCl_3$) *δ* 7.53 (t, *J* = 7.8 Hz, 1H), 7.32 (d, *J* = 7.8 Hz, 2H), 2.92 – 2.87 (m, 2H), 2.46 – 2.42 (m, 2H), 2.31 (hept, *J* = 6.8 Hz, 2H), 1.89 – 1.83 (m, 2H), 1.81 – 1.75 (m, 2H), 1.60 – 1.54 (m, 2H), 1.34 (d, *J* = 6.8 Hz, 6H), 1.12 (d, *J* = 6.9 Hz, 6H). $^{13}$C NMR (150 MHz, $CDCl_3$) *δ* 205.7, 205.3, 185.0, 184.7, 181.4, 180.9, 148.6, 145.5, 138.8, 138.6, 131.1, 125.0, 31.2, 28.9, 28.4, 27.9, 26.9, 26.2, 24.4, 24.0. IR νCO ($CH_2Cl_2$, $cm^{-1}$): 2078.0 (vs), 2001.4 (vs).

**Supplementary Method 6:. General Procedure for the Synthesis of NHC-Selenium Complexes.**

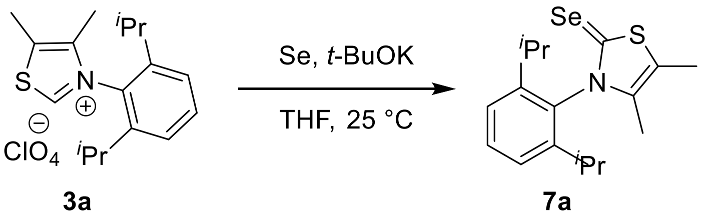


**3-(2,6-Diisopropylphenyl)-4,5-dimethylthiazole-2(3*H*)-selenone (7a).** An oven-dried vial equipped with a stir bar was charged with 3-(2,6-Diisopropylphenyl)-4,5-dimethylthiazol-3-ium perchlorate **(3a)** (186.9 mg, 0.5 mmol, 1.0 eq.), Se (59.2 mg, 0.75 mmol, 1.5 eq.) and *t*-BuOK (67.3 mg, 0.6 mmol, 1.2 eq.). The reaction mixture was placed under a positive pressure of argon and subjected to three evacuation/backfilling cycles under high vacuum. THF (2 ml, 0.1 M) was added and the reaction mixture was stirred at room temperature for 12 h. The reaction mixture was diluted with DCM and concentrated. The product **7a** was obtained by trituration from diethyl ether as a yellow solid (167.4 mg, 95%). $^{1}$H NMR (600 MHz, $CDCl_3$) $\delta$ 7.50 (t, $J$ = 7.7 Hz, 1H), 7.32 (d, $J$ = 7.7 Hz, 2H), 2.42 (hept, $J$ = 6.9 Hz, 2H), 2.23 (s, 3H), 1.85 (s, 3H), 1.32 (d, $J$ = 6.9 Hz, 6H), 1.15 (d, $J$ = 7.2 Hz, 6H). $^{13}$C NMR (150 MHz, $CDCl_3$) $\delta$ 180.5, 145.7, 137.5, 134.8, 130.5, 124.8, 122.5, 28.9, 24.7, 23.9, 13.7, 12.2. $^{77}$Se NMR (114 MHz, $CDCl_3$) $\delta$ 375.99. HRMS calcd for $C_{17}H_{24}NSSe^+$ $[M+H]^+$ 354.0790, found 354.0784.

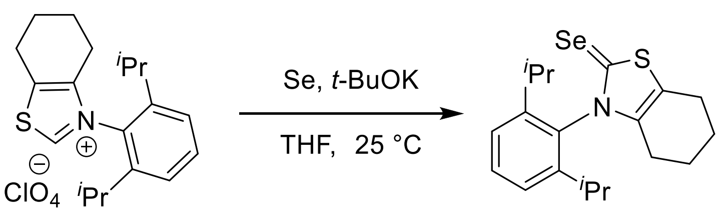


**3-(2,6-Diisopropylphenyl)-4,5,6,7-tetrahydrobenzo[*d*]thiazole-2(3*H*)-selenone (7b).** An oven-dried vial equipped with a stir bar was charged with 3-(2,6-Diisopropylphenyl)-4,5,6,7-tetrahydrobenzo[*d*]thiazol-3-ium perchlorate **(3b)** (199.9 mg, 0.5 mmol, 1.0 eq.), Se (59.2 mg, 0.75 mmol, 1.5 eq.) and *t*-BuOK (67.3 mg, 0.6 mmol, 1.2 eq.). The reaction mixture was placed under a positive pressure of argon and subjected to three evacuation/backfilling cycles under high vacuum. THF (2 ml, 0.1 M) was added and the reaction mixture was stirred at room temperature for 12 h. The reaction mixture was diluted with DCM and concentrated. The product **7b** was obtained by trituration from diethyl ether as a yellow solid (181.7 mg, 96%). $^{1}$H NMR (600 MHz, $CDCl_3$) $\delta$ 7.49 (t, $J$ = 7.8 Hz, 1H), 7.31 (d, $J$ = 7.8 Hz, 2H), 2.56 (t, $J$ = 6.1 Hz, 2H), 2.46 (hept, $J$ = 6.8 Hz, 2H), 2.03 (t, $J$ = 6.1 Hz, 2H), 1.85 (p, $J$ = 6.0 Hz, 2H), 1.73 (p, $J$ = 6.0 Hz, 2H), 1.32 (d, $J$ = 6.8 Hz, 6H), 1.15 (d, $J$ = 6.9 Hz, 6H). $^{13}$C NMR (150 MHz, $CDCl_3$) $\delta$ 180.9, 145.7, 140.3, 133.7, 130.5, 125.5, 124.7, 28.9, 25.4, 24.7, 23.9, 23.5, 22.8, 21.8. $^{77}$Se NMR (114 MHz, $CDCl_3$) $\delta$ 374.88. HRMS calcd for $C_{19}H_{26}NSSe^+$ $[M+H]^+$ 380.0946, found 380.0939.

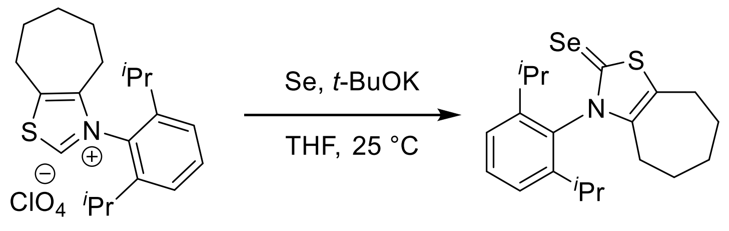


**3c** **7c**

**3-(2,6-Diisopropylphenyl)-3,4,5,6,7,8-hexahydro-2*H*-cyclohepta[*d*]thiazole-2-selenone (7c).** An oven-dried vial equipped with a stir bar was charged with 3-(2,6-Diisopropylphenyl)-5,6,7,8-tetrahydro-4*H*-cyclohepta[*d*]thiazol-3-ium perchlorate **(3c)** (206.9 mg, 0.5 mmol, 1.0 eq.), Se (59.2 mg, 0.75 mmol, 1.5 eq.) and *t*-BuOK (67.3 mg, 0.6 mmol, 1.2 eq.). The reaction mixture was placed under a positive pressure of argon and subjected to three evacuation/backfilling cycles under high vacuum. THF (2 ml, 0.1 M) was added and the reaction mixture was stirred at room temperature for 12 h. The reaction mixture was diluted with DCM and concentrated. The product **7c** was obtained by trituration from diethyl ether as a yellow solid (182.5 mg, 93%). $^1$H NMR (600 MHz, $CDCl_3$) $\delta$ 7.50 (t, $J$ = 7.7 Hz, 1H), 7.31 (d, $J$ = 7.7 Hz, 2H), 2.69 – 2.63 (m, 2H), 2.39 (hept, $J$ = 6.9 Hz, 2H), 2.32 – 2.25 (m, 2H), 1.81 (pd, $J$ = 5.7, 3.7, 2.3 Hz, 2H), 1.79 – 1.72 (m, 2H), 1.56 (ddt, $J$ = 11.5, 8.2, 4.4 Hz, 2H), 1.31 (d, $J$ = 6.7 Hz, 6H), 1.12 (d, $J$ = 6.9 Hz, 6H). $^{13}$C NMR (150 MHz, $CDCl_3$) $\delta$ 179.4, 146.1, 144.4, 134.7, 130.5, 128.7, 124.7, 31.0, 29.7, 28.8, 27.6, 27.1, 26.4, 24.4, 24.0. $^{77}$Se NMR (114 MHz, $CDCl_3$) $\delta$ 366.70. HRMS calcd for $C_{20}H_{28}NSSe^+$ $[M+H]^+$ 394.1103, found 394.1097.

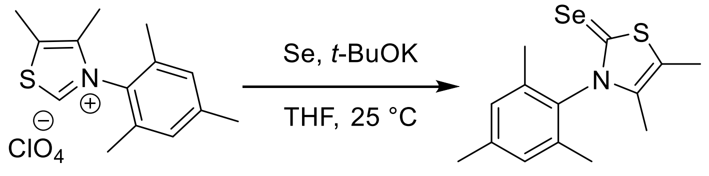


**3d** **7d**

**3-Mesityl-4,5-dimethylthiazole-2(3*H*)-selenone (7d).** An oven-dried vial equipped with a stir bar was charged with 3-mesityl-4,5-dimethylthiazol-3-ium perchlorate **(3d)** (165.9 mg, 0.5 mmol, 1.0 eq.), Se (59.2 mg, 0.75 mmol, 1.5 eq.) and *t*-BuOK (67.3 mg, 0.6 mmol, 1.2 eq.). The reaction mixture was placed under a positive pressure of argon and subjected to three evacuation/backfilling cycles under high vacuum. THF (2 ml, 0.1 M) was added and the reaction mixture was stirred at room temperature for 12 h. The reaction mixture was diluted with DCM and concentrated. The product **7d** was obtained by trituration from diethyl ether as a yellow solid (144.3 mg, 93%). $^1$H NMR (600 MHz, $CDCl_3$) $\delta$ 7.02 (s, 2H), 2.35 (s, 3H), 2.23 (d, J = 1.2 Hz, 3H), 2.02 (s, 6H), 1.83 (s, 3H). $^{13}$C NMR (150 MHz, $CDCl_3$) $\delta$ 178.7, 139.6, 136.6, 135.0, 134.9, 129.6, 122.6, 21.3, 17.7, 13.1, 12.1. $^{77}$Se NMR (114 MHz, $CDCl_3$) 329.96. HRMS calcd for $C_{14}H_{18}NSSe^+$ $[M+H]^+$ 312.0320, found 312.0312.

**Supplementary Method 7: General Procedure for the Synthesis of Starting Materials.**

General procedure for the synthesis of substrates (**8a-8ae**) (Procedure A): An oven-dried 100 ml round-bottomed flask equipped with a stir bar was charged with propargylic amine (5 mmol, 1.0 eq.), $Et_3N$ (0.7 ml, 5 mmol, 1.0 eq.), DMAP (12.2 mg, 0.1 mmol, 0.02 eq.) and DCM (20 ml). The resulting mixture was cooled to 0 °C, and the acid chloride (5 mmol, 1.0 eq.) was added. The mixture was stirred for 30 min at 0 °C and 3-12 h at room temperature. $H_2O$ (15 ml) was added, and the aqueous layer extracted with another 3 × 15 ml of DCM. The combined organic extracts were washed with saturated $NaHCO_3$, $H_2O$ and brine, dried over $Na_2SO_4$ and concentrated in vacuo. The crude product was purified by column chromatography on silica.

***N*-(Prop-2-yn-1-yl)benzamide (8a).**

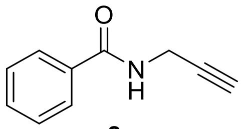

**8a**

Prepared from benzoyl chloride (695.0 mg, 5 mmol) and propargylamine (275.4 mg, 5 mmol), The crude product was purified by column chromatography (petroleum ether/ethyl acetate, 1:1) afford a white solid (756.1 mg, 95%). $^1$H NMR (400 MHz, $CDCl_3$) $\delta$ 7.79 (d, $J$ = 7.1 Hz, 2H), 7.51 (t, $J$ = 7.3 Hz, 1H), 7.43 (t, $J$ = 7.6 Hz, 2H), 6.47 (s, 1H), 4.25 (dd, $J$ = 5.2, 2.6 Hz, 2H), 2.28 (t, $J$ = 2.6 Hz, 1H). These data were compared and found identical to literature values.[2]

**4-Methyl-*N*-(prop-2-yn-1-yl)benzamide (8b).**

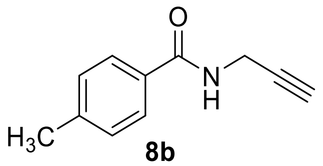

**8b**

Prepared from 4-methylbenzoyl chloride (773.0 mg, 5 mmol) and propargylamine (275.4 mg, 5 mmol), The crude product was purified by column chromatography (petroleum ether/ethyl acetate, 1:1) afford a white solid (778.9 mg, 90%). $^1$H NMR (400 MHz, $CDCl_3$) $\delta$ 7.84 - 7.61 (m, 2H), 7.28 - 7.20 (m, 2H), 6.47 (bs, 1H), 4.26 (dd, $J$ = 5.2, 2.6 Hz, 2H), 2.41 (s, 3H), 2.29 (t, $J$ = 2.6 Hz, 1H). These data were compared and found identical to literature values.[2]

**2-Methyl-*N*-(prop-2-yn-1-yl)benzamide (8c).**

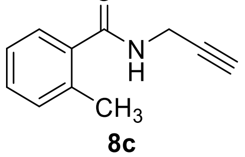

**8c**

Prepared from 2-methylbenzoyl chloride (773.0 mg, 5 mmol) and propargylamine (275.4 mg, 5 mmol), The crude product was purified by column chromatography (petroleum ether/ethyl acetate, 1:1) afford a white solid (735.6 mg, 85%). $^1$H NMR (400 MHz, $CDCl_3$) $\delta$ 7.34 - 7.11 (m, 4H), 5.85 (bs, 1H), 4.17 (dd, $J$ = 2.7, 5.4 Hz, 2H), 2.30

(s, 3H) 2.28 (t, $J$ = 2.7 Hz, 1H). These data were compared and found identical to literature values.[3]

**3-Methyl-*N*-(prop-2-yn-1-yl)benzamide (8d).**

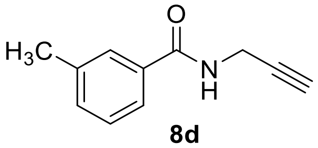

**8d**

Prepared from 3-methylbenzoyl chloride (773.0 mg, 5 mmol) and propargylamine (275.4 mg, 5 mmol), The crude product was purified by column chromatography (petroleum ether/ethyl acetate, 1:1) afford a white solid (761.6 mg, 88%). $^1$H NMR (400 MHz, $CDCl_3$) $\delta$ 7.43 - 7.20 (3m, 4H), 5.96 (s, 1H), 4.25 (dd, $J$ = 5.2, 2.5 Hz, 2H), 2.47 (s, 3H), 2.29 (t, $J$ = 2.5 Hz, 1H). These data were compared and found identical to literature values.[4]

**4-Methoxy-*N*-(prop-2-yn-1-yl)benzamide (8e).**

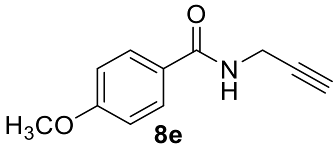

**8e**

Prepared from 4-methoxybenzoyl chloride (853.0 mg, 5 mmol) and propargylamine (275.4 mg, 5 mmol), The crude product was purified by column chromatography (petroleum ether/ethyl acetate, 1:1) afford a white solid (709.0 mg, 75%). $^1$H NMR (400 MHz, $CDCl_3$) $\delta$ 7.84 - 7.67 (m, 2H), 7.03 - 6.85 (m, 2H), 6.29 (bs, 1H), 4.24 (dd, $J$ = 5.2, 2.6 Hz, 2H), 3.84 (s, 3H), 2.27 (t, $J$ = 2.6 Hz, 1H). These data were compared and found identical to literature values.[2]

**3,4,5-Trimethoxy-*N*-(prop-2-yn-1-yl)benzamide (8f).**

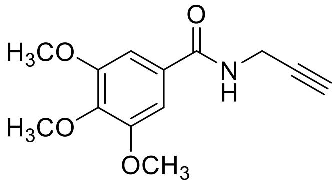

**8f**

Prepared from 3,4,5-trimethoxybenzoyl chloride (1.15 g, 5 mmol) and propargylamine (275.4 mg, 5 mmol), The crude product was purified by column chromatography (petroleum ether/ethyl acetate, 1:1) afford a yellow solid (978.1 mg, 72%). $^1$H NMR (400 MHz, $CDCl_3$) $\delta$ 6.93 (s, 2H), 6.16 (bs, 1H), 4.18 (dd, $J$ = 2.4, 5.1 Hz, 2H), 3.83 (s, 6H), 3.81 (s, 3H), 2.22 (t, $J$ = 2.4 Hz, 1H). These data were compared and found identical to literature values.[3]

**4-Nitro-*N*-(prop-2-yn-1-yl)benzamide (8g).**

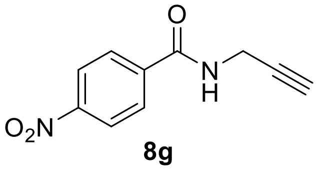

**8g**

Prepared from 4-nitrobenzoyl chloride (927.8 mg, 5 mmol) and propargylamine (275.4

mg, 5 mmol), The crude product was purified by column chromatography (petroleum ether/ethyl acetate, 1:1) afford a white solid (735.1 mg, 72%). $^1$H NMR (400 MHz, DMSO-$d_6$) $\delta$ 9.29 (t, $J$ = 5.5 Hz, 1H), 8.34 – 8.20 (m, 2H), 8.15 – 8.03 (m, 2H), 4.10 (dd, $J$ = 5.5, 2.6 Hz, 2H), 3.18 (s, 1H). These data were compared and found identical to literature values.[2]

**4-Cyano-*N*-(prop-2-yn-1-yl)benzamide (8h).**

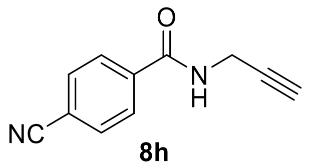


**8h**

Prepared from 4-cyanobenzoyl chloride (827.9 mg, 5 mmol) and propargylamine (275.4 mg, 5 mmol), The crude product was purified by column chromatography (petroleum ether/ethyl acetate, 1:1) afford a white solid (626.3 mg, 68%). $^1$H NMR (400 MHz, $CDCl_3$) $\delta$ 7.89 (m, 2H), 7.75 (m, 2H), 6.40 (bs, 1H), 4.27 (dd, $J$ = 5.2, 2.6 Hz, 2H), 2.31 (t, $J$ = 2.6 Hz, 1H). These data were compared and found identical to literature values.[2]

***N*-(Prop-2-yn-1-yl)-4-(trifluoromethyl)benzamide (8i).**

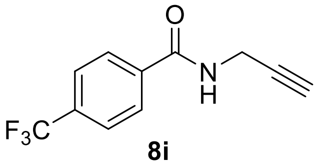


**8i**

Prepared from 4-(trifluoromethyl)benzoyl chloride (1.04 g, 5 mmol) and propargylamine (275.4 mg, 5 mmol), The crude product was purified by column chromatography (petroleum ether/ethyl acetate, 1:1) afford a white solid (1.01 g, 89%). $^1$H NMR (400 MHz, $CDCl_3$) $\delta$ 8.02 – 7.82 (m, 2H), 7.79 – 7.61 (m, 2H), 6.46 (bs, 1H), 4.27 (dd, $J$ = 5.2, 2.6 Hz, 2H), 2.30 (t, $J$ = 2.6 Hz, 1H). These data were compared and found identical to literature values.[2]

***N*-(Prop-2-yn-1-yl)-2-(trifluoromethyl)benzamide (8j).**

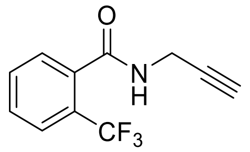


**8j**

Prepared from 2-(trifluoromethyl)benzoyl chloride (1.04 g, 5 mmol) and propargylamine (275.4 mg, 5 mmol), The crude product was purified by column chromatography (petroleum ether/ethyl acetate, 1:1) afford a white solid (1.05 g, 92%). $^1$H NMR (600 MHz, $CDCl_3$) $\delta$ 7.69 (d, $J$ = 6.3 Hz, 1H), 7.56 (dt, $J$ = 23.7, 6.9 Hz, 3H), 6.14 (s, 1H), 4.22 (s, 2H), 2.28 (s, 1H). $^{13}$C NMR (150 MHz, $CDCl_3$) $\delta$ 167.3, 135.0, 132.0, 130.1, 128.6, 127.3 (q, $J$ = 32.5, 32.1 Hz), 126.4 (q, $J$ = 4.3 Hz), 124.6 – 120.5 (m), 78.7, 72.1, 29.8. HRMS calcd for $C_{11}H_8F_3NO^+$ $[M+H]^+$ 228.0631, found 228.0628.

**4-Fluoro-*N*-(prop-2-yn-1-yl)benzamide (8k).**

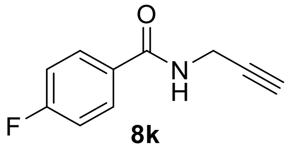

**8k**

Prepared from 4-fluorobenzoyl chloride (792.8 mg, 5 mmol) and propargylamine (275.4 mg, 5 mmol), The crude product was purified by column chromatography (petroleum ether/ethyl acetate, 1:1) afford a white solid (708.7 mg, 80%). $^1$H NMR (400 MHz, $CDCl_3$) $\delta$ 7.89 – 7.77 (m, 2H), 7.16 – 7.08 (m, 2H), 6.44 (bs, 1H), 4.26 (dd, $J$ = 5.3, 2.6 Hz, 2H), 2.30 (t, $J$ = 2.6 Hz, 1H). These data were compared and found identical to literature values.[2]

**4-Chloro-*N*-(prop-2-yn-1-yl)benzamide (8l).**

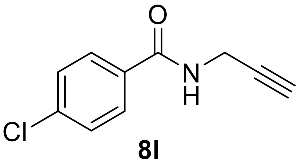

**8l**

Prepared from 4-chlorobenzoyl chloride (875.0 mg, 5 mmol) and propargylamine (275.4 mg, 5 mmol), The crude product was purified by column chromatography (petroleum ether/ethyl acetate, 1:1) afford a white solid (813.2 mg, 84%). $^1$H NMR (400 MHz, $CDCl_3$) $\delta$ 7.78-7.72 (m, 2 H), 7.46-7.40 (m, 2 H), 6.42 (br.s, 1 H), 4.26 (dd, $J$ = 5.2, 2.6 Hz, 2 H), 2.31 (t, $J$ = 2.6 Hz, 1 H). These data were compared and found identical to literature values.[5]

**3-Chloro-*N*-(prop-2-yn-1-yl)benzamide (8m).**

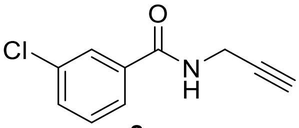

**8m**

Prepared from 3-chlorobenzoyl chloride (875.0 mg, 5 mmol) and propargylamine (275.4 mg, 5 mmol), The crude product was purified by column chromatography (petroleum ether/ethyl acetate, 1:1) afford a white solid (823.0 mg, 85%). $^1$H NMR (400 MHz, $CDCl_3$) $\delta$ 7.80 (t, $J$ = 1.7 Hz, 1 H), 7.68 (d, $J$ = 7.7 Hz, 1 H), 7.53-7.48 (m, 1 H), 7.40 (t, $J$ = 7.9 Hz, 1 H), 6.40 (br.s, 1 H), 4.27 (dd, $J$ = 5.2, 2.5 Hz, 2 H), 2.31 (t, $J$ = 2.5 Hz, 1 H). These data were compared and found identical to literature values.[5]

**4-Bromo-*N*-(prop-2-yn-1-yl)benzamide (8n).**

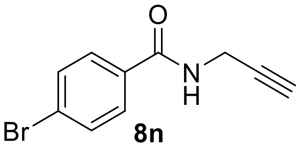

**8n**

Prepared from 4-bromobenzoyl chloride (1.10 g, 5 mmol) and propargylamine (275.4 mg, 5 mmol), The crude product was purified by column chromatography (petroleum ether/ethyl acetate, 1:1) afford a yellow solid (952.3 mg, 80%). $^1$H NMR (400 MHz, $CDCl_3$) $\delta$ 7.68 – 7.62 (m, 2H), 7.60 – 7.54 (m, 2H), 6.46 (bs, 1H), 4.23 (dd, $J$ = 5.3, 2.6

Hz, 2H), 2.28 (t, $J$ = 2.6 Hz, 1H). These data were compared and found identical to literature values.[2]

***N*-(Prop-2-yn-1-yl)-2-naphthamide (8o).**

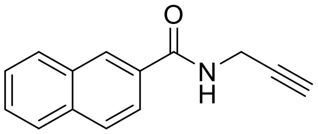

**8o**

Prepared from 2-naphthoyl chloride (953.1 mg, 5 mmol) and propargylamine (275.4 mg, 5 mmol), The crude product was purified by column chromatography (petroleum ether/ethyl acetate, 1:1) afford a white solid (763.8 mg, 73%). $^1$H NMR (400 MHz, $CDCl_3$) $\delta$ 8.36 – 8.14 (m, 1H), 8.06 – 7.71 (m, 4H), 7.64 – 7.39 (m, 2H), 6.57 (bs, 1H), 4.32 (dd, $J$ = 5.3, 2.6 Hz, 2H), 2.31 (t, $J$ = 2.6 Hz, 1H). These data were compared and found identical to literature values.[2]

***N*-(Prop-2-yn-1-yl)cinnamamide (8p).**

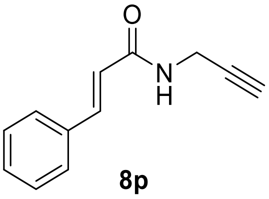

**8p**

Prepared from cinnamoyl chloride (833.0 mg, 5 mmol) and propargylamine (275.4 mg, 5 mmol), The crude product was purified by column chromatography (petroleum ether/ethyl acetate, 1:1) afford a yellow solid (648.3 mg, 70%). $^1$H NMR (400 MHz, $CDCl_3$) $\delta$ = 7.66 (d, $J$ = 15.6 Hz, 1H), 7.56 – 7.45 (m, 2H), 7.41 – 7.31 (m, 3H), 6.42 (d, $J$ = 15.6 Hz, 1H), 5.98 (bs, 1H), 4.20 (dd, $J$ = 5.3, 2.6 Hz, 2H), 2.26 (t, $J$ = 2.6 Hz, 1H). These data were compared and found identical to literature values.[2]

***N*-(Prop-2-yn-1-yl)thiophene-2-carboxamide (8q).**

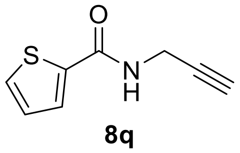

**8q**

Prepared from thiophene-2-carbonyl chloride (732.9 mg, 5 mmol) and propargylamine (275.4 mg, 5 mmol), The crude product was purified by column chromatography (petroleum ether/ethyl acetate, 1:1) afford a yellow solid (495.6 mg, 60%). $^1$H NMR (400 MHz, $CDCl_3$) $\delta$ 7.44 (dd, $J$ = 1.8, 0.8 Hz, 1H), 7.13 (dd, $J$ = 3.5, 0.8 Hz, 1H), 6.56 (bs, 1H), 6.49 (dd, $J$ = 3.5, 1.8 Hz, 1H), 4.22 (dd, $J$ = 5.4, 2.6 Hz, 2H), 2.27 (t, $J$ = 2.6 Hz, 1H). These data were compared and found identical to literature values.[2]

***N*$^1$,*N*$^3$-di(Prop-2-yn-1-yl)isophthalamide (8r).**

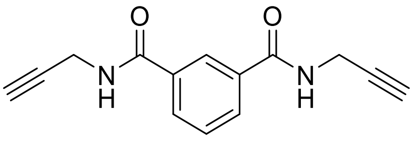

**8r**

Prepared from isophthaloyl dichloride (1.02 g, 5 mmol) and propargylamine (550.8 mg,

10 mmol), The crude product was purified by column chromatography (petroleum ether/ethyl acetate, 1:1) afford a white solid (540.6 mg, 45%). $^1$H NMR (400 MHz, DMSO-$d_6$)$\delta$ 9.06 ( t, $J$ = 5.51 Hz, 2H), 8.32 ( t, $J$ = 1.61 Hz, 1H), 8.00-7.98 (m, 2H), 7.58 ( t, $J$ = 7.75 Hz, 1H), 4.09-4.07 ( m, 4H), 3.11 (t, $J$ = 2.49 Hz, 2H). These data were compared and found identical to literature values.[6]

**2-Phenyl-*N*-(prop-2-yn-1-yl)butanamide (8s).**

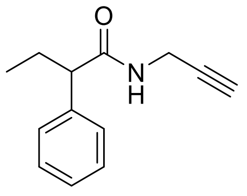

**8s**

Prepared from 2-phenylbutanoyl chloride (913.2 mg, 5 mmol) and propargylamine (275.4 mg, 5 mmol), The crude product was purified by column chromatography (petroleum ether/ethyl acetate, 1:1) afford a white solid (553.5 mg, 55%). $^1$H NMR (400 MHz, $CDCl_3$) $\delta$ 7.38-7.26 (m, 5H), 5.67 (s, 1H), 4.06 (ddd, $J$ = 2.7, 5.4 and 17.7 Hz, 1H), 3.94 (ddd, $J$ = 2.7, 5.1 and 17.7 Hz, 1H), 3.26 (t, $J$ = 7.5 Hz, 1H), 2.29-2.14 (m, 2H), 1.89-1.74 (m, 1H), 0.89 (t, $J$ = 7.5 Hz, 3H). These data were compared and found identical to literature values.[7]

***N*-(Prop-2-yn-1-yl)acetamide (8t).**

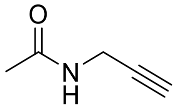

**8t**

Prepared from acetyl chloride (392.5 mg, 5 mmol) and propargylamine (275.4 mg, 5 mmol), The crude product was purified by column chromatography (petroleum ether/ethyl acetate, 5:1) afford a yellow solid (407.9 mg, 84%). $^1$H NMR (400 MHz, $CDCl_3$) $\delta$ 5.88 (s, 1H), 4.03 (dd, $J$ = 5.2, 2.5 Hz, 2H), 2.22 (t, $J$ = 2.5 Hz, 1H), 2.00 (s, 3H). These data were compared and found identical to literature values.[6]

***N*-(Prop-2-yn-1-yl)pivalamide (8u).**

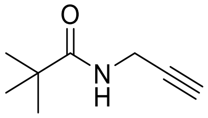

**8u**

Prepared from pivaloyl chloride (602.9 mg, 5 mmol) and propargylamine (275.4 mg, 5 mmol), The crude product was purified by column chromatography (petroleum ether/ethyl acetate, 5:1) afford a white solid (570.7 mg, 82%). $^1$H NMR (400 MHz, $CDCl_3$) $\delta$ 5.86 (br s, 1H), 4.02 (dd, $J$ = 5.0, 2.6, 2H), 2.22 (t, $J$ = 2.6, 1H), 1.19 (s, 9H). These data were compared and found identical to literature values.[8]

***N*-(Prop-2-yn-1-yl)cyclohexanecarboxamide (8v).**

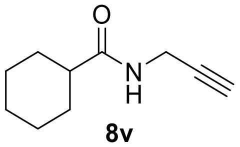

**8v**

Prepared from cyclohexanecarbonyl chloride (733.1 mg, 5 mmol) and propargylamine (275.4 mg, 5 mmol), The crude product was purified by column chromatography (petroleum ether/ethyl acetate, 5:1) afford a white solid (479.2 mg, 58%). $^1$H NMR (400 MHz, $CDCl_3$) $\delta$ 5.67 (s, 1H), 4.12 - 3.96 (m, 2H), 2.22 (t, $J$ = 2.2 Hz, 1H), 2.09 (tt, $J$ = 11.7, 3.3 Hz, 1H), 1.97 -1.68 (m, 5H), 1.52 - 1.35 (m, 2H), 1.35 - 1.13 (m, 3H). These data were compared and found identical to literature values.[9]

***N*-(Prop-2-yn-1-yl)heptanamide (8w).**

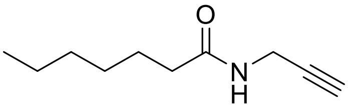

**8w**

Prepared from heptanoyl chloride (743.2 mg, 5 mmol) and propargylamine (275.4 mg, 5 mmol), The crude product was purified by column chromatography (petroleum ether/ethyl acetate, 5:1) afford a white solid (409.8 mg, 49%). $^1$H NMR (400 MHz, $CDCl_3$) $\delta$ 5.71 (br, s, 1H), 4.07 (q, $J$ = 5.1 Hz, 2H), 2.24 (t, $J$ = 5.1 Hz, 1H), 2.21 (t, $J$ = 7.4 Hz, 2H), 1.61-1.68 (m, 2H), 1.36-1.26 (m, 6H), 0.89 (t, $J$ = 7.0 Hz, 3H). These data were compared and found identical to literature values.[9]

***N*-(2-Methylbut-3-yn-2-yl)cyclohexanecarboxamide (8x).**

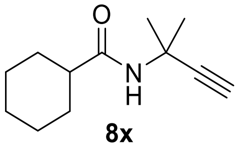

**8x**

Prepared from cyclohexanecarbonyl chloride (733.1 mg, 5 mmol) and 2-methylbut-3-yn-2-amine (415.7 mg, 5 mmol), The crude product was purified by column chromatography (petroleum ether/ethyl acetate, 5:1) afford a white solid (840.8 mg, 87%). $^1$H NMR (400 MHz, $CDCl_3$) $\delta$ 5.64 (bs, 1H), 2.28 (s, 1H), 2.01-1.94 (m, 1H), 1.82-1.72 (m, 4H), 1.62-1.59 (m, 1H), 1.59 (s, 6H), 1.43-1.33 (m, 2H), 1.26-1.17 (m, 3H). These data were compared and found identical to literature values.[9]

***N*-(2-Methylbut-3-yn-2-yl)-4-nitrobenzamide (8y).**

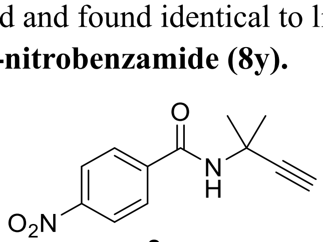

**8y**

Prepared from 4-nitrobenzoyl chloride (927.8 mg, 5 mmol) and 2-methylbut-3-yn-2-amine (415.7 mg, 5 mmol), The crude product was purified by column chromatography (petroleum ether/ethyl acetate, 1:1) afford a white solid (963.8 mg, 83%). $^1$H NMR (400 MHz, $CDCl_3$) $\delta$ 8.22 (d, $J$ = 8.2 Hz, 2H), 7.90 (d, $J$ = 8.2 Hz, 2H), 6.48 (bs, 1H), 2.40 (s, 1H), 1.75 (s, 6H). These data were compared and found identical to literature values.[9]

***N*-(2-Methylbut-3-yn-2-yl)benzamide (8z).**

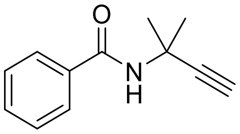

**8z**

Prepared from benzoyl chloride (695.0 mg, 5 mmol) and 2-methylbut-3-yn-2-amine (415.7 mg, 5 mmol), The crude product was purified by column chromatography (petroleum ether/ethyl acetate, 1:1) afford a white solid (889.4 mg, 95%). $^1$H NMR (400 MHz, $CDCl_3$) $\delta$ 7.79-7.74 (m, 2H), 7.53-7.48 (m, 1H), 7.46-7.40 (m, 2H), 6.21 (br. s., 1H), 2.40 (s, 1H), 1.78 (s, 6H). These data were compared and found identical to literature values.[2]

**2-Methyl-*N*-(2-methylbut-3-yn-2-yl)benzamide (8aa).**

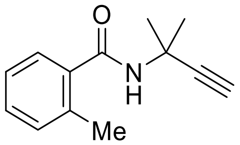


**8aa**

Prepared from 2-methylbenzoyl chloride (773.0 mg, 5 mmol) and 2-methylbut-3-yn-2-amine (415.7 mg, 5 mmol), The crude product was purified by column chromatography (petroleum ether/ethyl acetate, 1:1) afford a white solid (935.9 mg, 93%). $^1$H NMR (400 MHz, $CDCl_3$) $\delta$ 7.46-7.27 (m, 2H), 7.25-7.05 (m, 2H), 5.89 (bs, 1H), 2.44 (s, 3H), 2.38 (s, 1H), 1.75 (s, 6H). These data were compared and found identical to literature values.[9]

***N*-(2-Methyl-4-phenylbut-3-yn-2-yl)benzamide (8ab).**

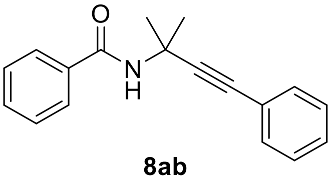

**8ab**

To a solution of 2-methyl-4-phenylbut-3-yn-2-amine (318.5 mg, 2.0 mmol, 1.0 eq.) in DCM (20 ml), $Et_3N$ (0.1 ml, 2.0 mmol, 1.0 eq.) and DMAP (4.9 mg, 0.04 mmol, 0.02 eq.) were added. The resulting mixture was cooled to 0 °C, and benzoyl chloride (281.1 mg, 2.0 mmol, 1.0 eq.) was added. The mixture was stirred for 30 min at 0 °C and 3 h at room temperature. $H_2O$ (5 ml) was added, and the aqueous layer extracted with another 3 × 5 ml of DCM. The combined organic extracts were washed with saturated $NaHCO_3$, $H_2O$ and brine, dried over $Na_2SO_4$ and concentrated in vacuo. The crude product was purified by column chromatography on silica (petroleum ether/ethyl acetate, 4:1) to afford **8ab** as a white solid (237.0 mg, 45%). $^1$H NMR (400 MHz, $CDCl_3$): $\delta$ 7.79 – 7.77 (m, 2H), 7.51 – 7.41 (m, 5H), 7.31 – 7.28 (m, 3H), 6.32 (br s, 1H), 1.87 (s, 6H). These data were compared and found identical to literature values.[8]

**4-Methyl-*N*-(2-methyl-4-phenylbut-3-yn-2-yl)benzamide (8ac).**

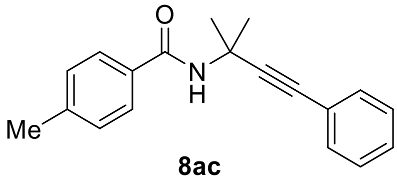

**8ac**

To a solution of 2-methyl-4-phenylbut-3-yn-2-amine (318.5 mg, 2.0 mmol, 1.0 eq.) in DCM (20 ml), $Et_3N$ (0.1 ml, 2.0 mmol, 1.0 eq.) and DMAP (4.9 mg, 0.04 mmol, 0.02 eq.) were added. The resulting mixture was cooled to 0 °C, and 4-methylbenzoyl chloride (309.2 mg, 2.0 mmol, 1.0 eq.) was added. The mixture was stirred for 30 min at 0 °C and 3 h at room temperature. $H_2O$ (5 ml) was added, and the aqueous layer extracted with another 3 × 5 ml of DCM. The combined organic extracts were washed with saturated $NaHCO_3$, $H_2O$ and brine, dried over $Na_2SO_4$ and concentrated in vacuo. The crude product was purified by column chromatography on silica (petroleum ether/ethyl acetate, 4:1) to afford **8ac** as a yellow solid (237.0 mg, 45%). $^1$H NMR (400 M, $CDCl_3$): $\delta$ 7.74 (d, $J$ = 7.5 Hz, 2H), 7.52 (dd, $J$ = 6.7, 3.3 Hz, 2H), 7.36 (s, 3H), 7.29 (d, $J$ = 7.8 Hz, 2H), 6.37 (s, 1H), 2.46 (s, 3H), 1.93 (s, 6H). $^{13}$C NMR (100 MHz, $CDCl_3$) $\delta$ 166.5, 141.8, 132.3, 131.8, 129.2, 128.6, 128.2, 126.9, 122.8, 92.9, 81.4, 49.1, 29.0, 21.5. HRMS calcd for $C_{19}H_{19}NO^+$ $[M+H]^+$ 278.1539, found 278.1537.

***N*-(2-Methyl-4-phenylbut-3-yn-2-yl)-4-nitrobenzamide (8ad).**

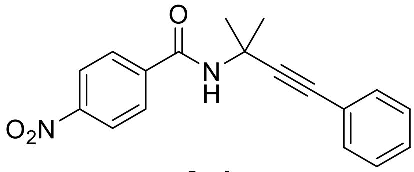

**8ad**

To a solution of 2-methyl-4-phenylbut-3-yn-2-amine (318.5 mg, 2.0 mmol, 1.0 eq.) in DCM (20 ml), $Et_3N$ (0.1 ml, 2.0 mmol, 1.0 eq.) and DMAP (4.9 mg, 0.04 mmol, 0.02 eq.) were added. The resulting mixture was cooled to 0 °C, and 4-nitrobenzoyl chloride (317.1 mg, 2.0 mmol, 1.0 eq.) was added. The mixture was stirred for 30 min at 0 °C and 3 h at room temperature. $H_2O$ (5 ml) was added, and the aqueous layer extracted with another 3 × 5 ml of DCM. The combined organic extracts were washed with saturated $NaHCO_3$, $H_2O$ and brine, dried over $Na_2SO_4$ and concentrated in vacuo. The crude product was purified by column chromatography on silica (petroleum ether/ethyl acetate, 4:1) to afford **8ad** as a yellow solid (237.0 mg, 45%). $^1$H NMR (400 M, $CDCl_3$): $\delta$ 8.31 (d, $J$ = 8.3 Hz, 2H), 8.01 – 7.95 (m, 2H), 7.48 (d, $J$ = 6.3 Hz, 2H), 7.34 (d, $J$ = 6.1 Hz, 3H), 6.45 (s, 1H), 1.92 (s, 6H). $^{13}$C NMR (100 MHz, $CDCl_3$) $\delta$ 164.5, 140.7, 131.8, 128.5, 128.3, 128.2, 123.8, 122.5, 92.0, 81.9, 49.6, 28.9. HRMS calcd for $C_{18}H_{16}N_2O_3^+$ $[M+H]^+$ 309.1234, found 309.1230.

***N*-(2-Methyl-4-phenylbut-3-yn-2-yl)thiophene-2-carboxamide (8ae).**

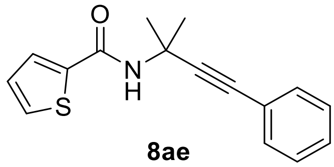

**8ae**

To a solution of 2-methyl-4-phenylbut-3-yn-2-amine (318.5 mg, 2.0 mmol, 1.0 eq.) in DCM (20 ml), $Et_3N$ (0.1 ml, 2.0 mmol, 1.0 eq.) and DMAP (4.9 mg, 0.04 mmol, 0.02 eq.) were added. The resulting mixture was cooled to 0 °C, and thiophene-2-carbonyl chloride (293.2 mg, 2.0 mmol, 1.0 eq.) was added. The mixture was stirred for 30 min at 0 °C and 3 h at room temperature. $H_2O$ (5 ml) was added, and the aqueous layer extracted with another 3 × 5 ml of DCM. The combined organic extracts were washed with saturated $NaHCO_3$, $H_2O$ and brine, dried over $Na_2SO_4$ and concentrated in vacuo. The crude product was purified by column chromatography on silica (petroleum ether/ethyl acetate, 4:1) to afford **8ae** as a yellow solid (237.0 mg, 45%). $^1$H NMR (600 M, $CDCl_3$): $\delta$ 7.51 – 7.47 (m, 1H), 7.44 (dd, $J$ = 6.8, 2.5 Hz, 3H), 7.28 (dd, $J$ = 6.7, 3.5 Hz, 3H), 7.03 (dd, $J$ = 4.9, 3.8 Hz, 1H), 6.26 (s, 1H), 1.84 (s, 6H). $^{13}$C NMR (150 MHz, $CDCl_3$) $\delta$ 160.9, 139.7, 131.8, 129.9, 128.3, 128.2, 128.0, 127.5, 122.8, 92.6, 81.6, 49.3, 29.1. HRMS calcd for $C_{16}H_{15}NOS^+$ $[M+H]^+$ 270.0947, found 270.0943.

General procedure for the synthesis of substrates (**8af-8aj**) (Procedure B):

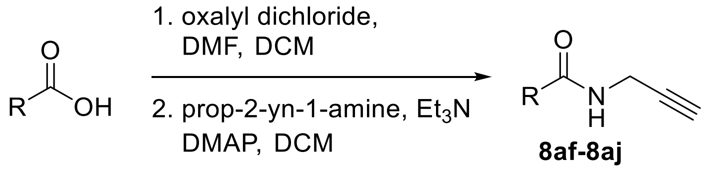


**5-(2-Chloro-4-(trifluoromethyl)phenoxy)-2-nitro-*N*-(prop-2-yn-1-yl)benzamide (8af).**

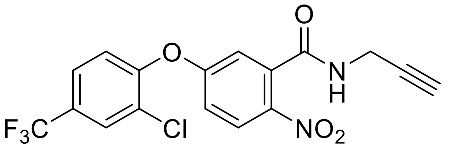


**8af**

A 100 ml round-bottom flask was charged with Acifluorfen (1.81 g, 5 mmol, 1.0 eq.), DCM (20 ml) and catalytic amount of DMF. The reaction mixture was cooled to 0 °C and stirred for 10 minutes. Then oxalyl dichloride (3.0 ml, 6 mmol, 2.0 M, 1.2 equiv.) was added dropwise to the reaction mixture and stirred at room temperature for 4 h. The resulting mixture was concentrated under reduced pressure to afford acid chloride which was used directly without further purification for the next step. A 100 ml round-bottom flask was charged with propargylic amine (330.5 mg, 6 mmol, 1.2 eq.), $Et_3N$ (0.7 ml, 5 mmol, 1.0 eq.), DMAP (12.2 mg, 0.1 mmol, 0.02 eq.) and DCM (20 ml). The reaction mixture was then cooled to 0 °C and acid chloride (1.0 equiv.) was added dropwise at 0 °C and the reaction mixture was stirred at room temperature for 12 h. Then water (20 ml) was added, the organic layer was separated and the aqueous layer was extracted with DCM (3 x 30 ml). The combined organic layer was washed with saturated aqueous $NaHCO_3$ (30 ml) solution followed by water (30 ml). After that, the organic layer was dried over $Na_2SO_4$ and concentrated under reduced pressure. The crude mass was purified by silica gel column chromatography (petroleum ether/ethyl acetate, 10:4) to give **8af** as white solid (1.30 g, 45%). $^1$H NMR (600 M, $CDCl_3$): $\delta$ 8.16 – 8.11 (m, 1H), 7.84 – 7.79 (m, 1H), 7.63 (d, $J$ = 8.4 Hz, 1H), 7.27 (d, $J$ = 8.2 Hz, 1H),

7.02 (dd, $J = 6.2, 2.6$ Hz, 2H), 6.20 – 6.14 (m, 1H), 4.23 (dd, $J = 5.2, 2.5$ Hz, 2H), 2.30 (t, $J = 2.4$ Hz, 1H). $^{13}$C NMR (150 MHz, $CDCl_3$) $\delta$ 165.4, 160.6, 152.6, 140.9, 134.9, 129.4 (q, $J = 33.7$ Hz), 128.8 (q, $J = 3.7$ Hz), 127.5 (d, $J = 13.8$ Hz), 125.9 (q, $J = 3.5$ Hz), 123.8, 122.8, 122.0, 117.7, 116.7, 78.4, 72.5, 30.1. HRMS calcd for $C_{17}H_{11}ClF_3N_2O_4^+$ $[M+H]^+$ 399.0354, found 399.0349.

**_N_-(Prop-2-yn-1-yl)-1,3-dihydroisobenzofuran-5-carboxamide (8ag).**

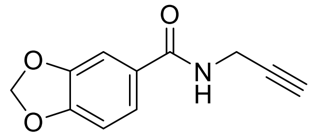

**8ag**

A 100 ml round-bottom flask was charged with Piperonylic acid (830.7 mg, 5 mmol, 1.0 eq.), DCM (20 ml) and catalytic amount of DMF. The reaction mixture was cooled to 0 °C and stirred for 10 minutes. Then oxalyl dichloride (3.0 ml, 6 mmol, 2.0 M, 1.2 equiv.) was added dropwise to the reaction mixture and stirred at room temperature for 4 h. The resulting mixture was concentrated under reduced pressure to afford acid chloride which was used directly without further purification for the next step. A 100 ml round-bottom flask was charged with propargylic amine (330.5 mg, 6 mmol, 1.2 eq.), $Et_3N$ (0.7 ml, 5 mmol, 1.0 eq.), DMAP (12.2 mg, 0.1 mmol, 0.02 eq.) and DCM (20 ml). The reaction mixture was then cooled to 0 °C and acid chloride (1.0 equiv.) was added dropwise at 0 °C and the reaction mixture was stirred at room temperature for 12 h. Then water (20 ml) was added, the organic layer was separated and the aqueous layer was extracted with DCM (3 x 30 ml). The combined organic layer was washed with saturated aqueous $NaHCO_3$ (30 ml) solution followed by water (30 ml). After that, the organic layer was dried over $Na_2SO_4$ and concentrated under reduced pressure. The crude mass was purified by silica gel column chromatography (petroleum ether/ethyl acetate, 10:4) to give **8ag** as white solid (772.1 mg, 76%). $^1$H NMR (600 MHz,$CDCl_3$): $\delta$ 7.32 (dd, $J = 8.1, 1.7$ Hz, 1H), 7.29 (d, $J = 1.6$ Hz, 1H), 6.83 (d, $J = 8.1$ Hz, 1H), 6.27 (s, 1H), 6.03 (s, 2H), 4.27 – 4.18 (m, 2H), 2.28 (t, $J = 2.5$ Hz, 1H). $^{13}$C NMR (150 MHz, $CDCl_3$): $\delta$ 166.4, 150.6, 148.0, 127.9, 121.7, 108.0, 107.7, 101.8, 79.6, 71.9, 29.8.166.4, 150.6, 148.0, 127.9, 121.7, 108.0, 107.7, 101.8, 79.6, 71.9, 29.8. HRMS calcd for $C_{11}H_9NO_3^+$ $[M+H]^+$ 204.0655, found 204.0653.

**3,6-Dichloro-2-methoxy-_N_-(prop-2-yn-1-yl)benzamide (8ah).**

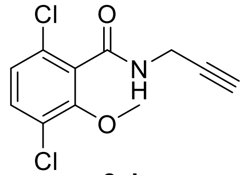

**8ah**

A 100 ml round-bottom flask was charged with 3,6-dichloro-2-methoxybenzoic acid (1.11 g, 5 mmol, 1.0 eq.), DCM (20 ml) and catalytic amount of DMF. The reaction mixture was cooled to 0 °C and stirred for 10 minutes. Then oxalyl dichloride (3.0 ml, 6 mmol, 2.0 M, 1.2 equiv.) was added dropwise to the reaction mixture and stirred at room temperature for 4 h. The resulting mixture was concentrated under reduced pressure to afford acid chloride which was used directly without further purification for

the next step. A 100 ml round-bottom flask was charged with propargylic amine (330.5 mg, 6 mmol, 1.2 eq.), $Et_3N$ (0.7 ml, 5 mmol, 1.0 eq.), DMAP (12.2 mg, 0.1 mmol, 0.02 eq.) and DCM (20 ml). The reaction mixture was then cooled to 0 °C and acid chloride (1.0 equiv.) was added dropwise at 0 °C and the reaction mixture was stirred at room temperature for 12 h. Then water (20 ml) was added, the organic layer was separated and the aqueous layer was extracted with DCM (3 x 30 ml). The combined organic layer was washed with saturated aqueous $NaHCO_3$ (30 ml) solution followed by water (30 ml). After that, the organic layer was dried over $Na_2SO_4$ and concentrated under reduced pressure. The crude mass was purified by silica gel column chromatography (petroleum ether/ethyl acetate, 10:4) to give **8ah** as yellow solid (864.6 mg, 67%). $^1$H NMR (600 MHz, $CDCl_3$): $\delta$ 7.34 (d, $J$ = 8.6 Hz, 1H), 7.12 (d, $J$ = 8.6 Hz, 1H), 6.09 (s, 1H), 4.27 (dd, $J$ = 5.3, 2.6 Hz, 2H), 3.91 (s, 3H), 2.29 (t, $J$ = 2.6 Hz, 1H). $^{13}$C NMR (150 MHz, $CDCl_3$): δ 163.7, 153.9, 132.1, 131.7, 130.2, 126.9, 126.1, 78.6, 72.2, 62.7, 29.7. HRMS calcd for $C_{11}H_9Cl_2NO_2^+$ $[M+H]^+$ 258.0083, found 258.0082.

**2-(3-Cyano-4-isobutoxyphenyl)-4-methyl-*N*-(prop-2-yn-1-yl)thiazole-5-carboxamide (8ai).**

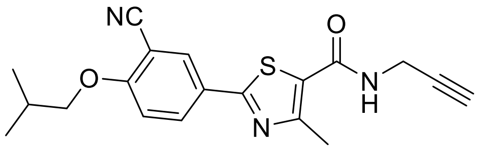


**8ai**

A 100 ml round-bottom flask was charged with Febuxostat (1.58 g, 5 mmol, 1.0 eq.), DCM (20 ml) and catalytic amount of DMF. The reaction mixture was cooled to 0 °C and stirred for 10 minutes. Then oxalyl dichloride (3.0 ml, 6 mmol, 2.0 M, 1.2 equiv.) was added dropwise to the reaction mixture and stirred at room temperature for 4 h. The resulting mixture was concentrated under reduced pressure to afford acid chloride which was used directly without further purification for the next step. A 100 ml round-bottom flask was charged with propargylic amine (330.5 mg, 6 mmol, 1.2 eq.), $Et_3N$ (0.7 ml, 5 mmol, 1.0 eq.), DMAP (12.2 mg, 0.1 mmol, 0.02 eq.) and DCM (20 ml). The reaction mixture was then cooled to 0 °C and acid chloride (1.0 equiv.) was added dropwise at 0 °C and the reaction mixture was stirred at room temperature for 12 h. Then water (20 ml) was added, the organic layer was separated and the aqueous layer was extracted with DCM (3 x 30 ml). The combined organic layer was washed with saturated aqueous $NaHCO_3$ (30 ml) solution followed by water (30 ml). After that, the organic layer was dried over $Na_2SO_4$ and concentrated under reduced pressure. The crude mass was purified by silica gel column chromatography (petroleum ether/ethyl acetate, 10:4) to give **8ai** as white solid (1.41 g, 80%). $^1$H NMR (400 MHz, $CDCl_3$): $\delta$ 8.18 (d, $J$ = 37.5 Hz, 1H), 8.06 (d, $J$ = 9.1 Hz, 1H), 7.03 (dd, $J$ = 17.0, 8.6 Hz, 1H), 6.01 (s, 1H), 4.24 (d, $J$ = 4.7 Hz, 1H), 3.90 (d, $J$ = 4.8 Hz, 2H), 2.85 (s, 1H), 2.76 (d, $J$ = 10.3 Hz, 2H), 2.32 (s, 1H), 2.21 (dq, $J$ = 13.7, 6.8 Hz, 1H), 1.09 (d, $J$ = 5.7 Hz, 6H). $^{13}$C NMR (100 MHz, $CDCl_3$): δ 165.0, 162.4, 161.3, 156.9, 132.6, 132.0, 125.8, 124.9, 115.5, 112.7, 102.9, 79.0, 75.7, 72.3, 29.9, 28.2, 19.1, 17.5. These data were compared and found identical to literature values.[10]

**4-(*N*,*N*-Dipropylsulfamoyl)-*N*-(prop-2-yn-1-yl)benzamide (8aj).**

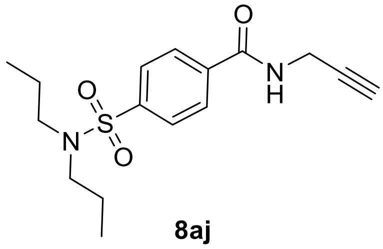

**8aj**

A 100 ml round-bottom flask was charged with Probenecid (1.43 g, 5 mmol, 1.0 eq.), DCM (20 ml) and catalytic amount of DMF. The reaction mixture was cooled to 0 °C and stirred for 10 minutes. Then oxalyl dichloride (3.0 ml, 6 mmol, 2.0 M, 1.2 equiv.) was added dropwise to the reaction mixture and stirred at room temperature for 4 h. The resulting mixture was concentrated under reduced pressure to afford acid chloride which was used directly without further purification for the next step. A 100 ml round-bottom flask was charged with propargylic amine (330.5 mg, 6 mmol, 1.2 eq.), $Et_3N$ (0.7 ml, 5 mmol, 1.0 eq.), DMAP (12.2 mg, 0.1 mmol, 0.02 eq.) and DCM (20 ml). The reaction mixture was then cooled to 0 °C and acid chloride (1.0 equiv.) was added dropwise at 0 °C and the reaction mixture was stirred at room temperature for 12 h. Then water (20 ml) was added, the organic layer was separated and the aqueous layer was extracted with DCM (3 x 30 ml). The combined organic layer was washed with saturated aqueous $NaHCO_3$ (30 ml) solution followed by water (30 ml). After that, the organic layer was dried over $Na_2SO_4$ and concentrated under reduced pressure. The crude mass was purified by silica gel column chromatography (petroleum ether/ethyl acetate, 10:4) to give **8aj** as white solid (1.18 g, 73%). $^1$H NMR (600 MHz, $CDCl_3$) $\delta$ 7.92 (d, $J$ = 8.3 Hz, 2H), 7.80 (d, $J$ = 8.2 Hz, 2H), 7.09 (t, $J$ = 4.8 Hz, 1H), 4.24 (dd, $J$ = 5.2, 2.4 Hz, 2H), 3.13 – 3.04 (m, 4H), 2.28 (t, $J$ = 2.4 Hz, 1H), 1.53 (h, $J$ = 7.4 Hz, 4H), 0.86 (t, $J$ = 7.4 Hz, 6H). $^{13}$C NMR (150 MHz, $CDCl_3$): δ 166.0, 142.9, 137.3, 128.0, 127.2, 79.2, 71.9, 49.9, 29.8, 21.9, 11.1. HRMS calcd for $C_{16}H_{22}N_2O_3S^+$ $[M+H]^+$ 323.1424, found 323.1421.

**Supplementary Method 8: Optimization of the Reaction Conditions.**

**Supplementary Table S1.** Optimization of reaction conditions[a]

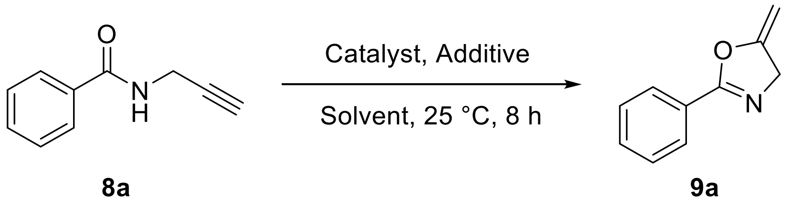


| Entry | Catalyst (mol%) | Solvent | Additive | Yield (%) |
|---|---|---|---|---|
| 1 | **4a** (10) | DCM | - | - |
| 2 | **4a** (10) | DCM | $CH_3COOH$ | 90 |
| 3 | **4a** (10) | DCM | benzoic acid | 82 |
| 4 | - | DCM | $CH_3COOH$ | - |
| 5 | - | DCM | benzoic acid | - |
| 6 | **4a** (10) | THF | $CH_3COOH$ | 78 |
| 7 | **4a** (10) | MeOH | $CH_3COOH$ | 34 |
| 8 | **4a** (10) | Toluene | $CH_3COOH$ | 83 |
| 9 | **4a** (10) | DMF | $CH_3COOH$ | 15 |
| 10 | **4a** (10) | 1,4-dioxane | $CH_3COOH$ | 69 |
| 11 | **4a** (10) | Acetone | $CH_3COOH$ | 75 |
| 12 | **4a** (10) | MeCN | $CH_3COOH$ | 50 |
| 13 | **4a** (10) | CPME | $CH_3COOH$ | 85 |
| 14 | **4b** (10) | DCM | $CH_3COOH$ | 95 |
| 15 | **4c** (10) | DCM | $CH_3COOH$ | 98 |
| 16 | **4d** (10) | DCM | $CH_3COOH$ | 96 |
| 17 | IPr-Ag-Cl (10) | DCM | $CH_3COOH$ | 16 |
| 18 | IMes-Ag-Cl (10) | DCM | $CH_3COOH$ | 7 |
| 19 | **4a** (5) | DCM | $CH_3COOH$ | 90 |
| 20 | **4b** (5) | DCM | $CH_3COOH$ | 93 |
| 21 | **4c** (5) | DCM | $CH_3COOH$ | 96 |
| 22 | **4d** (5) | DCM | $CH_3COOH$ | 91 |
| 23 | **4a** (1) | DCM | $CH_3COOH$ | 88 |
| 24 | **4b** (1) | DCM | $CH_3COOH$ | 85 |
| 25 | **4c** (1) | DCM | $CH_3COOH$ | >95 |
| 26 | **4d** (1) | DCM | $CH_3COOH$ | 84 |

[a]Reaction conditions: substrate **8a** (0.20 mmol, 1.0 equiv), Catalyst, Additive (0.20 mmol, 1.0 equiv), Solvent (0.2 mL, 1.0 M), 25 °C, 8 h. Yields were determined by $^1$H NMR analysis using dibromomethane as an internal standard.

**Supplementary Method 9: General Procedure for the Cyclisation of Propargylic Amides.**

An oven-dried vial equipped with a stir bar was charged with propargylic amides (0.2 mmol, 1.0 eq.), Ag catalyst **4c** (1.5 mg, 1.0 mol%). The reaction mixture was placed under a positive pressure of argon and subjected to three evacuation/backfilling cycles under high vacuum.Then AcOH (12.0 mg, 0.2 mmol, 1.0 eq.) and DCM (0.2 mL, 1.0 M) was added and the reaction mixture was stirred at room temperature for 8 h. The volatiles were removed in vacuo and the products were purified by column chromatography on silica gel.

**5-Methylene-2-phenyl-4,5-dihydrooxazole 9a.**

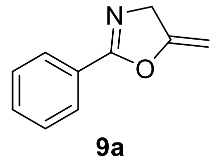


**9a**

After purification (Silica Gel, n-hexane/ethyl acetate 10:1) **9a** was isolated as a colorless oil in 98% yield (31.2 mg). $^{1}$H NMR (600 MHz, $CDCl_3$) $\delta$ 7.96 (d, $J$ = 7.8 Hz, 2H), 7.48 (t, $J$ = 7.4 Hz, 1H), 7.41 (t, $J$ = 7.7 Hz, 2H), 4.80 (q, $J$ = 3.0 Hz, 1H), 4.62 (t, $J$ = 2.8 Hz, 2H), 4.34 (q, $J$ = 2.7 Hz, 1H). $^{13}$C NMR (150 MHz, $CDCl_3$) $\delta$ 163.7, 158.8, 131.8, 128.5, 128.0, 127.8, 83.7, 57.7 . This compound showed identical spectroscopic properties to those reported previously.[6]

**5-Methylene-2-(*p*-tolyl)-4,5-dihydrooxazole 9b.**

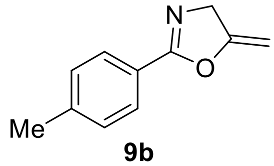


**9b**

After purification (Silica Gel, n-hexane/ethyl acetate 10:1) **9b** was isolated as a yellow solid in 97% yield (33.6 mg). $^{1}$H NMR (600 MHz, $CDCl_3$) $\delta$ 7.85 (d, $J$ = 8.1 Hz, 2H), 7.22 (d, $J$ = 8.1 Hz, 2H), 4.80 – 4.77 (m, 1H), 4.63 – 4.59 (m, 2H), 4.35 – 4.31 (m, 1H), 2.38 (s, 3H). $^{13}$C NMR (150 MHz, $CDCl_3$) $\delta$ 163.8, 158.9, 142.2, 129.2, 128.0, 124.0, 83.5, 57.7, 21.6 .This compound showed identical spectroscopic properties to those reported previously.[11]

**5-Methylene-2-(*o*-tolyl)-4,5-dihydrooxazole 9c.**

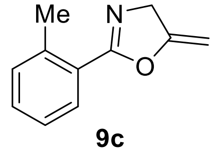


**9c**

After purification (Silica Gel, n-hexane/ethyl acetate 10:1) **9c** was isolated as a yellow oil in 92% yield (31.9 mg). $^{1}$H NMR (600 MHz, $CDCl_3$) $\delta$ 7.84 (d, $J$ = 7.7 Hz, 1H), 7.32 (t, $J$ = 7.5 Hz, 1H), 7.25 – 7.21 (m, 2H), 4.76 (d, $J$ = 2.8 Hz, 1H), 4.67 – 4.63 (m, 2H), 4.31 (d, $J$ = 2.5 Hz, 1H), 2.60 (s, 3H). $^{13}$C NMR (150 MHz, $CDCl_3$) $\delta$ 163.8, 158.4, 139.2, 131.4, 131.0, 129.8, 126.0, 125.7, 83.2, 58.3, 21.9. HRMS calcd for $C_{11}H_{11}NO^{+}$ $[M+H]^{+}$ 174.0913, found 174.0911.

**5-Methylene-2-(*m*-tolyl)-4,5-dihydrooxazole 9d.**

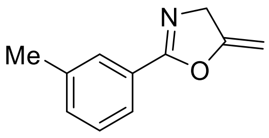

**9d**

After purification (Silica Gel, n-hexane/ethyl acetate 10:1) **9d** was isolated as a yellow oil in 99% yield (34.3 mg). $^1$H NMR (600 MHz, $CDCl_3$) $\delta$ 7.79 (s, 1H), 7.75 (d, $J$ = 6.6 Hz, 1H), 7.28 (d, $J$ = 6.9 Hz, 2H), 4.79 (q, $J$ = 2.9 Hz, 1H), 4.60 (t, $J$ = 2.8 Hz, 2H), 4.32 (q, $J$ = 2.6 Hz, 1H), 2.36 (s, 3H). $^{13}$C NMR (150 MHz, $CDCl_3$) $\delta$ 163.8, 158.9, 138.2, 132.5, 128.5, 128.3, 126.6, 125.1, 83.6, 57.7, 21.2. This compound showed identical spectroscopic properties to those reported previously.[12]

**2-(4-Methoxyphenyl)-5-methylene-4,5-dihydrooxazole 9e.**

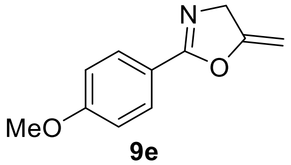

**9e**

After purification (Silica Gel, n-hexane/ethyl acetate 10:1) **9e** was isolated as a white solid in 95% yield (36.0 mg). $^1$H NMR (400 MHz, $CDCl_3$) $\delta$ 7.91 (d, $J$ = 8.5 Hz, 2H), 6.94 (d, $J$ = 8.5 Hz, 2H), 4.79 (t, $J$ = 2.8 Hz, 1H), 4.62 (t, $J$ = 2.6 Hz, 2H), 4.34 (t, $J$ = 2.7 Hz, 1H), 3.86 (s, 3H). $^{13}$C NMR (100 MHz, $CDCl_3$) $\delta$ 163.63, 162.56, 159.13, 129.90, 114.01, 83.55, 57.81, 55.53. This compound showed identical spectroscopic properties to those reported previously.[11]

**5-Methylene-2-(3,4,5-trimethoxyphenyl)-4,5-dihydrooxazole 9f.**

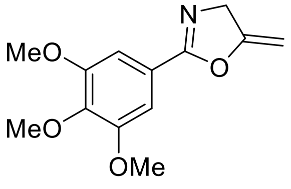

**9f**

After purification (Silica Gel, n-hexane/ethyl acetate 10:1) **9f** was isolated as a white solid in 74% yield (36.9 mg). $^1$H NMR (600 MHz, $CDCl_3$) $\delta$ 7.22 (s, 2H), 4.83 (q, $J$ = 2.9 Hz, 1H), 4.65 (t, $J$ = 2.7 Hz, 2H), 4.37 (q, $J$ = 2.6 Hz, 1H), 3.92 (s, 6H), 3.91 (s, 3H). $^{13}$C NMR (150 MHz, $CDCl_3$) $\delta$ 163.5, 158.8, 153.2, 141.2, 121.9, 105.1, 83.9, 61.0, 57.8, 56.3. This compound showed identical spectroscopic properties to those reported previously.[13]

**5-Methylene-2-(4-nitrophenyl)-4,5-dihydrooxazole 9g.**

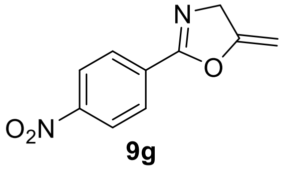

**9g**

After purification (Silica Gel, n-hexane/ethyl acetate 10:1) **9g** was isolated as a white solid in 50% yield (20.4 mg). $^1$H NMR (400 MHz, $CDCl_3$) $\delta$ 8.31 (d, $J$ = 8.5 Hz, 1H), 8.16 (d, $J$ = 8.4 Hz, 1H), 4.88 (d, $J$ = 3.3 Hz, 1H), 4.71 (d, $J$ = 3.3 Hz, 1H), 4.44 (d, $J$ = 3.1 Hz, 1H). $^{13}$C NMR (100 MHz, $CDCl_3$) $\delta$ 162.15, 158.43, 149.91, 132.60, 129.20, 123.87, 85.07, 58.16. HRMS calcd for $C_{10}H_8N_2O_3^+$ $[M+H]^+$ 205.0608, found 205.0606.

**4-(5-Methylene-4,5-dihydrooxazol-2-yl)benzonitrile 9h.**

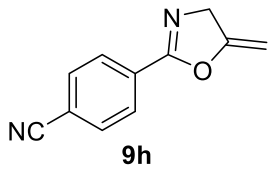


**9h**

After purification (Silica Gel, n-hexane/ethyl acetate 10:1) **9h** was isolated as a white solid in 51% yield (18.7 mg). $^1$H NMR (400 MHz, $CDCl_3$) $\delta$ 8.08 (d, $J$ = 7.9 Hz, 1H), 7.74 (d, $J$ = 7.9 Hz, 1H), 4.85 (d, $J$ = 2.7 Hz, 1H), 4.68 (d, $J$ = 2.3 Hz, 1H), 4.42 (d, $J$ = 2.6 Hz, 1H). $^{13}$C NMR (100 MHz, $CDCl_3$) $\delta$ 162.35, 158.44, 132.43, 130.94, 128.68, 118.23, 115.40, 84.92, 58.06. HRMS calcd for $C_{11}H_8N_2O^+$ $[M+H]^+$ 185.0709, found 185.0707.

**5-Methylene-2-(4-(trifluoromethyl)phenyl)-4,5-dihydrooxazole 9i.**

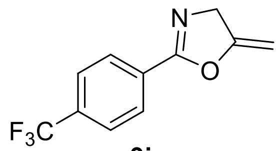


**9i**

After purification (Silica Gel, n-hexane/ethyl acetate 10:1) **9i** was isolated as a white solid in 95% yield (43.1 mg). $^1$H NMR (600 MHz, $CDCl_3$) $\delta$ 8.09 (d, $J$ = 8.1 Hz, 2H), 7.70 (d, $J$ = 8.2 Hz, 2H), 4.85 (q, $J$ = 3.0 Hz, 1H), 4.67 (t, $J$ = 2.7 Hz, 2H), 4.40 (q, $J$ = 2.7 Hz, 1H). $^{13}$C NMR (150 MHz, $CDCl_3$) $\delta$ 162.5, 158.5, 133.5, 133.3, 130.1, 128.4, 125.5, 125.5, 125.5, 125.4, 124.6, 122.8, 84.4, 57.8. $^{19}$F NMR (565 MHz, $CDCl_3$) $\delta$ -63.07. This compound showed identical spectroscopic properties to those reported previously.[11]

**5-Methylene-2-(2-(trifluoromethyl)phenyl)-4,5-dihydrooxazole 9j.**

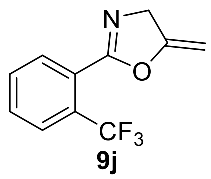


**9j**

After purification (Silica Gel, n-hexane/ethyl acetate 10:1) **9j** was isolated as a yellow liquid in 33% yield (15.0 mg). $^1$H NMR (600 MHz, $CDCl_3$) $\delta$ 7.83 (d, $J$ = 6.5 Hz, 1H), 7.77 (d, $J$ = 8.2 Hz, 1H), 7.61 (q, $J$ = 6.4, 4.1 Hz, 2H), 4.80 (q, $J$ = 3.0 Hz, 1H), 4.68 (t, $J$ = 2.8 Hz, 2H), 4.38 (q, $J$ = 2.7 Hz, 1H). $^{13}$C NMR (150 MHz, $CDCl_3$) $\delta$ 162.6, 158.7, 131.7, 131.1 (d, $J$ = 8.2 Hz), 129.2 (q, $J$ = 32.3 Hz), 126.7 (q, $J$ = 5.4 Hz), 126.4 (d, $J$ = 1.8 Hz), 123.3 (q, $J$ = 273.5 Hz), 84.3, 58.1. $^{19}$F NMR (565 MHz, $CDCl_3$) $\delta$ -59.7. HRMS calcd for $C_{11}H_8F_3NO^+$ $[M+H]^+$ 228.0631, found 228.0628.

**2-(4-Fluorophenyl)-5-methylene-4,5-dihydrooxazole 9k.**

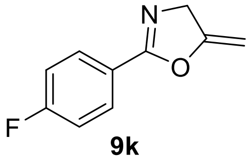

**9k**

After purification (Silica Gel, n-hexane/ethyl acetate 10:1) **9k** was isolated as a white solid in 97% yield (34.3 mg). $^1$H NMR (600 MHz, $CDCl_3$) $\delta$ 8.01 – 7.94 (m, 2H), 7.16 – 7.08 (m, 2H), 4.81 (q, $J$ = 3.0 Hz, 1H), 4.63 (t, $J$ = 2.9 Hz, 2H), 4.36 (q, $J$ = 2.8 Hz, 1H). $^{13}$C NMR (150 MHz, $CDCl_3$) $\delta$ 162.8, 158.8, 130.3, 123.0, 115.7, 115.60, 83.9, 57.7. $^{19}$F NMR (565 MHz, $CDCl_3$) $\delta$ -107.3. This compound showed identical spectroscopic properties to those reported previously.[11]

**2-(4-Chlorophenyl)-5-methylene-4,5-dihydrooxazole 9l.**

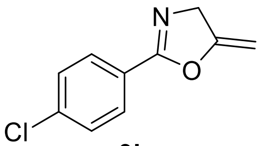

**9l**

After purification (Silica Gel, n-hexane/ethyl acetate 10:1) **9l** was isolated as a yellow solid in 95% yield (36.8 mg). $^1$H NMR (400 MHz, $CDCl_3$) $\delta$ 7.90 (d, $J$ = 8.1 Hz, 2H), 7.41 (d, $J$ = 8.1 Hz, 2H), 4.81 (d, $J$ = 3.6 Hz, 1H), 4.64 (s, 2H), 4.37 (s, 1H). $^{13}$C NMR (100 MHz, $CDCl_3$) $\delta$ 162.97, 158.84, 138.19, 129.46, 128.96, 125.44, 84.16, 57.92. HRMS calcd for $C_{10}H_8ClNO^+$ $[M+H]^+$ 194.0367, found 194.0367.

**2-(3-Chlorophenyl)-5-methylene-4,5-dihydrooxazole 9m.**

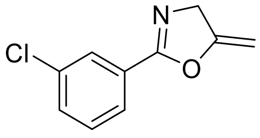

**9m**

After purification (Silica Gel, n-hexane/ethyl acetate 10:1) **9m** was isolated as a white solid in 83% yield (32.2 mg). $^1$H NMR (600 MHz, $CDCl_3$) $\delta$ 7.97 (s, 1H), 7.86 (d, $J$ = 7.8 Hz, 1H), 7.49 (d, $J$ = 9.0 Hz, 1H), 7.38 (t, $J$ = 7.9 Hz, 1H), 4.83 (q, $J$ = 3.0 Hz, 1H), 4.65 (t, $J$ = 2.8 Hz, 2H), 4.39 (q, $J$ = 2.7 Hz, 1H). $^{13}$C NMR (150 MHz, $CDCl_3$) $\delta$ 162.6, 158.6, 134.6, 131.8, 129.8, 128.5, 128.1, 126.1, 84.3, 57.8. This compound showed identical spectroscopic properties to those reported previously.[13]

**2-(4-Bromophenyl)-5-methylene-4,5-dihydrooxazole 9n.**

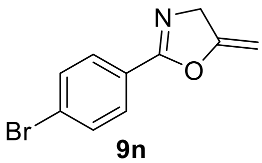

**9n**

After purification (Silica Gel, n-hexane/ethyl acetate 10:1) **9n** was isolated as a white solid in 98% yield (46.6 mg). $^1$H NMR (600 MHz, $CDCl_3$) $\delta$ 7.85 – 7.82 (m, 2H), 7.60

– 7.56 (m, 2H), 4.81 (q, $J$ = 3.1 Hz, 1H), 4.63 (t, $J$ = 2.9 Hz, 2H), 4.37 (q, $J$ = 2.7 Hz, 1H). $^{13}$C NMR (150 MHz, $CDCl_3$) $\delta$ 163.0, 158.6, 131.8, 129.5, 126.6, 125.7, 84.1, 57.8. HRMS calcd for $C_{10}H_8BrNO^+$ $[M+H]^+$ 237.9862, found 237.9861.

**5-Methylene-2-(naphthalen-2-yl)-4,5-dihydrooxazole 9o.**

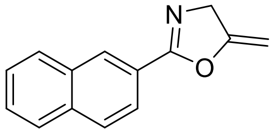


**9o**

After purification (Silica Gel, n-hexane/ethyl acetate 10:1) **9o** was isolated as a colorless oil in 85% yield (35.5 mg). $^{1}$H NMR (600 MHz, $CDCl_3$) $\delta$ 8.46 (s, 1H), 8.04 (d, $J$ = 8.6 Hz, 1H), 7.91 (d, $J$ = 7.8 Hz, 1H), 7.86 (dd, $J$ = 13.6, 8.2 Hz, 2H), 7.58 – 7.49 (m, 2H), 4.86 (q, $J$ = 3.0 Hz, 1H), 4.71 – 4.68 (m, 2H), 4.39 (q, $J$ = 2.6 Hz, 1H). $^{13}$C NMR (150 MHz, $CDCl_3$) $\delta$ 158.9, 134.9, 132.6, 129.0, 128.8, 128.4, 127.8, 127.8, 126.7, 124.2, 124.0, 83.8, 57.9. This compound showed identical spectroscopic properties to those reported previously.[13]

**(*E*)-5-methylene-2-styryl-4,5-dihydrooxazole 9p.**

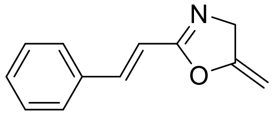


**9p**

After purification (Silica Gel, n-hexane/ethyl acetate 10:1) **9p** was isolated as a white solid in 98% yield (35.2 mg). $^{1}$H NMR (600 MHz, $CDCl_3$) $\delta$ 7.51 (d, $J$ = 7.5 Hz, 2H), 7.44 (d, $J$ = 16.3 Hz, 1H), 7.38 (dq, $J$ = 14.1, 6.9 Hz, 3H), 6.63 (d, $J$ = 16.3 Hz, 1H), 4.77 (q, $J$ = 2.9 Hz, 1H), 4.61 – 4.56 (m, 2H), 4.34 – 4.30 (m, 1H). $^{13}$C NMR (150 MHz, $CDCl_3$) $\delta$ 163.5, 158.5, 140.8, 134.9, 129.8, 128.9, 127.6, 114.1, 83.4, 57.8. This compound showed identical spectroscopic properties to those reported previously.[6]

**5-Methylene-2-(thiophen-2-yl)-4,5-dihydrooxazole 9q.**

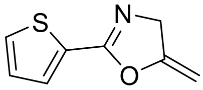


**9q**

After purification (Silica Gel, n-hexane/ethyl acetate 10:1) **9q** was isolated as a yellow oil in 84% yield (27.7 mg). $^{1}$H NMR (600 MHz, $CDCl_3$) $\delta$ 7.65 (d, $J$ = 3.6 Hz, 1H), 7.49 (d, $J$ = 5.0 Hz, 1H), 7.12 – 7.08 (m, 1H), 4.80 (q, $J$ = 3.0 Hz, 1H), 4.61 (t, $J$ = 2.8 Hz, 2H), 4.35 (q, $J$ = 2.6 Hz, 1H). $^{13}$C NMR (150 MHz, $CDCl_3$) $\delta$ 159.5, 158.6, 130.7, 130.4, 129.3, 127.7, 84.0, 57.7. This compound showed identical spectroscopic properties to those reported previously.[14]

**3-(5-Methylene-4,5-dihydrooxazol-2-yl)-*N*-(prop-2-yn-1-yl)benzamide 9r.**

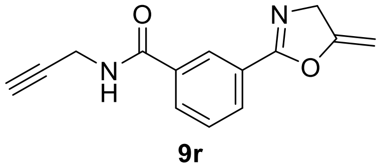


**9r**

After purification (Silica Gel, n-hexane/ethyl acetate 10:4) **9r** was isolated as a white solid in 45% yield (21.6 mg). $^{1}$H NMR (600 MHz, $CDCl_3$) $\delta$ 8.32 (s, 1H), 8.12 (d, $J$ = 7.8 Hz, 1H), 8.03 (d, $J$ = 7.8 Hz, 1H), 7.55 (t, $J$ = 7.8 Hz, 1H), 6.41 (s, 1H), 4.86 (q, $J$ = 3.0 Hz, 1H), 4.68 (t, $J$ = 2.8 Hz, 2H), 4.41 (q, $J$ = 2.7 Hz, 1H), 4.28 (dd, $J$ = 5.1, 2.5 Hz, 2H), 2.30 (t, $J$ = 2.5 Hz, 1H). $^{13}$C NMR (150 MHz, $CDCl_3$) $\delta$ 166.0, 163.0, 158.5, 134.2, 131.1, 131.0, 129.2, 127.2, 125.9, 84.4, 79.2, 72.2, 57.8, 29.9. HRMS calcd for $C_{14}H_{12}N_2O_2^+$ $[M+H]^+$ 241.0972, found 241.0969.

**5-Methylene-2-(1-phenylpropyl)-4,5-dihydrooxazole 9s.**

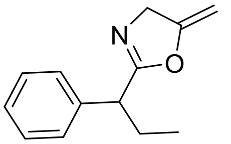


**9s**

After purification (Silica Gel, n-hexane/ethyl acetate 10:1) **9s** was isolated as a colorless oil in 80% yield (32.2 mg). $^{1}$H NMR (600 MHz, $CDCl_3$) $\delta$ 7.32 (d, $J$ = 5.0 Hz, 4H), 7.25 (t, $J$ = 7.0 Hz, 1H), 4.61 (q, $J$ = 3.0 Hz, 1H), 4.42 (d, $J$ = 2.5 Hz, 2H), 4.21 (q, $J$ = 2.6 Hz, 1H), 3.52 (t, $J$ = 7.7 Hz, 1H), 2.18 – 2.09 (m, 1H), 1.88 (td, $J$ = 14.4, 13.9, 7.5 Hz, 1H), 0.91 (t, $J$ = 7.4 Hz, 3H). $^{13}$C NMR (150 MHz, $CDCl_3$) $\delta$ 158.9, 128.6, 128.0, 127.3, 83.3, 57.2, 47.3, 26.7, 12.1. HRMS calcd for $C_{13}H_{16}NO^+$ $[M+H]^+$ 202.1227, found 202.1224.

**2-Methyl-5-methylene-4,5-dihydrooxazole 9t.**

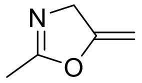


**9t**

An oven-dried vial equipped with a stir bar was charged with *N*-(prop-2-yn-1-yl)acetamide (19.4 mg, 0.2 mmol, 1.0 eq.), Ag catalyst **4c** (1.5 mg, 1.0 mol%). The reaction mixture was placed under a positive pressure of argon and subjected to three evacuation/backfilling cycles under high vacuum. Then AcOH (12.0 mg, 0.2 mmol, 2.0 eq.) and $CDCl_3$ (0.2 ml, 1.0 M) was added and the reaction mixture was stirred at room temperature for 8 h. The reaction mixture without further purification was used to measure $^{1}$H NMR yield directly. $^{1}$H NMR (600 MHz, $CDCl_3$) $\delta$ 4.90 (q, $J$ = 3.2 Hz, 1H), 4.64 (s, 2H), 4.64 – 4.54 (m, 1H), 2.43 (s, 3H).

**2-(*tert*-Butyl)-5-methylene-4,5-dihydrooxazole 9u.**

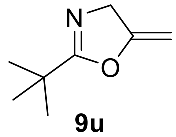


**9u**

After purification (Silica Gel, n-hexane/ethyl acetate 10:1) **9u** was isolated as a colorless liquid in 42% yield (11.7 mg). $^{1}$H NMR (600 MHz, $CDCl_3$) $\delta$ 4.64 (q, $J$ = 2.8 Hz, 1H), 4.41 (t, $J$ = 2.7 Hz, 2H), 4.25 – 4.21 (m, 1H), 1.26 (s, 9H). $^{13}$C NMR (150 MHz, $CDCl_3$) $\delta$ 173.8, 159.5, 82.7, 57.3, 33.3, 27.3. This compound showed identical spectroscopic properties to those reported previously.[8]

**2-Cyclohexyl-5-methylene-4,5-dihydrooxazole 9v.**

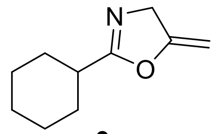

**9v**

After purification (Silica Gel, n-hexane/ethyl acetate 10:1) **9v** was isolated as a colorless liquid in 78% yield (25.8 mg). $^{1}$H NMR (600 MHz, $CDCl_3$) $\delta$ 4.63 (q, $J$ = 2.9 Hz, 1H), 4.43 – 4.35 (m, 2H), 4.23 (q, $J$ = 2.5 Hz, 1H), 2.38 – 2.32 (m, 1H), 2.00 – 1.92 (m, 2H), 1.83 – 1.75 (m, 2H), 1.70 – 1.65 (m, 1H), 1.50 – 1.41 (m, 2H), 1.34 – 1.24 (m, 3H). $^{13}$C NMR (150 MHz, $CDCl_3$) $\delta$ 170.6, 159.1, 57.1, 37.3, 29.3, 25.8, 25.5. This compound showed identical spectroscopic properties to those reported previously.[15]

**2-Hexyl-5-methylene-4,5-dihydrooxazole 9w.**

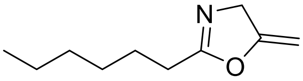

**9w**

After purification (Silica Gel, n-hexane/ethyl acetate 10:1) **9w** was isolated as a colorless liquid in 52% yield (17.4 mg). $^{1}$H NMR (600 MHz, $CDCl_3$) $\delta$ 4.64 (q, $J$ = 2.8 Hz, 1H), 4.43 – 4.36 (m, 2H), 4.27 – 4.20 (m, 1H), 2.38 – 2.30 (m, 2H), 1.66 (p, $J$ = 7.6 Hz, 2H), 1.36 (dt, $J$ = 14.2, 7.4 Hz, 3H), 1.31 – 1.29 (m, 3H), 0.89 (t, $J$ = 6.7 Hz, 3H). $^{13}$C NMR (150 MHz, $CDCl_3$) $\delta$ 167.7, 159.1, 82.8, 57.2, 31.4, 28.8, 28.1, 25.4, 22.5, 14.0. This compound showed identical spectroscopic properties to those reported previously.[16]

**2-Cyclohexyl-4,4-dimethyl-5-methylene-4,5-dihydrooxazole 9x.**

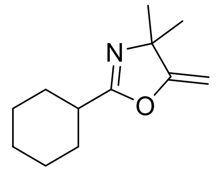

**9x**

After purification (Silica Gel, n-hexane/ethyl acetate 10:1) **9x** was isolated as a colorless liquid in 80% yield (30.9 mg). $^{1}$H NMR (600 MHz, $CDCl_3$) $\delta$ 4.55 (d, $J$ = 2.7 Hz, 1H), 4.11 (d, $J$ = 2.7 Hz, 1H), 2.34 (tt, $J$ = 11.4, 3.5 Hz, 1H), 1.96 (d, $J$ = 13.1 Hz, 2H), 1.82 – 1.75 (m, 2H), 1.67 (d, $J$ = 11.5 Hz, 1H), 1.50 – 1.43 (m, 2H), 1.32 (s, 6H), 1.30 – 1.20 (m, 2H). $^{13}$C NMR (150 MHz, $CDCl_3$) $\delta$ 168.1, 166.6, 81.1, 68.0, 37.1, 29.5, 29.4, 25.7, 25.4. HRMS calcd for $C_{12}H_{19}NO^+$ $[M+H]^+$ 194.1539, found 194.1538.

**4,4-Dimethyl-5-methylene-2-(4-nitrophenyl)-4,5-dihydrooxazole 9y.**

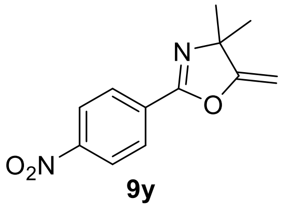

**9y**

After purification (Silica Gel, n-hexane/ethyl acetate 10:1) **9y** was isolated as a white

solid in 98% yield (45.5 mg). $^{1}$H NMR (600 MHz, $CDCl_3$) $\delta$ 8.30 (d, $J$ = 9.0 Hz, 2H), 8.17 (d, $J$ = 9.0 Hz, 2H), 4.80 (d, $J$ = 3.1 Hz, 1H), 4.32 (d, $J$ = 3.1 Hz, 1H), 1.48 (s, 6H). $^{13}$C NMR (150 MHz, $CDCl_3$) $\delta$ 167.5, 158.3, 149.8, 132.9, 129.3, 123.8, 83.6, 77.4, 77.2, 77.0, 69.8, 29.8, 29.8, 14.3. This compound showed identical spectroscopic properties to those reported previously.[17]

**4,4-Dimethyl-5-methylene-2-phenyl-4,5-dihydrooxazole 9z.**

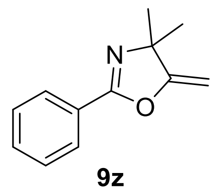

**9z**

After purification (Silica Gel, n-hexane/ethyl acetate 10:1) **9z** was isolated as a colorless liquid in 98% yield (36.7 mg). $^{1}$H NMR (400 MHz, $CDCl_3$) $\delta$ 7.99 (d, $J$ = 7.4 Hz, 2H), 7.53 – 7.46 (m, 1H), 7.43 (t, $J$ = 7.3 Hz, 2H), 4.74 (s, 1H), 4.25 (s, 1H), 1.45 (s, 6H). $^{13}$C NMR (100 MHz, $CDCl_3$) $\delta$ 168.00, 159.93, 131.75, 128.54, 128.19, 127.07, 82.41, 69.17, 29.85. This compound showed identical spectroscopic properties to those reported previously.[11]

**4,4-Dimethyl-5-methylene-2-(*o*-tolyl)-4,5-dihydrooxazole 9aa.**

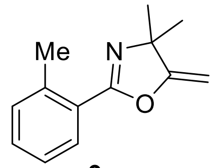

**9aa**

After purification (Silica Gel, n-hexane/ethyl acetate 10:1) **9aa** was isolated as a colorless liquid in 98% yield (39.4 mg). $^{1}$H NMR (600 MHz, $CDCl_3$) $\delta$ 7.82 (d, $J$ = 8.4 Hz, 1H), 7.31 (t, $J$ = 7.5 Hz, 1H), 7.22 (d, $J$ = 7.5 Hz, 2H), 4.69 (d, $J$ = 2.7 Hz, 1H), 4.20 (d, $J$ = 2.7 Hz, 1H), 2.60 (s, 3H), 1.44 (s, 6H). $^{13}$C NMR (150 MHz, $CDCl_3$) $\delta$ 167.7, 160.1, 139.0, 131.3, 130.8, 129.8, 126.4, 125.6, 81.8, 69.4, 29.9, 21.7. HRMS calcd for $C_{13}H_{15}NO^+$ $[M+H]^+$ 202.1226, found 202.1225.

**(*Z*)-5-Benzylidene-4,4-dimethyl-2-phenyl-4,5-dihydrooxazole 9ab.**

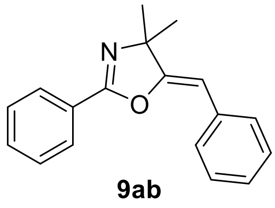

**9ab**

After purification (Silica Gel, n-hexane/ethyl acetate 10:1) **9ab** was isolated as a white solid in 98% yield (51.6 mg). $^{1}$H NMR (600 MHz, $CDCl_3$) $\delta$ 8.09 (d, $J$ = 7.1 Hz, 2H), 7.65 (d, $J$ = 7.4 Hz, 2H), 7.53 (t, $J$ = 7.3 Hz, 1H), 7.48 (t, $J$ = 7.4 Hz, 2H), 7.39 (t, $J$ = 7.8 Hz, 2H), 7.21 (t, $J$ = 7.6 Hz, 1H), 5.53 (s, 1H), 1.53 (s, 6H). $^{13}$C NMR (150 MHz, $CDCl_3$) $\delta$ 160.7, 159.9, 135.1, 131.9, 128.6, 128.6, 128.3, 128.0, 126.9, 126.2, 99.4, 77.3, 77.1, 76.9, 70.9, 29.7. This compound showed identical spectroscopic properties to those reported previously.[6]

**(*Z*)-5-Benzylidene-4,4-dimethyl-2-(*p*-tolyl)-4,5-dihydrooxazole 9ac.**

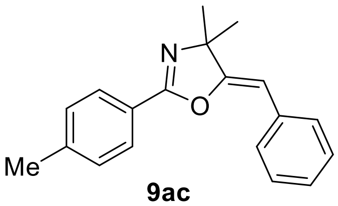

**9ac**

After purification (Silica Gel, n-hexane/ethyl acetate 10:1) **9ac** was isolated as a white solid in 97% yield (53.8 mg). $^1$H NMR (600 MHz, $CDCl_3$) $\delta$ 7.97 (d, $J$ = 8.1 Hz, 2H), 7.64 (d, $J$ = 8.0 Hz, 2H), 7.38 (t, $J$ = 7.7 Hz, 2H), 7.27 (d, $J$ = 8.0 Hz, 2H), 7.20 (t, $J$ = 7.4 Hz, 1H), 5.51 (s, 1H), 2.40 (s, 3H), 1.52 (s, 6H). $^{13}$C NMR (150 MHz, $CDCl_3$) $\delta$ 160.8, 160.0, 142.4, 135.2, 129.4, 128.6, 128.3, 128.0, 126.2, 124.1, 99.2, 70.8, 29.7, 21.7. HRMS calcd for $C_{19}H_{20}NO^+$ $[M+H]^+$ 278.1540, found 278.1536.

**(*Z*)-5-Benzylidene-4,4-dimethyl-2-(4-nitrophenyl)-4,5-dihydrooxazole 9ad.**

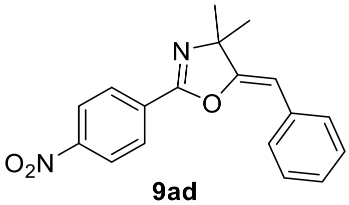

**9ad**

After purification (Silica Gel, n-hexane/ethyl acetate 10:1) **9ad** was isolated as a yellow solid in 86% yield (53.0 mg). $^1$H NMR (600 MHz, $CDCl_3$) $\delta$ 8.33 (d, $J$ = 8.9 Hz, 2H), 8.25 (d, $J$ = 8.9 Hz, 2H), 7.62 (d, $J$ = 7.4 Hz, 2H), 7.41 (t, $J$ = 7.8 Hz, 2H), 7.24 (t, $J$ = 7.4 Hz, 1H), 5.59 (s, 1H), 1.56 (s, 6H). $^{13}$C NMR (150 MHz, $CDCl_3$) $\delta$ 159.9, 158.2, 149.8, 134.6, 132.6, 129.3, 128.6, 128.0, 126.6, 123.8, 100.4, 77.3, 77.1, 76.9, 71.4, 29.6. HRMS calcd for $C_{18}H_{17}N_2O_3^+$ $[M+H]^+$ 309.1234, found 309.1230.

**(*Z*)-5-Benzylidene-4,4-dimethyl-2-(thiophen-2-yl)-4,5-dihydrooxazole 9ae.**

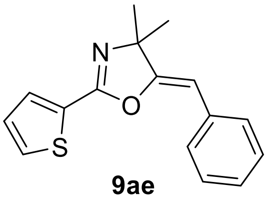

**9ae**

After purification (Silica Gel, n-hexane/ethyl acetate 10:1) **9ae** was isolated as a white solid in 83% yield (64.9 mg). $^1$H NMR (600 MHz, $CDCl_3$) $\delta$ 7.78 (d, $J$ = 3.6 Hz, 1H), 7.62 (d, $J$ = 7.8 Hz, 2H), 7.51 (d, $J$ = 4.9 Hz, 1H), 7.37 (t, $J$ = 7.6 Hz, 2H), 7.21 (t, $J$ = 7.3 Hz, 1H), 7.14 (t, $J$ = 4.3 Hz, 1H), 5.52 (s, 1H), 1.52 (s, 6H). $^{13}$C NMR (150 MHz, $CDCl_3$) $\delta$ 160.4, 155.6, 134.9, 131.0, 130.5, 129.4, 128.6, 128.0, 127.8, 126.3, 99.5, 71.1, 29.7. HRMS calcd for $C_{16}H_{16}NOS^+$ $[M+H]^+$ 270.0948, found 270.0942.

**2-(5-(2-Chloro-4-(trifluoromethyl)phenoxy)-2-nitrophenyl)-5-methylene-4,5-dihydrooxazole 9af.**

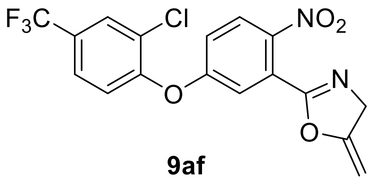


**9af**

After purification (Silica Gel, n-hexane/ethyl acetate 10:2) **9af** was isolated as a white solid in 45% yield (35.9 mg). $^{1}$H NMR (600 MHz, $CDCl_3$) $\delta$ 8.01 (d, $J$ = 9.0 Hz, 1H), 7.81 (s, 1H), 7.61 (d, $J$ = 8.9 Hz, 1H), 7.29 (d, $J$ = 2.7 Hz, 1H), 7.25 (d, $J$ = 8.5 Hz, 1H), 7.14 (d, $J$ = 9.0 Hz, 1H), 4.77 (q, $J$ = 3.1 Hz, 1H), 4.65 (t, $J$ = 2.8 Hz, 2H), 4.40 (q, $J$ = 2.7 Hz, 1H), 1.59 (d, $J$ = 1.2 Hz, 2H). $^{13}$C NMR (150 MHz, $CDCl_3$) $\delta$ 160.7, 159.5, 158.2, 152.9, 143.9, 128.8 (q, $J$ = 3.7 Hz), 127.5, 126.9, 125.8 (q, $J$ = 3.1 Hz), 125.3, 123.9, 122.4, 122.1, 119.3, 119.0, 85.1, 57.9. $^{19}$F NMR (565 MHz, $CDCl_3$) δ -62.4. HRMS calcd for $C_{17}H_{11}ClF_3N_2O_4^+$ $[M+H]^+$ 399.0354, found 399.0349.

**2-(1,3-Dihydroisobenzofuran-5-yl)-5-methylene-4,5-dihydrooxazole 9ag.**

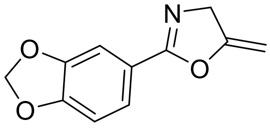


**9ag**

After purification (Silica Gel, n-hexane/ethyl acetate 10:1) **9ag** was isolated as a white solid in 71% yield (28.6 mg). $^{1}$H NMR (600 MHz, $CDCl_3$) $\delta$ 7.52 (d, $J$ = 9.6 Hz, 1H), 7.42 (s, 1H), 6.84 (d, $J$ = 8.1 Hz, 1H), 6.02 (s, 2H), 4.78 (q, $J$ = 2.9 Hz, 1H), 4.61 (t, $J$ = 2.8 Hz, 2H), 4.33 (q, $J$ = 2.6 Hz, 1H). $^{13}$C NMR (150 MHz, $CDCl_3$) $\delta$ 163.2, 158.9, 150.6, 147.8, 123.2, 120.7, 108.2, 108.0, 101.7, 83.5, 57.7. HRMS calcd for $C_{11}H_9NO_3^+$ $[M+H]^+$ 204.0655, found 204.0654.

**2-(3,6-Dichloro-2-methoxyphenyl)-5-methylene-4,5-dihydrooxazole 9ah.**

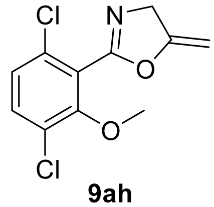


**9ah**

After purification (Silica Gel, n-hexane/ethyl acetate 10:1) **9ah** was isolated as a yellow liquid in 71% yield (28.6 mg). $^{1}$H NMR (600 MHz, $CDCl_3$) $\delta$ 7.41 (d, $J$ = 8.7 Hz, 1H), 7.16 (d, $J$ = 8.7 Hz, 1H), 4.79 (q, $J$ = 3.0 Hz, 1H), 4.72 (t, $J$ = 2.9 Hz, 2H), 4.41 (q, $J$ = 2.7 Hz, 1H), 3.92 (s, 3H). $^{13}$C NMR (150 MHz, $CDCl_3$) $\delta$ 159.2, 158.4, 155.9, 132.7, 132.6, 126.9, 125.8, 124.9, 84.5, 62.4, 58.0. HRMS calcd for $C_{11}H_9Cl_2NO_2^+$ $[M+H]^+$ 258.0083, found 258.0083.

**2-Isobutoxy-5-(4-methyl-5-(5-methylene-4,5-dihydrooxazol-2-yl)thiazol-2-yl)benzonitrile 9ai.**

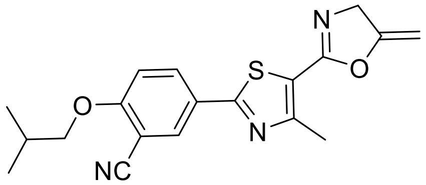

**9ai**

After purification (Silica Gel, n-hexane/ethyl acetate 10:1) **9ai** was isolated as a white solid in 71% yield (50.1 mg). $^1$H NMR (600 MHz, $CDCl_3$) $\delta$ 8.16 (d, $J$ = 2.2 Hz, 1H), 8.06 (d, $J$ = 8.8 Hz, 1H), 7.01 (d, $J$ = 8.9 Hz, 1H), 4.82 (q, $J$ = 2.9 Hz, 1H), 4.65 (t, $J$ = 2.5 Hz, 2H), 4.40 (q, $J$ = 2.6 Hz, 1H), 3.90 (d, $J$ = 6.5 Hz, 2H), 2.76 (s, 3H), 2.20 (dp, $J$ = 13.3, 6.6 Hz, 1H), 1.09 (d, $J$ = 6.7 Hz, 6H). $^{13}$C NMR (150 MHz, $CDCl_3$) $\delta$ 166.1, 162.4, 158.2, 157.9, 132.5, 131.9, 126.0, 118.1, 115.4, 112.6, 103.0, 84.4, 75.7, 57.7, 28.2, 19.1, 17.5. HRMS calcd for $C_{19}H_{20}N_3O_2S^+$ $[M+H]^+$ 354.1271, found 354.1265.

**4-(5-Methylene-4,5-dihydrooxazol-2-yl)-*N*,*N*-dipropylbenzenesulfonamide 9aj.**

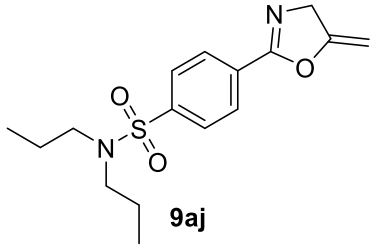

**9aj**

After purification (Silica Gel, n-hexane/ethyl acetate 10:1) **9aj** was isolated as a white solid in 65% yield (41.9 mg). $^1$H NMR (600 MHz, $CDCl_3$) $\delta$ 8.10 (d, $J$ = 8.4 Hz, 2H), 7.88 (d, $J$ = 8.4 Hz, 2H), 4.85 (q, $J$ = 2.9 Hz, 1H), 4.68 (t, $J$ = 2.7 Hz, 2H), 4.42 (q, $J$ = 2.5 Hz, 1H), 3.14 – 3.07 (m, 4H), 1.55 (h, $J$ = 7.4 Hz, 4H), 0.87 (d, $J$ = 14.8 Hz, 6H). $^{13}$C NMR (150 MHz, $CDCl_3$) $\delta$ 162.5, 158.4, 143.2, 130.2, 128.6, 127.1, 84.6, 57.9, 49.9, 21.9, 11.2. HRMS calcd for $C_{16}H_{23}N_2O_3S^+$ $[M+H]^+$ 323.1424, found 323.1419.

## Supplementary Method 10: Mechanism Studies

The catalysis is consistent with the dissociation of [NHC–M]X to $[NHC–M]^+$ and $[NHC–M]^+$ as the active catalyst. When catalyst **4c** reacts with AcOH, AgOAc and $^7$IPrS-Ag-OAc are not formed, but the thiazolium salt **3c** and $[NHC–Ag]^+$ are obtained (Supplementary Figure S1). Complexes $[NHC–Ag]_2ClO_4$ (NHC = **3a–3d**) are bench-stable and easy to handle. However, to eliminate the effect of counterion, we have also prepared and tested thiazol-2-ylidene $[NHC–Ag]_2PF_6$ (NHC = **3c**, 90% yield), imidazol-2-ylidene $[NHC–Ag]_2ClO_4$ (NHC = IPr, <5% yield, IMes, 30% yield) and imidazol-2-ylidene $[NHC–Ag]_2PF_6$ (NHC = IPr, <5% yield, IMes, 21% yield). These results further demonstrate the superior effect of N-aryl thiazol-2-ylidene Ag(I) complexes.

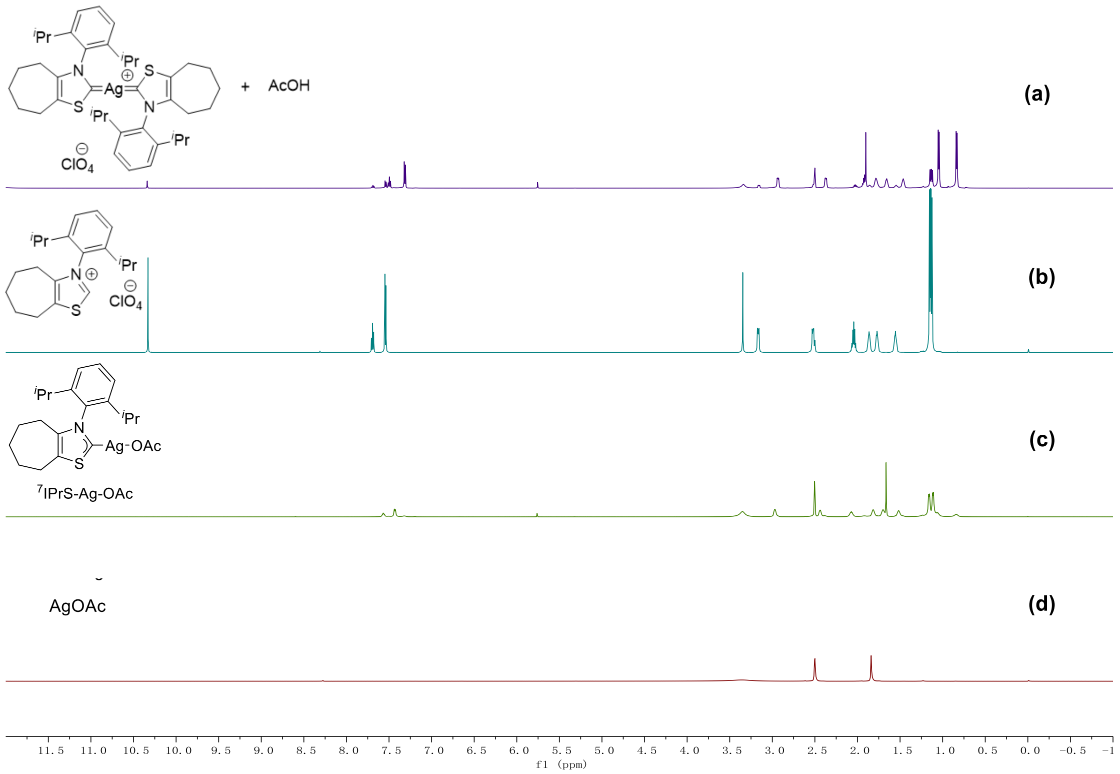


**Supplementary Figure S1.** $^1$H NMR spectra of (a) catalyst **4c** and AcOH, (b) thiazolium salt **3c**, (c) $^7$IPrS-Ag-OAc, and (d) AgOAc.

# Supplementary Notes

## Supplementary Note 1: Crystallographic Studies

### A. Crystal Structure of 4a.

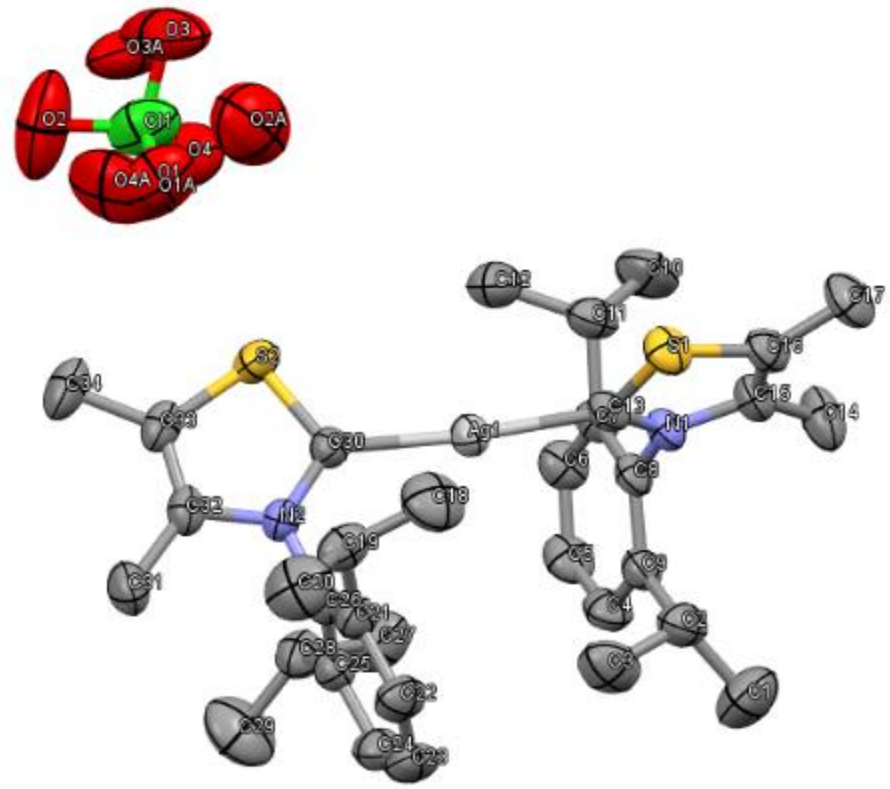


**Supplementary Figure S2.** Crystal structure of **4a** *(50% ellipsoids)*. (Crystallographic data has been deposited with the Cambridge Crystallographic Data Center as supplementary publication no. CCDC 2117719.)

**Supplementary Table S2.** *Crystal Data and Structure Refinement Summary for* ***4a****.*

*Crystal data*

| | |
|---|---|
| $C_{34}H_{46}AgN_2S_2{\cdot}ClO_4$ | $F(000) = 1568$ |
| $M_r = 754.17$ | $D_x = 1.360$ Mg m$^{-3}$ |
| Monoclinic, $P2_1/c$ | Mo $K\alpha$ radiation, $\lambda = 0.71073$ Å |
| $a = 9.4811$ (4) Å | Cell parameters from 9367 reflections |
| $b = 19.0223$ (9) Å | $\theta = 2.4$–$27.5°$ |
| $c = 20.5566$ (10) Å | $\mu = 0.77$ mm$^{-1}$ |
| $\beta = 96.456$ (2)° | $T = 273$ K |
| $V = 3683.9$ (3) Å$^3$ | Block |

| | |
|---|---|
| $Z = 4$ | 0.11 × 0.1 × 0.09 mm |

*Data collection*

| | |
|---|---|
| Bruker APEX-II CCD diffractometer | $R_{int} = 0.027$ |
| $\phi$ and $\omega$ scans | $\theta_{max} = 25.0°$, $\theta_{min} = 2.4°$ |
| 32295 measured reflections | $h = -11 \rightarrow 10$ |
| 6494 independent reflections | $k = -22 \rightarrow 22$ |
| 5766 reflections with $I > 2\sigma(I)$ | $l = -22 \rightarrow 24$ |

*Refinement*

| | |
|---|---|
| Refinement on $F^2$ | Primary atom site location: dual |
| Least-squares matrix: full | Hydrogen site location: inferred from neighbouring sites |
| $R[F^2 > 2\sigma(F^2)] = 0.033$ | H-atom parameters constrained |
| $wR(F^2) = 0.099$ | $w = 1/[\sigma^2(F_o^2) + (0.0522P)^2 + 1.8745P]$ where $P = (F_o^2 + 2F_c^2)/3$ |
| $S = 1.13$ | $(\Delta/\sigma)_{max} = 0.001$ |
| 6494 reflections | $\Delta\rangle_{max} = 0.44$ e Å$^{-3}$ |
| 446 parameters | $\Delta\rangle_{min} = -0.73$ e Å$^{-3}$ |
| 177 restraints | |

*Special details*

| |
|---|
| *Geometry*. All esds (except the esd in the dihedral angle between two l.s. planes) are estimated using the full covariance matrix. The cell esds are taken into account individually in the estimation of esds in distances, angles and torsion angles; correlations between esds in cell parameters are only used when they are defined by crystal symmetry. An approximate (isotropic) treatment of cell esds is used for estimating esds involving l.s. planes. |

*Fractional atomic coordinates and isotropic or equivalent isotropic displacement parameters (Å2) for (**4a**).*

| | $x$ | $y$ | $z$ | $U_{iso}*/U_{eq}$ | Occ. (<1) |
|---|---|---|---|---|---|
| Ag1 | 0.61719 (2) | 0.28206 (2) | 0.48143 (2) | 0.04307 (9) | |

| | | | | |
|---|---|---|---|---|
| S1 | 0.27313 (8) | 0.32322 (4) | 0.48536 (4) | 0.05116 (19) |
| S2 | 0.86717 (9) | 0.31784 (4) | 0.38347 (4) | 0.05049 (19) |
| N1 | 0.4337 (2) | 0.33209 (12) | 0.58710 (11) | 0.0395 (5) |
| N2 | 0.8588 (2) | 0.19804 (12) | 0.42823 (11) | 0.0371 (5) |
| C1 | 0.4772 (6) | 0.1839 (3) | 0.7388 (2) | 0.0990 (15) |
| H1A | 0.5644 | 0.1646 | 0.7596 | 0.148* |
| H1B | 0.4055 | 0.1481 | 0.7347 | 0.148* |
| H1C | 0.4473 | 0.2218 | 0.7649 | 0.148* |
| C2 | 0.5000 (4) | 0.21148 (16) | 0.67099 (18) | 0.0570 (8) |
| H2 | 0.4070 | 0.2253 | 0.6491 | 0.068* |
| C3 | 0.5565 (4) | 0.15383 (18) | 0.6304 (2) | 0.0749 (11) |
| H3A | 0.5667 | 0.1715 | 0.5874 | 0.112* |
| H3B | 0.4915 | 0.1150 | 0.6270 | 0.112* |
| H3C | 0.6472 | 0.1385 | 0.6509 | 0.112* |
| C4 | 0.7202 (3) | 0.27836 (17) | 0.71499 (16) | 0.0528 (8) |
| H4 | 0.7458 | 0.2407 | 0.7427 | 0.063* |
| C5 | 0.8077 (3) | 0.33618 (18) | 0.71578 (15) | 0.0544 (8) |
| H5 | 0.8925 | 0.3366 | 0.7433 | 0.065* |
| C6 | 0.7716 (3) | 0.39377 (17) | 0.67621 (15) | 0.0520 (7) |
| H6 | 0.8314 | 0.4326 | 0.6780 | 0.062* |
| C7 | 0.6472 (3) | 0.39393 (15) | 0.63406 (14) | 0.0435 (6) |
| C8 | 0.5627 (3) | 0.33373 (14) | 0.63307 (13) | 0.0387 (6) |
| C9 | 0.5943 (3) | 0.27541 (15) | 0.67344 (14) | 0.0445 (6) |
| C10 | 0.5880 (6) | 0.5232 (2) | 0.6331 (3) | 0.0957 (15) |
| H10A | 0.5182 | 0.5138 | 0.6624 | 0.143* |
| H10B | 0.5572 | 0.5619 | 0.6051 | 0.143* |
| H10C | 0.6768 | 0.5348 | 0.6580 | 0.143* |
| C11 | 0.6071 (3) | 0.45810 (16) | 0.59152 (17) | 0.0557 (8) |
| H11 | 0.5156 | 0.4482 | 0.5659 | 0.067* |
| C12 | 0.7151 (4) | 0.4715 (2) | 0.54347 (19) | 0.0698 (10) |
| H12A | 0.8028 | 0.4870 | 0.5669 | 0.105* |
| H12B | 0.6796 | 0.5070 | 0.5127 | 0.105* |
| H12C | 0.7310 | 0.4288 | 0.5204 | 0.105* |

| | | | | |
|---|---|---|---|---|
| C13 | 0.4387 (3) | 0.31136 (15) | 0.52536 (14) | 0.0414 (6) |
| C14 | 0.2892 (4) | 0.3790 (2) | 0.67240 (17) | 0.0712 (10) |
| H14A | 0.3186 | 0.3411 | 0.7018 | 0.107* |
| H14B | 0.1918 | 0.3903 | 0.6760 | 0.107* |
| H14C | 0.3475 | 0.4194 | 0.6835 | 0.107* |
| C15 | 0.3045 (3) | 0.35732 (16) | 0.60441 (15) | 0.0465 (7) |
| C16 | 0.2025 (3) | 0.35730 (16) | 0.55263 (15) | 0.0487 (7) |
| C17 | 0.0547 (3) | 0.3847 (2) | 0.5494 (2) | 0.0689 (10) |
| H17A | 0.0386 | 0.4181 | 0.5143 | 0.103* |
| H17B | 0.0413 | 0.4073 | 0.5900 | 0.103* |
| H17C | -0.0112 | 0.3465 | 0.5418 | 0.103* |
| C18 | 0.4797 (4) | 0.1361 (3) | 0.3718 (2) | 0.0865 (12) |
| H18A | 0.4246 | 0.1005 | 0.3903 | 0.130* |
| H18B | 0.4368 | 0.1476 | 0.3286 | 0.130* |
| H18C | 0.4831 | 0.1774 | 0.3988 | 0.130* |
| C19 | 0.6303 (3) | 0.10896 (18) | 0.36828 (15) | 0.0555 (8) |
| H19 | 0.6828 | 0.1458 | 0.3479 | 0.067* |
| C20 | 0.6285 (5) | 0.0444 (2) | 0.3249 (2) | 0.0902 (13) |
| H20A | 0.7234 | 0.0267 | 0.3250 | 0.135* |
| H20B | 0.5912 | 0.0566 | 0.2810 | 0.135* |
| H20C | 0.5699 | 0.0088 | 0.3413 | 0.135* |
| C21 | 0.7071 (3) | 0.09518 (15) | 0.43613 (14) | 0.0453 (6) |
| C22 | 0.6683 (4) | 0.03871 (17) | 0.47310 (17) | 0.0575 (8) |
| H22 | 0.5951 | 0.0093 | 0.4560 | 0.069* |
| C23 | 0.7367 (4) | 0.02563 (17) | 0.53457 (18) | 0.0626 (9) |
| H23 | 0.7073 | -0.0117 | 0.5590 | 0.075* |
| C24 | 0.8484 (4) | 0.06734 (17) | 0.56045 (16) | 0.0571 (8) |
| H24 | 0.8954 | 0.0568 | 0.6015 | 0.069* |
| C25 | 0.8912 (3) | 0.12461 (16) | 0.52606 (14) | 0.0458 (7) |
| C26 | 0.8177 (3) | 0.13743 (14) | 0.46458 (13) | 0.0387 (6) |
| C27 | 0.9637 (5) | 0.2128 (2) | 0.6124 (2) | 0.0765 (11) |
| H27A | 0.8810 | 0.2398 | 0.5973 | 0.115* |
| H27B | 1.0385 | 0.2439 | 0.6296 | 0.115* |

| | | | | | |
|---|---|---|---|---|---|
| H27C | 0.9415 | 0.1811 | 0.6462 | 0.115* | |
| C28 | 1.0113 (3) | 0.17104 (18) | 0.55582 (15) | 0.0549 (8) | |
| H28 | 1.0342 | 0.2044 | 0.5222 | 0.066* | |
| C29 | 1.1446 (5) | 0.1289 (3) | 0.5779 (3) | 0.1036 (16) | |
| H29A | 1.1306 | 0.1027 | 0.6166 | 0.155* | |
| H29B | 1.2235 | 0.1603 | 0.5873 | 0.155* | |
| H29C | 1.1638 | 0.0970 | 0.5438 | 0.155* | |
| C30 | 0.7912 (3) | 0.25943 (15) | 0.43133 (13) | 0.0401 (6) | |
| C31 | 1.0511 (4) | 0.1292 (2) | 0.38341 (18) | 0.0652 (9) | |
| H31A | 1.1010 | 0.1174 | 0.4252 | 0.098* | |
| H31B | 1.1179 | 0.1352 | 0.3520 | 0.098* | |
| H31C | 0.9862 | 0.0921 | 0.3693 | 0.098* | |
| C32 | 0.9711 (3) | 0.19554 (16) | 0.38903 (13) | 0.0428 (6) | |
| C33 | 0.9903 (3) | 0.25806 (17) | 0.36084 (13) | 0.0465 (7) | |
| C34 | 1.1001 (4) | 0.2773 (2) | 0.31590 (18) | 0.0712 (11) | |
| H34A | 1.0559 | 0.2796 | 0.2716 | 0.107* | |
| H34B | 1.1735 | 0.2423 | 0.3191 | 0.107* | |
| H34C | 1.1407 | 0.3222 | 0.3285 | 0.107* | |
| Cl1 | 0.92088 (15) | 0.46413 (5) | 0.25919 (6) | 0.0915 (3) | |
| O1 | 0.8578 (7) | 0.4008 (4) | 0.2450 (4) | 0.097 (2) | 0.682 (8) |
| O2 | 1.0542 (7) | 0.4605 (5) | 0.2262 (3) | 0.162 (3) | 0.682 (8) |
| O3 | 0.8404 (13) | 0.5178 (4) | 0.2230 (3) | 0.163 (4) | 0.682 (8) |
| O4 | 0.9423 (9) | 0.4804 (4) | 0.3232 (4) | 0.101 (2) | 0.682 (8) |
| O1A | 1.000 (2) | 0.4585 (9) | 0.3292 (8) | 0.101 (5) | 0.318 (8) |
| O2A | 0.7557 (16) | 0.4598 (8) | 0.2789 (10) | 0.169 (6) | 0.318 (8) |
| O3A | 0.9431 (15) | 0.5241 (8) | 0.2425 (8) | 0.114 (5) | 0.318 (8) |
| O4A | 0.933 (2) | 0.3994 (10) | 0.2418 (11) | 0.146 (7) | 0.318 (8) |

**B. Crystal Structure of 4b.**

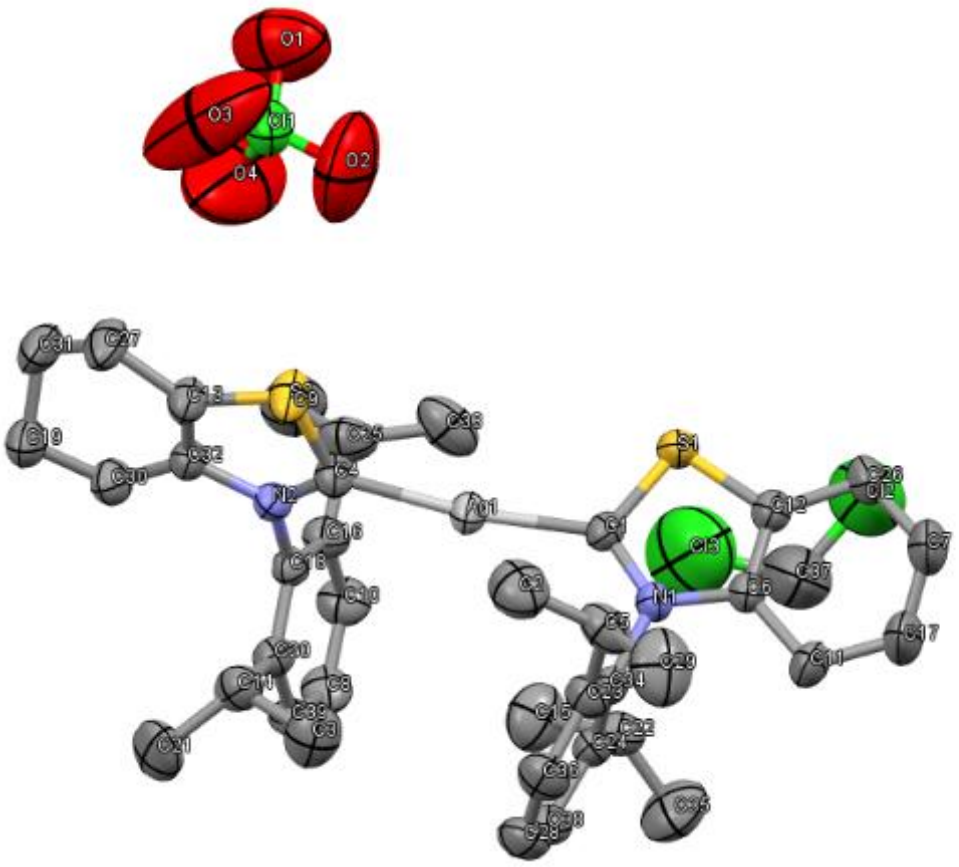

**Supplementary Figure S3.** Crystal structure of **4b** *(50% ellipsoids)*. (Crystallographic data has been deposited with the Cambridge Crystallographic Data Center as supplementary publication no. CCDC 2117722.)

**Supplementary Table S3.** *Crystal Data and Structure Refinement Summary for* ***4b****.*

*Crystal data*

| | |
|---|---|
| $C_{38}H_{50}AgN_2S_2 \cdot ClO_4 \cdot CH_2Cl_2$ | $F(000) = 1848$ |
| $M_r = 891.16$ | $D_x = 1.364$ Mg m$^{-3}$ |
| Monoclinic, $P2_1/c$ | Mo $K\alpha$ radiation, $\lambda = 0.71073$ Å |
| $a = 16.1022$ (11) Å | Cell parameters from 9883 reflections |
| $b = 15.8121$ (11) Å | $\theta = 2.4$–$27.4°$ |
| $c = 18.1191$ (13) Å | $\mu = 0.78$ mm$^{-1}$ |
| $\beta = 109.815$ (2)° | $T = 273$ K |
| $V = 4340.2$ (5) Å$^3$ | Block, colourless |
| $Z = 4$ | |

*Data collection*

| | |
|---|---|
| Bruker APEX-II CCD diffractometer | 5547 reflections with $I > 2\sigma(I)$ |
| $\phi$ and $\omega$ scans | $R_{int}$ = 0.092 |
| Absorption correction: multi-scan *SADABS2016*/2 (Bruker,2016/2) was used for absorption correction. wR2(int) was 0.0818 before and 0.0698 after correction. The Ratio of minimum to maximum transmission is 0.8785. The $\lambda/2$ correction factor is Not present. | $\theta_{max}$ = 25.0°, $\theta_{min}$ = 2.6° |
| $T_{min}$ = 0.655, $T_{max}$ = 0.746 | $h$ = -19→19 |
| 92075 measured reflections | $k$ = -18→18 |
| 7632 independent reflections | $l$ = -21→21 |

*Refinement*

| | |
|---|---|
| Refinement on $F^2$ | Primary atom site location: dual |
| Least-squares matrix: full | Hydrogen site location: inferred from neighbouring sites |
| $R[F^2 > 2\sigma(F^2)]$ = 0.056 | H-atom parameters constrained |
| $wR(F^2)$ = 0.163 | $w = 1/[\sigma^2(F_o^2) + (0.0716P)^2 + 11.9443P]$ where $P = (F_o^2 + 2F_c^2)/3$ |
| $S$ = 1.03 | $(\Delta/\sigma)_{max}$ = 0.001 |
| 7632 reflections | $\Delta\rangle_{max}$ = 1.49 e Å$^{-3}$ |
| 468 parameters | $\Delta\rangle_{min}$ = -1.07 e Å$^{-3}$ |
| 0 restraints | |

*Special details*

*Geometry*. All esds (except the esd in the dihedral angle between two l.s. planes) are estimated using the full covariance matrix. The cell esds are taken into account individually in the estimation of esds in distances, angles and torsion angles; correlations between esds in cell parameters are only used when they are defined by crystal symmetry. An approximate (isotropic) treatment of cell esds is used for estimating esds involving l.s. planes.

*Fractional atomic coordinates and isotropic or equivalent isotropic displacement*

*parameters (Å2) for (**4b**).*

| | $x$ | $y$ | $z$ | $U_{iso}*/U_{eq}$ |
|---|---|---|---|---|
| Ag1 | 0.60840 (2) | 0.65194 (3) | 0.34826 (2) | 0.04275 (15) |
| S1 | 0.74191 (9) | 0.79797 (8) | 0.45633 (9) | 0.0461 (3) |
| S2 | 0.38885 (9) | 0.63666 (11) | 0.28916 (9) | 0.0565 (4) |
| N1 | 0.7974 (3) | 0.6518 (3) | 0.4720 (2) | 0.0381 (9) |
| C39 | 0.6276 (4) | 0.5025 (4) | 0.1219 (3) | 0.0518 (14) |
| H39 | 0.651416 | 0.449376 | 0.119818 | 0.062* |
| N2 | 0.4634 (3) | 0.6047 (3) | 0.1940 (2) | 0.0378 (10) |
| C1 | 0.7257 (3) | 0.6953 (3) | 0.4291 (3) | 0.0398 (12) |
| C2 | 0.6340 (5) | 0.5115 (5) | 0.5366 (5) | 0.086 (2) |
| H2A | 0.604567 | 0.534648 | 0.485343 | 0.129* |
| H2B | 0.608671 | 0.534828 | 0.573031 | 0.129* |
| H2C | 0.627174 | 0.451168 | 0.534830 | 0.129* |
| C3 | 0.6060 (5) | 0.4104 (5) | 0.2676 (4) | 0.082 (2) |
| H3A | 0.657604 | 0.393772 | 0.256003 | 0.123* |
| H3B | 0.585966 | 0.363862 | 0.291142 | 0.123* |
| H3C | 0.620217 | 0.457395 | 0.303273 | 0.123* |
| C4 | 0.4837 (3) | 0.6243 (3) | 0.2695 (3) | 0.0414 (12) |
| C5 | 0.7320 (4) | 0.5337 (4) | 0.5628 (4) | 0.0600 (16) |
| H5 | 0.737516 | 0.595347 | 0.567433 | 0.072* |
| C6 | 0.8647 (3) | 0.6981 (3) | 0.5264 (3) | 0.0397 (12) |
| C7 | 0.9789 (5) | 0.8039 (5) | 0.6391 (5) | 0.092 (3) |
| H7A | 1.027028 | 0.844172 | 0.656538 | 0.110* |
| H7B | 0.960633 | 0.791464 | 0.683736 | 0.110* |
| C8 | 0.6565 (4) | 0.5711 (4) | 0.0908 (4) | 0.0572 (16) |
| H8 | 0.698800 | 0.563725 | 0.066890 | 0.069* |
| C9 | 0.4822 (6) | 0.7898 (5) | 0.0493 (5) | 0.105 (3) |
| H9A | 0.526556 | 0.797402 | 0.025354 | 0.158* |
| H9B | 0.455949 | 0.843392 | 0.052972 | 0.158* |
| H9C | 0.437608 | 0.751860 | 0.017947 | 0.158* |
| C10 | 0.6237 (4) | 0.6501 (4) | 0.0946 (4) | 0.0561 (15) |
| H10 | 0.645298 | 0.695877 | 0.074372 | 0.067* |

| | | | | |
|---|---|---|---|---|
| C11 | 0.9484 (4) | 0.6599 (4) | 0.5778 (4) | 0.0579 (16) |
| H11A | 0.973693 | 0.625122 | 0.546740 | 0.069* |
| H11B | 0.936870 | 0.624107 | 0.616736 | 0.069* |
| C12 | 0.8447 (3) | 0.7804 (3) | 0.5258 (3) | 0.0449 (13) |
| C13 | 0.3230 (4) | 0.6132 (4) | 0.1947 (3) | 0.0490 (14) |
| C14 | 0.5335 (4) | 0.4361 (4) | 0.1921 (4) | 0.0567 (16) |
| H14 | 0.481425 | 0.452667 | 0.205024 | 0.068* |
| C15 | 0.8150 (5) | 0.5996 (6) | 0.2720 (4) | 0.088 (2) |
| H15A | 0.814528 | 0.544922 | 0.248809 | 0.132* |
| H15B | 0.835840 | 0.641031 | 0.243657 | 0.132* |
| H15C | 0.756226 | 0.614000 | 0.269661 | 0.132* |
| C16 | 0.5593 (4) | 0.6634 (3) | 0.1277 (3) | 0.0447 (13) |
| C17 | 1.0122 (5) | 0.7287 (5) | 0.6179 (6) | 0.115 (4) |
| H17A | 1.054126 | 0.704848 | 0.665261 | 0.138* |
| H17B | 1.045034 | 0.743986 | 0.583853 | 0.138* |
| C18 | 0.5300 (3) | 0.5927 (3) | 0.1579 (3) | 0.0367 (11) |
| C19 | 0.2412 (4) | 0.5518 (5) | 0.0418 (4) | 0.0641 (18) |
| H19A | 0.215430 | 0.551826 | -0.015012 | 0.077* |
| H19B | 0.235760 | 0.495151 | 0.060102 | 0.077* |
| C20 | 0.5634 (3) | 0.5116 (3) | 0.1563 (3) | 0.0424 (12) |
| C21 | 0.5082 (5) | 0.3617 (4) | 0.1348 (5) | 0.087 (2) |
| H21A | 0.468162 | 0.380773 | 0.085238 | 0.130* |
| H21B | 0.480333 | 0.318611 | 0.155534 | 0.130* |
| H21C | 0.560370 | 0.339070 | 0.127788 | 0.130* |
| C22 | 0.8759 (4) | 0.5978 (4) | 0.3574 (4) | 0.0557 (15) |
| H22 | 0.875009 | 0.654360 | 0.379292 | 0.067* |
| C23 | 0.7725 (4) | 0.5055 (3) | 0.5037 (3) | 0.0502 (14) |
| C24 | 0.8410 (3) | 0.5364 (3) | 0.4040 (3) | 0.0438 (13) |
| C25 | 0.5246 (5) | 0.7526 (4) | 0.1314 (4) | 0.0640 (17) |
| H25 | 0.478971 | 0.749001 | 0.155896 | 0.077* |
| C26 | 0.9031 (4) | 0.8463 (4) | 0.5779 (4) | 0.0618 (17) |
| H26A | 0.925332 | 0.884165 | 0.546734 | 0.074* |
| H26B | 0.869518 | 0.879462 | 0.602935 | 0.074* |

| | | | | |
|---|---|---|---|---|
| C27 | 0.2229 (4) | 0.6114 (5) | 0.1639 (4) | 0.0650 (18) |
| H27A | 0.201873 | 0.560715 | 0.182056 | 0.078* |
| H27B | 0.199954 | 0.660123 | 0.183304 | 0.078* |
| C28 | 0.8128 (4) | 0.3925 (4) | 0.4330 (4) | 0.0645 (18) |
| H28 | 0.815314 | 0.334814 | 0.423783 | 0.077* |
| C29 | 0.7798 (6) | 0.4956 (6) | 0.6437 (4) | 0.101 (3) |
| H29A | 0.754318 | 0.517346 | 0.680706 | 0.151* |
| H29B | 0.841200 | 0.510389 | 0.660270 | 0.151* |
| H29C | 0.773792 | 0.435172 | 0.640984 | 0.151* |
| C30 | 0.3389 (4) | 0.5749 (4) | 0.0655 (3) | 0.0512 (14) |
| H30A | 0.371979 | 0.527505 | 0.055874 | 0.061* |
| H30B | 0.346311 | 0.622624 | 0.034671 | 0.061* |
| C31 | 0.1910 (4) | 0.6128 (5) | 0.0751 (4) | 0.0714 (19) |
| H31A | 0.128796 | 0.598395 | 0.055182 | 0.086* |
| H31B | 0.197470 | 0.669531 | 0.057475 | 0.086* |
| C32 | 0.3724 (3) | 0.5972 (3) | 0.1508 (3) | 0.0384 (12) |
| C33 | 0.5985 (6) | 0.8097 (4) | 0.1828 (5) | 0.083 (2) |
| H33A | 0.620359 | 0.787464 | 0.235158 | 0.125* |
| H33B | 0.575954 | 0.865706 | 0.183762 | 0.125* |
| H33C | 0.645673 | 0.811663 | 0.161593 | 0.125* |
| C34 | 0.8045 (3) | 0.5612 (3) | 0.4596 (3) | 0.0394 (12) |
| C35 | 0.9709 (5) | 0.5790 (6) | 0.3636 (6) | 0.096 (3) |
| H35A | 0.973184 | 0.525978 | 0.338573 | 0.144* |
| H35B | 1.007322 | 0.576080 | 0.417846 | 0.144* |
| H35C | 0.992079 | 0.623208 | 0.338208 | 0.144* |
| C36 | 0.7788 (4) | 0.4196 (4) | 0.4881 (4) | 0.0624 (17) |
| H36 | 0.759075 | 0.379784 | 0.516226 | 0.075* |
| C38 | 0.8435 (4) | 0.4492 (4) | 0.3908 (4) | 0.0579 (16) |
| H38 | 0.866251 | 0.429584 | 0.353167 | 0.069* |
| Cl1 | 0.19968 (12) | 0.78917 (13) | 0.34499 (12) | 0.0784 (5) |
| O1 | 0.1727 (7) | 0.8727 (5) | 0.3292 (7) | 0.179 (4) |
| O2 | 0.2774 (6) | 0.7827 (8) | 0.4039 (6) | 0.217 (5) |
| O3 | 0.1347 (6) | 0.7439 (7) | 0.3483 (11) | 0.309 (10) |

| | | | | |
|---|---|---|---|---|
| O4 | 0.2151 (10) | 0.7610 (12) | 0.2842 (7) | 0.348 (12) |
| Cl2 | 0.9767 (3) | 0.9520 (3) | 0.3954 (4) | 0.274 (3) |
| Cl3 | 0.8666 (3) | 0.8310 (3) | 0.3013 (4) | 0.208 (2) |
| C37 | 0.9684 (9) | 0.8663 (9) | 0.3467 (11) | 0.209 (8) |
| H37A | 1.001103 | 0.822548 | 0.382324 | 0.251* |
| H37B | 0.996661 | 0.874695 | 0.307788 | 0.251* |

## C. Crystal Structure of 4c.

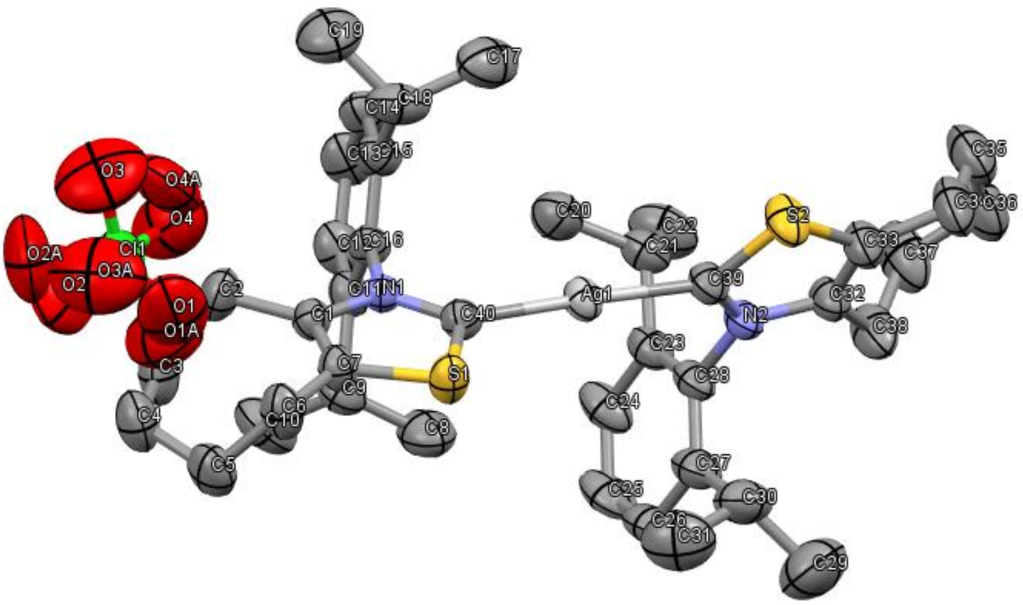


**Supplementary Figure S4.** Crystal structure of **4c** *(50% ellipsoids)*. (Crystallographic data has been deposited with the Cambridge Crystallographic Data Center as supplementary publication no. CCDC 2117721.)

**Supplementary Table S4.** *Crystal Data and Structure Refinement Summary for* ***4c****.*

*Crystal data*

| | |
|---|---|
| $C_{40}H_{54}AgN_2S_2{\cdot}ClO_4$ | $F(000) = 1744$ |
| $M_r = 834.29$ | $D_x = 1.310$ Mg m$^{-3}$ |
| Monoclinic, $P2_1/c$ | Mo $K\alpha$ radiation, $\lambda = 0.71073$ Å |
| $a = 15.0604$ (3) Å | Cell parameters from 9977 reflections |
| $b = 14.2190$ (3) Å | $\theta = 2.5–26.9°$ |
| $c = 22.6671$ (4) Å | $\mu = 0.68$ mm$^{-1}$ |
| $\beta = 119.366$ (1)° | $T = 293$ K |
| $V = 4230.30$ (15) Å$^3$ | Block |
| $Z = 4$ | |

*Data collection*

| | |
|---|---|
| Bruker APEX-II CCD diffractometer | $R_{int}$ = 0.033 |
| ϕ and ω scans | $\theta_{max}$ = 25.0°, $\theta_{min}$ = 2.7° |
| 37170 measured reflections | $h$ = -17→17 |
| 7449 independent reflections | $k$ = -16→16 |
| 6233 reflections with $I > 2\sigma(I)$ | $l$ = -26→26 |

## *Refinement*

| | |
|---|---|
| Refinement on $F^2$ | Primary atom site location: dual |
| Least-squares matrix: full | Hydrogen site location: inferred from neighbouring sites |
| $R[F^2 > 2\sigma(F^2)]$ = 0.031 | H-atom parameters constrained |
| $wR(F^2)$ = 0.095 | $w = 1/[\sigma^2(F_o^2) + (0.0519P)^2 + 1.0032P]$ where $P = (F_o^2 + 2F_c^2)/3$ |
| $S$ = 1.09 | $(\Delta/\sigma)_{max}$ = 0.001 |
| 7449 reflections | $\Delta\rangle_{max}$ = 0.38 e Å$^{-3}$ |
| 496 parameters | $\Delta\rangle_{min}$ = -0.47 e Å$^{-3}$ |
| 183 restraints | |

## *Special details*

| |
|---|
| *Geometry*. All esds (except the esd in the dihedral angle between two l.s. planes) are estimated using the full covariance matrix. The cell esds are taken into account individually in the estimation of esds in distances, angles and torsion angles; correlations between esds in cell parameters are only used when they are defined by crystal symmetry. An approximate (isotropic) treatment of cell esds is used for estimating esds involving l.s. planes. |

## *Fractional atomic coordinates and isotropic or equivalent isotropic displacement parameters (Å2) for (**4c**).*

| | $x$ | $y$ | $z$ | $U_{iso}$*/$U_{eq}$ | Occ. (<1) |
|---|---|---|---|---|---|
| Ag1 | 0.19530 (2) | -0.32824 (2) | -0.08133 (2) | 0.04437 (9) | |
| S1 | 0.13268 (6) | -0.33726 (5) | -0.24327 (3) | 0.05104 (18) | |
| S2 | 0.24990 (6) | -0.48636 (5) | 0.04232 (4) | 0.0597 (2) | |
| N1 | 0.05363 (15) | -0.21110 (14) | -0.21087 (10) | 0.0376 (4) | |

| | | | | |
|---|---|---|---|---|
| N2 | 0.35670 (16) | -0.34520 (15) | 0.06967 (10) | 0.0439 (5) |
| C28 | 0.4010 (2) | -0.25691 (19) | 0.06398 (12) | 0.0461 (6) |
| C16 | 0.0304 (2) | -0.14597 (17) | -0.17055 (13) | 0.0420 (6) |
| C1 | 0.01517 (19) | -0.19821 (18) | -0.28073 (12) | 0.0417 (6) |
| C40 | 0.11811 (19) | -0.28292 (17) | -0.18178 (12) | 0.0417 (6) |
| C39 | 0.2758 (2) | -0.38259 (18) | 0.01629 (13) | 0.0457 (6) |
| C11 | 0.0888 (2) | -0.06466 (18) | -0.14774 (13) | 0.0476 (6) |
| C7 | 0.0524 (2) | -0.26186 (17) | -0.30646 (12) | 0.0446 (6) |
| C23 | 0.3694 (2) | -0.17441 (19) | 0.08037 (14) | 0.0502 (7) |
| C6 | 0.0343 (3) | -0.2680 (2) | -0.37745 (14) | 0.0589 (8) |
| H6A | -0.0365 | -0.2842 | -0.4074 | 0.071* |
| H6B | 0.0759 | -0.3181 | -0.3800 | 0.071* |
| C25 | 0.4906 (3) | -0.0935 (3) | 0.05912 (15) | 0.0706 (10) |
| H25 | 0.5220 | -0.0377 | 0.0580 | 0.085* |
| C15 | -0.0484 (2) | -0.16768 (19) | -0.15738 (15) | 0.0523 (7) |
| C27 | 0.4759 (2) | -0.2614 (2) | 0.04469 (15) | 0.0587 (8) |
| C24 | 0.4151 (2) | -0.0911 (2) | 0.07684 (14) | 0.0628 (8) |
| H24 | 0.3949 | -0.0340 | 0.0864 | 0.075* |
| C12 | 0.0669 (2) | -0.0034 (2) | -0.10852 (15) | 0.0588 (7) |
| H12 | 0.1042 | 0.0517 | -0.0921 | 0.071* |
| C5 | 0.0586 (3) | -0.1767 (2) | -0.40200 (17) | 0.0692 (9) |
| H5A | 0.1251 | -0.1548 | -0.3677 | 0.083* |
| H5B | 0.0626 | -0.1899 | -0.4427 | 0.083* |
| C26 | 0.5201 (3) | -0.1756 (3) | 0.04323 (18) | 0.0723 (10) |
| H26 | 0.5709 | -0.1747 | 0.0311 | 0.087* |
| C21 | 0.2838 (2) | -0.1714 (2) | 0.09782 (15) | 0.0563 (7) |
| H21 | 0.2716 | -0.2358 | 0.1075 | 0.068* |
| C18 | -0.1124 (2) | -0.2557 (2) | -0.18382 (18) | 0.0682 (9) |
| H18 | -0.0919 | -0.2880 | -0.2134 | 0.082* |
| C13 | -0.0095 (3) | -0.0240 (2) | -0.09404 (16) | 0.0645 (8) |
| H13 | -0.0226 | 0.0171 | -0.0673 | 0.077* |
| C32 | 0.3979 (2) | -0.3948 (2) | 0.13053 (13) | 0.0524 (7) |
| C14 | -0.0666 (2) | -0.1039 (2) | -0.11826 (16) | 0.0625 (8) |

| | | | | |
|---|---|---|---|---|
| H14 | -0.1186 | -0.1157 | -0.1083 | 0.075* |
| C9 | 0.1754 (2) | -0.0432 (2) | -0.16201 (14) | 0.0539 (7) |
| H9 | 0.1647 | -0.0811 | -0.2011 | 0.065* |
| C2 | -0.0578 (2) | -0.1207 (2) | -0.31965 (15) | 0.0621 (8) |
| H2A | -0.0697 | -0.0830 | -0.2886 | 0.074* |
| H2B | -0.1224 | -0.1484 | -0.3522 | 0.074* |
| C22 | 0.3105 (3) | -0.1110 (3) | 0.16037 (18) | 0.0903 (12) |
| H22A | 0.3750 | -0.1307 | 0.1971 | 0.136* |
| H22B | 0.2587 | -0.1182 | 0.1731 | 0.136* |
| H22C | 0.3145 | -0.0461 | 0.1501 | 0.136* |
| C38 | 0.4841 (3) | -0.3564 (3) | 0.19496 (16) | 0.0760 (10) |
| H38A | 0.5051 | -0.2962 | 0.1857 | 0.091* |
| H38B | 0.5417 | -0.3989 | 0.2112 | 0.091* |
| C33 | 0.3478 (2) | -0.4754 (2) | 0.12416 (15) | 0.0574 (7) |
| C20 | 0.1865 (3) | -0.1361 (3) | 0.03708 (17) | 0.0711 (9) |
| H20A | 0.1965 | -0.0727 | 0.0269 | 0.107* |
| H20B | 0.1318 | -0.1377 | 0.0473 | 0.107* |
| H20C | 0.1700 | -0.1756 | -0.0013 | 0.107* |
| C4 | -0.0179 (3) | -0.0990 (2) | -0.41718 (17) | 0.0750 (10) |
| H4A | -0.0851 | -0.1232 | -0.4482 | 0.090* |
| H4B | -0.0035 | -0.0489 | -0.4403 | 0.090* |
| C17 | -0.0931 (3) | -0.3221 (2) | -0.1263 (2) | 0.0896 (13) |
| H17A | -0.0214 | -0.3342 | -0.0996 | 0.134* |
| H17B | -0.1286 | -0.3802 | -0.1445 | 0.134* |
| H17C | -0.1173 | -0.2939 | -0.0983 | 0.134* |
| C30 | 0.5073 (3) | -0.3526 (3) | 0.0254 (2) | 0.0756 (10) |
| H30 | 0.4693 | -0.4040 | 0.0317 | 0.091* |
| C3 | -0.0208 (3) | -0.0571 (2) | -0.35682 (17) | 0.0772 (10) |
| H3A | -0.0643 | -0.0020 | -0.3720 | 0.093* |
| H3B | 0.0474 | -0.0359 | -0.3247 | 0.093* |
| C34 | 0.3690 (3) | -0.5454 (2) | 0.17960 (18) | 0.0802 (11) |
| H34A | 0.4369 | -0.5709 | 0.1966 | 0.096* |
| H34B | 0.3209 | -0.5970 | 0.1610 | 0.096* |

| | | | | | |
|---|---|---|---|---|---|
| C35 | 0.3608 (3) | -0.5015 (3) | 0.23766 (18) | 0.0931 (13) | |
| H35A | 0.3589 | -0.5516 | 0.2661 | 0.112* | |
| H35B | 0.2966 | -0.4679 | 0.2192 | 0.112* | |
| C31 | 0.4797 (4) | -0.3512 (3) | -0.0490 (2) | 0.1043 (14) | |
| H31A | 0.4073 | -0.3438 | -0.0768 | 0.156* | |
| H31B | 0.5004 | -0.4093 | -0.0602 | 0.156* | |
| H31C | 0.5138 | -0.2997 | -0.0568 | 0.156* | |
| C19 | -0.2251 (3) | -0.2332 (3) | -0.2254 (2) | 0.0996 (13) | |
| H19A | -0.2473 | -0.2021 | -0.1974 | 0.149* | |
| H19B | -0.2628 | -0.2904 | -0.2428 | 0.149* | |
| H19C | -0.2363 | -0.1928 | -0.2624 | 0.149* | |
| C8 | 0.2764 (2) | -0.0722 (3) | -0.10254 (18) | 0.0818 (10) | |
| H8A | 0.2905 | -0.0340 | -0.0640 | 0.123* | |
| H8B | 0.3295 | -0.0639 | -0.1139 | 0.123* | |
| H8C | 0.2734 | -0.1371 | -0.0920 | 0.123* | |
| C10 | 0.1778 (3) | 0.0599 (2) | -0.1799 (2) | 0.0888 (12) | |
| H10A | 0.1125 | 0.0776 | -0.2166 | 0.133* | |
| H10B | 0.2289 | 0.0686 | -0.1931 | 0.133* | |
| H10C | 0.1937 | 0.0985 | -0.1412 | 0.133* | |
| C36 | 0.4463 (3) | -0.4341 (3) | 0.28187 (18) | 0.0963 (13) | |
| H36A | 0.4386 | -0.4181 | 0.3207 | 0.116* | |
| H36B | 0.5103 | -0.4676 | 0.2987 | 0.116* | |
| C29 | 0.6211 (3) | -0.3725 (4) | 0.0704 (3) | 0.1175 (16) | |
| H29A | 0.6600 | -0.3247 | 0.0633 | 0.176* | |
| H29B | 0.6372 | -0.4328 | 0.0591 | 0.176* | |
| H29C | 0.6375 | -0.3724 | 0.1170 | 0.176* | |
| C37 | 0.4546 (4) | -0.3437 (3) | 0.25013 (18) | 0.0951 (14) | |
| H37A | 0.5049 | -0.3038 | 0.2854 | 0.114* | |
| H37B | 0.3896 | -0.3113 | 0.2308 | 0.114* | |
| Cl1 | -0.27144 (7) | -0.31235 (6) | -0.54220 (5) | 0.0736 (2) | |
| O1 | -0.2018 (9) | -0.3745 (6) | -0.5399 (6) | 0.150 (4) | 0.578 (7) |
| O4 | -0.2415 (5) | -0.2929 (7) | -0.4705 (3) | 0.133 (3) | 0.578 (7) |
| O2 | -0.2561 (8) | -0.2225 (5) | -0.5496 (6) | 0.162 (4) | 0.578 (7) |

| | | | | | |
|---|---|---|---|---|---|
| O3 | -0.3708 (6) | -0.3454 (6) | -0.5661 (5) | 0.156 (4) | 0.578 (7) |
| O1A | -0.1660 (8) | -0.3237 (11) | -0.5200 (6) | 0.140 (5) | 0.422 (7) |
| O4A | -0.3087 (11) | -0.3587 (8) | -0.5100 (8) | 0.147 (5) | 0.422 (7) |
| O2A | -0.3191 (11) | -0.2332 (9) | -0.5854 (6) | 0.162 (5) | 0.422 (7) |
| O3A | -0.2993 (9) | -0.3690 (9) | -0.6062 (6) | 0.166 (4) | 0.422 (7) |

**D. Crystal Structure of 5a.**

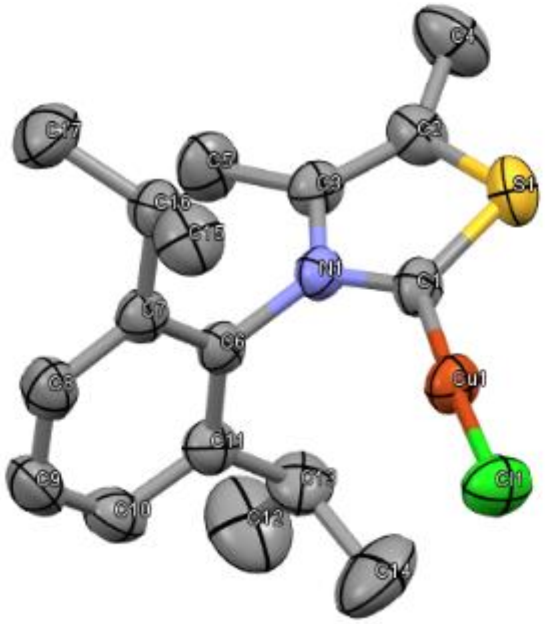


**Supplementary Figure S5.** Crystal structure of **5a** *(50% ellipsoids)*. (Crystallographic data has been deposited with the Cambridge Crystallographic Data Center as supplementary publication no. CCDC 2117739.)

**Supplementary Table S5.** *Crystal Data and Structure Refinement Summary for **5a**.*

*Crystal data*

| | |
|---|---|
| $C_{17}H_{23}ClCuNS$ | $F(000) = 776$ |
| $M_r = 372.41$ | $D_x = 1.345$ Mg m$^{-3}$ |
| Monoclinic, $P2_1/c$ | Mo $K\alpha$ radiation, $\lambda = 0.71073$ Å |
| $a = 8.6707$ (4) Å | Cell parameters from 9938 reflections |
| $b = 12.4816$ (6) Å | $\theta = 2.9$–$28.3°$ |
| $c = 17.3671$ (7) Å | $\mu = 1.44$ mm$^{-1}$ |
| $\beta = 101.887$ (2)° | $T = 273$ K |
| $V = 1839.23$ (14) Å$^3$ | Block |
| $Z = 4$ | 0.13 × 0.12 × 0.11 mm |

*Data collection*

| | |
|---|---|
| Bruker APEX-II CCD | $R_{int} = 0.023$ |

| | |
|---|---|
| diffractometer | |
| ϕ and ω scans | $\theta_{max}$ = 25.0°, $\theta_{min}$ = 2.4° |
| 15419 measured reflections | $h$ = -10→10 |
| 3235 independent reflections | $k$ = -12→14 |
| 2947 reflections with $I > 2\sigma(I)$ | $l$ = -20→20 |

*Refinement*

| | |
|---|---|
| Refinement on $F^2$ | Primary atom site location: dual |
| Least-squares matrix: full | Hydrogen site location: inferred from neighbouring sites |
| $R[F^2 > 2\sigma(F^2)]$ = 0.027 | H-atom parameters constrained |
| $wR(F^2)$ = 0.096 | $w = 1/[\sigma^2(F_o^2) + (0.0562P)^2 + 0.4615P]$ where $P = (F_o^2 + 2F_c^2)/3$ |
| $S$ = 1.16 | $(\Delta/\sigma)_{max}$ = 0.001 |
| 3235 reflections | $\Delta\rangle_{max}$ = 0.51 e Å$^{-3}$ |
| 196 parameters | $\Delta\rangle_{min}$ = -0.64 e Å$^{-3}$ |
| 0 restraints | |

*Special details*

| |
|---|
| *Geometry*. All esds (except the esd in the dihedral angle between two l.s. planes) are estimated using the full covariance matrix. The cell esds are taken into account individually in the estimation of esds in distances, angles and torsion angles; correlations between esds in cell parameters are only used when they are defined by crystal symmetry. An approximate (isotropic) treatment of cell esds is used for estimating esds involving l.s. planes. |

*Fractional atomic coordinates and isotropic or equivalent isotropic displacement parameters (Å$^2$) for (**5a**)*

| | $x$ | $y$ | $z$ | $U_{iso}$*/$U_{eq}$ |
|---|---|---|---|---|
| Cu1 | 0.40595 (3) | 0.22809 (2) | 0.32391 (2) | 0.04679 (13) |
| Cl1 | 0.26388 (8) | 0.24864 (6) | 0.21177 (4) | 0.06084 (18) |
| S1 | 0.45168 (6) | 0.15007 (5) | 0.49917 (4) | 0.05301 (17) |
| N1 | 0.6689 (2) | 0.24532 (13) | 0.45780 (10) | 0.0371 (4) |
| C1 | 0.5234 (2) | 0.21090 (16) | 0.42631 (13) | 0.0415 (4) |

| | | | | |
|---|---|---|---|---|
| C2 | 0.6186 (3) | 0.17296 (18) | 0.57034 (13) | 0.0483 (5) |
| C3 | 0.7246 (3) | 0.22598 (16) | 0.53826 (13) | 0.0421 (5) |
| C4 | 0.6306 (4) | 0.1353 (3) | 0.65336 (17) | 0.0759 (8) |
| H4A | 0.6477 | 0.0593 | 0.6559 | 0.114* |
| H4B | 0.5345 | 0.1518 | 0.6702 | 0.114* |
| H4C | 0.7171 | 0.1708 | 0.6871 | 0.114* |
| C5 | 0.8839 (3) | 0.2650 (2) | 0.57795 (15) | 0.0632 (7) |
| H5A | 0.8895 | 0.3412 | 0.5714 | 0.095* |
| H5B | 0.9628 | 0.2308 | 0.5550 | 0.095* |
| H5C | 0.9019 | 0.2481 | 0.6330 | 0.095* |
| C6 | 0.7676 (2) | 0.29624 (17) | 0.40961 (11) | 0.0378 (4) |
| C7 | 0.7605 (2) | 0.40689 (17) | 0.39979 (11) | 0.0421 (4) |
| C8 | 0.8580 (3) | 0.4522 (2) | 0.35432 (14) | 0.0545 (6) |
| H8 | 0.8550 | 0.5257 | 0.3456 | 0.065* |
| C9 | 0.9585 (3) | 0.3896 (2) | 0.32210 (14) | 0.0602 (6) |
| H9 | 1.0245 | 0.4215 | 0.2928 | 0.072* |
| C10 | 0.9630 (3) | 0.2806 (2) | 0.33264 (15) | 0.0562 (6) |
| H10 | 1.0314 | 0.2397 | 0.3099 | 0.067* |
| C11 | 0.8670 (2) | 0.23019 (17) | 0.37670 (13) | 0.0444 (5) |
| C12 | 1.0330 (4) | 0.0623 (3) | 0.4036 (3) | 0.1012 (12) |
| H12A | 1.0916 | 0.0947 | 0.4508 | 0.152* |
| H12B | 1.0852 | 0.0760 | 0.3610 | 0.152* |
| H12C | 1.0263 | -0.0136 | 0.4113 | 0.152* |
| C13 | 0.8675 (3) | 0.1098 (2) | 0.38430 (16) | 0.0595 (6) |
| H13 | 0.8151 | 0.0915 | 0.4274 | 0.071* |
| C14 | 0.7715 (5) | 0.0607 (3) | 0.3089 (2) | 0.0983 (12) |
| H14A | 0.8187 | 0.0792 | 0.2653 | 0.147* |
| H14B | 0.6657 | 0.0878 | 0.2998 | 0.147* |
| H14C | 0.7699 | -0.0159 | 0.3142 | 0.147* |
| C15 | 0.5158 (3) | 0.5176 (2) | 0.37200 (17) | 0.0659 (7) |
| H15A | 0.4566 | 0.4577 | 0.3466 | 0.099* |
| H15B | 0.5574 | 0.5580 | 0.3339 | 0.099* |
| H15C | 0.4481 | 0.5625 | 0.3953 | 0.099* |

| | | | | |
|---|---|---|---|---|
| C16 | 0.6506 (3) | 0.47715 (17) | 0.43558 (13) | 0.0484 (5) |
| H16 | 0.6058 | 0.4332 | 0.4723 | 0.058* |
| C17 | 0.7399 (4) | 0.5700 (2) | 0.48173 (18) | 0.0705 (7) |
| H17A | 0.7786 | 0.6170 | 0.4462 | 0.106* |
| H17B | 0.8269 | 0.5426 | 0.5200 | 0.106* |
| H17C | 0.6703 | 0.6090 | 0.5080 | 0.106* |

## E. Crystal Structure of 9ad.

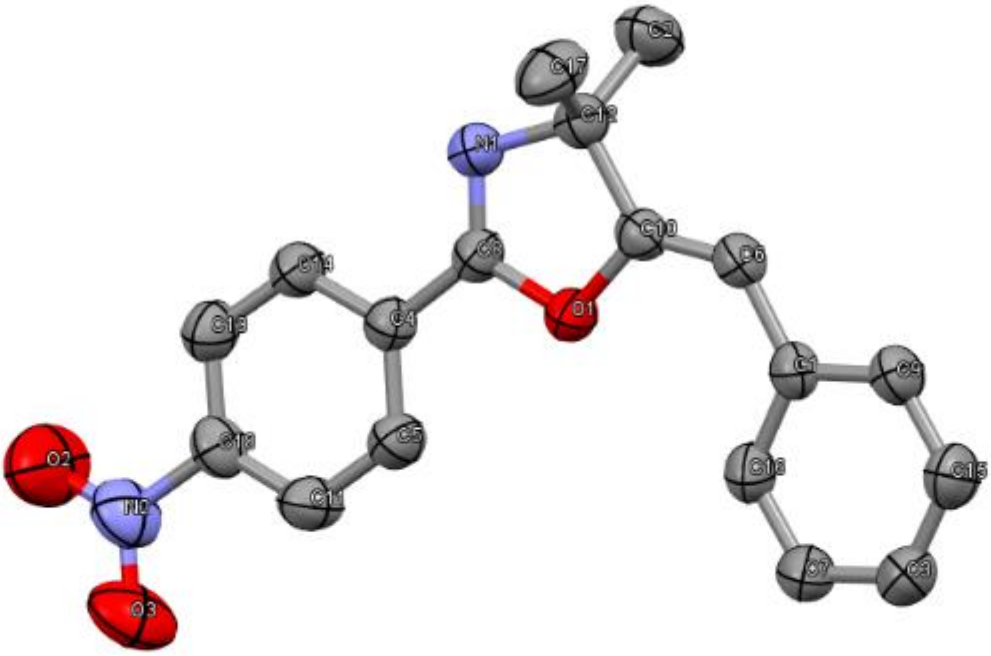


**Supplementary Figure S6.** Crystal structure of **9ad** *(50% ellipsoids)*. (Crystallographic data has been deposited with the Cambridge Crystallographic Data Center as supplementary publication no. CCDC 2125052.)

**Supplementary Table S6.** *Crystal Data and Structure Refinement Summary for* ***9ad****.*

*Crystal data*

| | |
|---|---|
| $C_{18}H_{16}N_2O_3$ | *F*(000) = 648 |
| $M_r$ = 308.33 | $D_x$ = 1.252 Mg m$^{-3}$ |
| Monoclinic, $P2_1/n$ | Mo *K*α radiation, λ = 0.71073 Å |
| *a* = 6.9122 (5) Å | Cell parameters from 9975 reflections |
| *b* = 24.975 (2) Å | θ = 2.7–27.4° |
| *c* = 9.6235 (8) Å | μ = 0.09 mm$^{-1}$ |
| β = 100.183 (2)° | *T* = 301 K |
| *V* = 1635.2 (2) Å$^3$ | Block, colourless |
| *Z* = 4 | |

*Data collection*

| | |
|---|---|
| Bruker APEX-II CCD diffractometer | 2697 reflections with $I > 2\sigma(I)$ |
| $\phi$ and $\omega$ scans | $R_{int}$ = 0.050 |
| Absorption correction: multi-scan *SADABS2016*/2 (Bruker,2016/2) was used for absorption correction. wR2(int) was 0.0620 before and 0.0571 after correction. The Ratio of minimum to maximum transmission is 0.9127. The $\lambda/2$ correction factor is Not present. | $\theta_{max}$ = 27.5°, $\theta_{min}$ = 2.7° |
| $T_{min}$ = 0.681, $T_{max}$ = 0.746 | $h = -8\rightarrow 8$ |
| 40795 measured reflections | $k = -32\rightarrow 32$ |
| 3736 independent reflections | $l = -12\rightarrow 12$ |

*Refinement*

| | |
|---|---|
| Refinement on $F^2$ | Primary atom site location: dual |
| Least-squares matrix: full | Hydrogen site location: inferred from neighbouring sites |
| $R[F^2 > 2\sigma(F^2)]$ = 0.052 | H-atom parameters constrained |
| $wR(F^2)$ = 0.131 | $w = 1/[\sigma^2(F_o^2) + (0.0492P)^2 + 0.5083P]$ where $P = (F_o^2 + 2F_c^2)/3$ |
| $S$ = 1.03 | $(\Delta/\sigma)_{max}$ < 0.001 |
| 3736 reflections | $\Delta\rangle_{max}$ = 0.15 e Å$^{-3}$ |
| 210 parameters | $\Delta\rangle_{min}$ = -0.23 e Å$^{-3}$ |
| 0 restraints | |

*Special details*

| |
|---|
| *Geometry*. All esds (except the esd in the dihedral angle between two l.s. planes) are estimated using the full covariance matrix. The cell esds are taken into account individually in the estimation of esds in distances, angles and torsion angles; correlations between esds in cell parameters are only used when they are defined by crystal symmetry. An approximate (isotropic) treatment of cell esds is used for estimating esds involving l.s. planes. |

*Fractional atomic coordinates and isotropic or equivalent isotropic displacement parameters (Å$^2$) for (**9ad**)*

| | $x$ | $y$ | $z$ | $U_{iso}$*/$U_{eq}$ |
|---|---|---|---|---|

| | | | | |
|---|---|---|---|---|
| O1 | 0.52989 (17) | 0.36856 (4) | 0.56627 (12) | 0.0492 (3) |
| O2 | 1.2616 (4) | 0.41133 (11) | 0.1270 (3) | 0.1617 (12) |
| O3 | 1.0680 (2) | 0.35151 (8) | 0.03305 (17) | 0.0907 (5) |
| N1 | 0.7028 (2) | 0.44080 (6) | 0.65919 (15) | 0.0519 (4) |
| N2 | 1.1208 (3) | 0.38357 (8) | 0.1258 (2) | 0.0731 (5) |
| C1 | 0.1747 (2) | 0.31264 (6) | 0.64135 (16) | 0.0443 (4) |
| C2 | 0.6412 (3) | 0.42319 (9) | 0.8986 (2) | 0.0715 (6) |
| H2A | 0.725552 | 0.392435 | 0.904129 | 0.107* |
| H2B | 0.540373 | 0.416997 | 0.953659 | 0.107* |
| H2C | 0.717035 | 0.453979 | 0.934525 | 0.107* |
| C3 | -0.0538 (3) | 0.22722 (8) | 0.5165 (2) | 0.0624 (5) |
| H3 | -0.130494 | 0.199019 | 0.474407 | 0.075* |
| C4 | 0.7978 (2) | 0.39668 (6) | 0.45467 (17) | 0.0430 (4) |
| C5 | 0.7450 (3) | 0.35912 (7) | 0.34894 (19) | 0.0545 (4) |
| H5 | 0.636788 | 0.337122 | 0.350146 | 0.065* |
| C6 | 0.2836 (2) | 0.35855 (6) | 0.70971 (17) | 0.0462 (4) |
| H6 | 0.236981 | 0.371985 | 0.787676 | 0.055* |
| C7 | 0.1080 (3) | 0.24372 (7) | 0.4639 (2) | 0.0618 (5) |
| H7 | 0.141822 | 0.226307 | 0.386193 | 0.074* |
| C8 | 0.6814 (2) | 0.40451 (6) | 0.56643 (17) | 0.0443 (4) |
| C9 | 0.0112 (3) | 0.29484 (8) | 0.69373 (19) | 0.0575 (5) |
| H9 | -0.022851 | 0.311657 | 0.772189 | 0.069* |
| C10 | 0.4383 (2) | 0.38402 (6) | 0.67799 (16) | 0.0438 (4) |
| C11 | 0.8529 (3) | 0.35425 (8) | 0.2417 (2) | 0.0576 (5) |
| H11 | 0.819390 | 0.328899 | 0.170695 | 0.069* |
| C12 | 0.5471 (2) | 0.43296 (7) | 0.74518 (18) | 0.0488 (4) |
| C13 | 1.0679 (3) | 0.42480 (7) | 0.3465 (2) | 0.0569 (5) |
| H13 | 1.176318 | 0.446656 | 0.344759 | 0.068* |
| C14 | 0.9607 (2) | 0.42877 (7) | 0.45366 (19) | 0.0510 (4) |
| H14 | 0.998085 | 0.453285 | 0.526171 | 0.061* |
| C15 | -0.1014 (3) | 0.25286 (8) | 0.6321 (2) | 0.0672 (5) |
| H15 | -0.210399 | 0.241784 | 0.668967 | 0.081* |
| C16 | 0.2212 (3) | 0.28581 (7) | 0.52478 (19) | 0.0545 (4) |

| | | | | |
|---|---|---|---|---|
| H16 | 0.330338 | 0.296450 | 0.487429 | 0.065* |
| C17 | 0.4151 (3) | 0.48229 (7) | 0.7302 (3) | 0.0719 (6) |
| H17A | 0.490414 | 0.512957 | 0.767633 | 0.108* |
| H17B | 0.309291 | 0.476800 | 0.781191 | 0.108* |
| H17C | 0.362403 | 0.488162 | 0.632188 | 0.108* |
| C18 | 1.0102 (2) | 0.38763 (7) | 0.24223 (19) | 0.0520 (4) |

## Supplementary Note 2: $^{1}$H and $^{13}$C Spectra

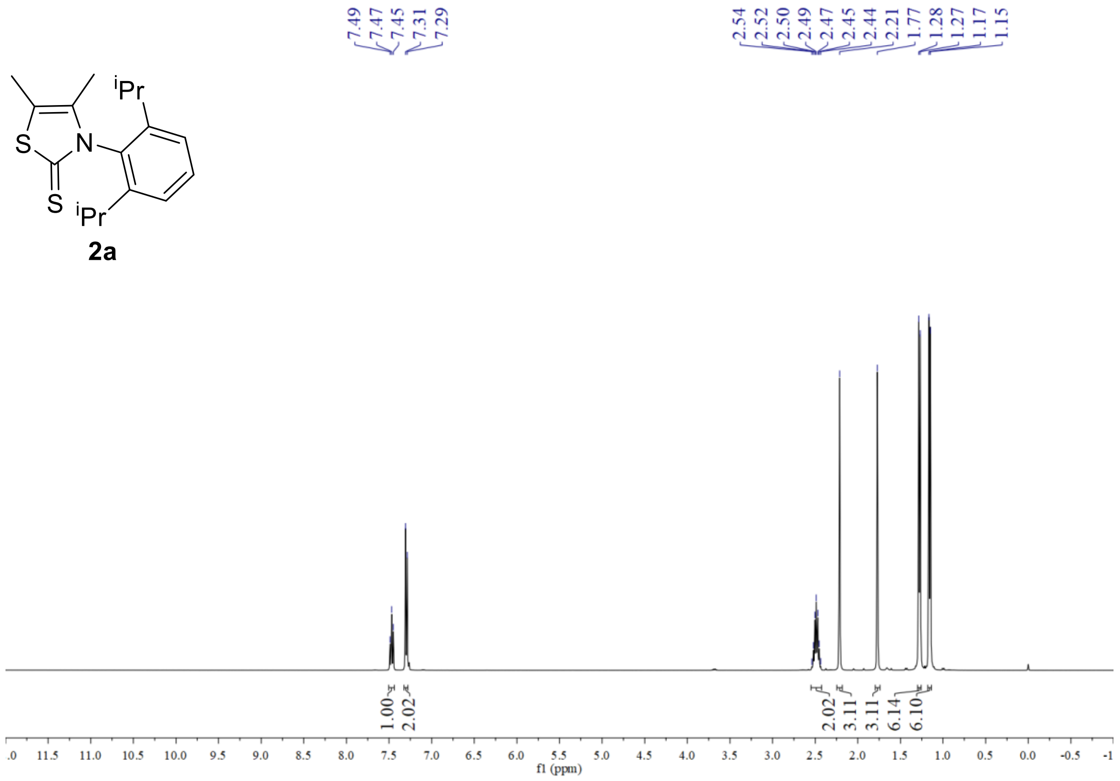


**Supplementary Figure S7.** $^{1}$H NMR (400 MHz, $CDCl_3$) Spectrum of Compound **2a**

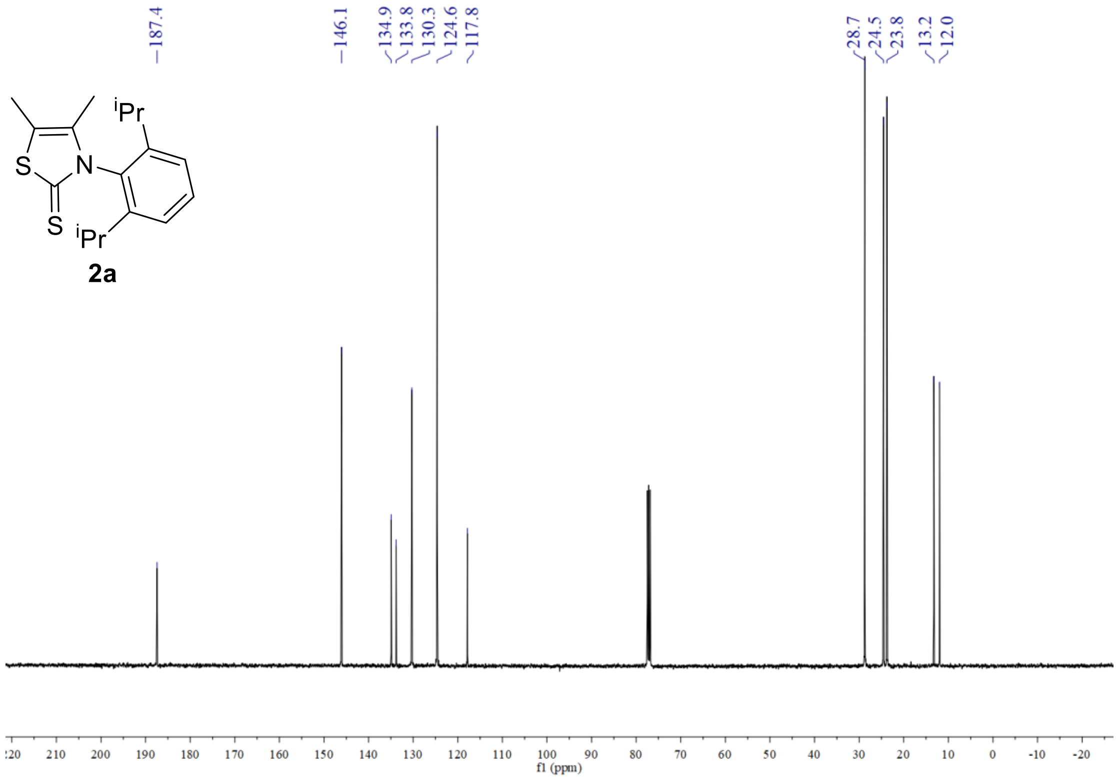


**Supplementary Figure S8.** $^{13}$C NMR (100 MHz, $CDCl_3$) Spectrum of Compound **2a**

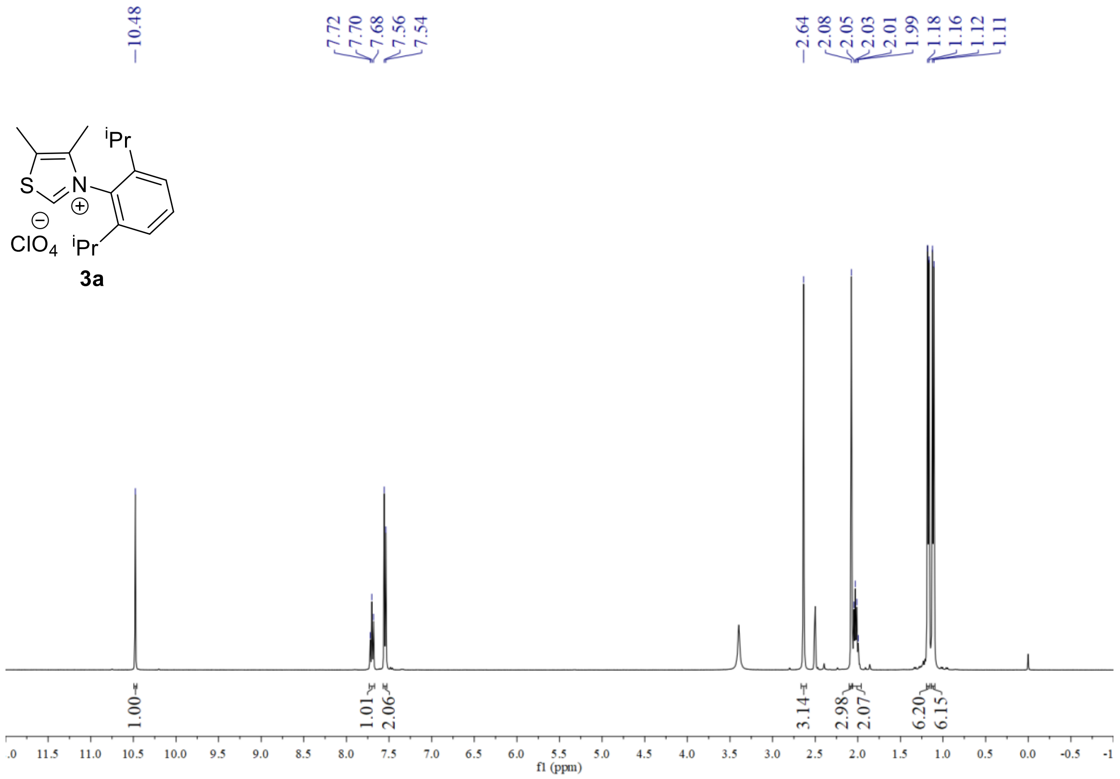


**Supplementary Figure S9.** $^{1}H$ NMR (400 MHz, DMSO-$d_6$) Spectrum of Compound **3a**

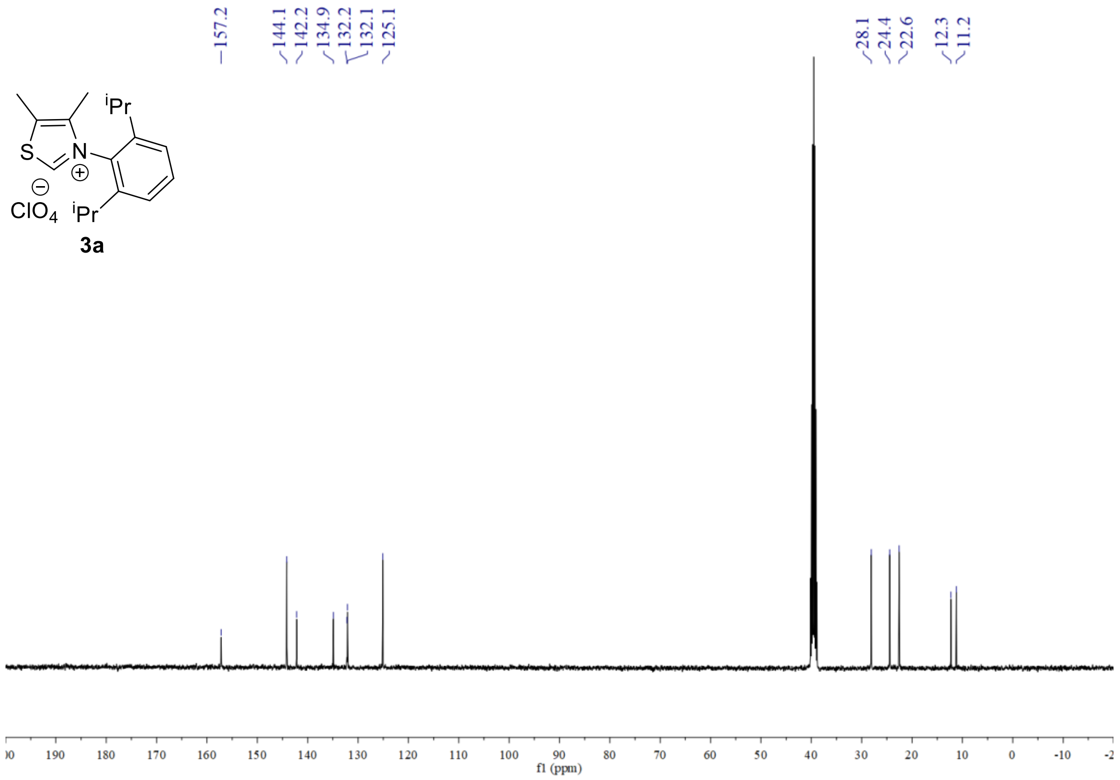


**Supplementary Figure S10.** $^{13}C$ NMR (100 MHz, DMSO-$d_6$) Spectrum of Compound **3a**

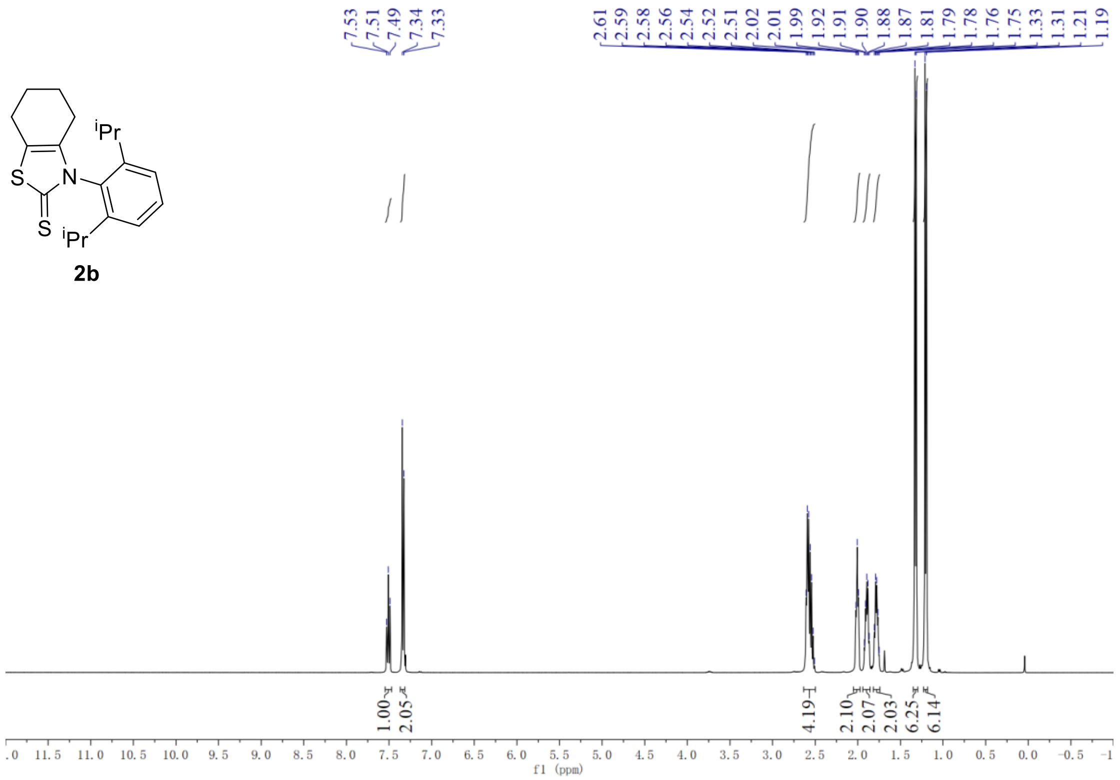


**Supplementary Figure S11.** $^{1}$H NMR (400 MHz, $CDCl_3$) Spectrum of Compound **2b**

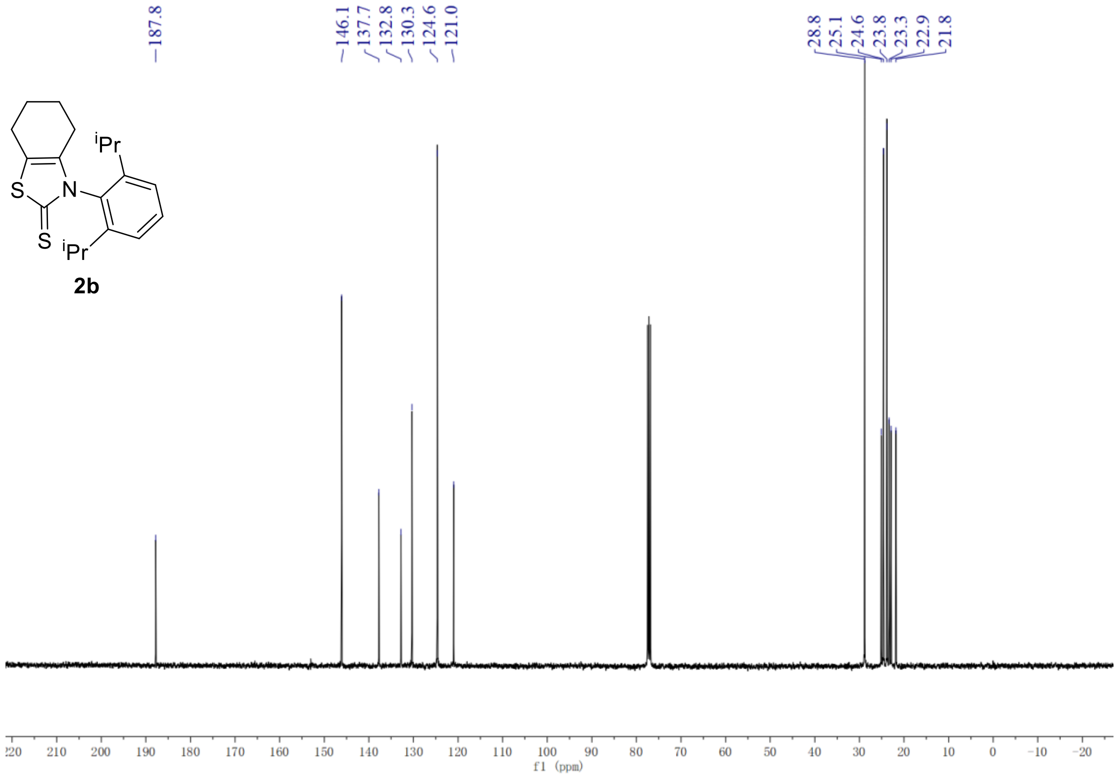


**Supplementary Figure S12.** $^{13}$C NMR (100 MHz, $CDCl_3$) Spectrum of Compound **2b**

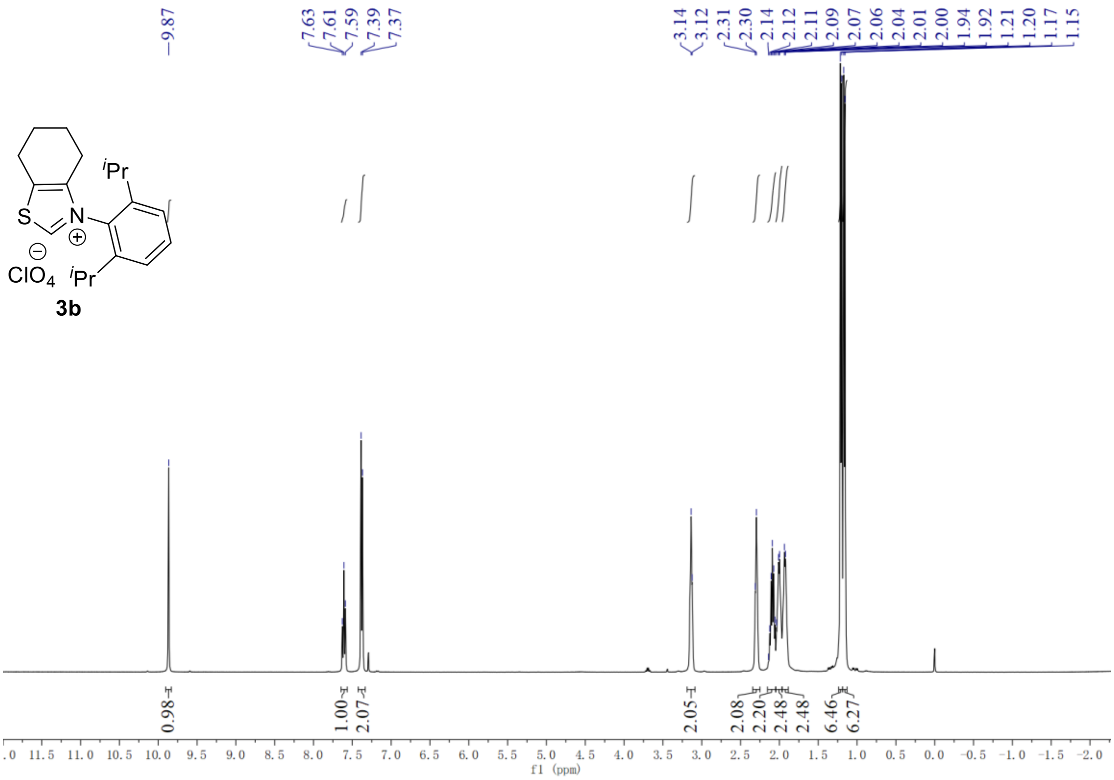


**Supplementary Figure S13.** $^{1}$H NMR (400 MHz, $CDCl_3$) Spectrum of Compound **3b**

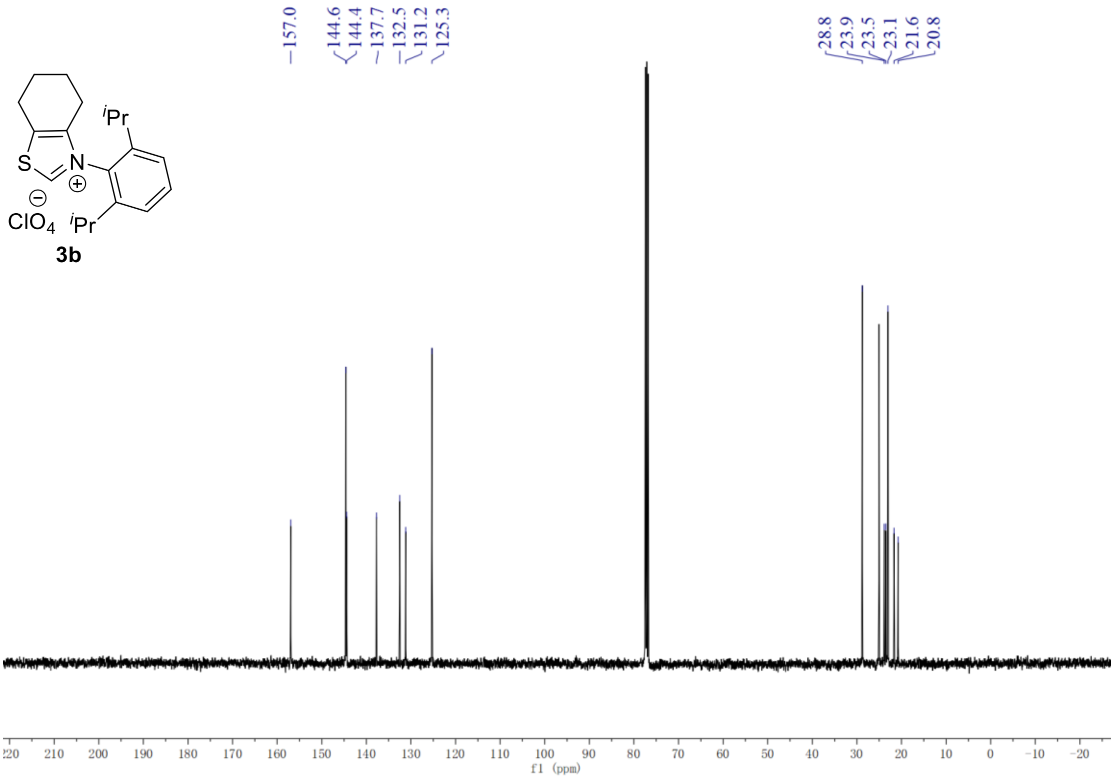


**Supplementary Figure S14.** $^{13}$C NMR (100 MHz, DMSO-$d_6$) Spectrum of Compound **3b**

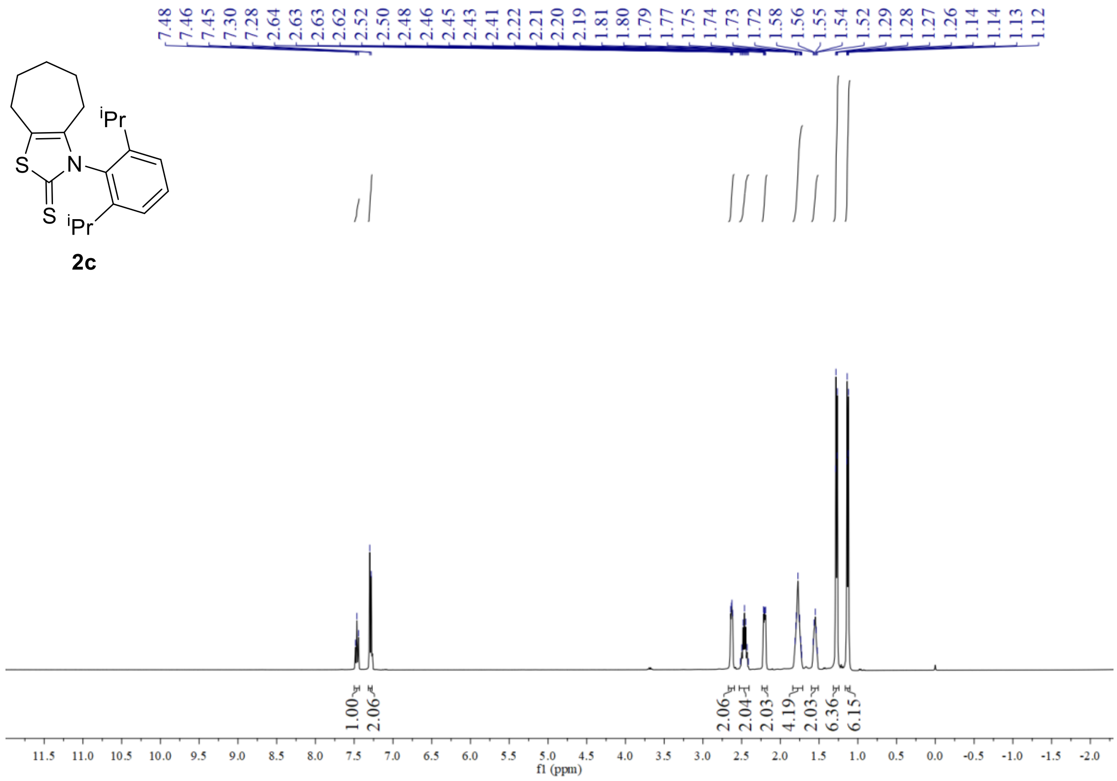

**Supplementary Figure S15.** $^{1}H$ NMR (400 MHz, $CDCl_3$) Spectrum of Compound **2c**

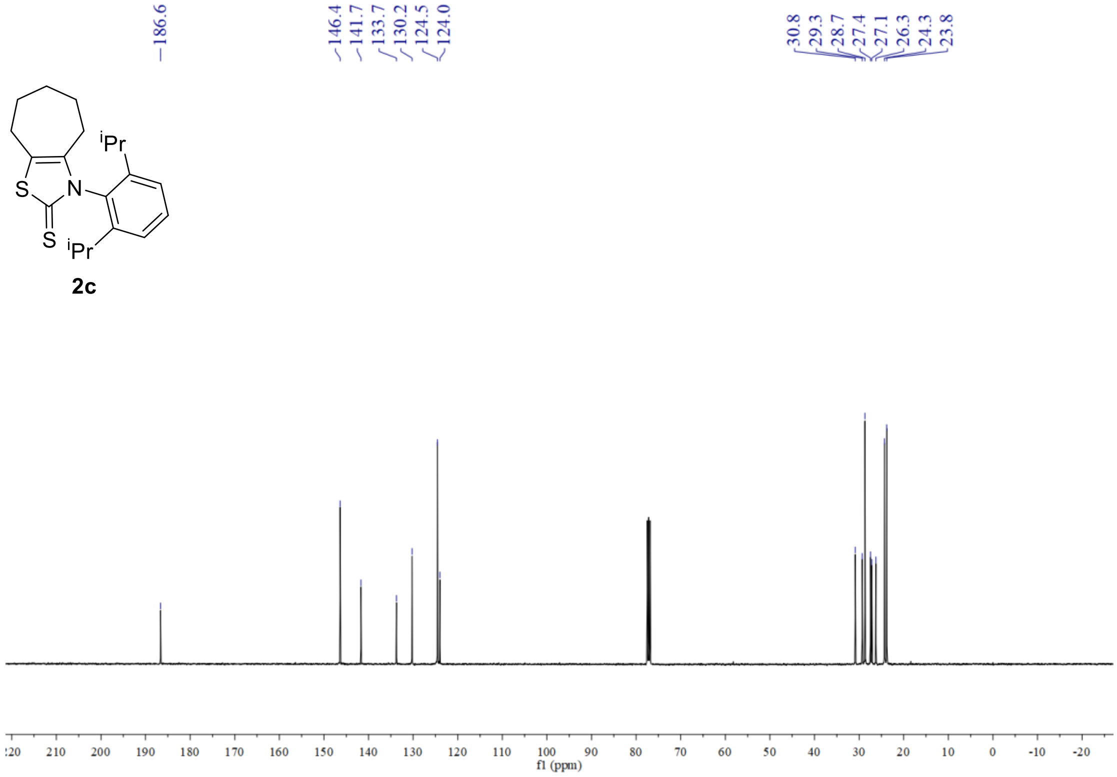

**Supplementary Figure S16.** $^{13}C$ NMR (100 MHz, $CDCl_3$) Spectrum of Compound **2c**

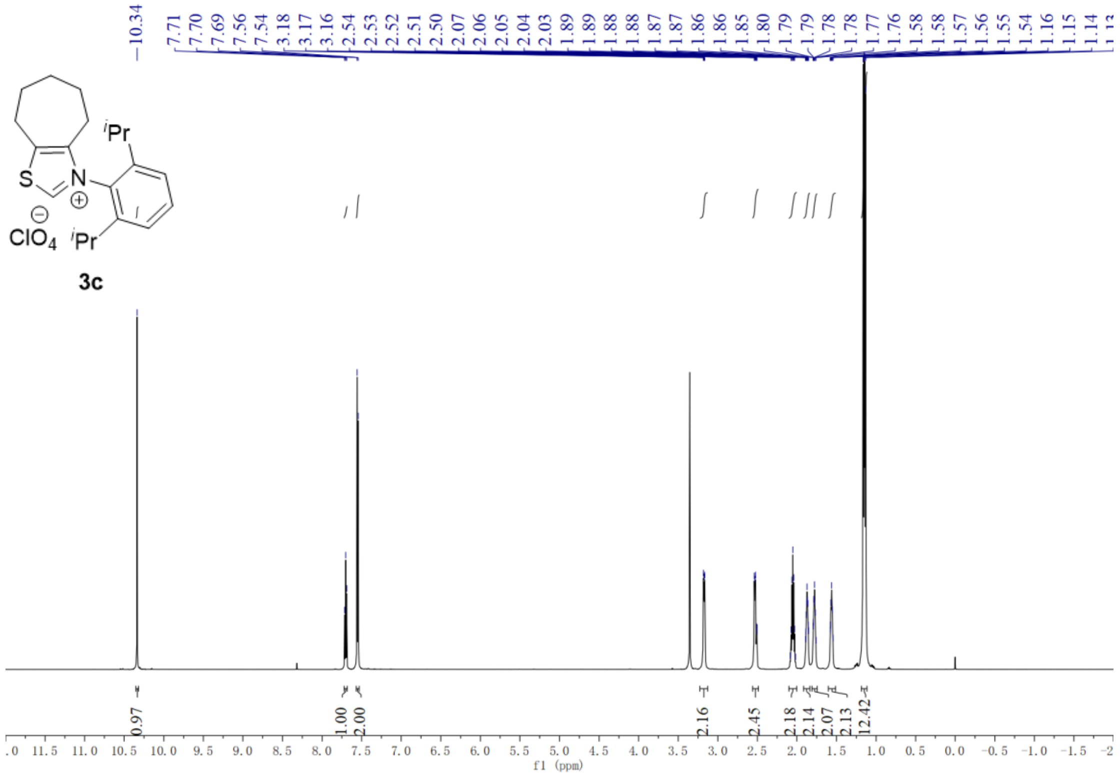


**Supplementary Figure S17.** $^{1}$H NMR (600 MHz, DMSO-$d_6$) Spectrum of Compound **3c**

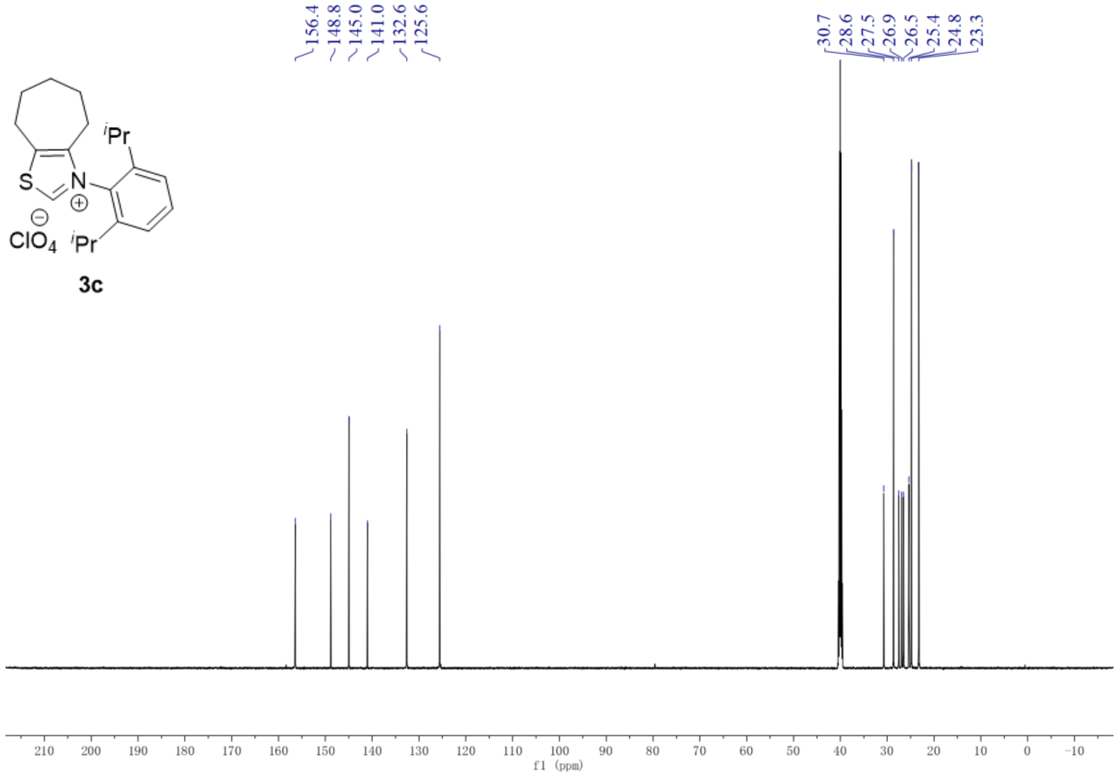


**Supplementary Figure S18.** $^{13}$C NMR (150 MHz, DMSO-$d_6$) Spectrum of Compound **3c**

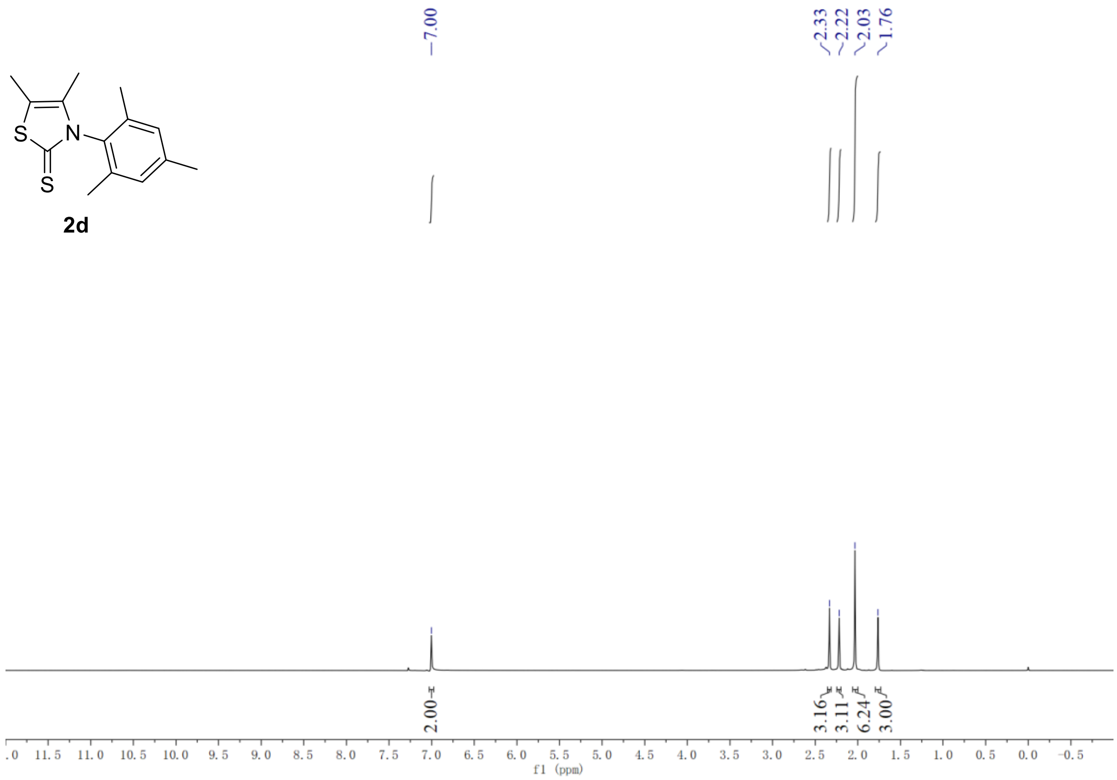


**Supplementary Figure S19.** $^{1}$H NMR (400 MHz, $CDCl_3$) Spectrum of Compound **2d**

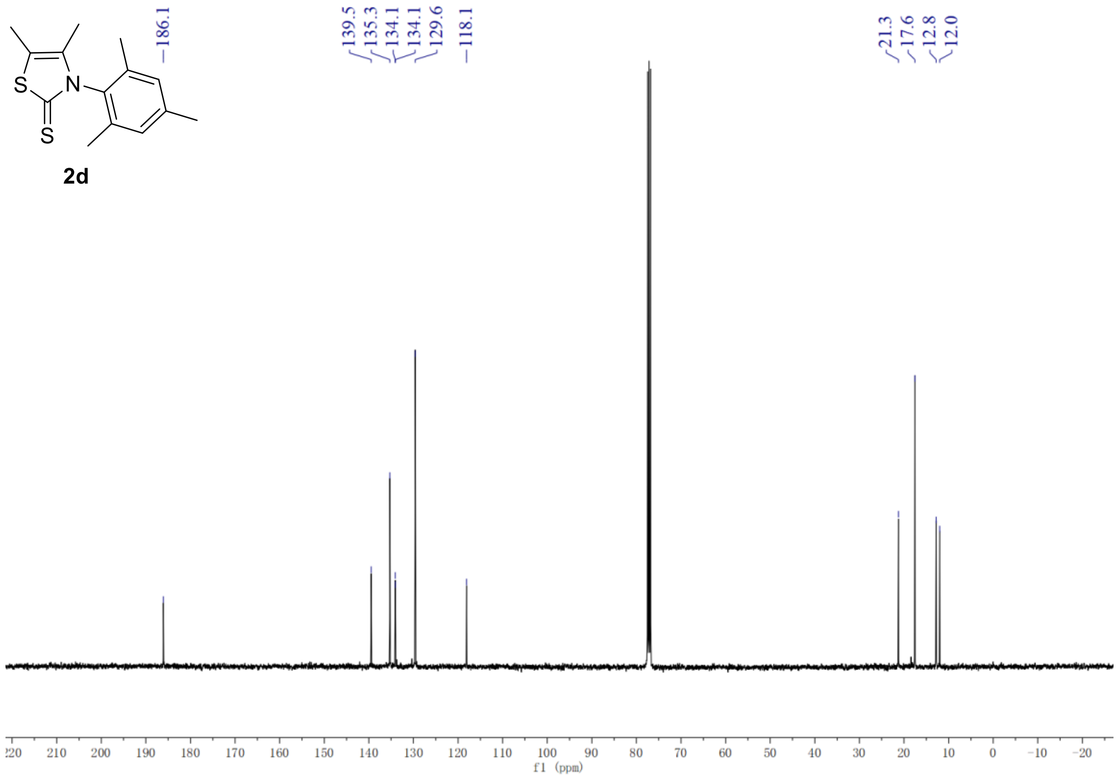


**Supplementary Figure S20.** $^{13}$C NMR (100 MHz, $CDCl_3$) Spectrum of Compound **2d**

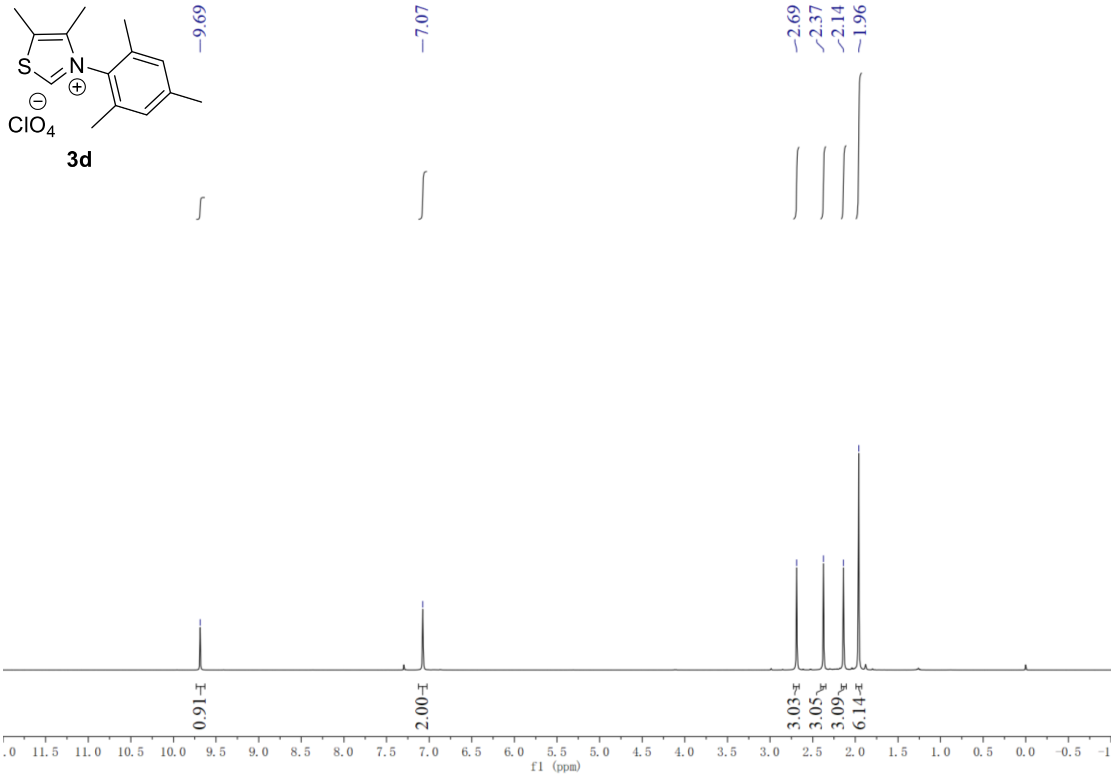


**Supplementary Figure S21.** $^{1}$H NMR (400 MHz, $CDCl_3$) Spectrum of Compound **3d**

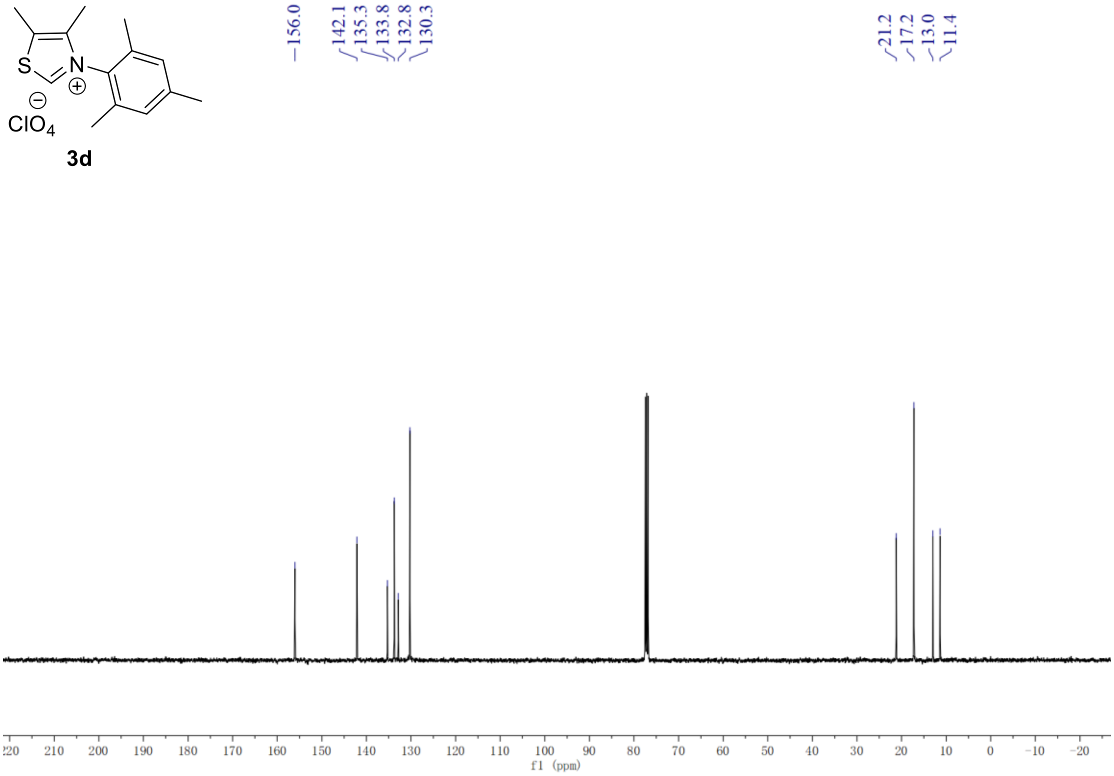


**Supplementary Figure S22.** $^{13}$C NMR (100 MHz, $CDCl_3$) Spectrum of Compound **3d**

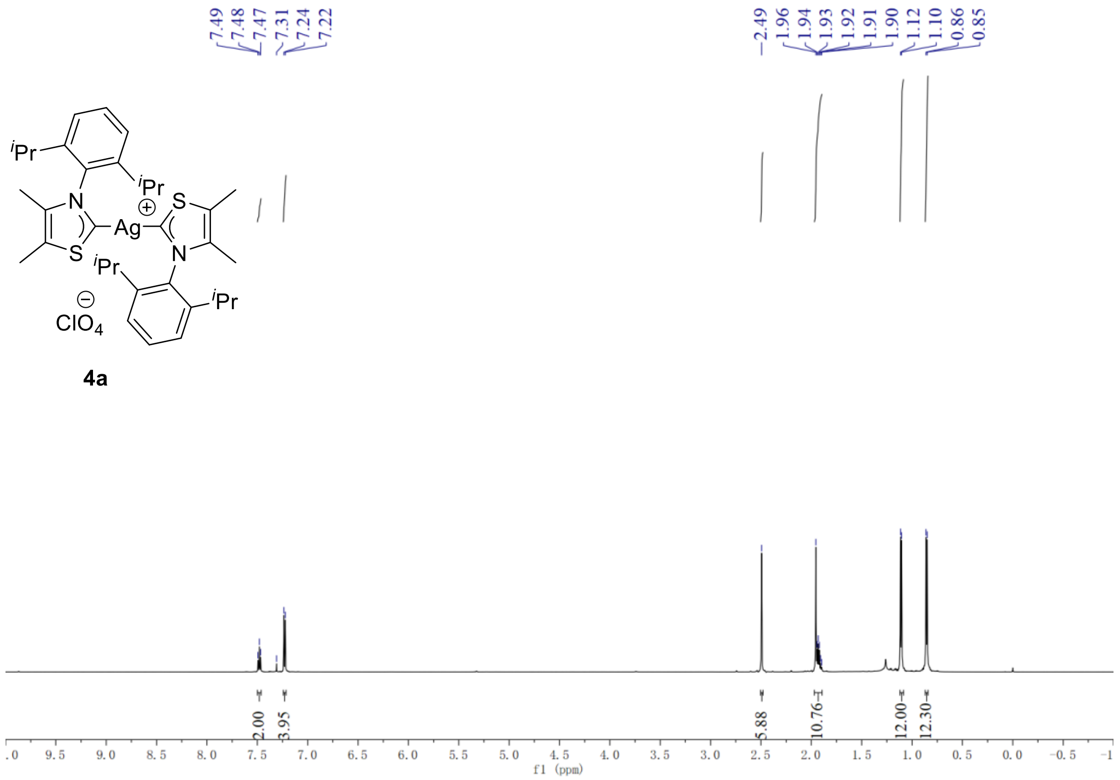


**Supplementary Figure S23.** $^{1}$H NMR (600 MHz, $CDCl_3$) Spectrum of Compound **4a**

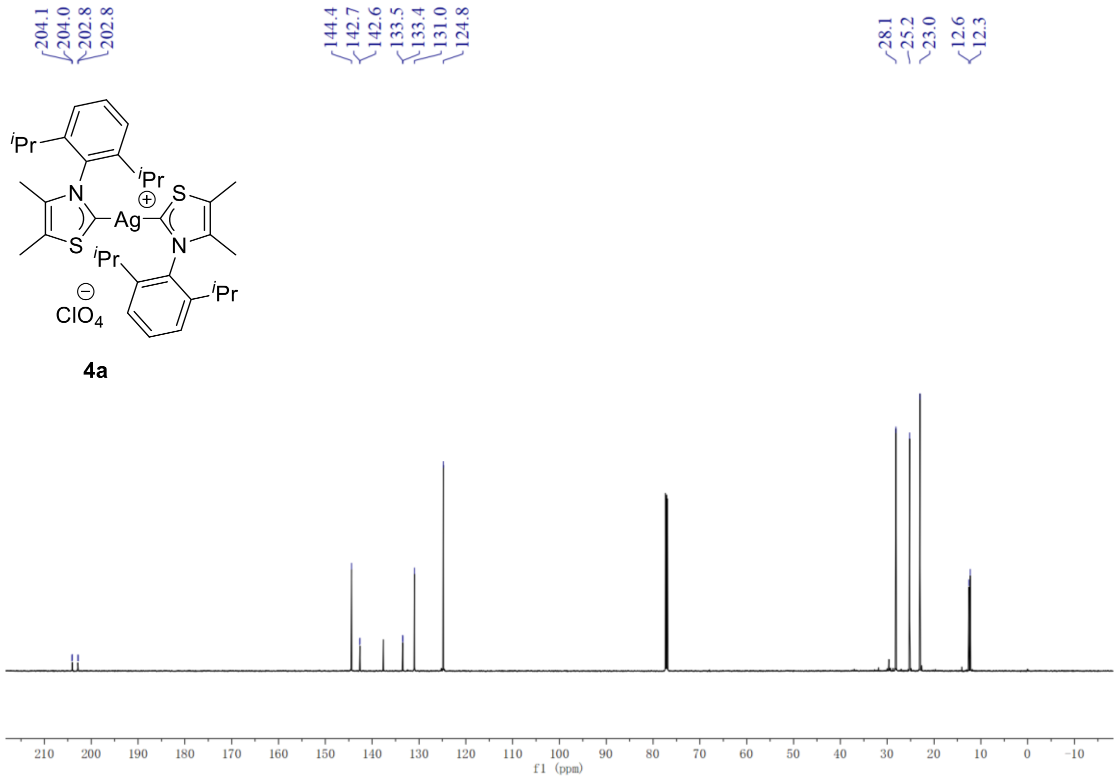


**Supplementary Figure S24.** $^{13}$C NMR (150 MHz, $CDCl_3$) Spectrum of Compound **4a**

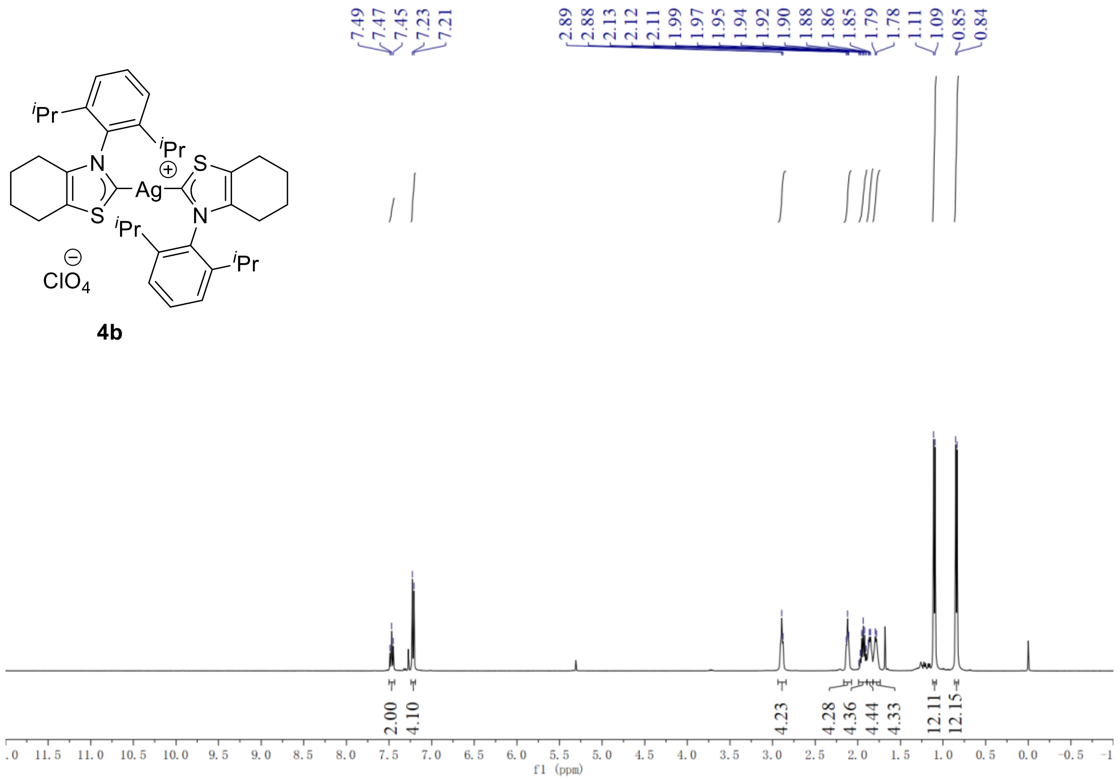


**Supplementary Figure S25.** $^{1}$H NMR (400 MHz, CDCl3) Spectrum of Compound **4b**

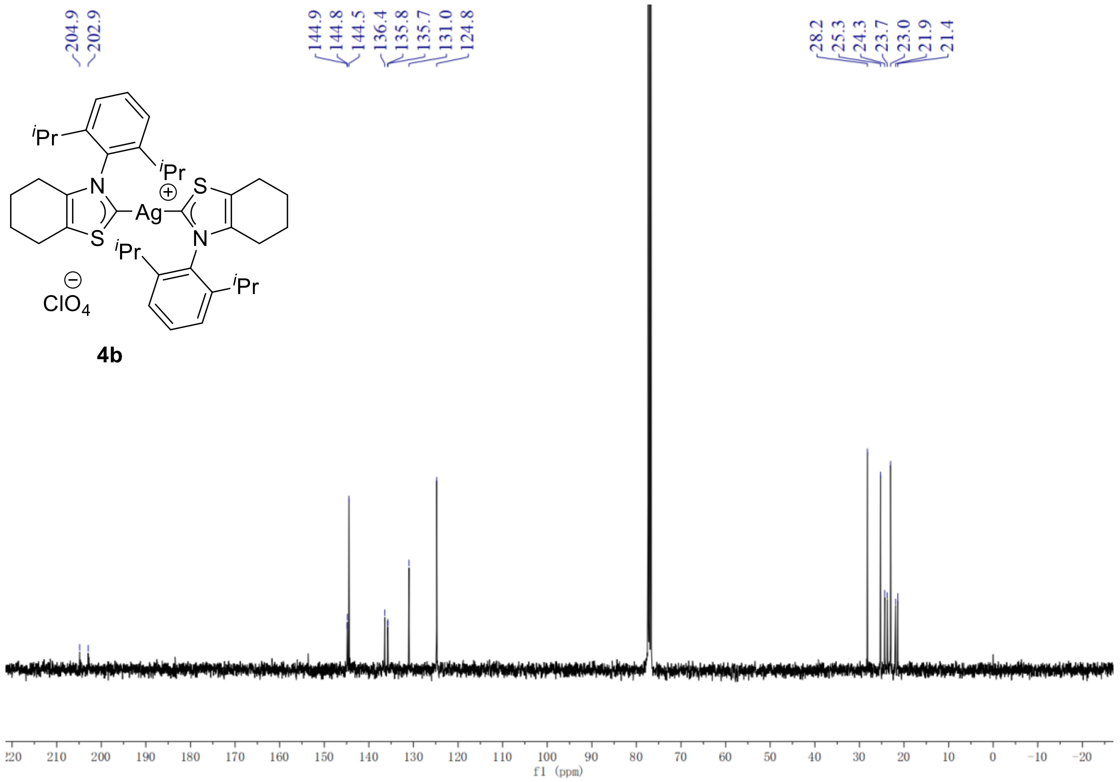


**Supplementary Figure S26.** $^{13}$C NMR (100 MHz, CDCl3) Spectrum of Compound **4b**

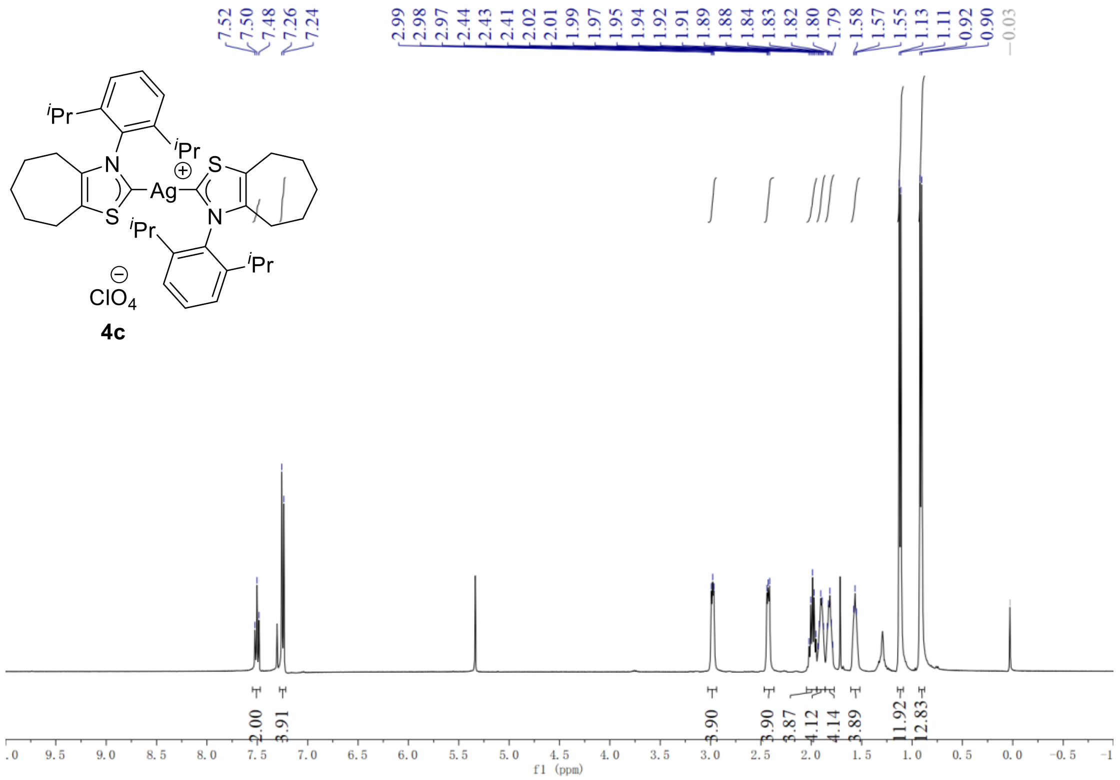

**Supplementary Figure S27.** $^{1}$H NMR (600 MHz, $CDCl_3$) Spectrum of Compound **4c**

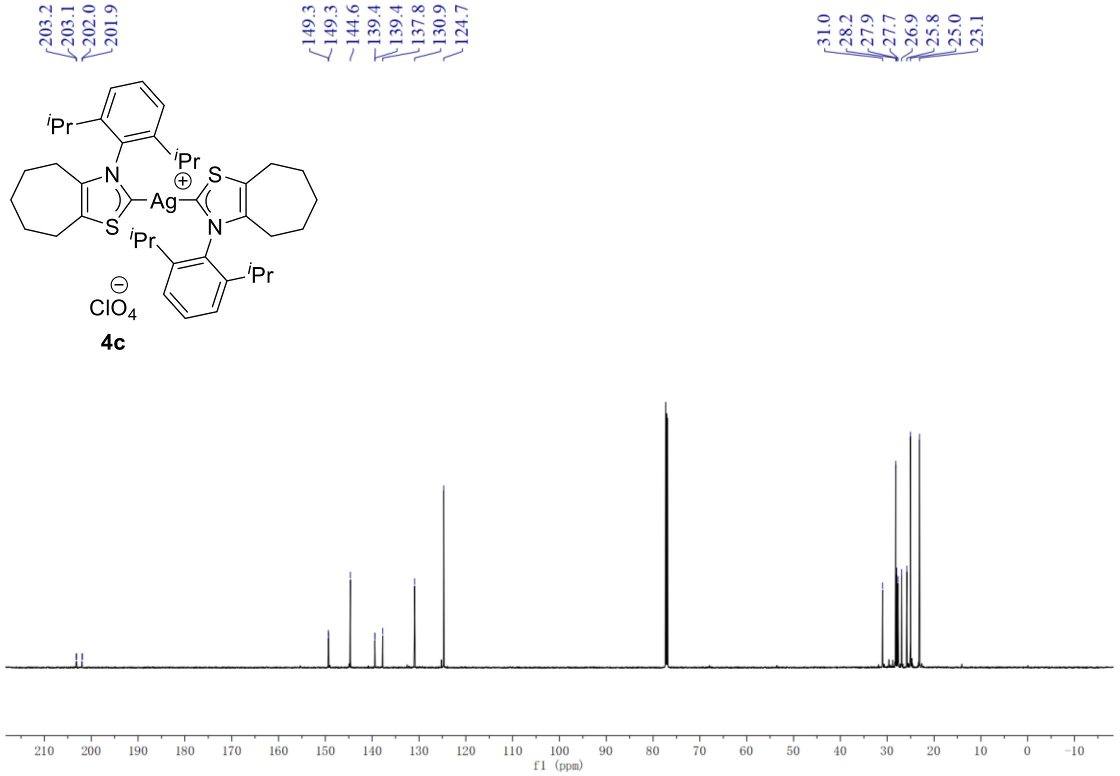

**Supplementary Figure S28.** $^{13}$C NMR (150 MHz, $CDCl_3$) Spectrum of Compound **4c**

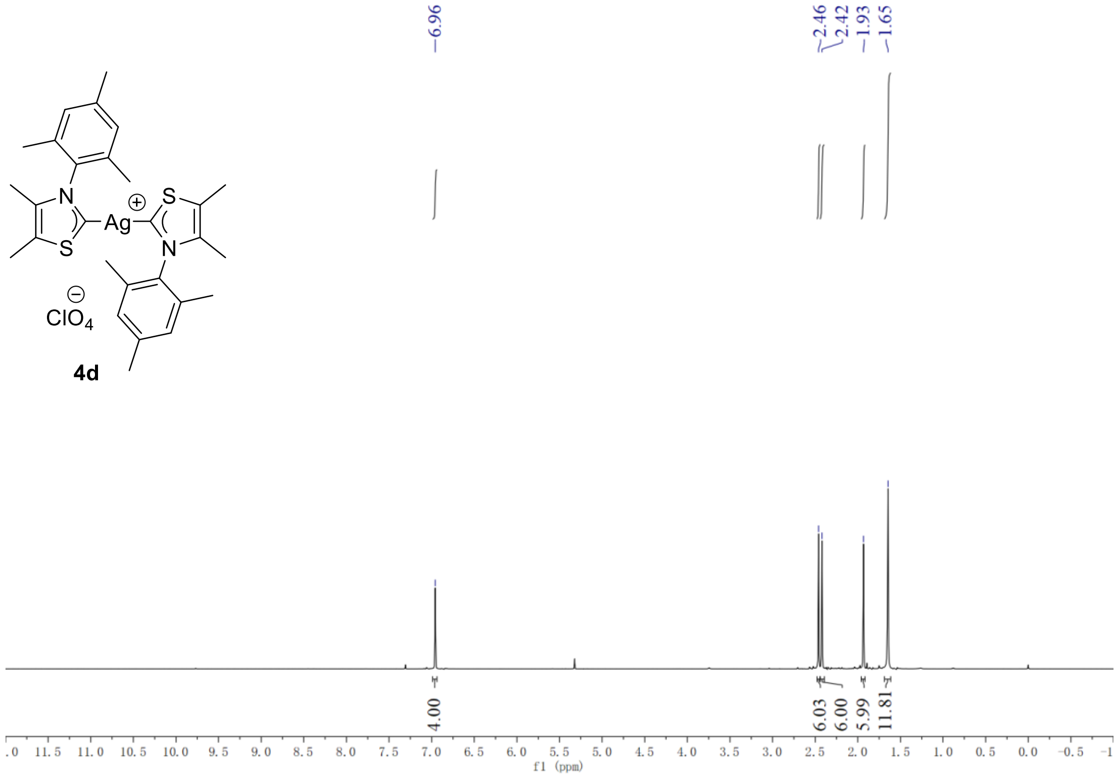


**Supplementary Figure S29.** $^{1}H$ NMR (600 MHz, $CDCl_3$) Spectrum of Compound **4d**

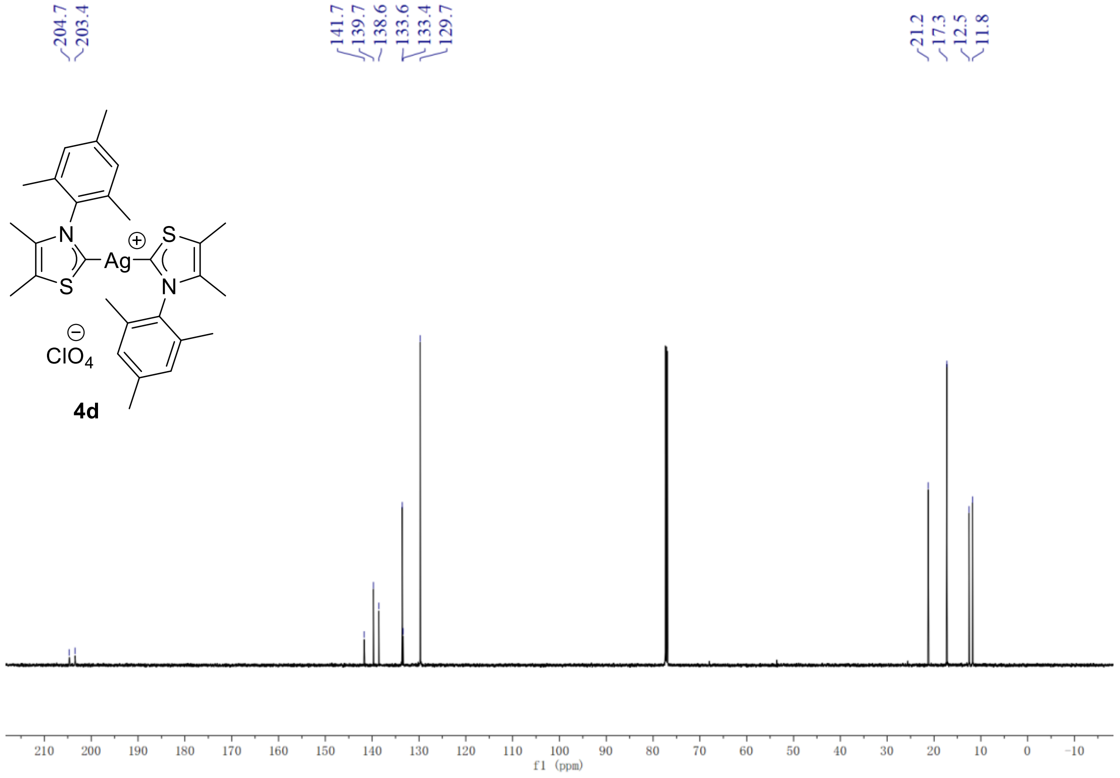


**Supplementary Figure S30.** $^{13}C$ NMR (150 MHz, $CDCl_3$) Spectrum of Compound **4d**

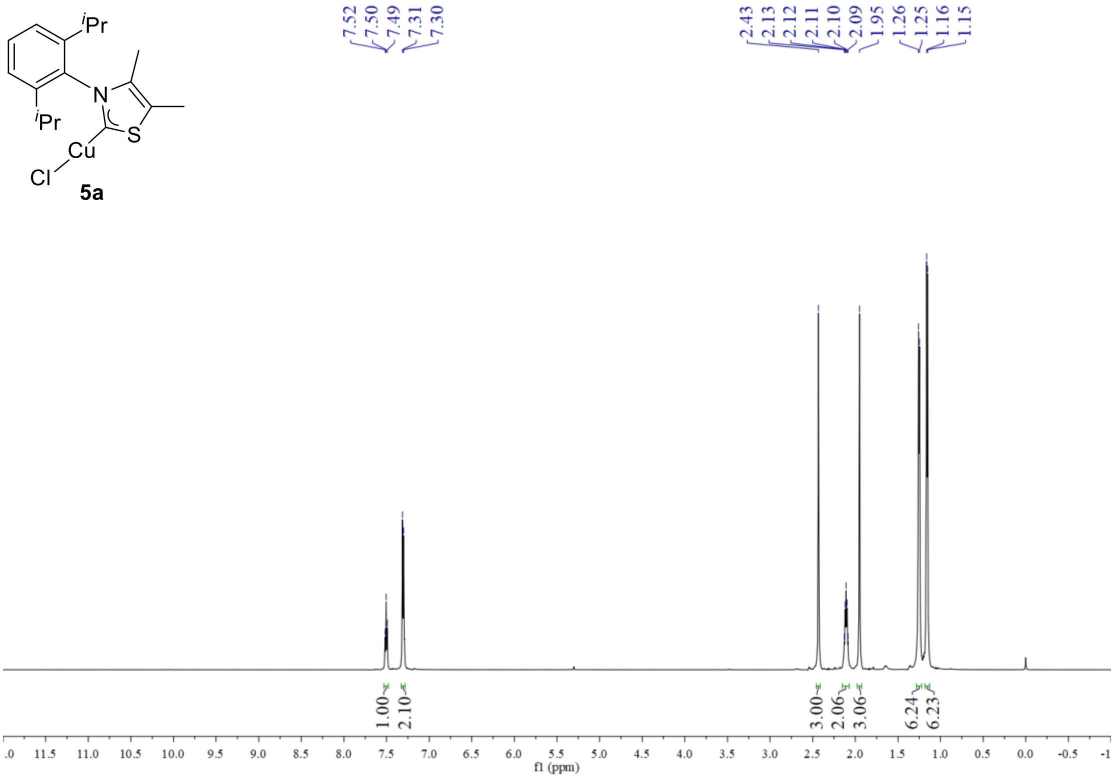


**Supplementary Figure S31.** $^1$H NMR (600 MHz, $CDCl_3$) Spectrum of Compound **5a**

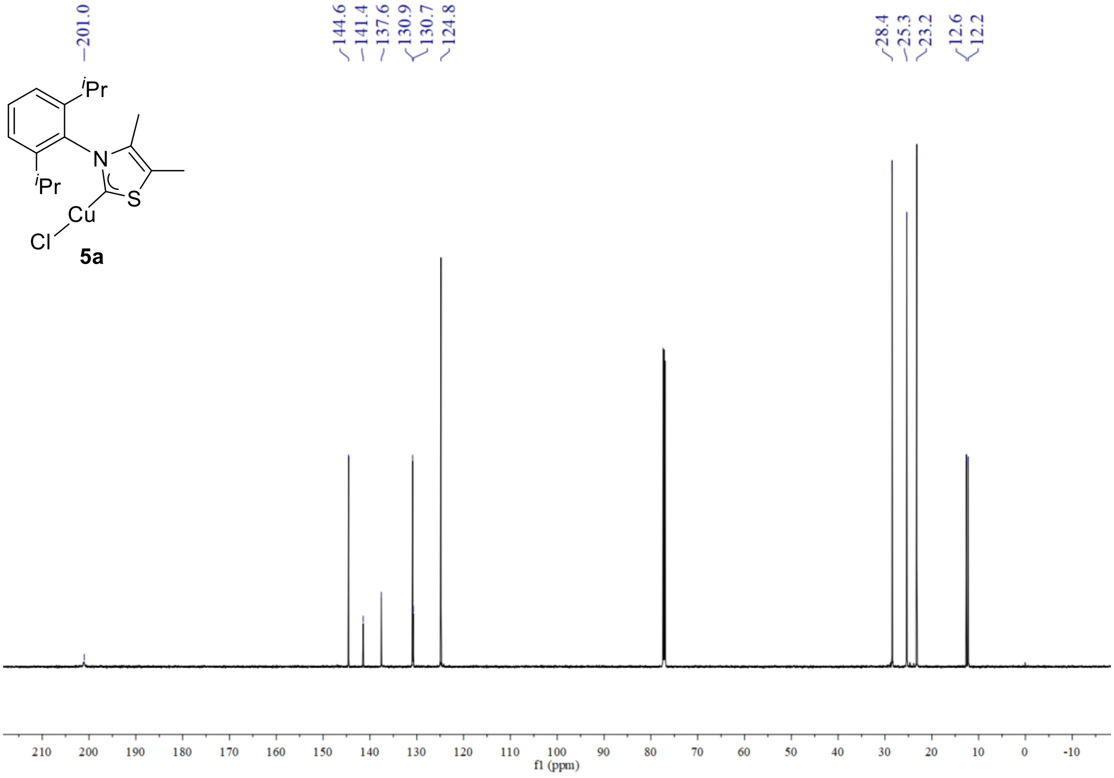


**Supplementary Figure S32.** $^{13}$C NMR (150 MHz, $CDCl_3$) Spectrum of Compound **5a**

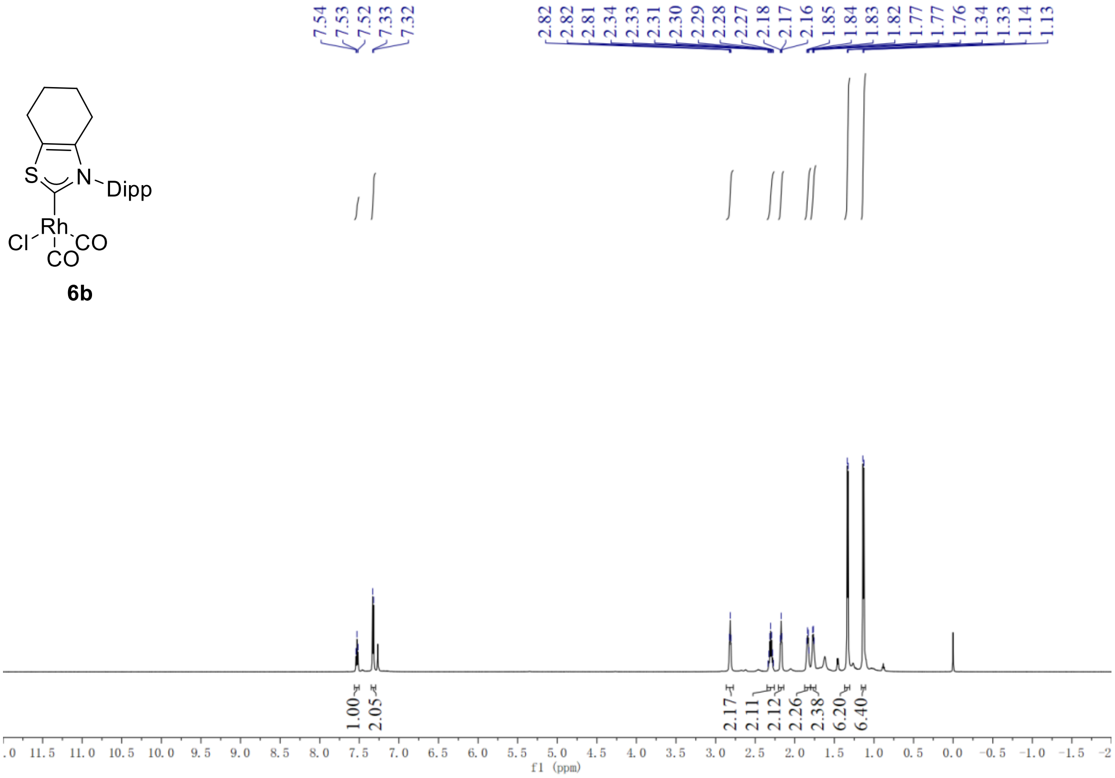


**Supplementary Figure S33.** $^{1}$H NMR (600 MHz, $CDCl_3$) Spectrum of Compound **6b**

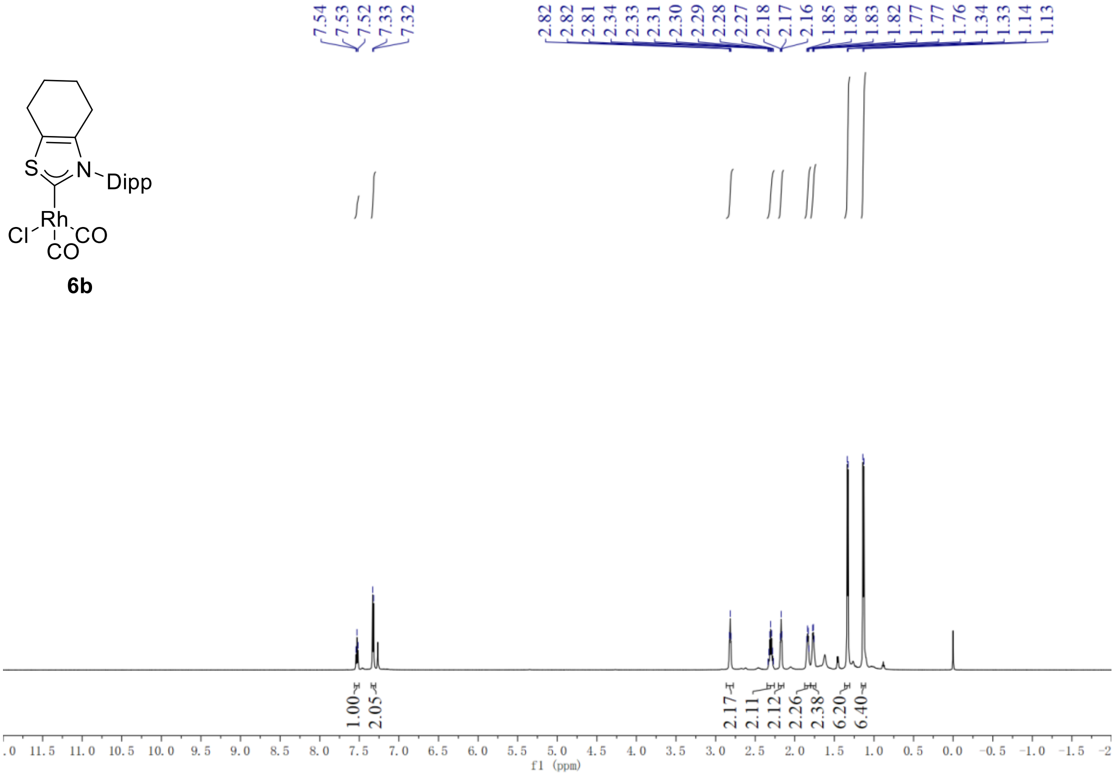


**Supplementary Figure S34.** $^{13}$C NMR (150 MHz, $CDCl_3$) Spectrum of Compound **6b**

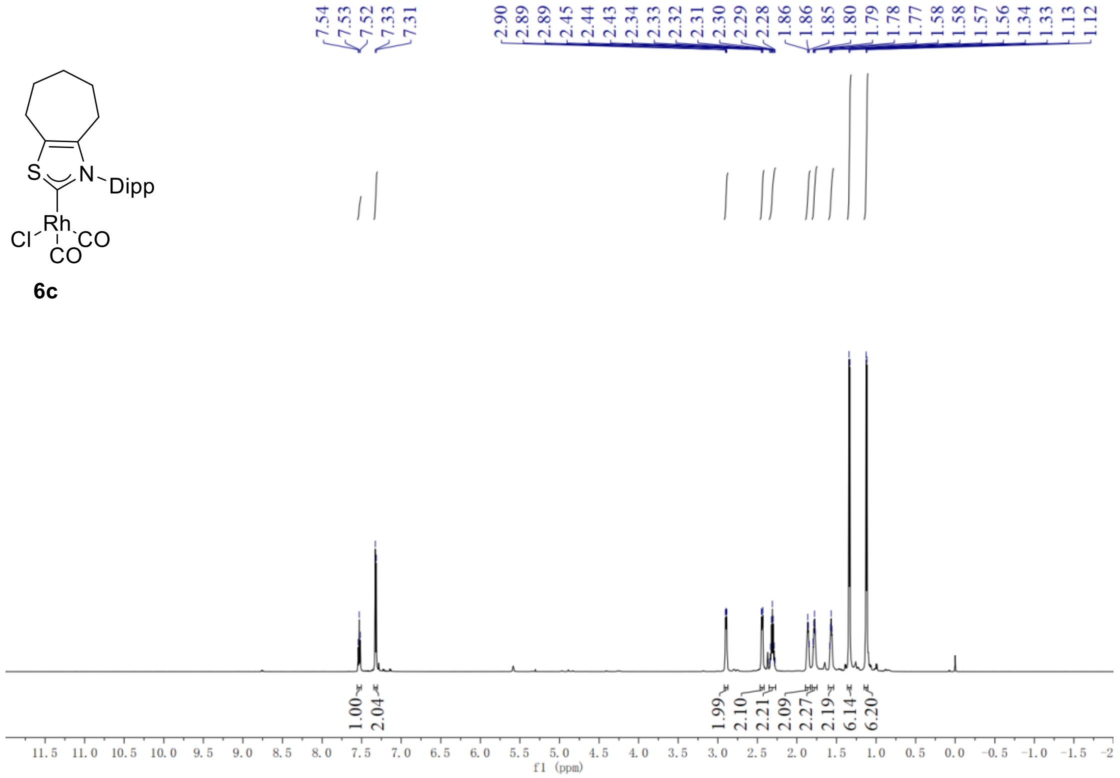


**Supplementary Figure S35.** $^{1}H$ NMR (600 MHz, $CDCl_3$) Spectrum of Compound **6c**

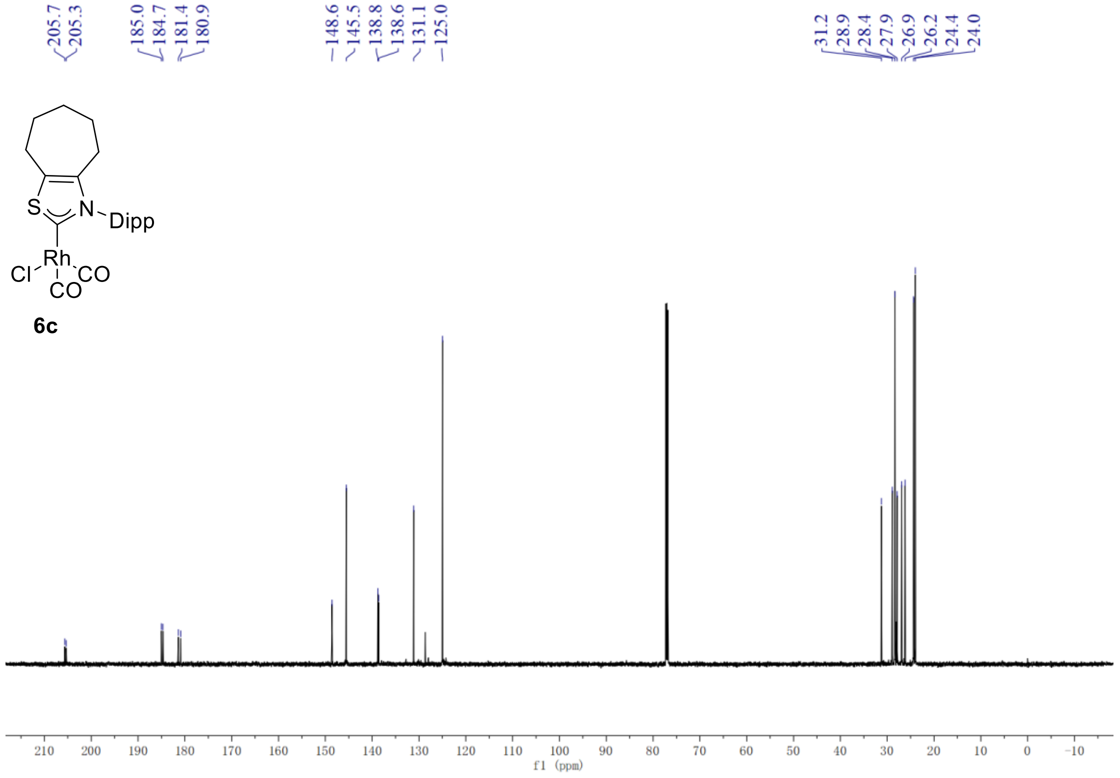


**Supplementary Figure S36.** $^{13}C$ NMR (150 MHz, $CDCl_3$) Spectrum of Compound **6c**

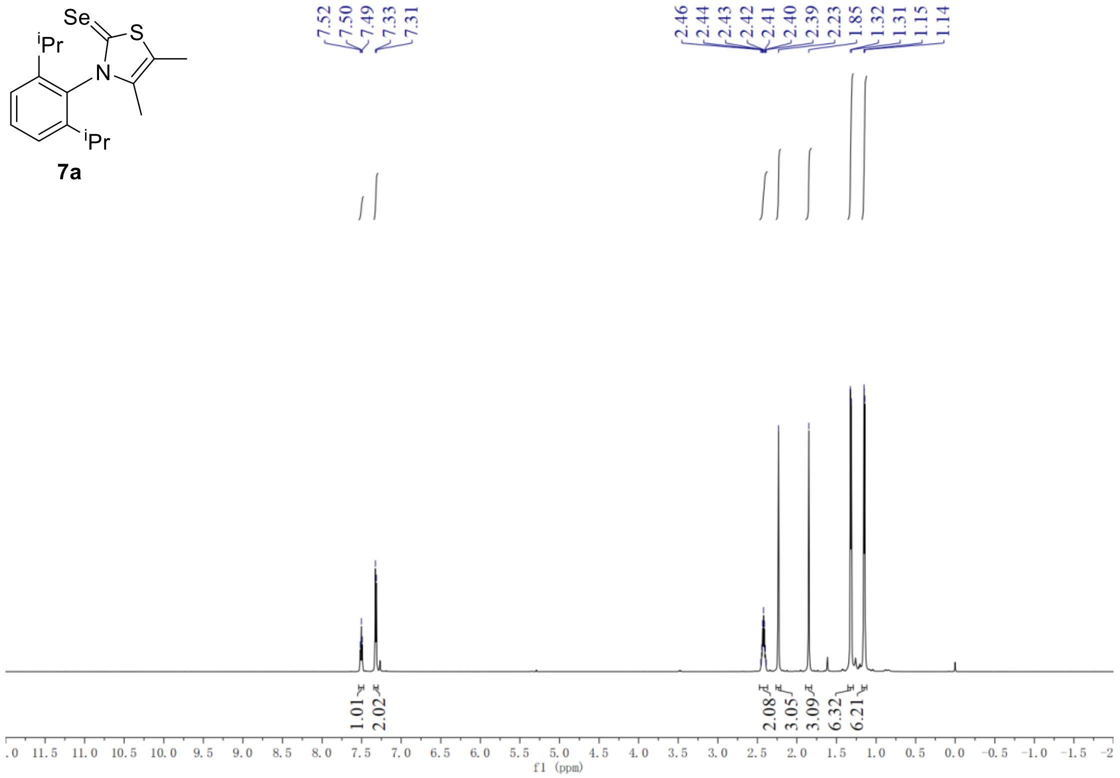


**Supplementary Figure S37.** $^{1}H$ NMR (600 MHz, $CDCl_3$) Spectrum of Compound **7a**

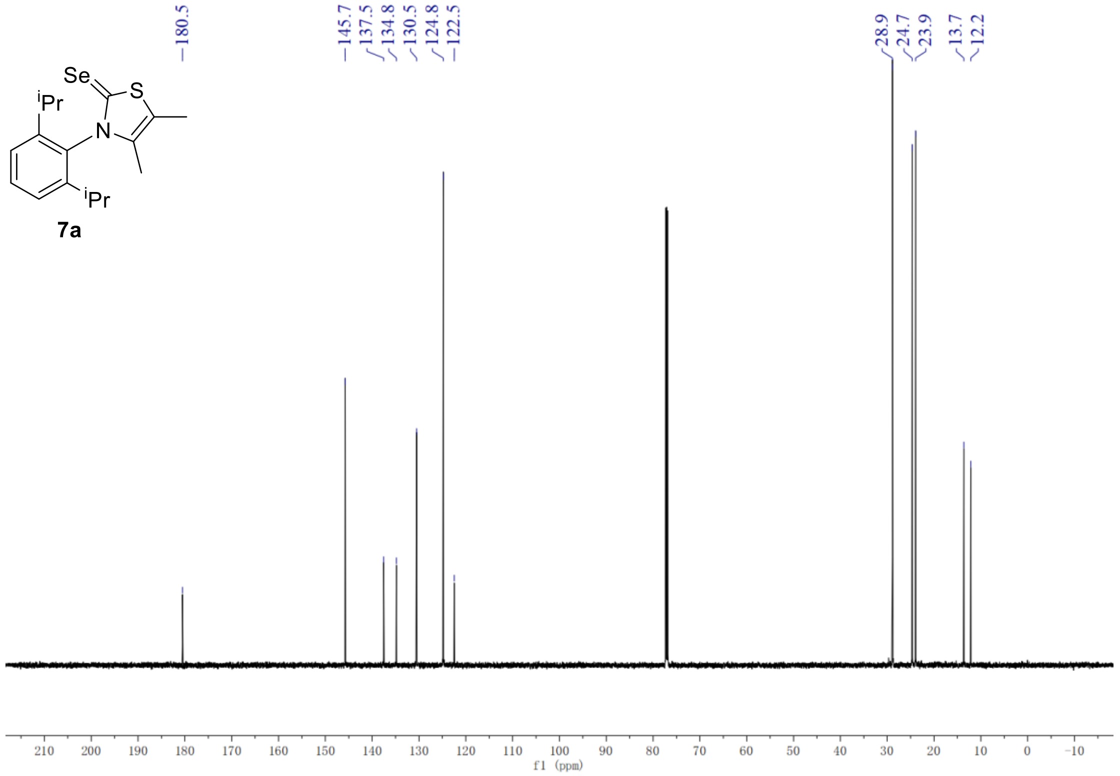


**Supplementary Figure S38.** $^{13}C$ NMR (150 MHz, $CDCl_3$) Spectrum of Compound **7a**

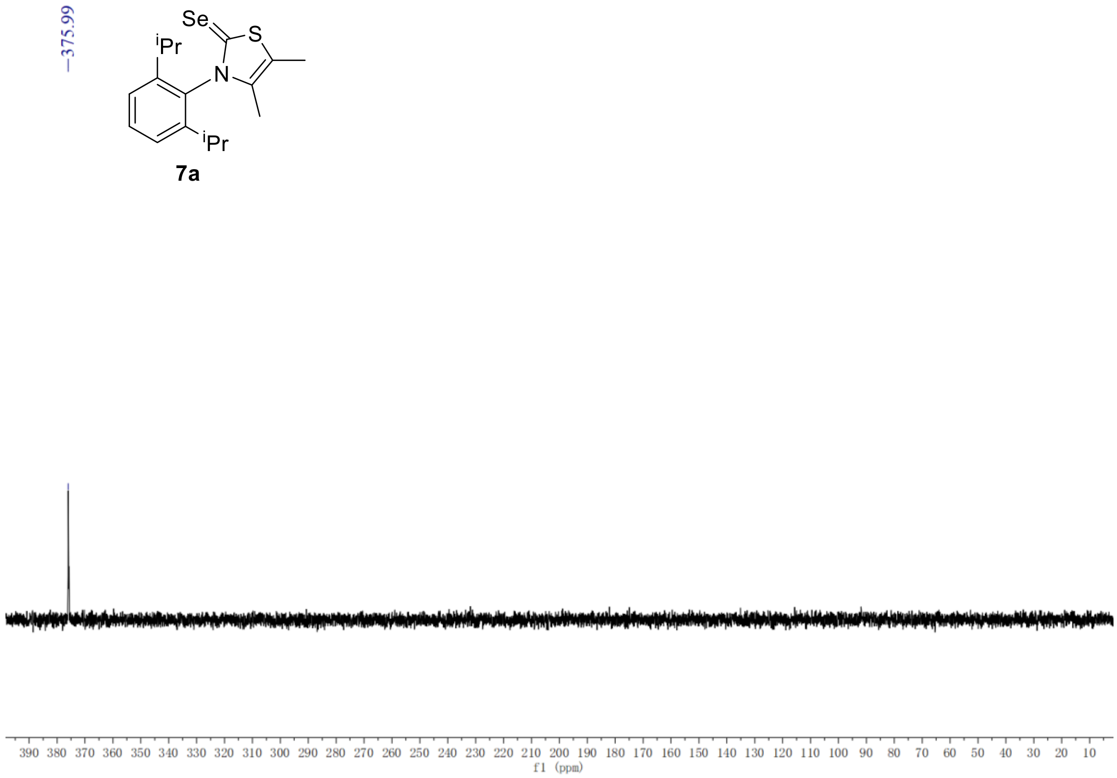


**Supplementary Figure S39.** $^{77}$Se NMR (114 MHz, $CDCl_3$) Spectrum of Compound **5a**

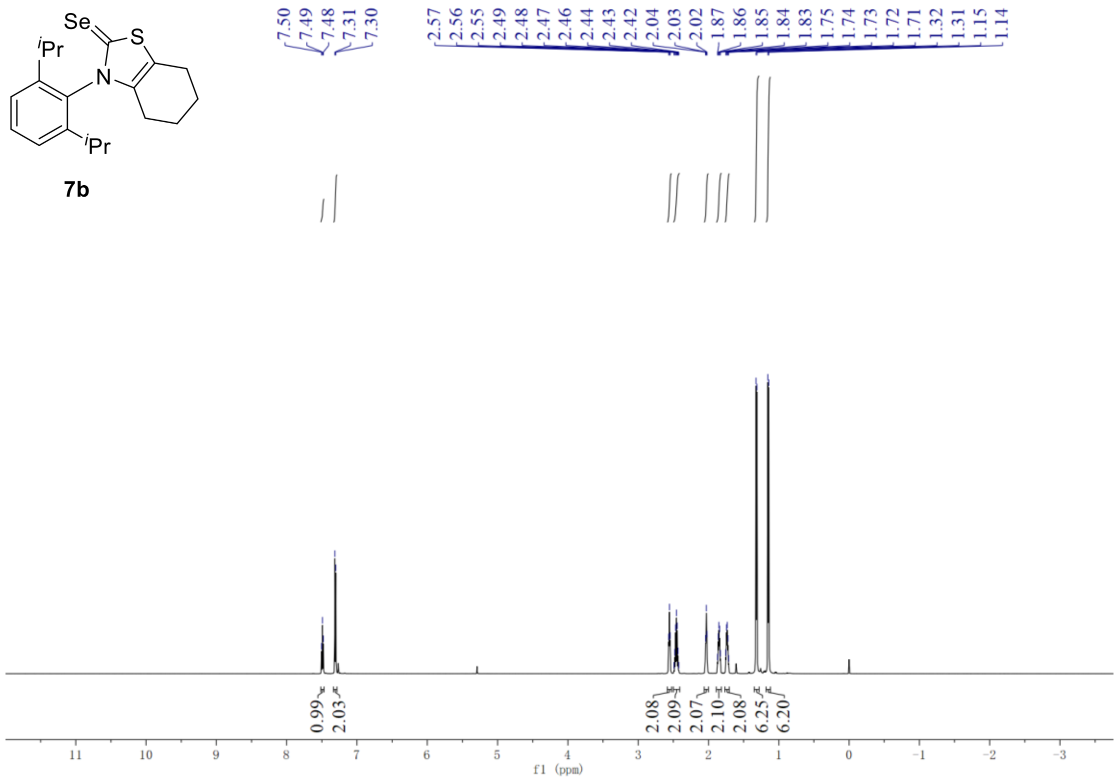


**Supplementary Figure S40.** $^{1}$H NMR (600 MHz, CDCl3) Spectrum of Compound **7b**

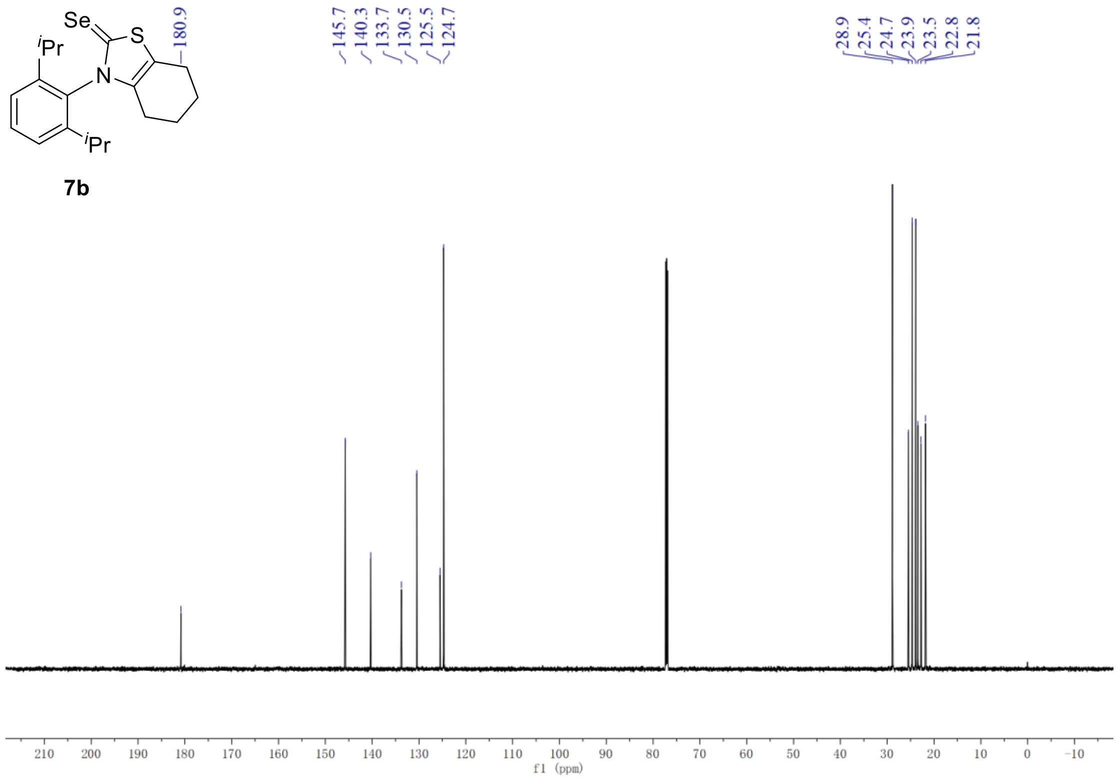


**Supplementary Figure S41.** $^{13}$C NMR (150 MHz, CDCl3) Spectrum of Compound **7b**

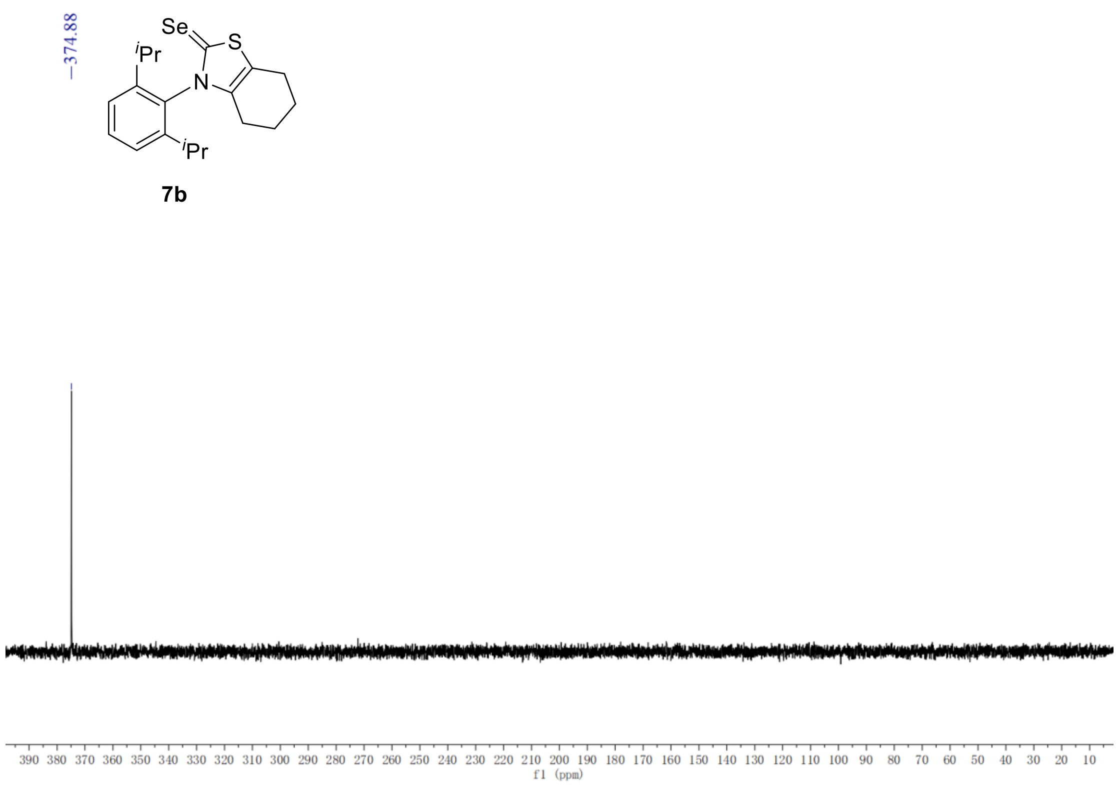


**Supplementary Figure S42.** $^{77}$Se NMR (114 MHz, $CDCl_3$) Spectrum of Compound **7b**

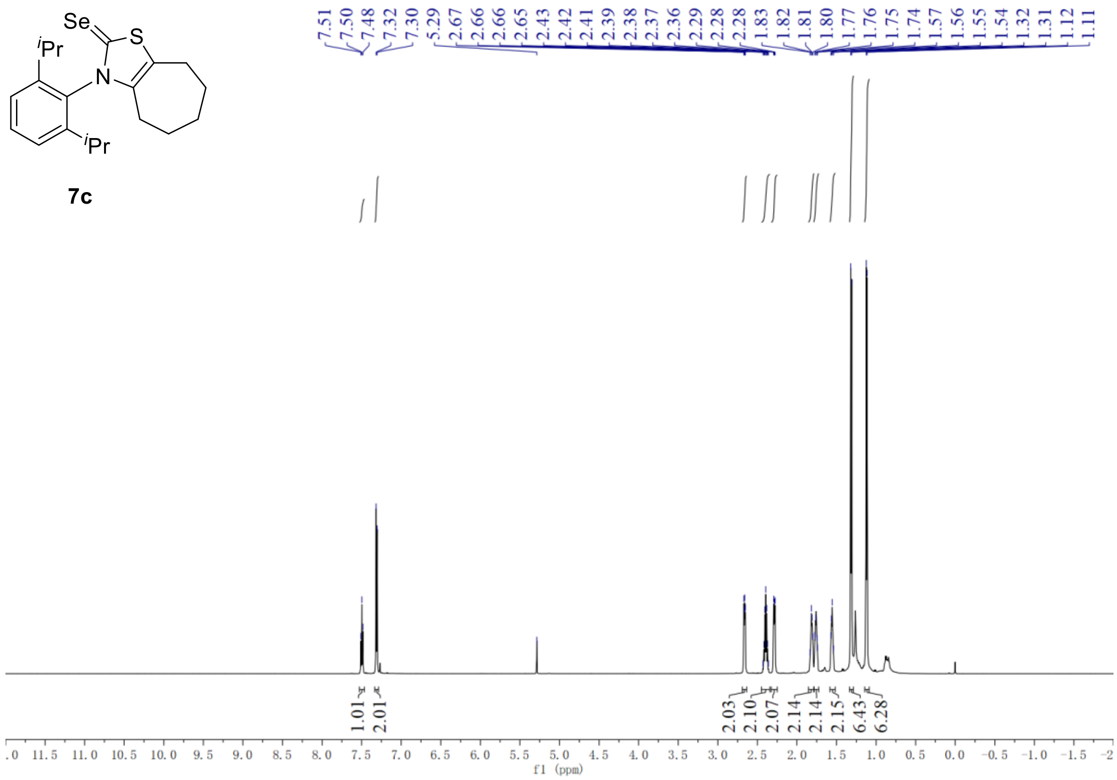


**Supplementary Figure S43.** $^{1}$H NMR (600 MHz, $CDCl_3$) Spectrum of Compound **7c**

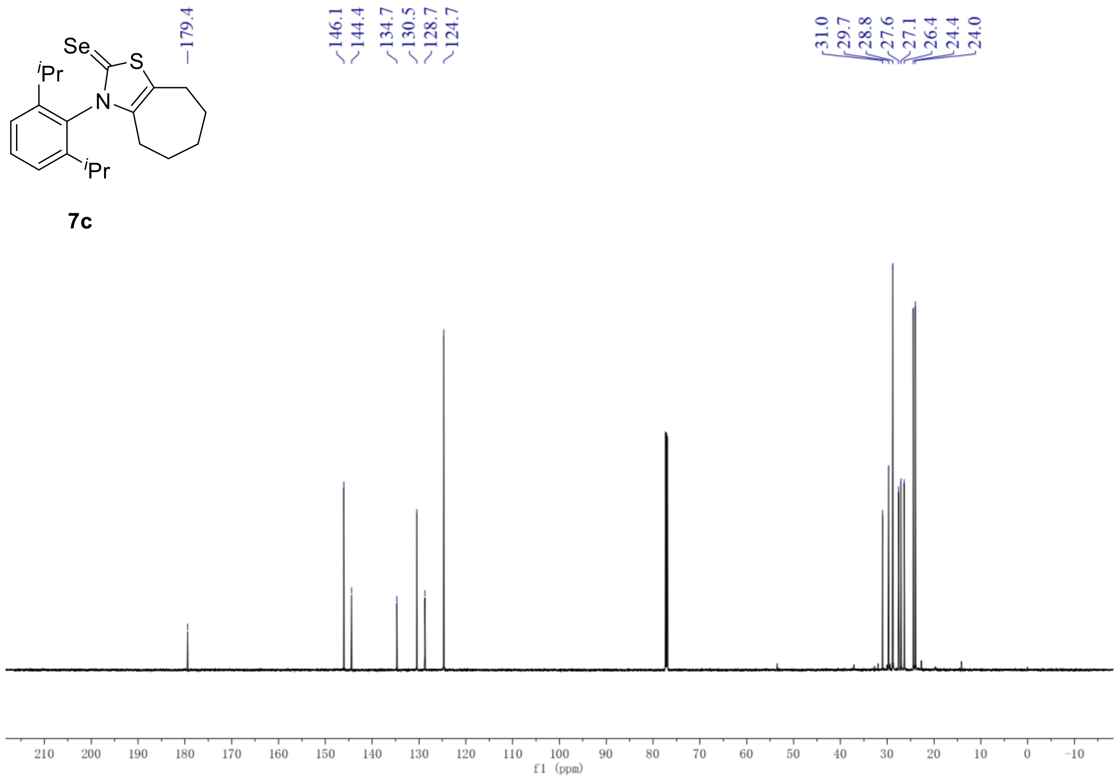


**Supplementary Figure S44.** $^{13}$C NMR (150 MHz, $CDCl_3$) Spectrum of Compound **7c**

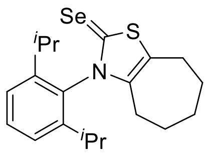


**7c**

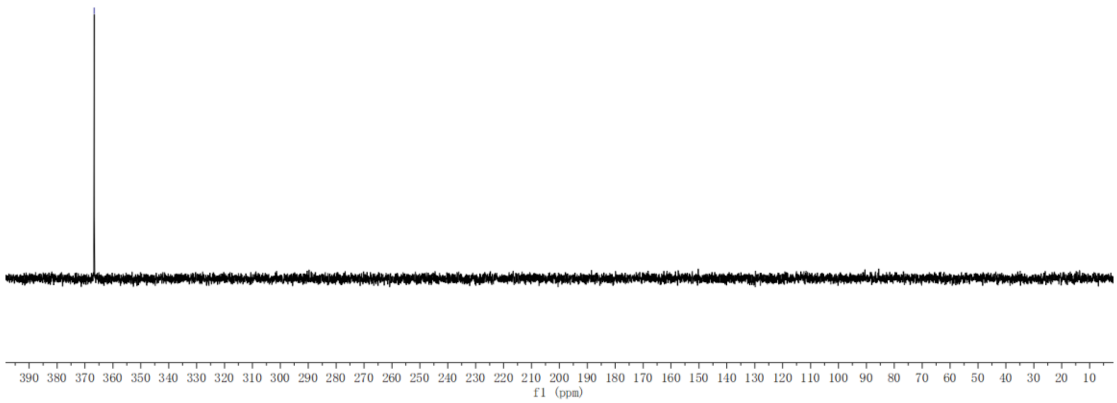


**Supplementary Figure S45.** $^{77}$Se NMR (114 MHz, $CDCl_3$) Spectrum of Compound **7c**

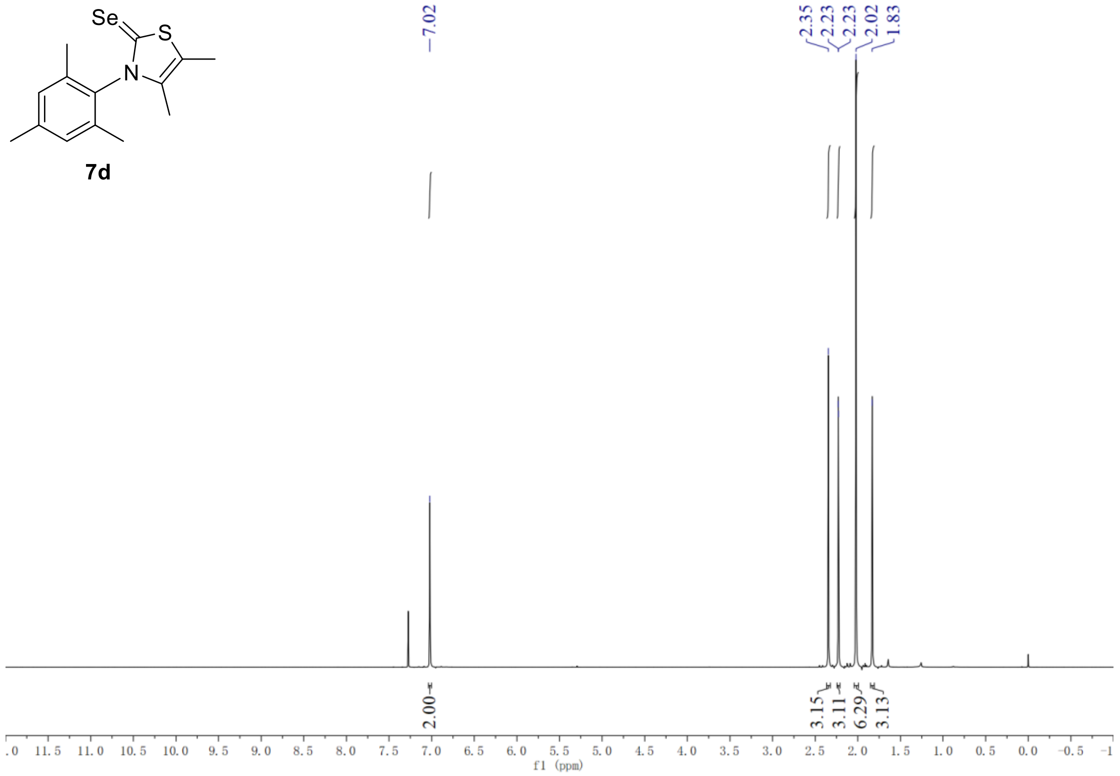


**Supplementary Figure S46.** $^{1}$H NMR (600 MHz, $CDCl_3$) Spectrum of Compound **7d**

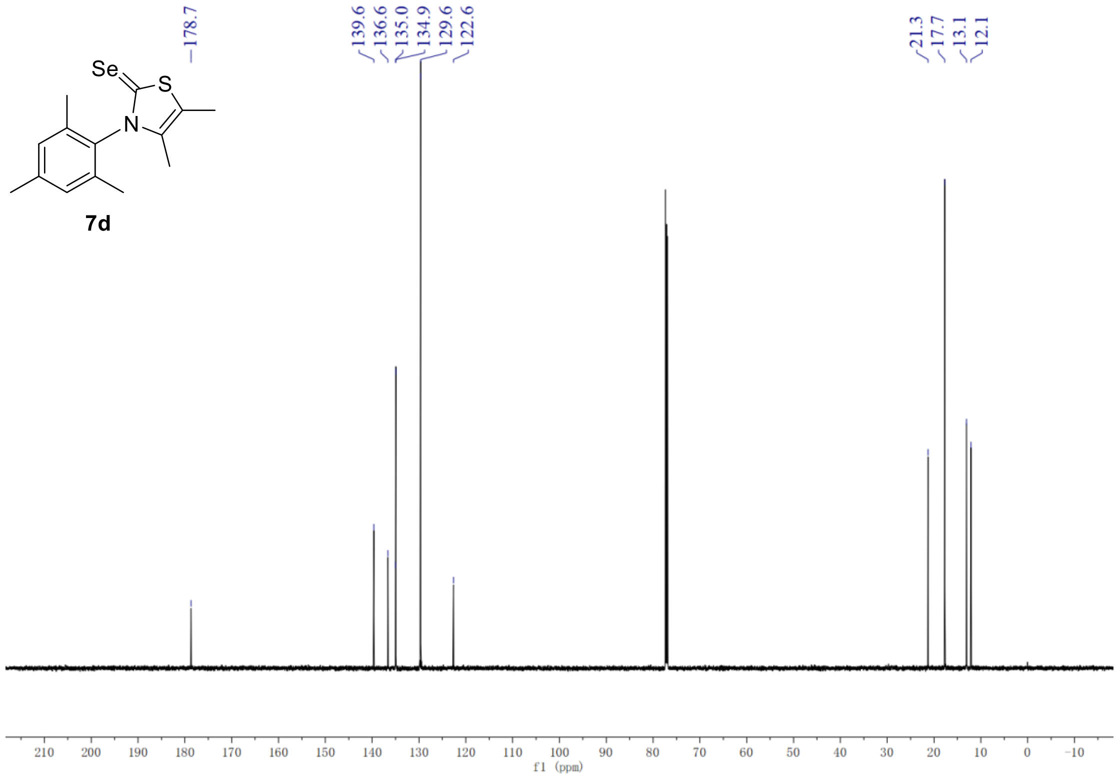


**Supplementary Figure S47.** $^{13}$C NMR (150 MHz, $CDCl_3$) Spectrum of Compound **7d**

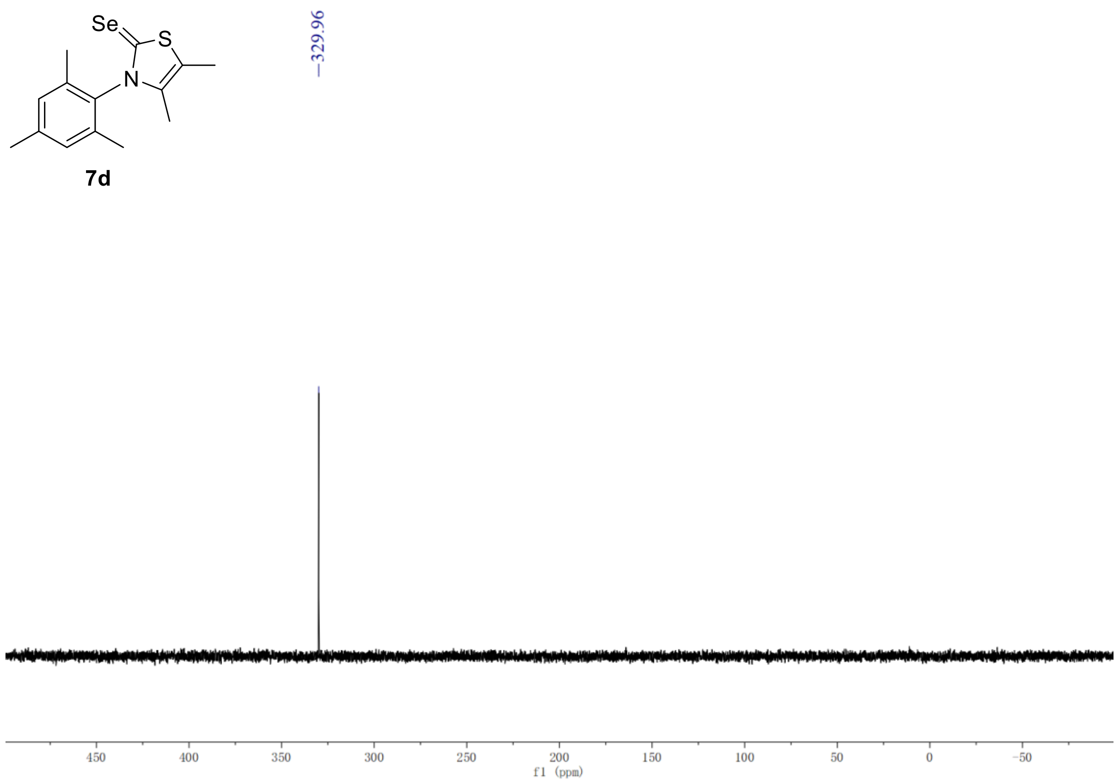


**Supplementary Figure S48.** $^{77}$Se NMR (114 MHz, $CDCl_3$) Spectrum of Compound **7d**

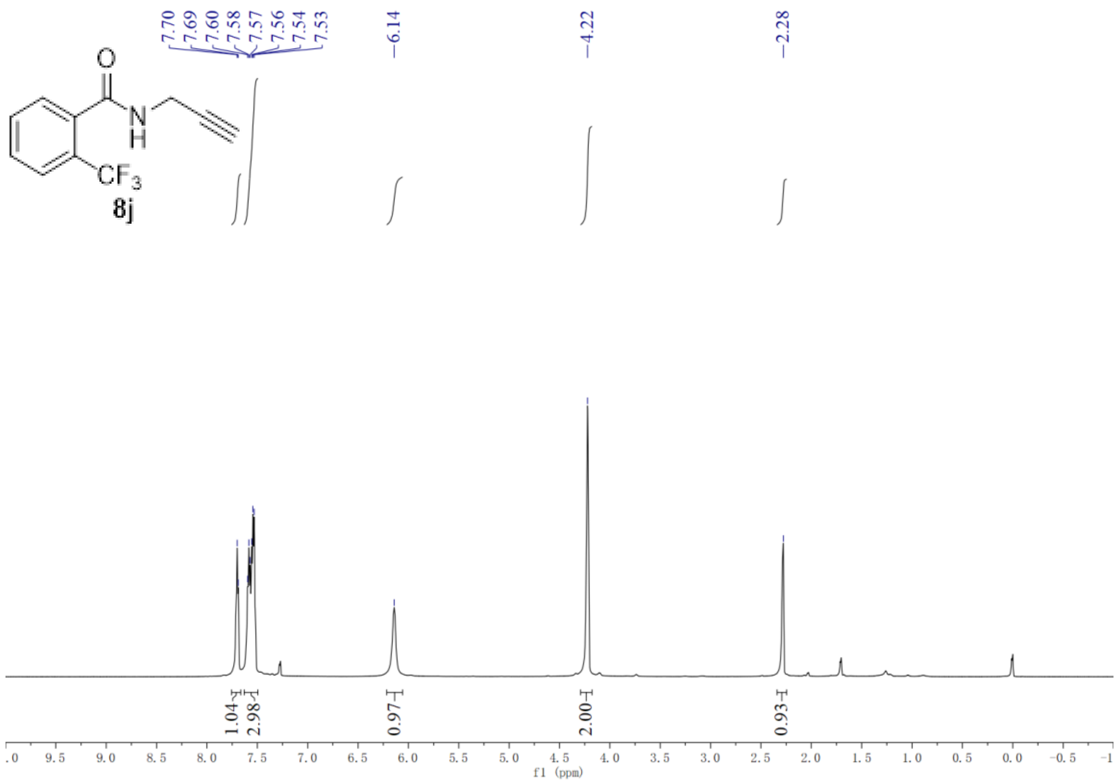


**Supplementary Figure S49.** $^{1}$H NMR (600 MHz, $CDCl_3$) Spectrum of Compound **8j**

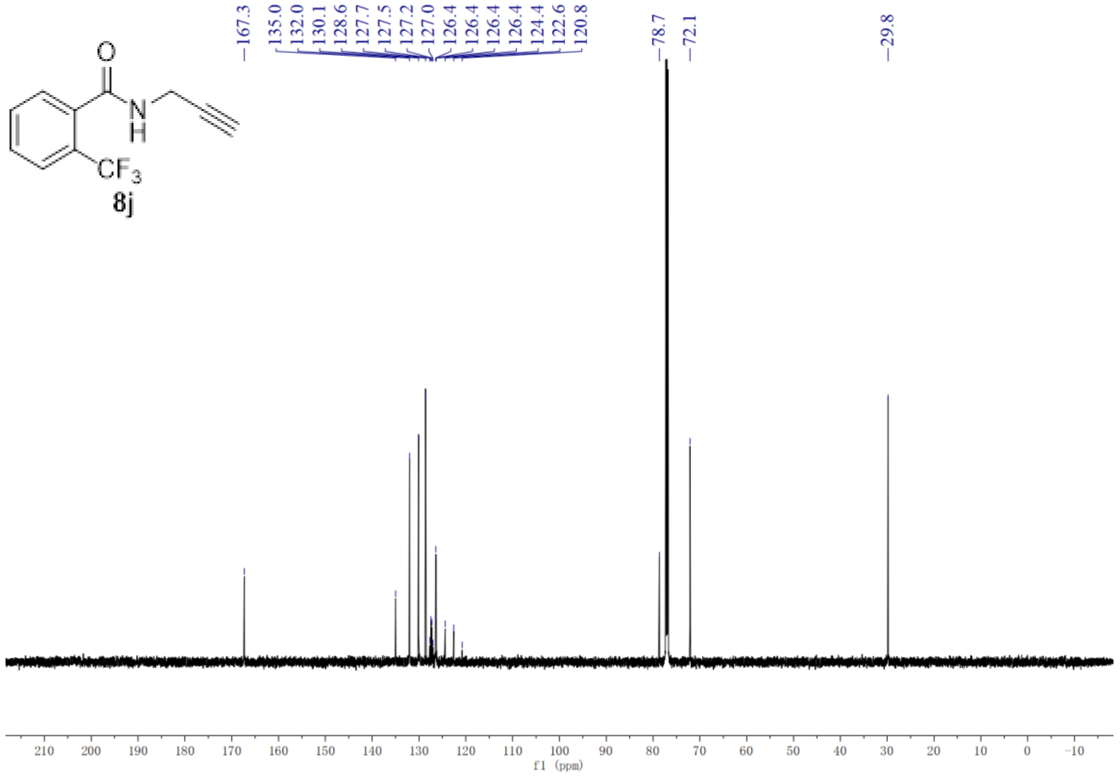


**Supplementary Figure S50.** $^{13}$C NMR (150 MHz, $CDCl_3$) Spectrum of Compound **8j**

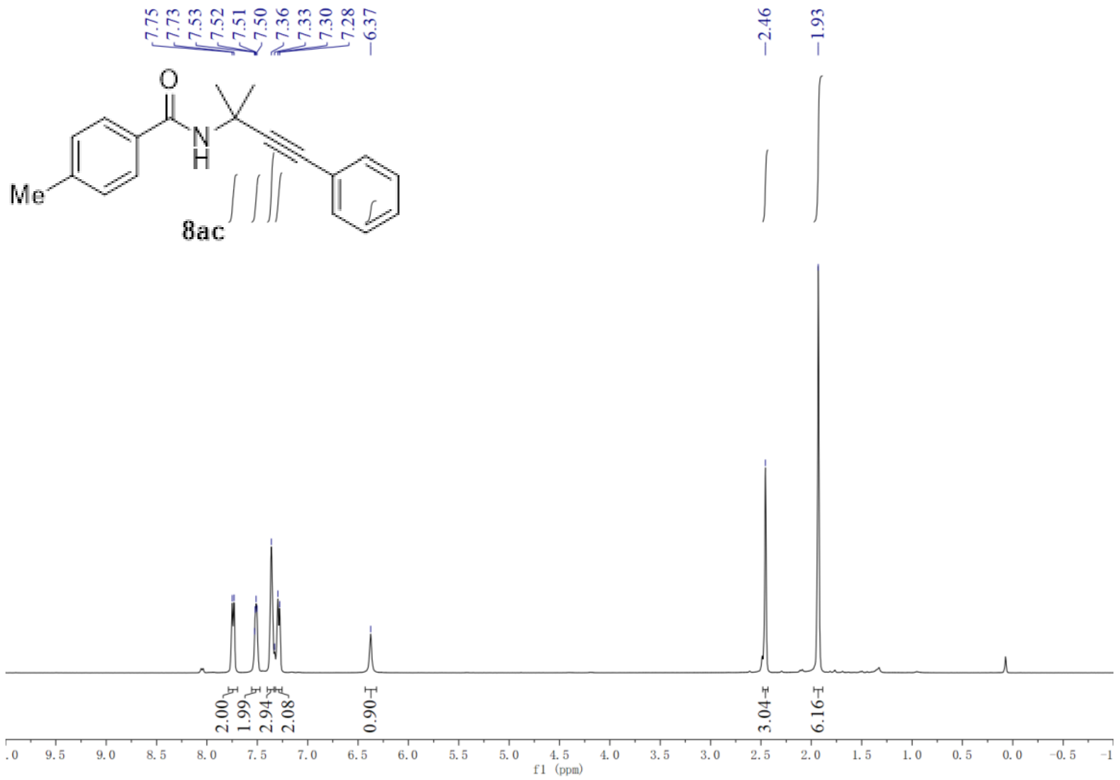


**Supplementary Figure S51.** $^{1}H$ NMR (400 MHz, $CDCl_3$) Spectrum of Compound **8ac**

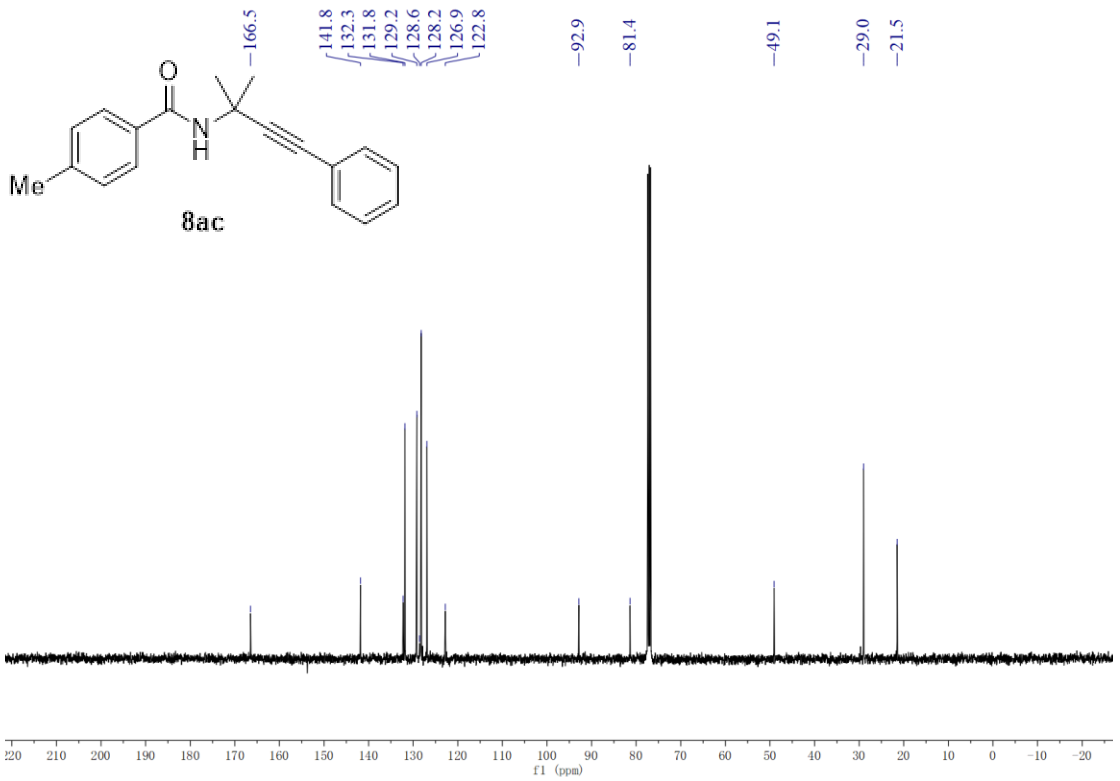


**Supplementary Figure S52.** $^{13}C$ NMR (100 MHz, $CDCl_3$) Spectrum of Compound **8ac**

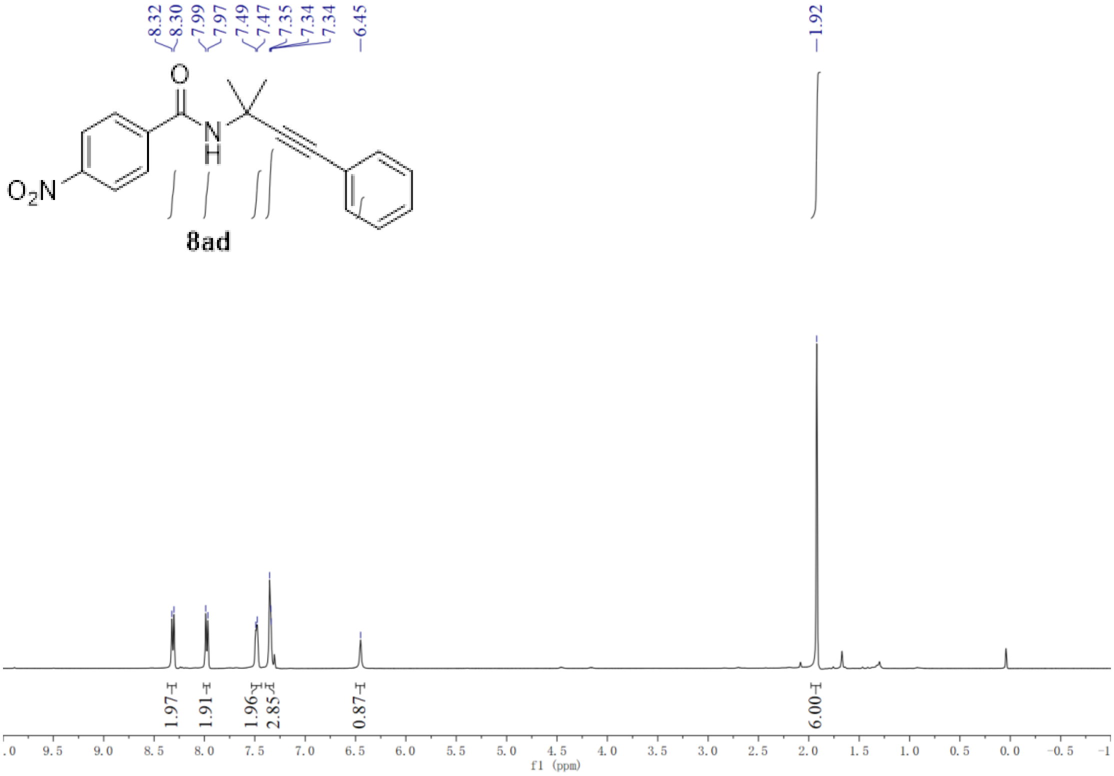


**Supplementary Figure S53.** $^{1}$H NMR (400 MHz, $CDCl_3$) Spectrum of Compound **8ad**

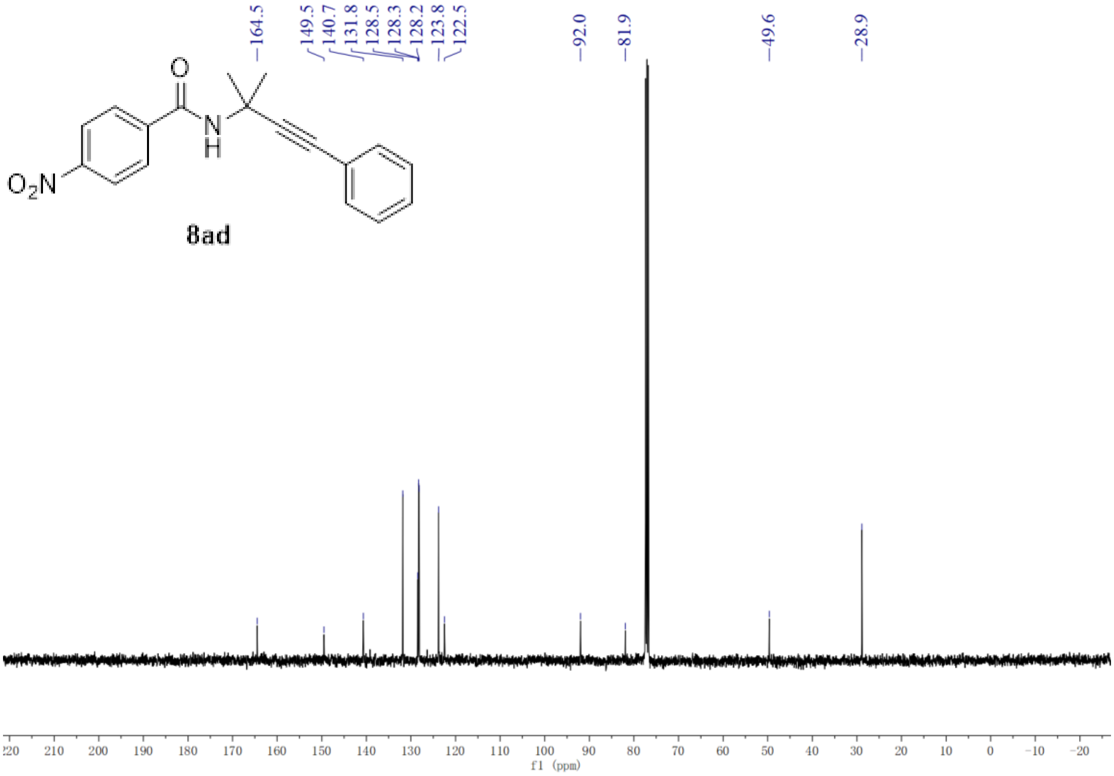


**Supplementary Figure S54.** $^{13}$C NMR (100 MHz, $CDCl_3$) Spectrum of Compound **8ad**

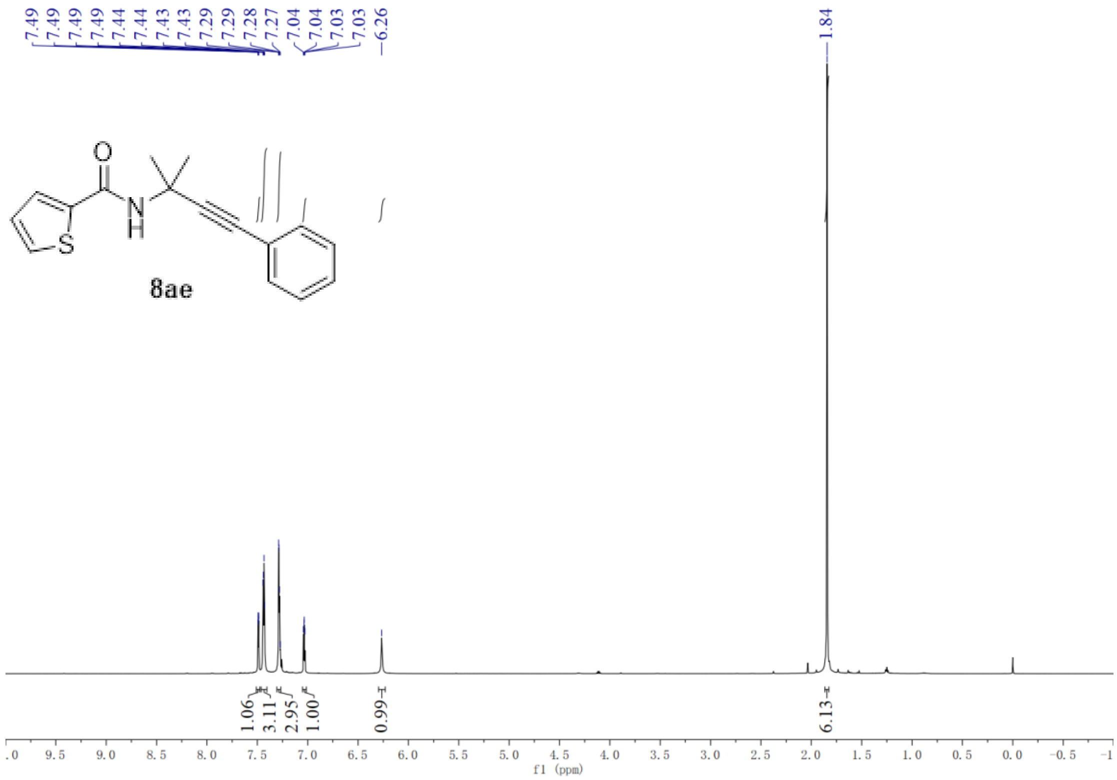


**Supplementary Figure S55.** $^{1}H$ NMR (600 MHz, $CDCl_3$) Spectrum of Compound **8ae**

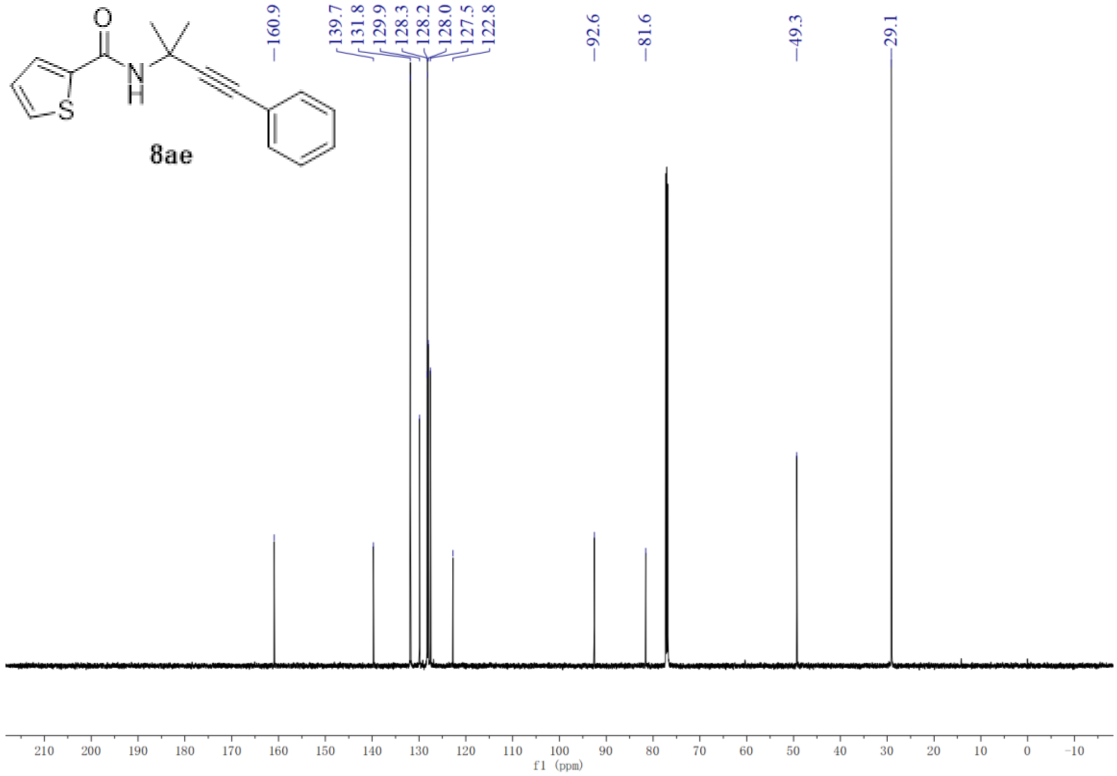


**Supplementary Figure S56.** $^{13}C$ NMR (150 MHz, $CDCl_3$) Spectrum of Compound **8ae**

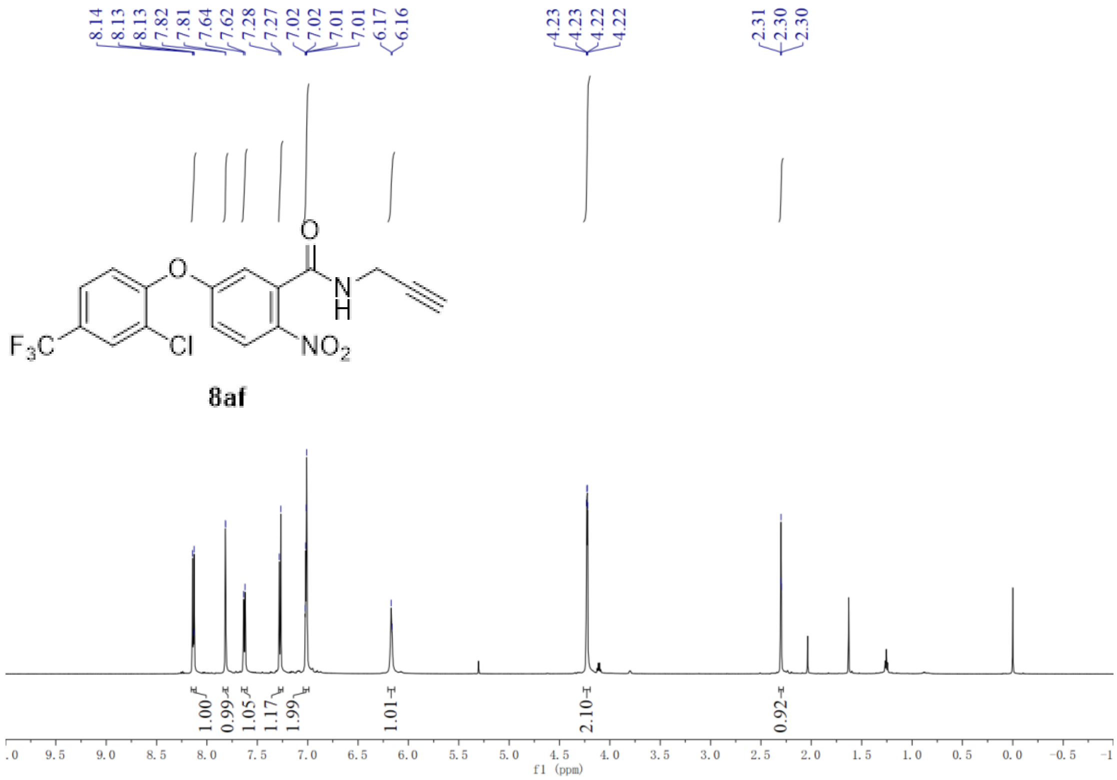


**Supplementary Figure S57.** $^{1}$H NMR (600 MHz, $CDCl_3$) Spectrum of Compound **8af**

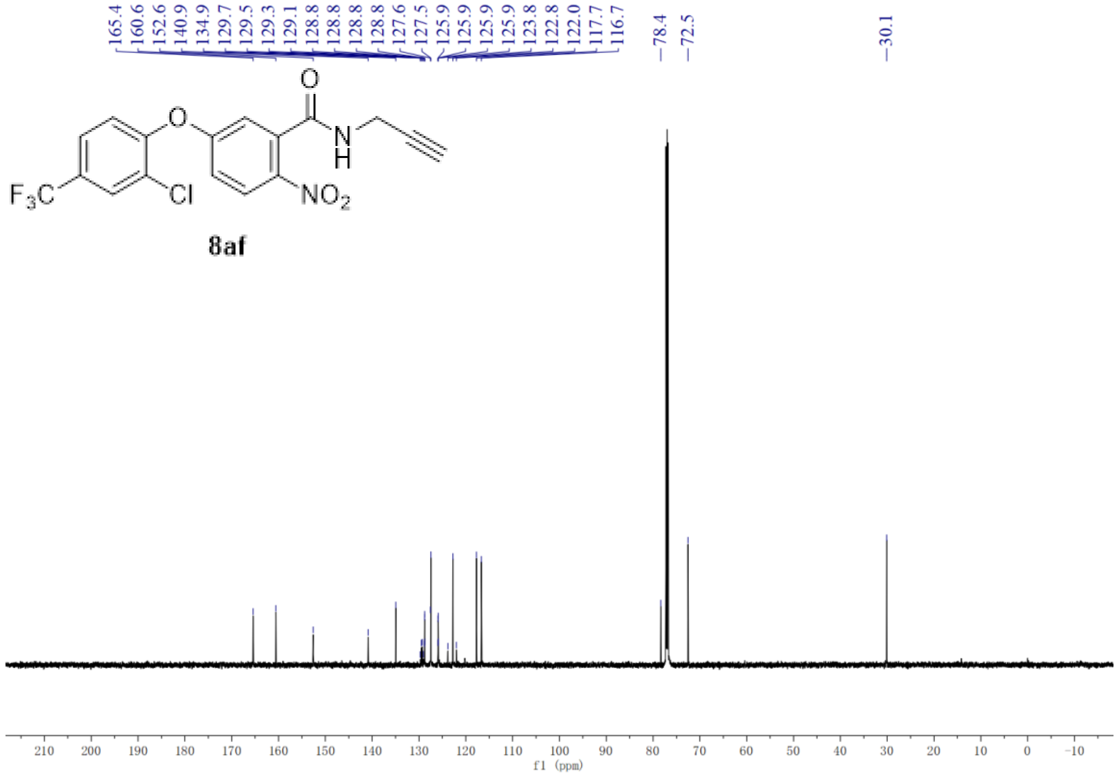


**Supplementary Figure S58.** $^{13}$C NMR (150 MHz, $CDCl_3$) Spectrum of Compound **8af**

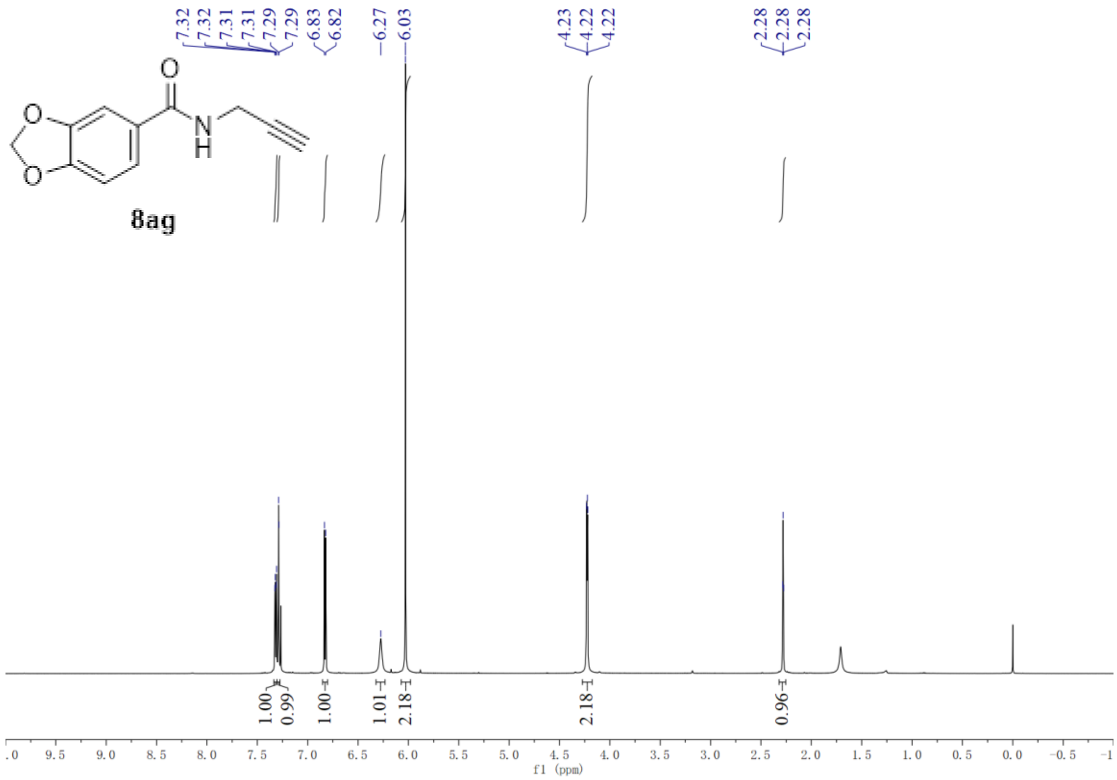


**Supplementary Figure S59.** $^{1}H$ NMR (600 MHz, $CDCl_3$) Spectrum of Compound **8ag**

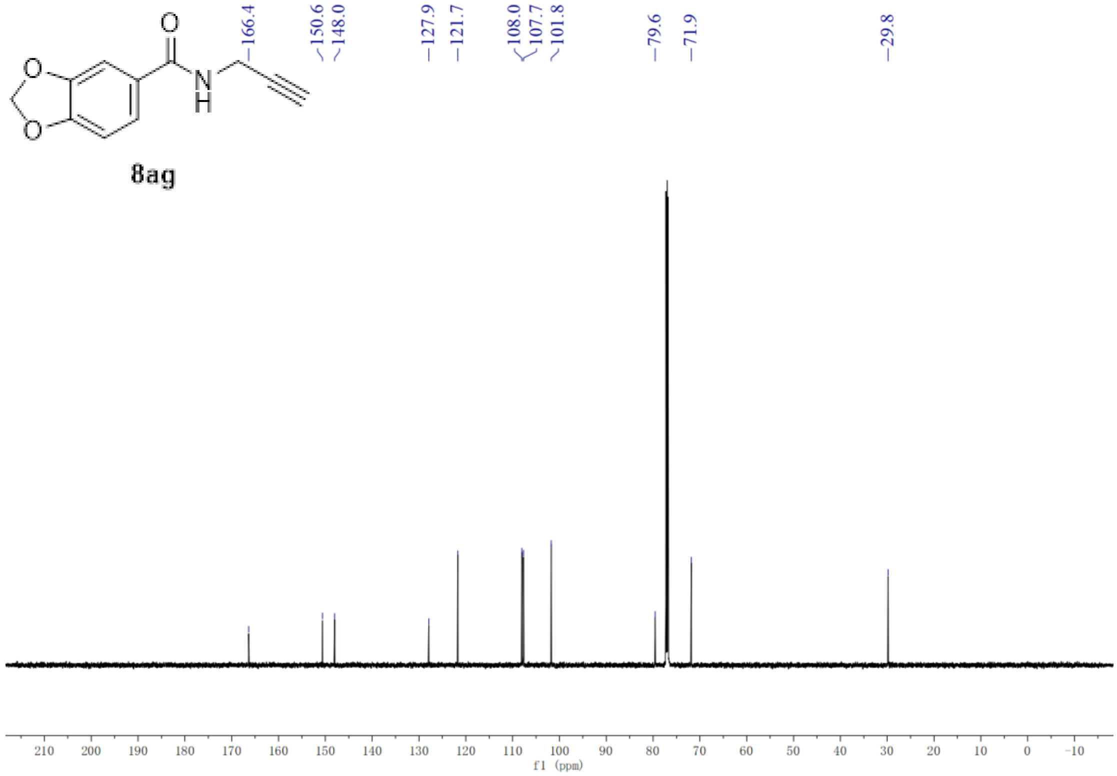


**Supplementary Figure S60.** $^{13}C$ NMR (150 MHz, $CDCl_3$) Spectrum of Compound **8ag**

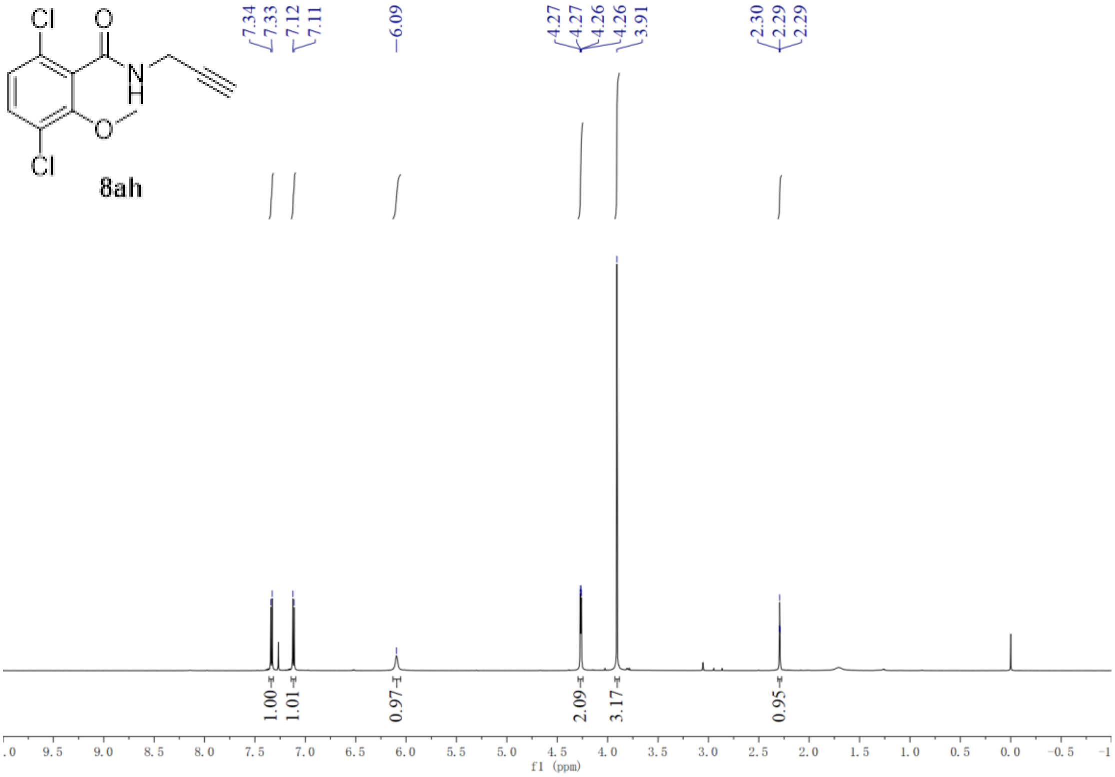


**Supplementary Figure S61.** $^{1}H$ NMR (600 MHz, $CDCl_3$) Spectrum of Compound **8ah**

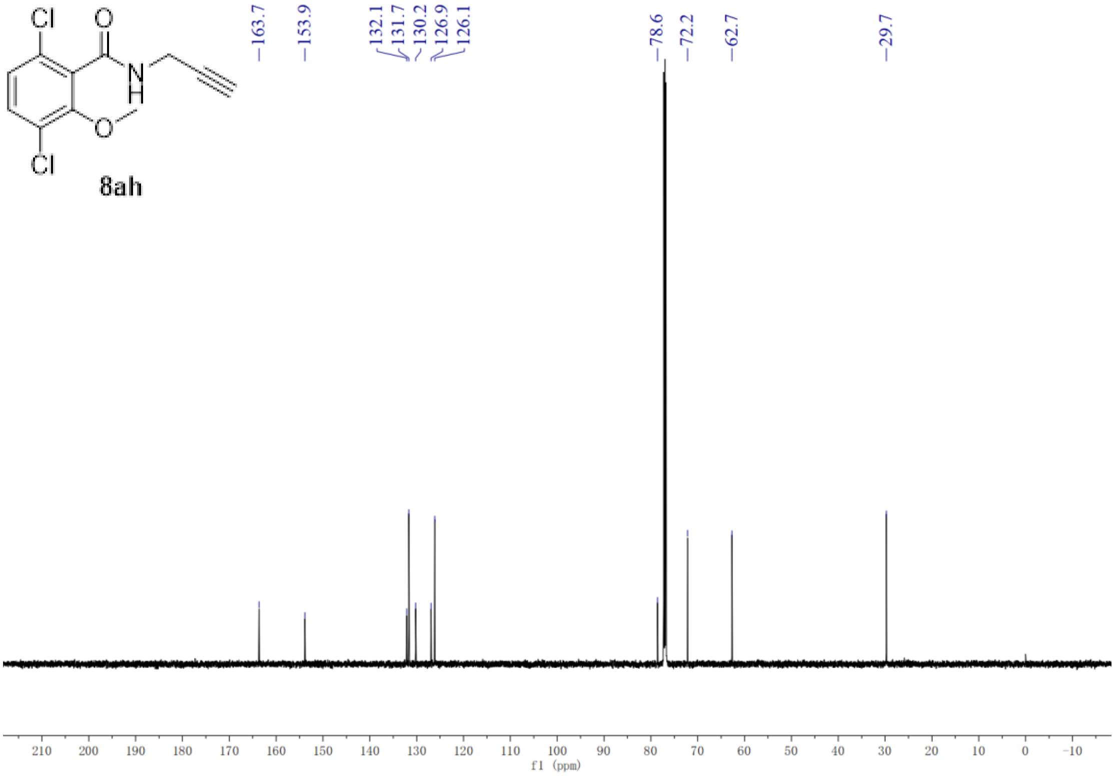


**Supplementary Figure S62.** $^{13}C$ NMR (150 MHz, $CDCl_3$) Spectrum of Compound **8ah**

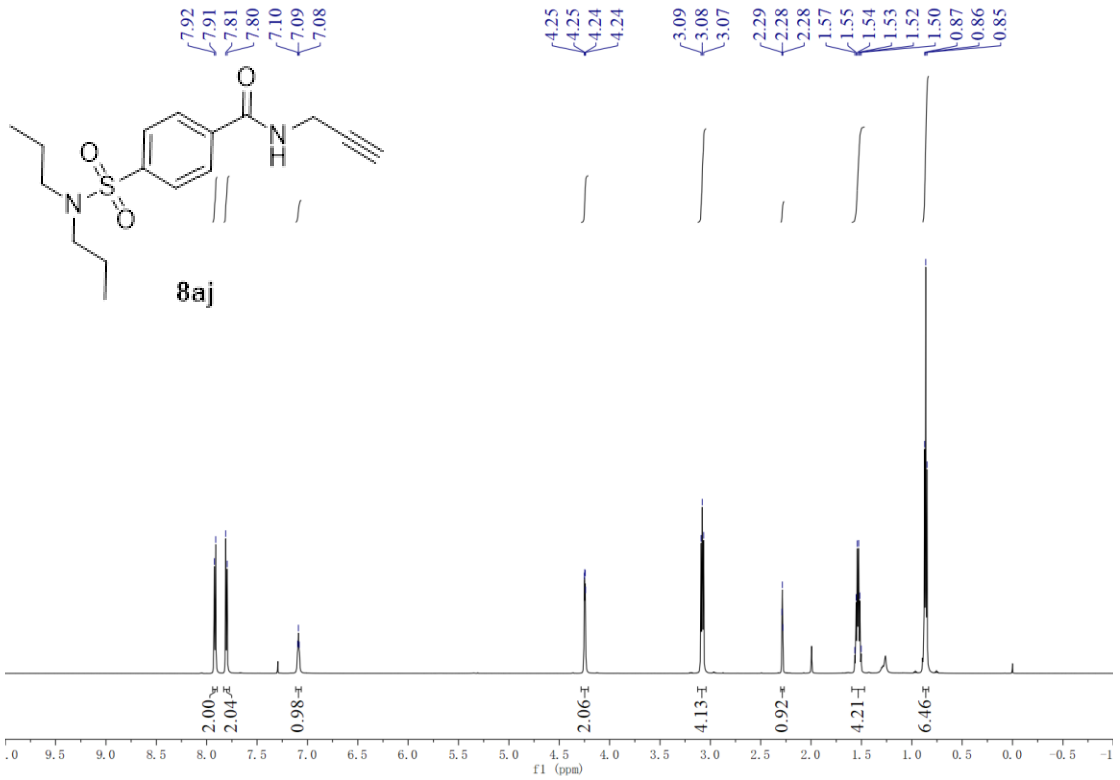


**Supplementary Figure S63.** $^{1}$H NMR (600 MHz, $CDCl_3$) Spectrum of Compound **8aj**

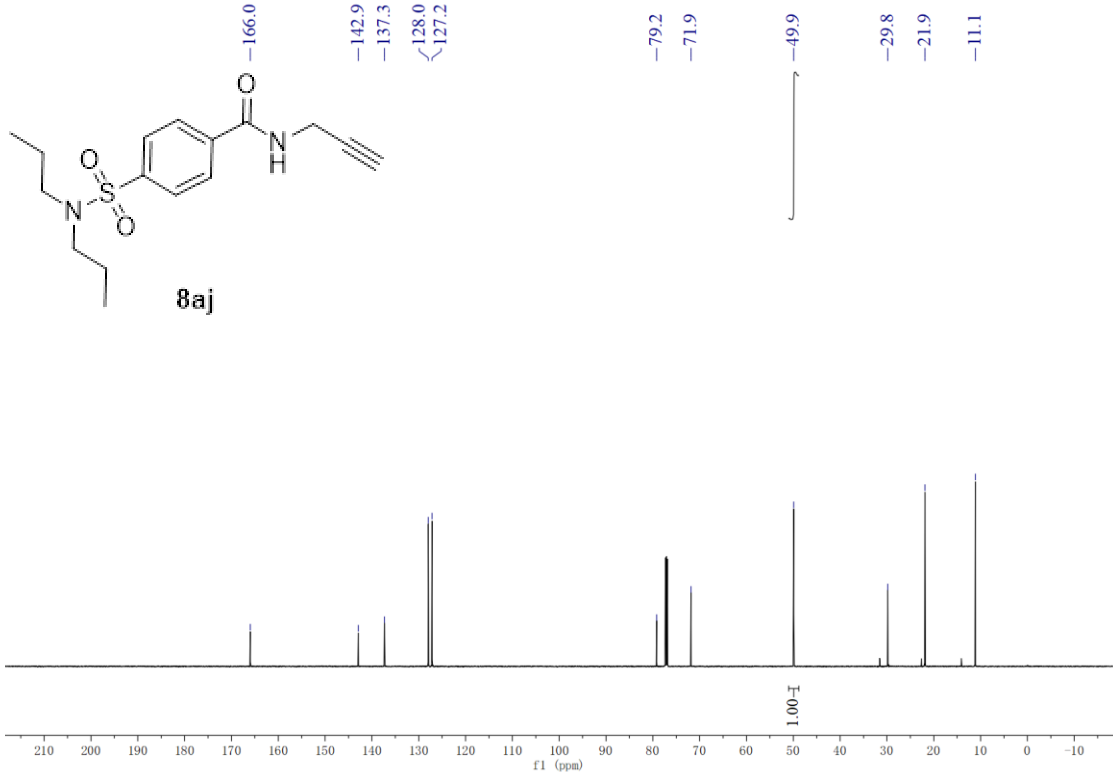


**Supplementary Figure S64.** $^{13}$C NMR (150 MHz, $CDCl_3$) Spectrum of Compound **8aj**

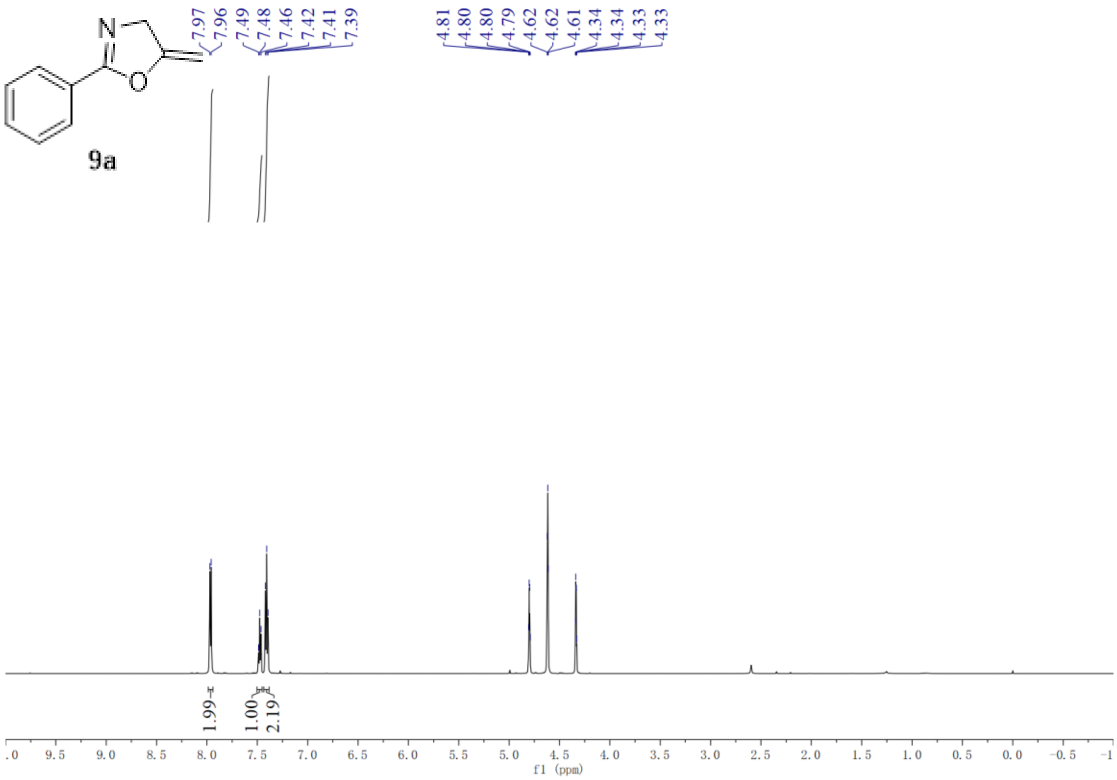

**Supplementary Figure S65.** $^{1}$H NMR (600 MHz, $CDCl_3$) Spectrum of Compound **9a**

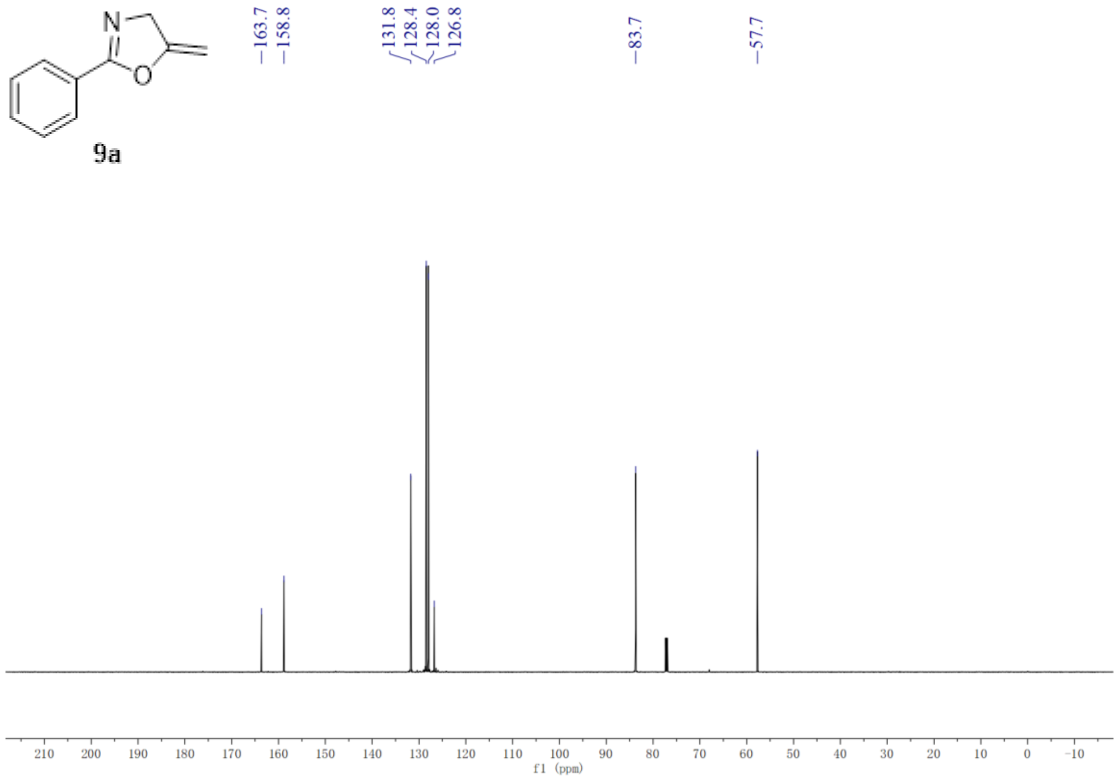

**Supplementary Figure S66.** $^{13}$C NMR (150 MHz, $CDCl_3$) Spectrum of Compound **9a**

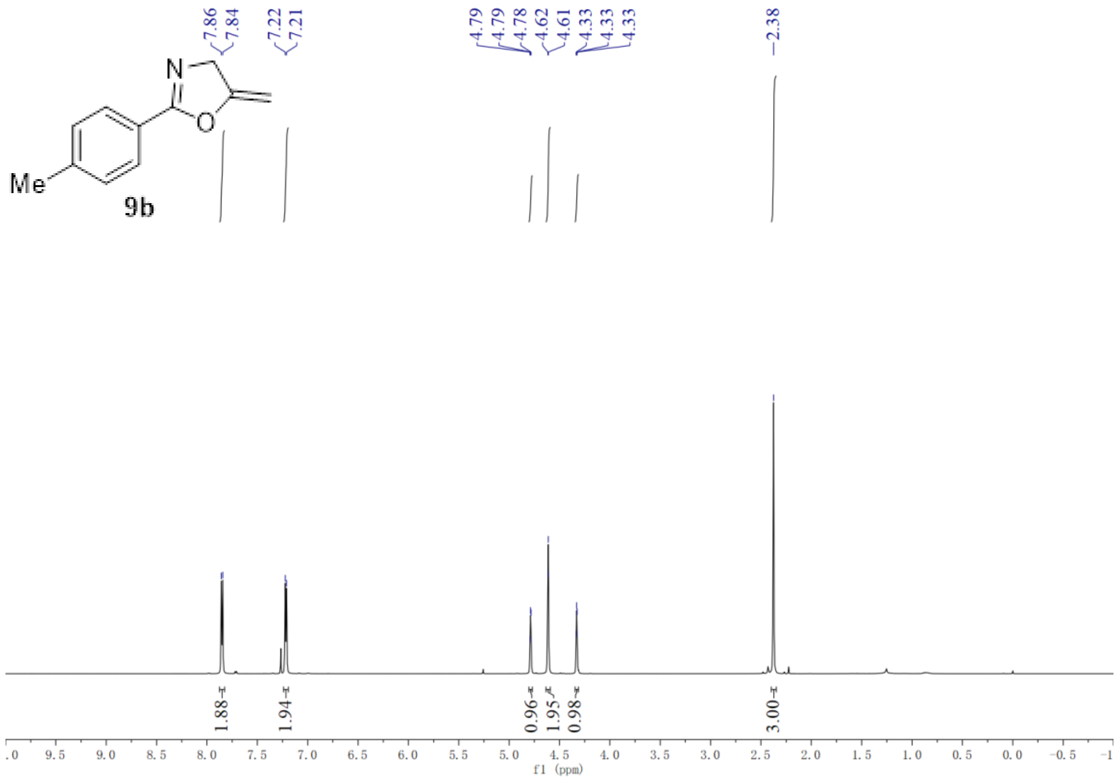

**Supplementary Figure S67.** $^{1}$H NMR (600 MHz, $CDCl_3$) Spectrum of Compound **9b**

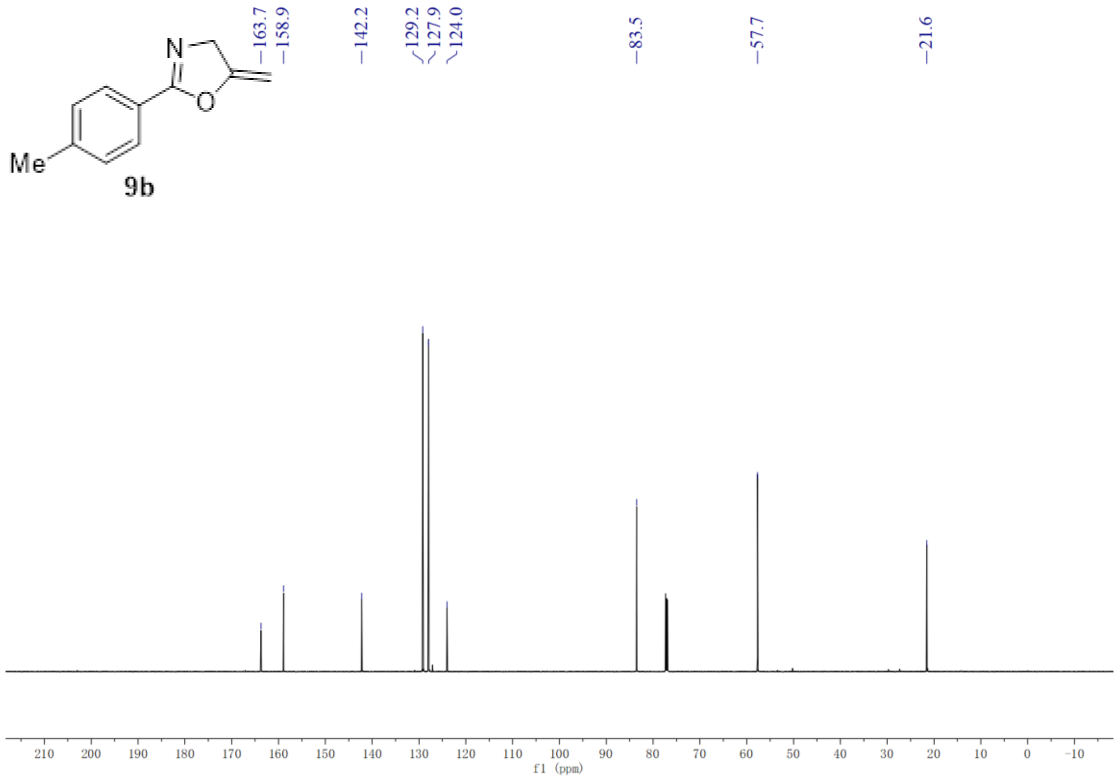

**Supplementary Figure S68.** $^{13}$C NMR (150 MHz, $CDCl_3$) Spectrum of Compound **9b**

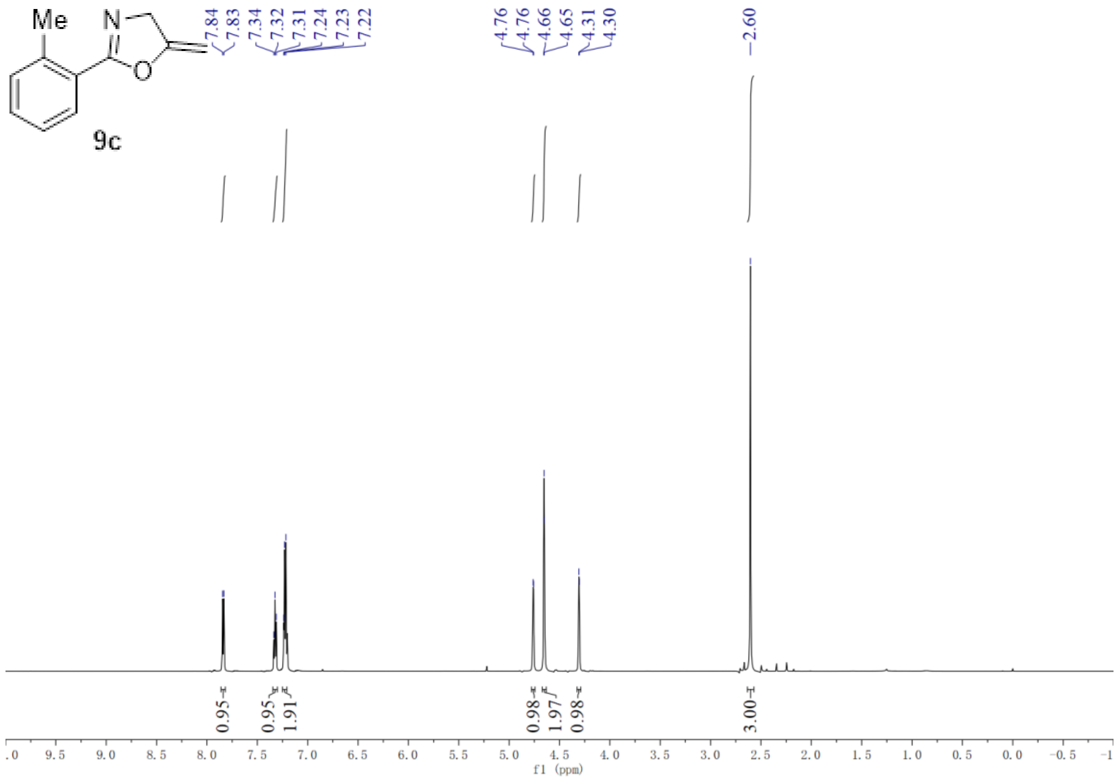


**Supplementary Figure S69.** $^{1}H$ NMR (600 MHz, $CDCl_3$) Spectrum of Compound **9c**

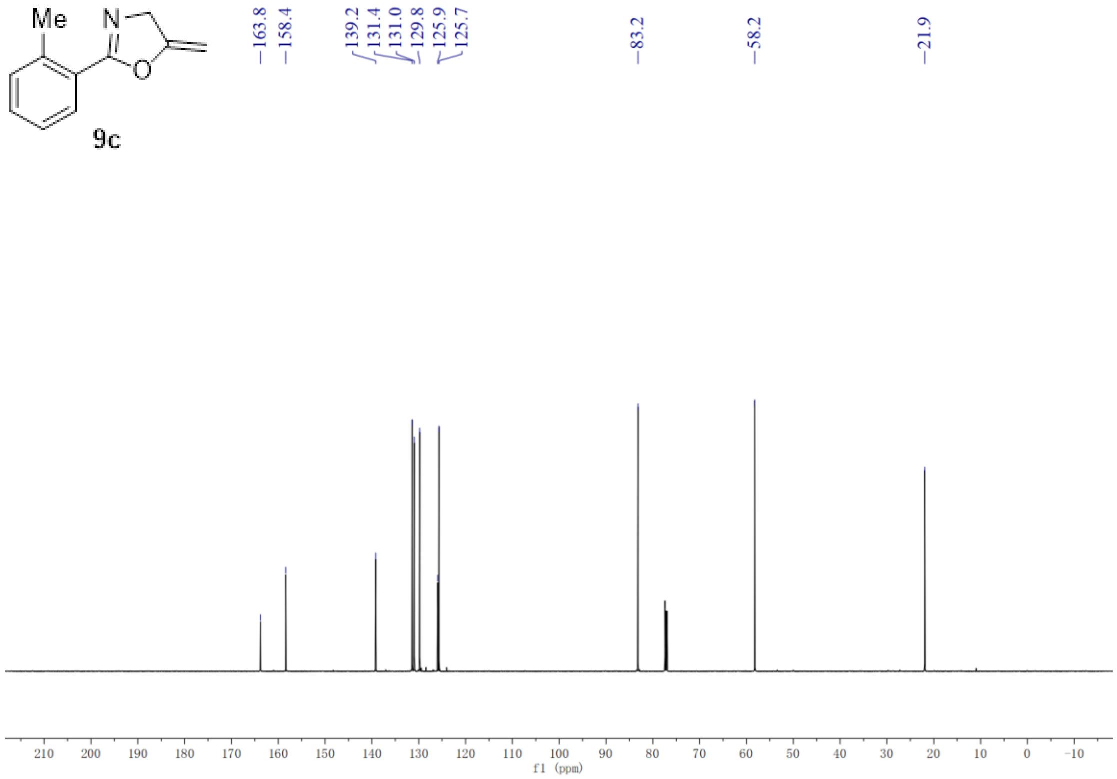


**Supplementary Figure S70.** $^{13}C$ NMR (150 MHz, $CDCl_3$) Spectrum of Compound **9c**

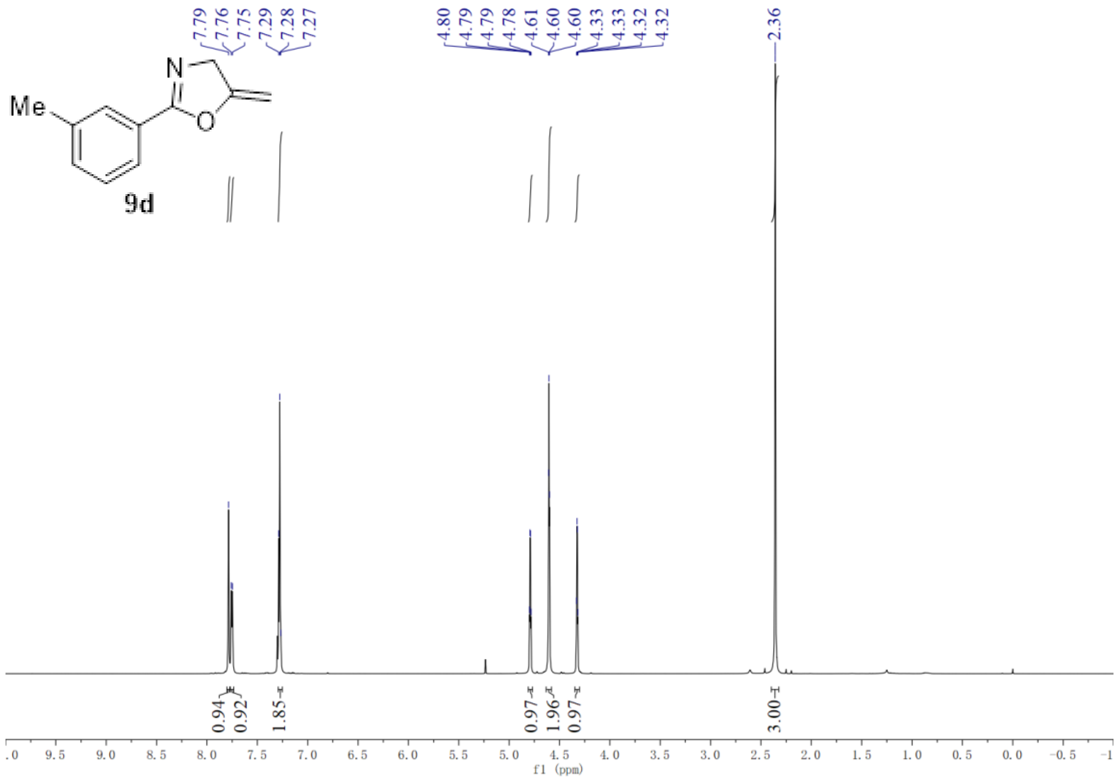


**Supplementary Figure S71.** $^{1}$H NMR (600 MHz, $CDCl_3$) Spectrum of Compound **9d**

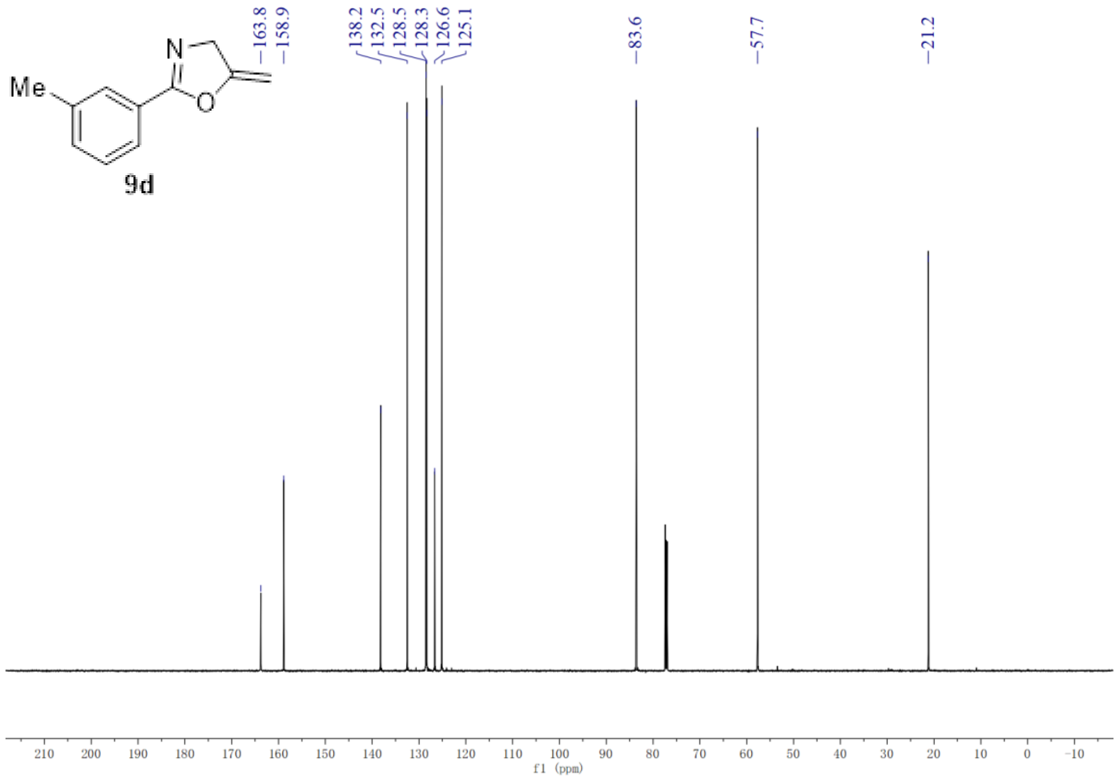


**Supplementary Figure S72.** $^{13}$C NMR (150 MHz, $CDCl_3$) Spectrum of Compound **9d**

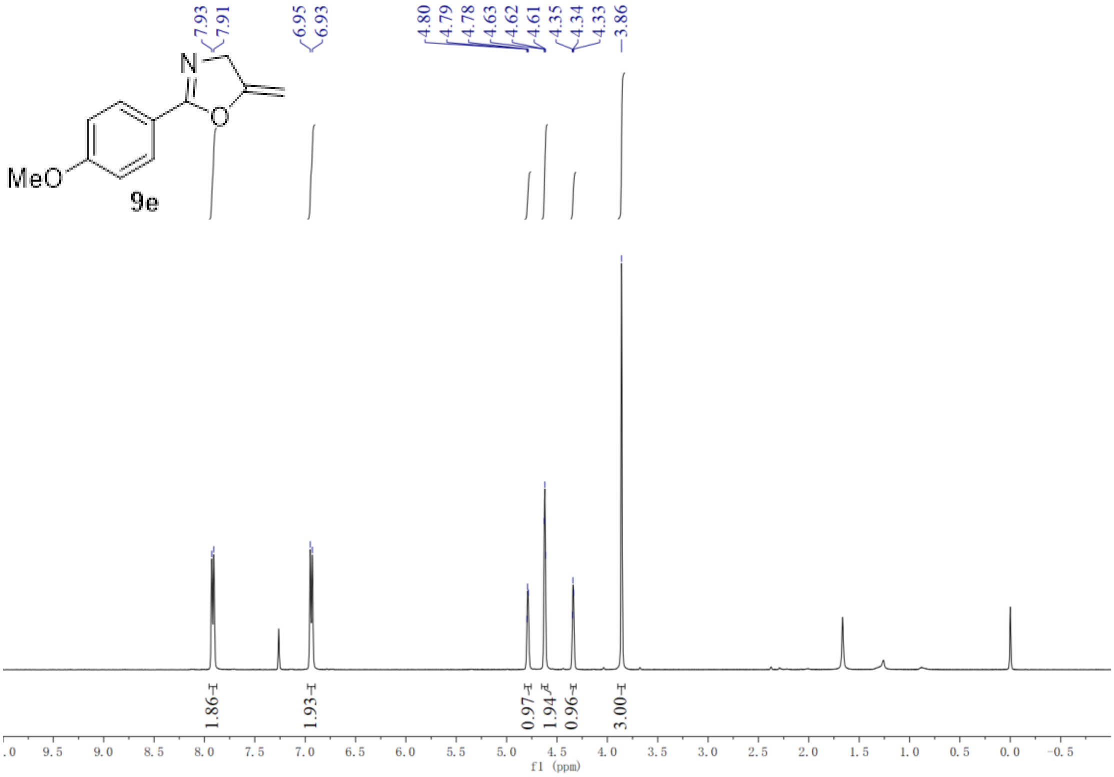


**Supplementary Figure S73.** $^{1}$H NMR (400 MHz, $CDCl_3$) Spectrum of Compound **9e**

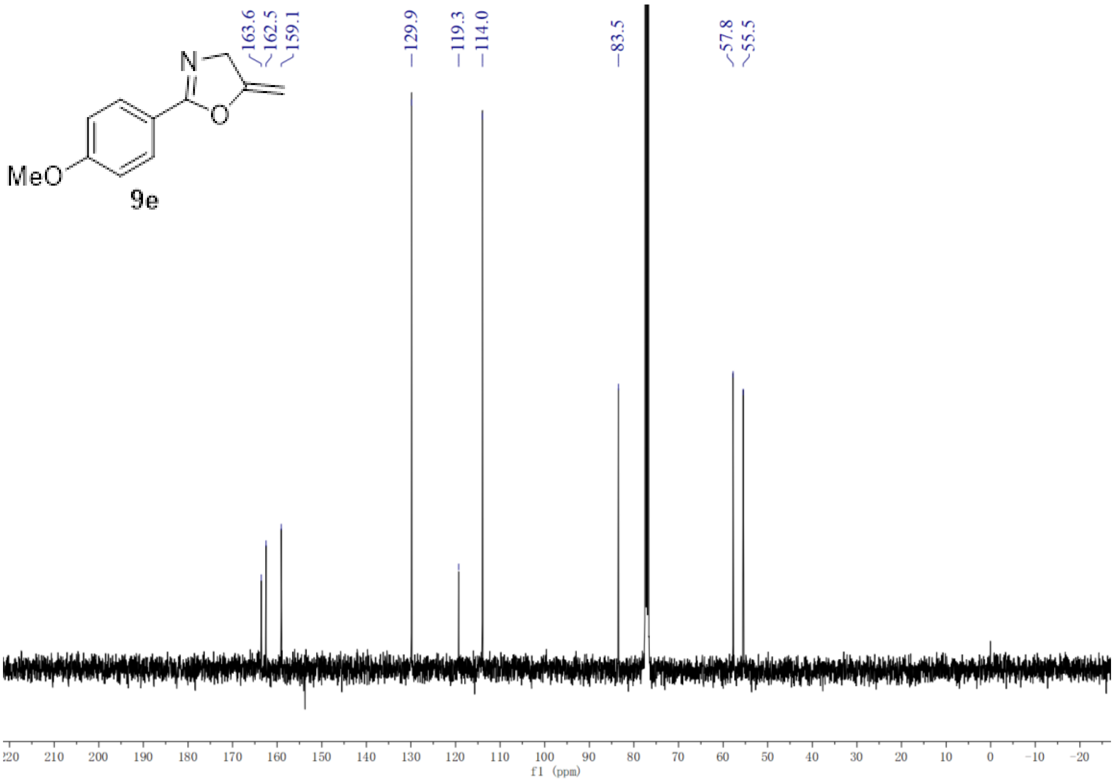


**Supplementary Figure S74.** $^{13}$C NMR (100 MHz, $CDCl_3$) Spectrum of Compound **9e**

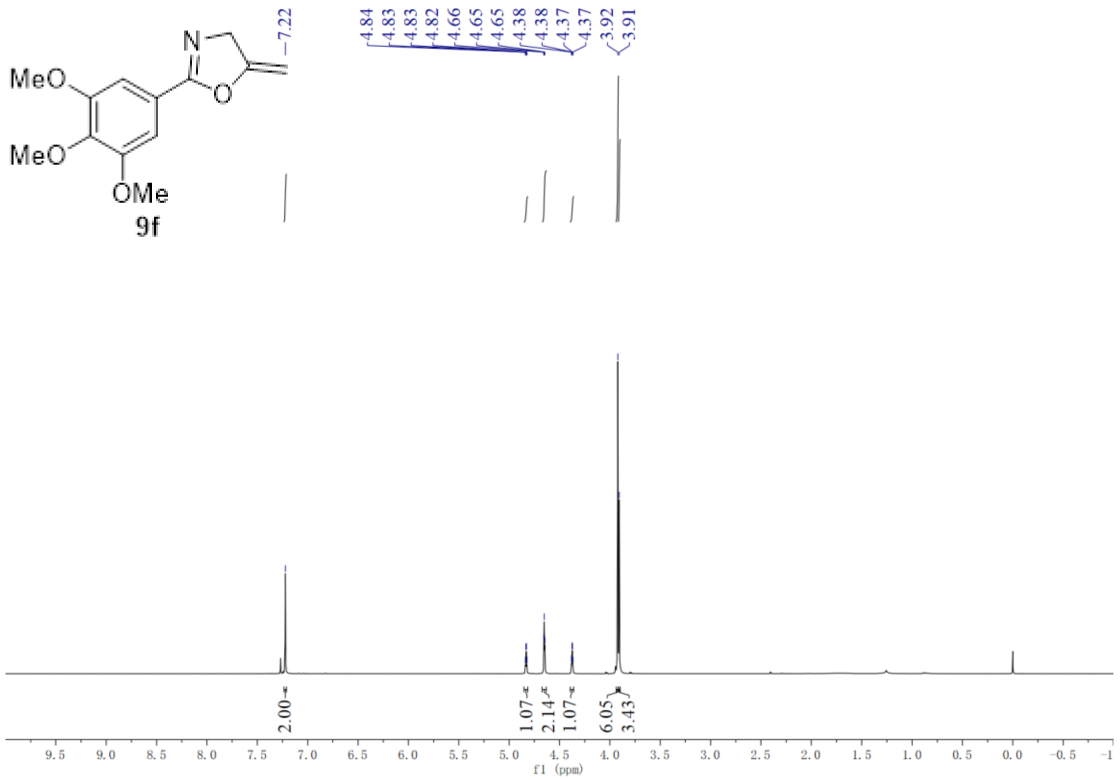


**Supplementary Figure S75.** $^{1}H$ NMR (600 MHz, $CDCl_3$) Spectrum of Compound **9f**

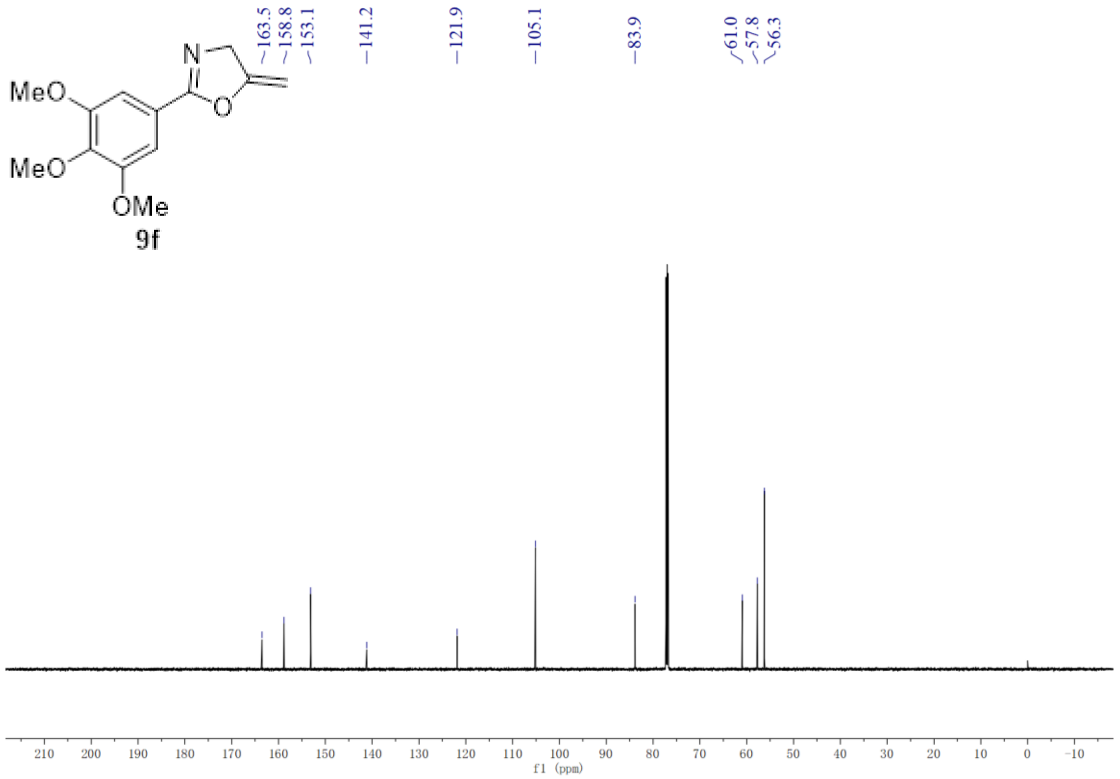


**Supplementary Figure S76.** $^{13}C$ NMR (150 MHz, $CDCl_3$) Spectrum of Compound **9f**

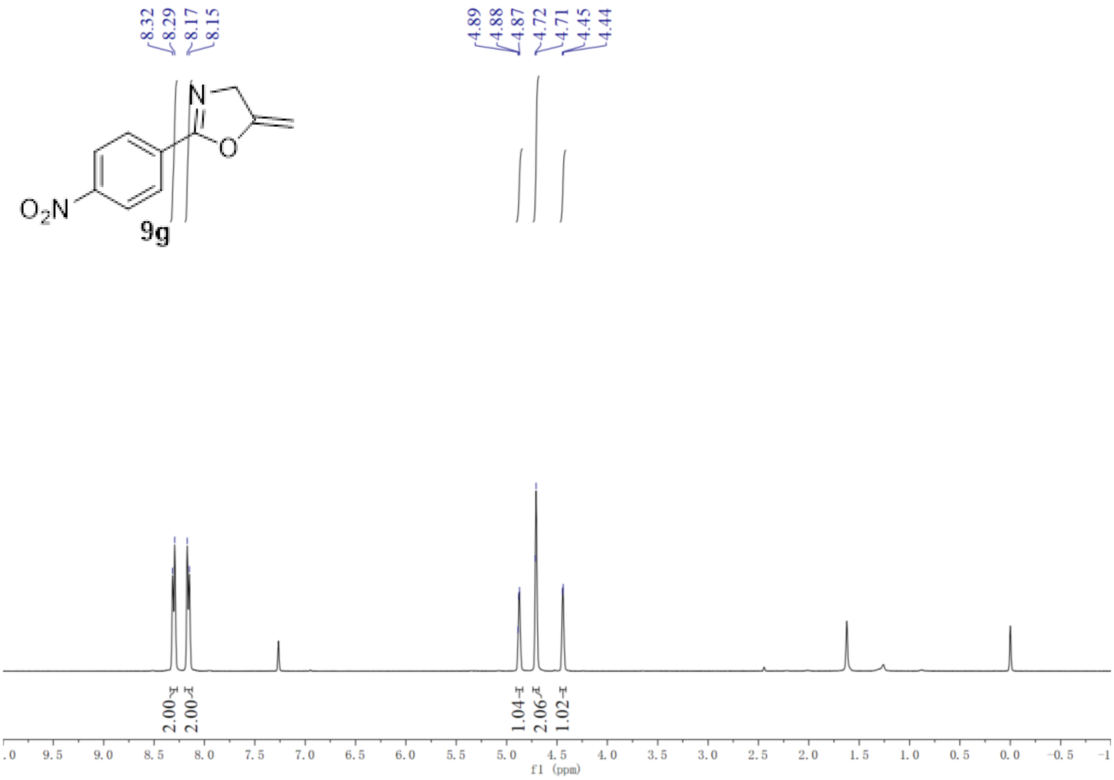


**Supplementary Figure S77.** $^{1}$H NMR (400 MHz, $CDCl_3$) Spectrum of Compound **9g**

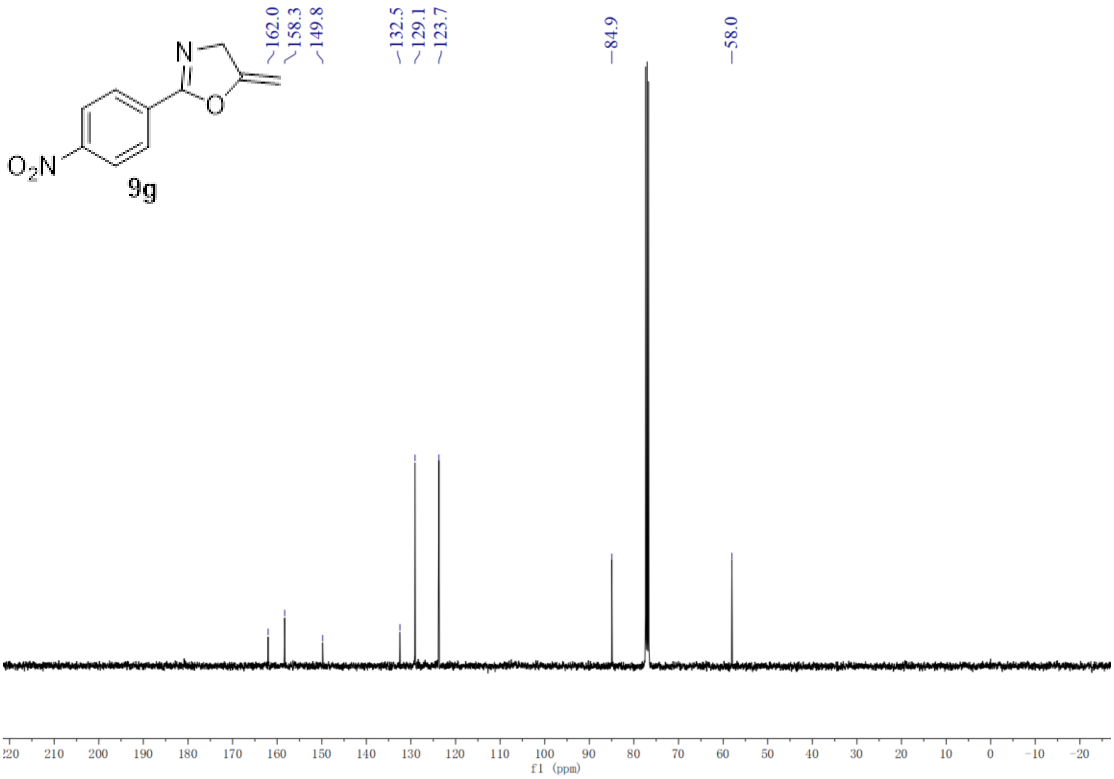


**Supplementary Figure S78.** $^{13}$C NMR (100 MHz, $CDCl_3$) Spectrum of Compound **9g**

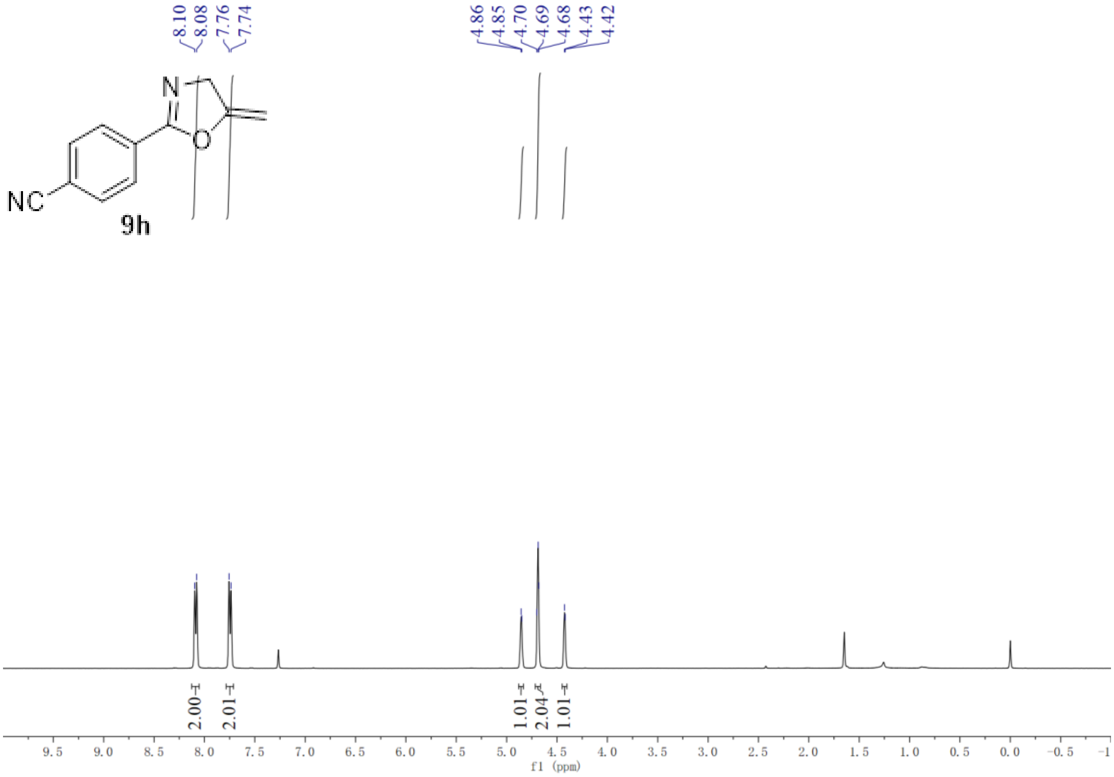

**Supplementary Figure S79.** $^{1}H$ NMR (400 MHz, $CDCl_3$) Spectrum of Compound **9h**

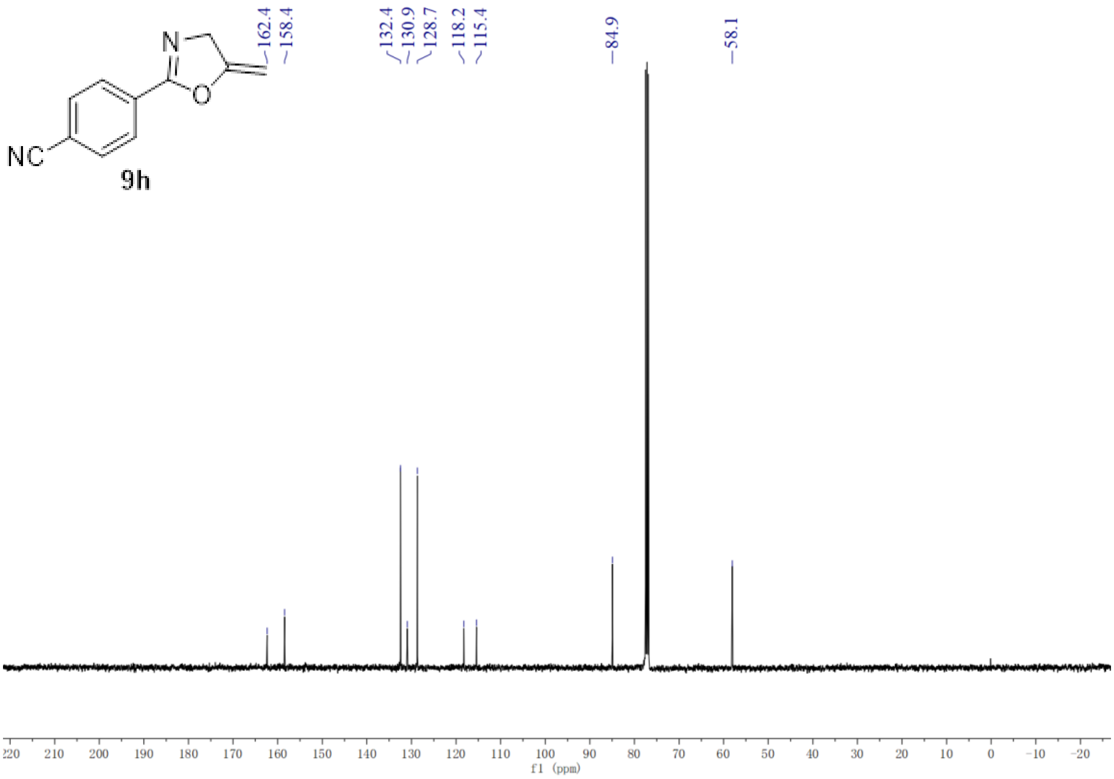

**Supplementary Figure S80.** $^{13}C$ NMR (100 MHz, $CDCl_3$) Spectrum of Compound **9h**

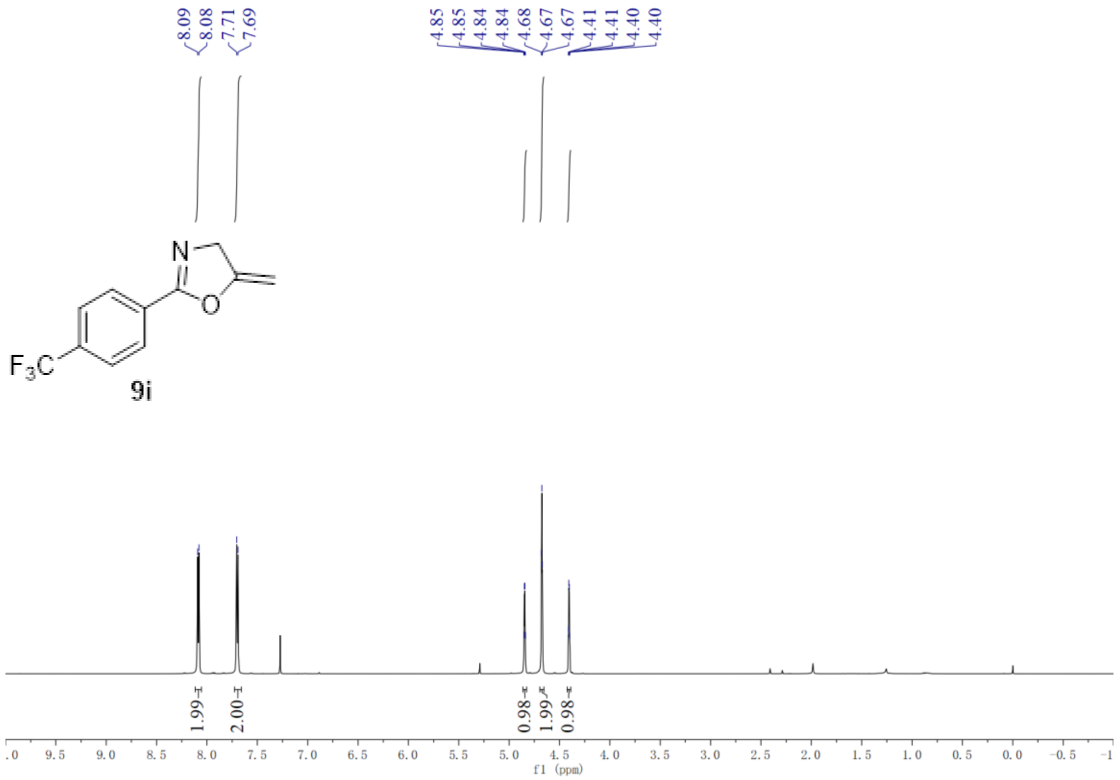

**Supplementary Figure S81.** $^{1}H$ NMR (600 MHz, $CDCl_3$) Spectrum of Compound **9i**

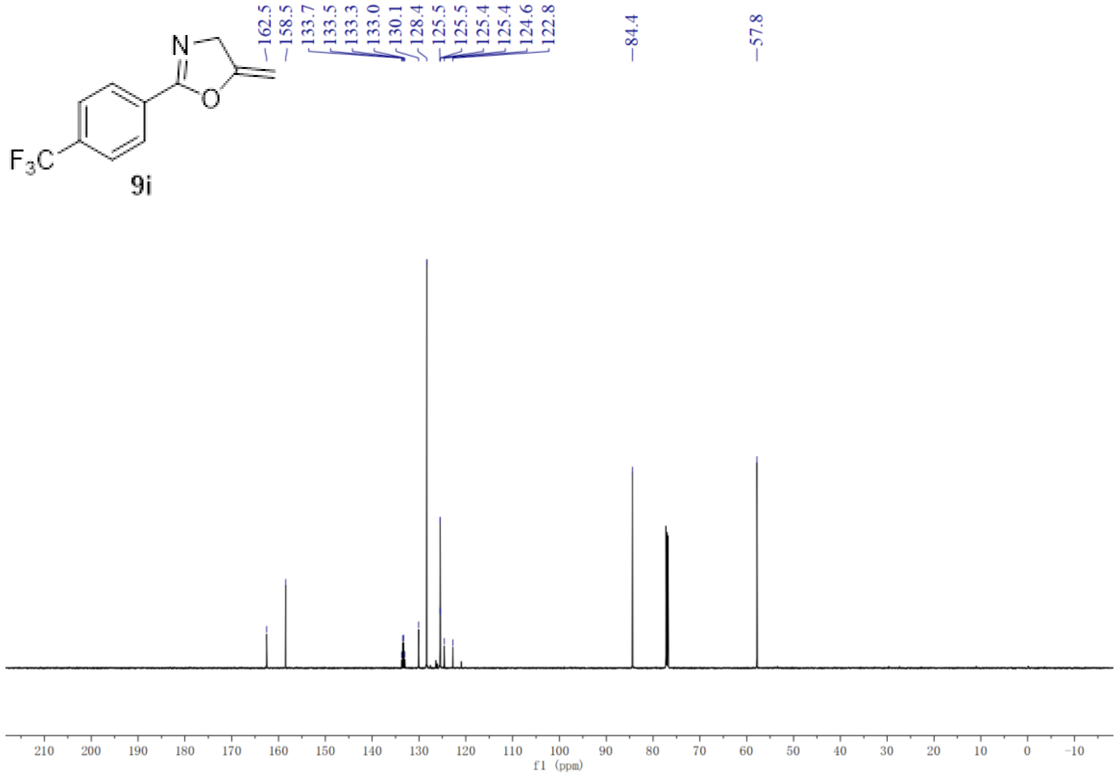

**Supplementary Figure S82.** $^{13}C$ NMR (150 MHz, $CDCl_3$) Spectrum of Compound **9i**

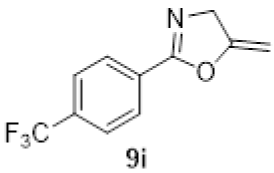

9i

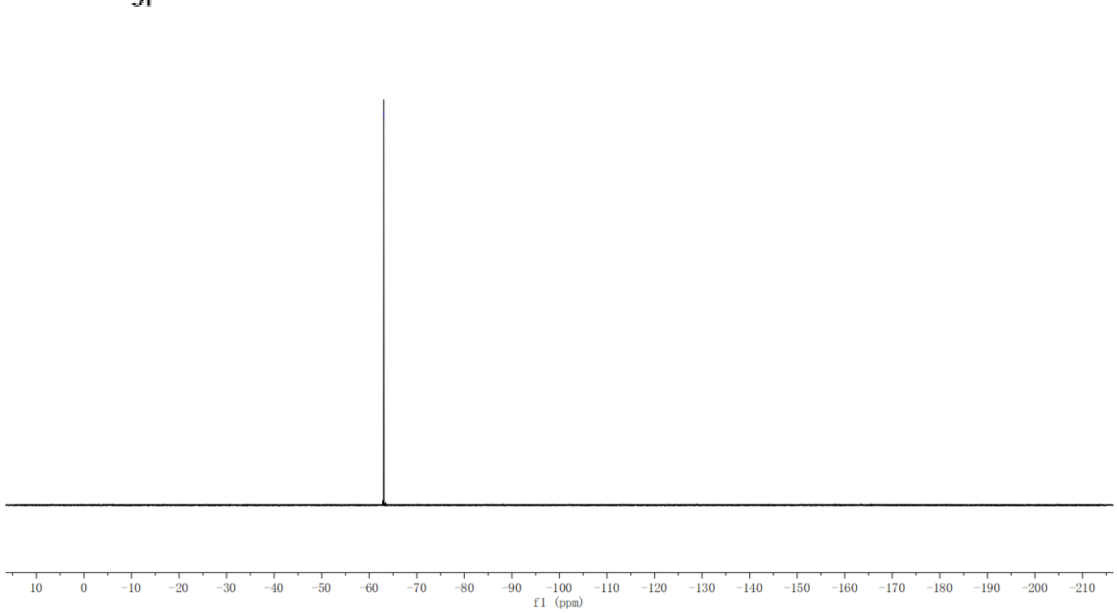


**Supplementary Figure S83.** $^{19}F$ NMR (565 MHz, $CDCl_3$) Spectrum of Compound **9i**

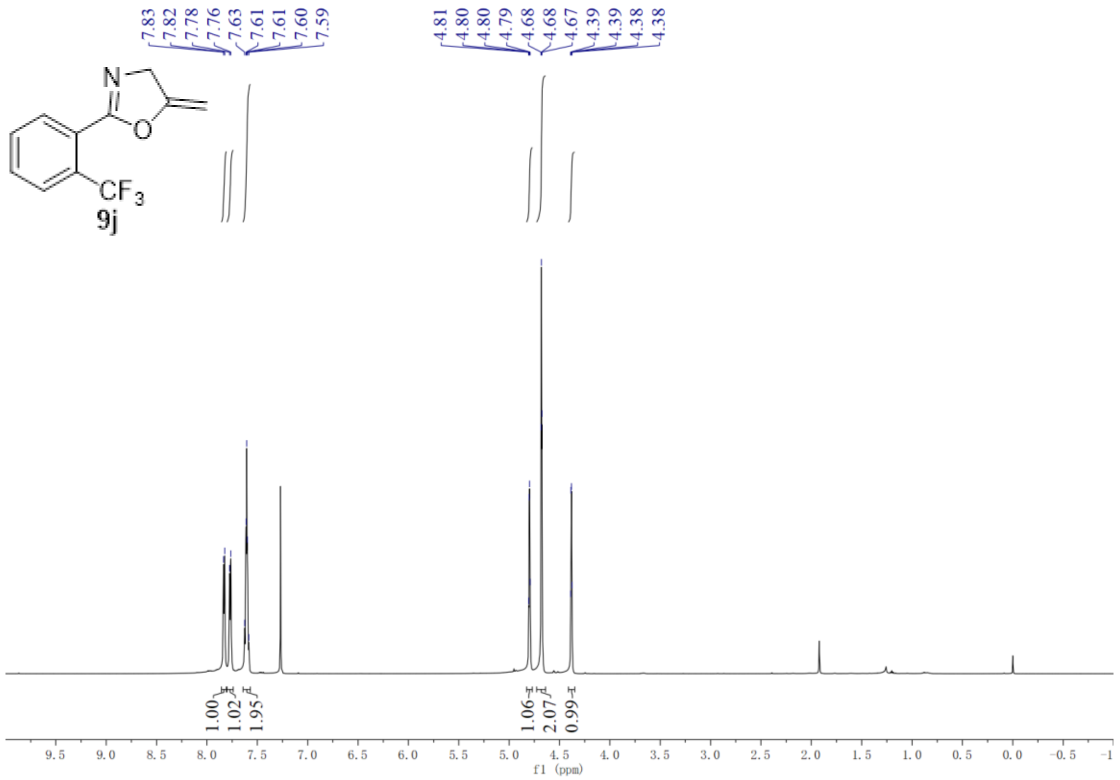


**Supplementary Figure S84.** $^{1}$H NMR (600 MHz, $CDCl_3$) Spectrum of Compound **9j**

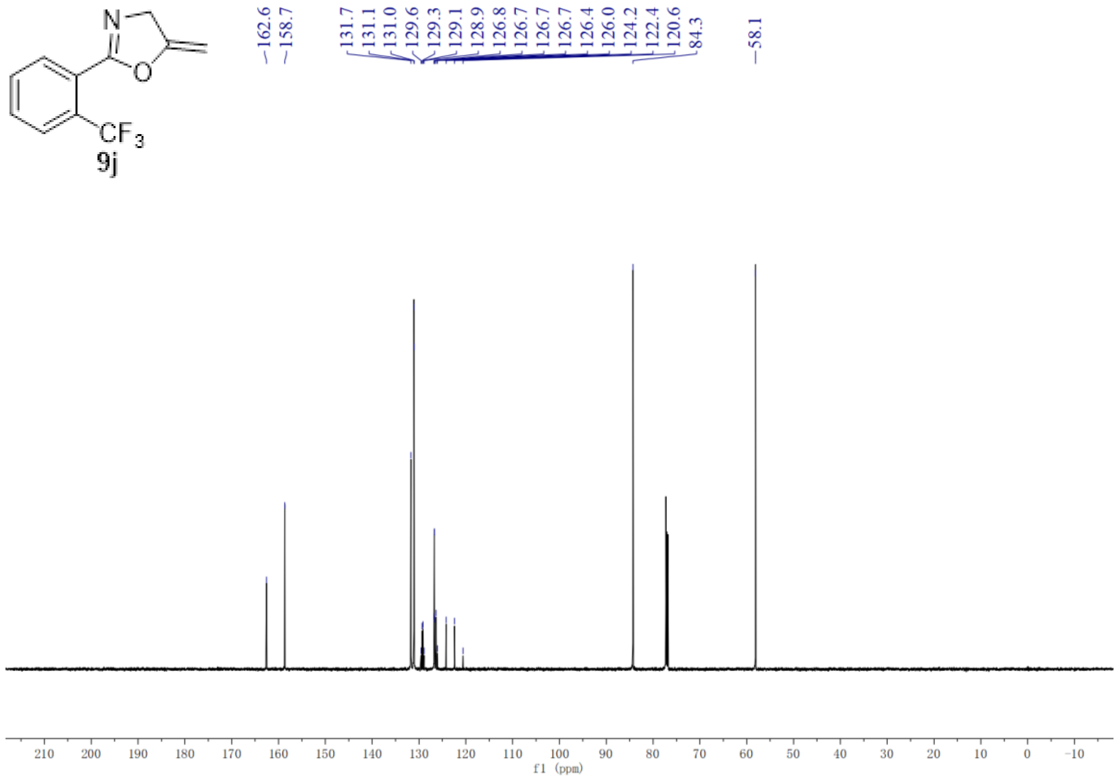


**Supplementary Figure S85.** $^{13}$C NMR (150 MHz, $CDCl_3$) Spectrum of Compound **9j**

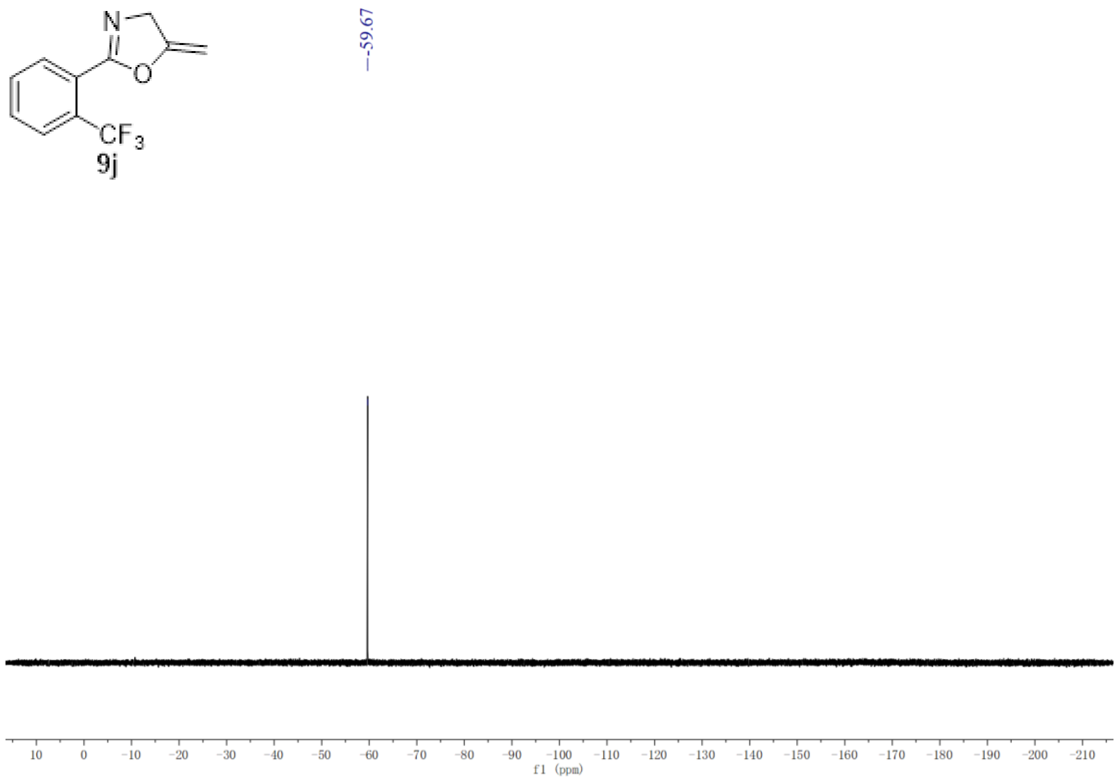

**Supplementary Figure S86.** $^{19}F$ NMR (565 MHz, $CDCl_3$) Spectrum of Compound **9j**

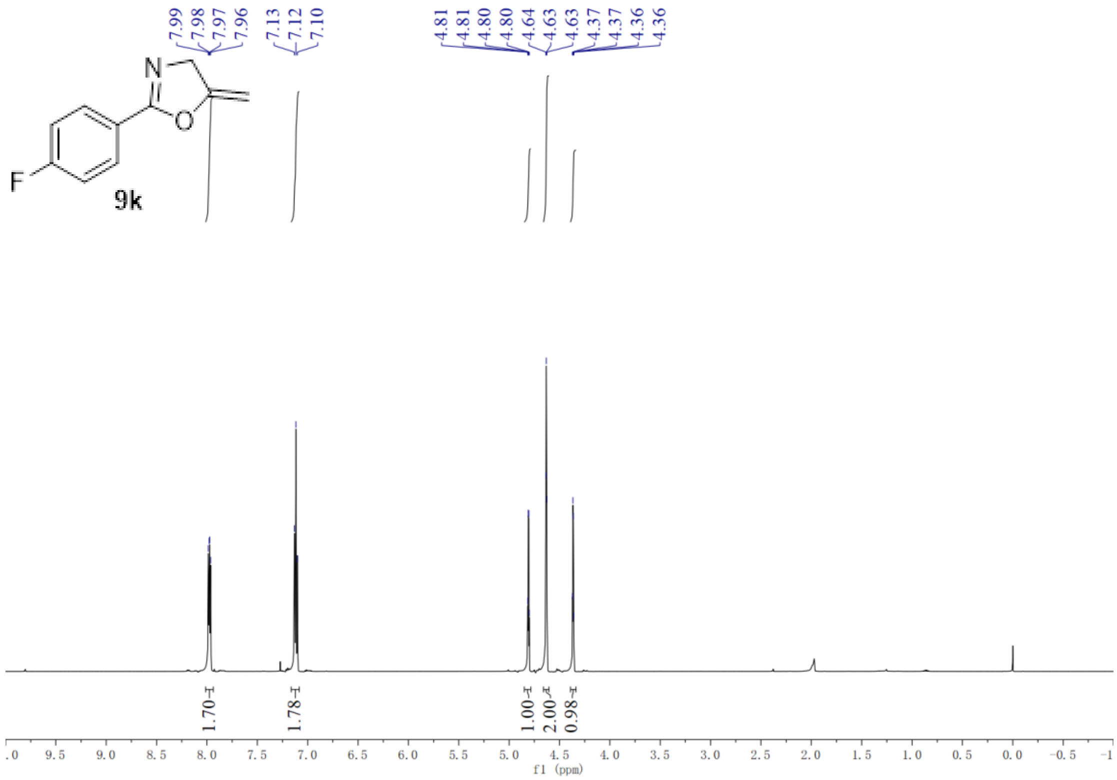


**Supplementary Figure S87.** $^{1}H$ NMR (600 MHz, $CDCl_3$) Spectrum of Compound **9k**

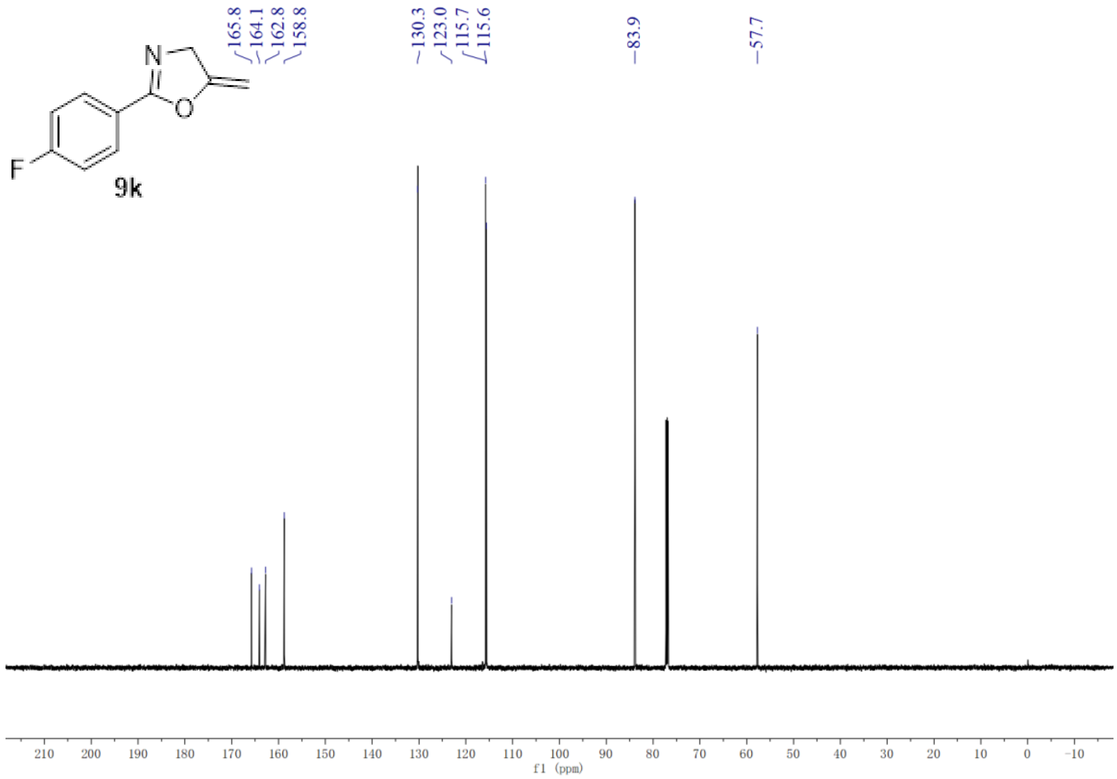


**Supplementary Figure S88.** $^{13}C$ NMR (150 MHz, $CDCl_3$) Spectrum of Compound **9k**

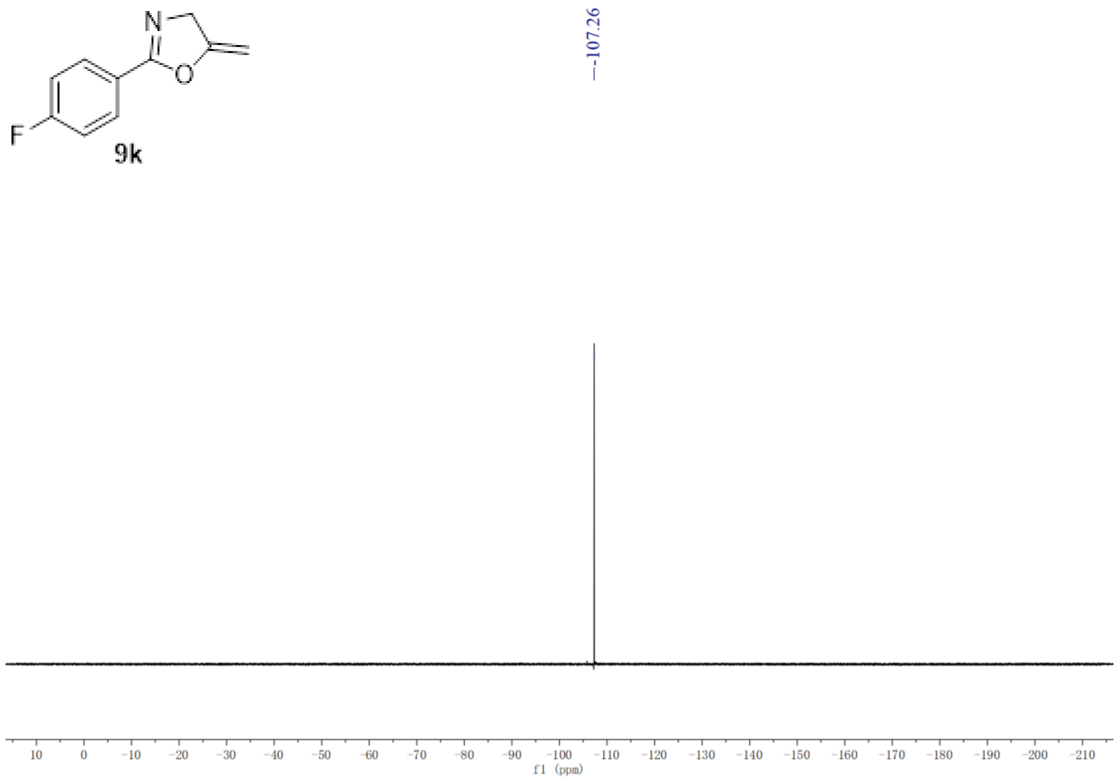


**Supplementary Figure S89.** $^{19}$F NMR (565 MHz, $CDCl_3$) Spectrum of Compound **9k**

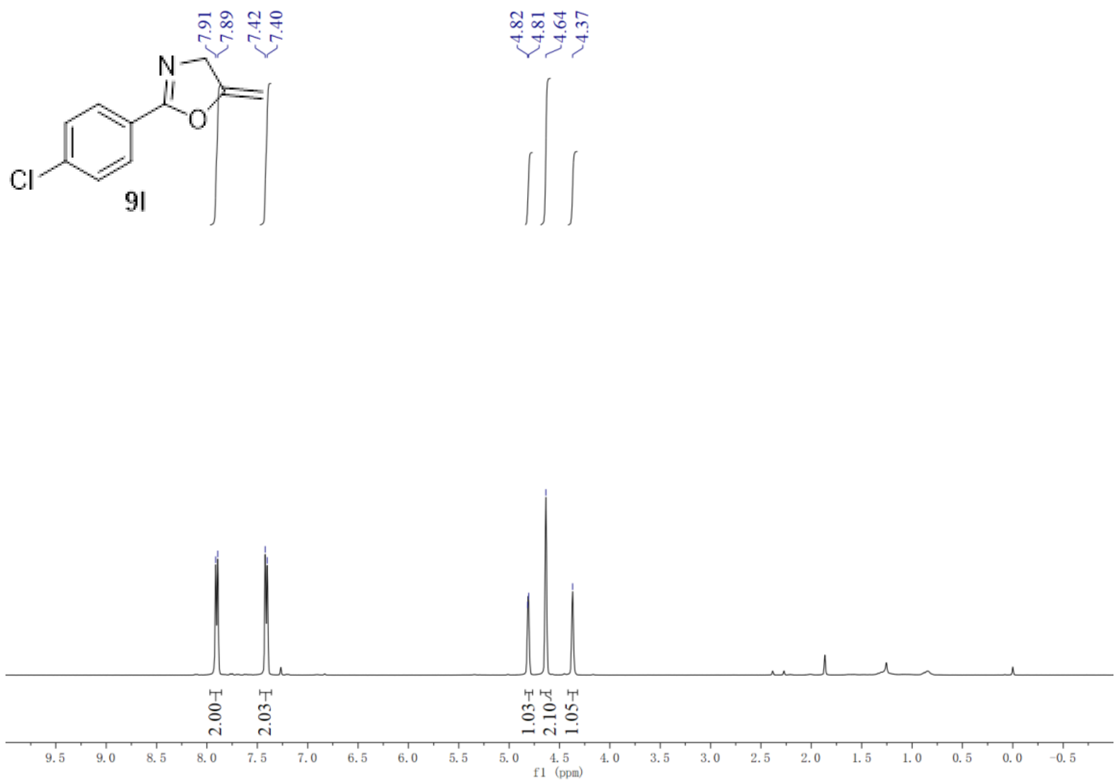


**Supplementary Figure S90.** $^{1}$H NMR (400 MHz, $CDCl_3$) Spectrum of Compound **9l**

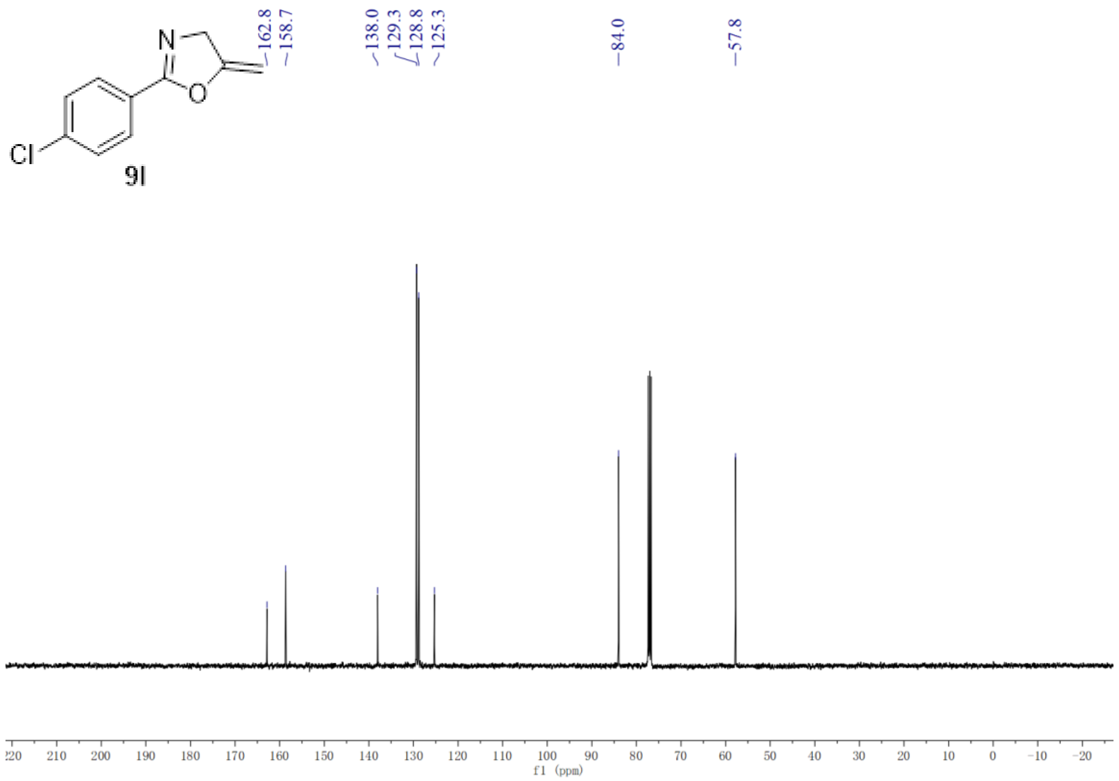


**Supplementary Figure S91.** $^{13}$C NMR (100 MHz, $CDCl_3$) Spectrum of Compound **9l**

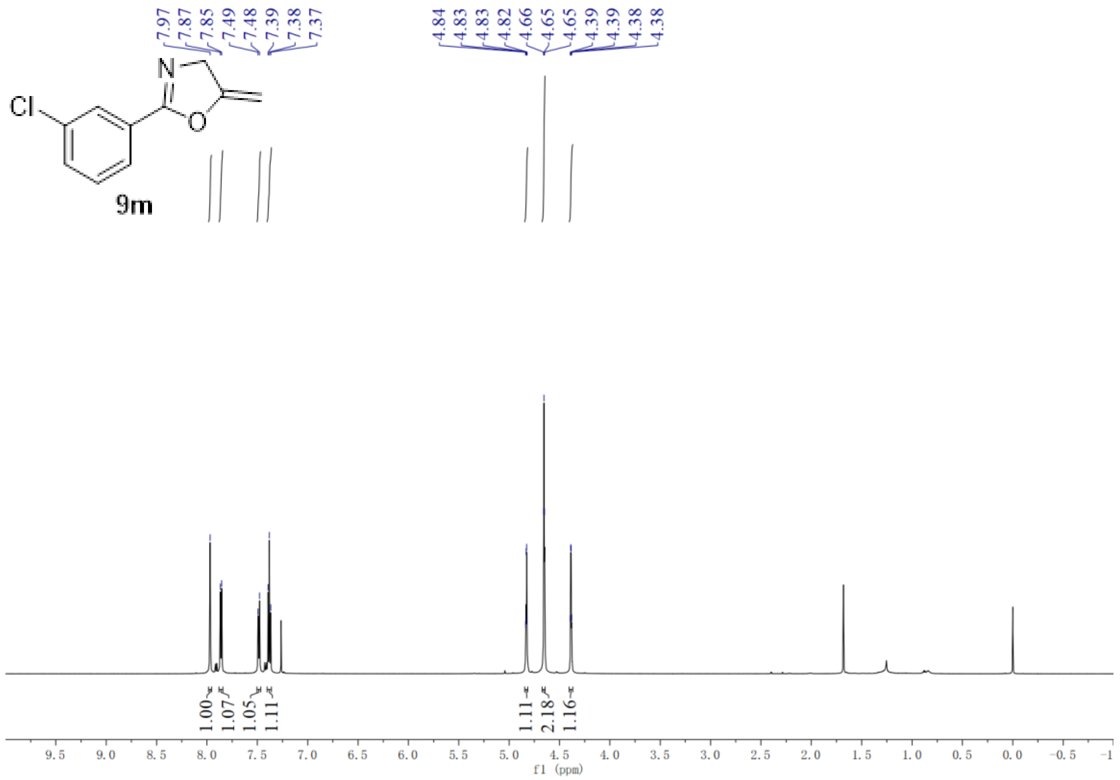


**Supplementary Figure S92.** $^{1}$H NMR (600 MHz, $CDCl_3$) Spectrum of Compound **9m**

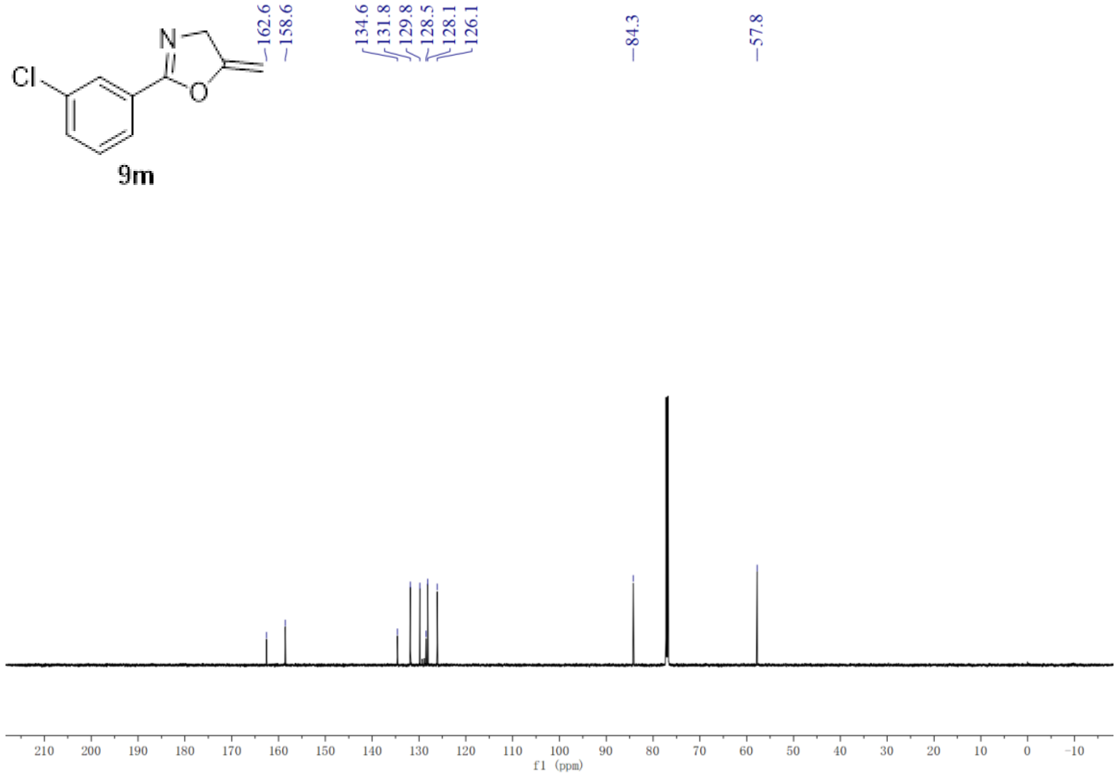


**Supplementary Figure S93.** $^{13}$C NMR (150 MHz, $CDCl_3$) Spectrum of Compound **9m**

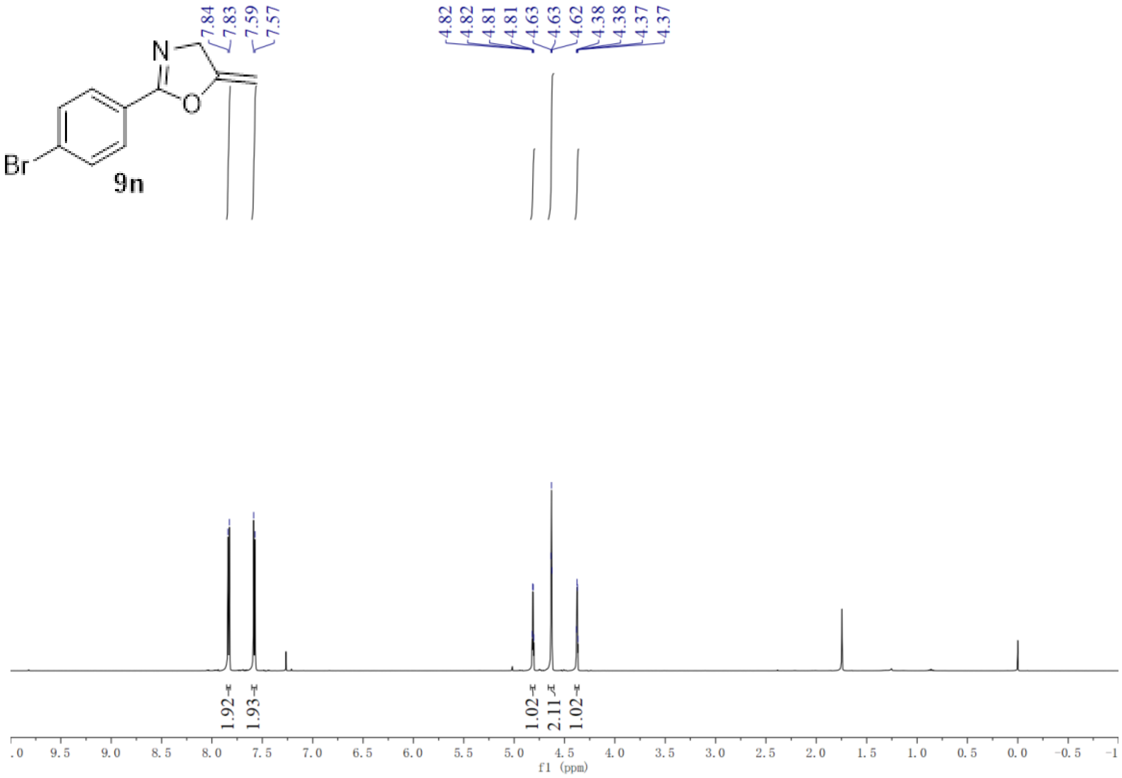


**Supplementary Figure S94.** $^{1}$H NMR (600 MHz, $CDCl_3$) Spectrum of Compound **9n**

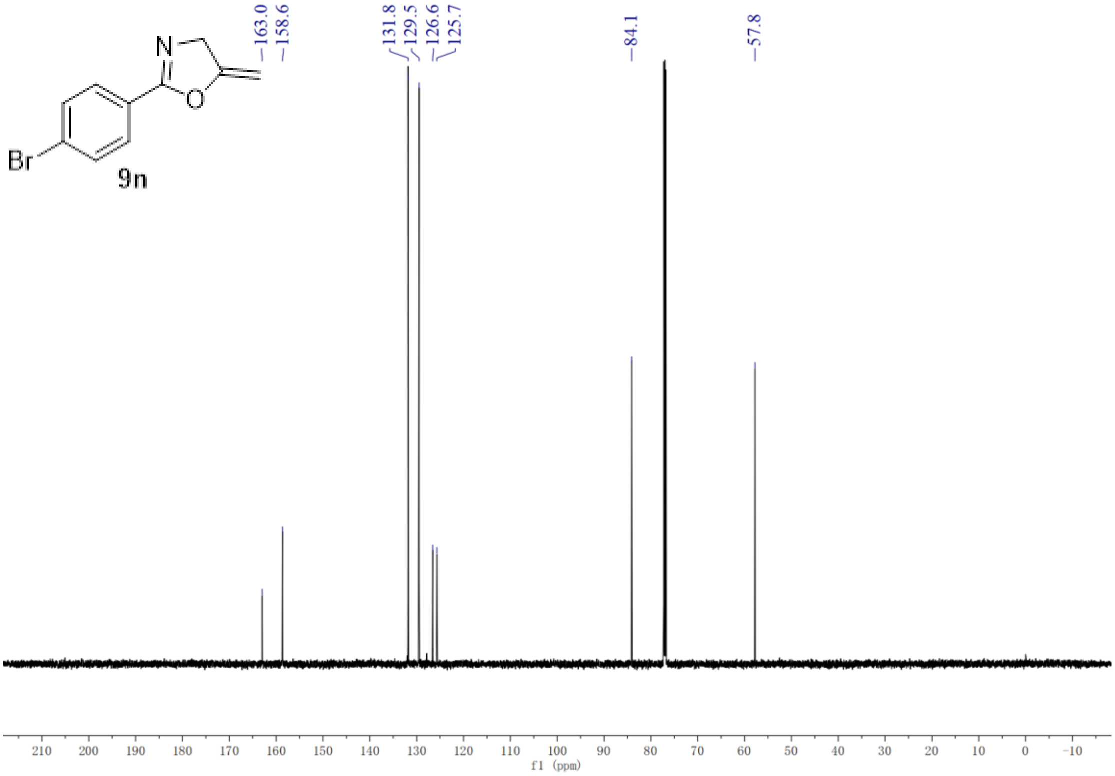


**Supplementary Figure S95.** $^{13}$C NMR (150 MHz, $CDCl_3$) Spectrum of Compound **9n**

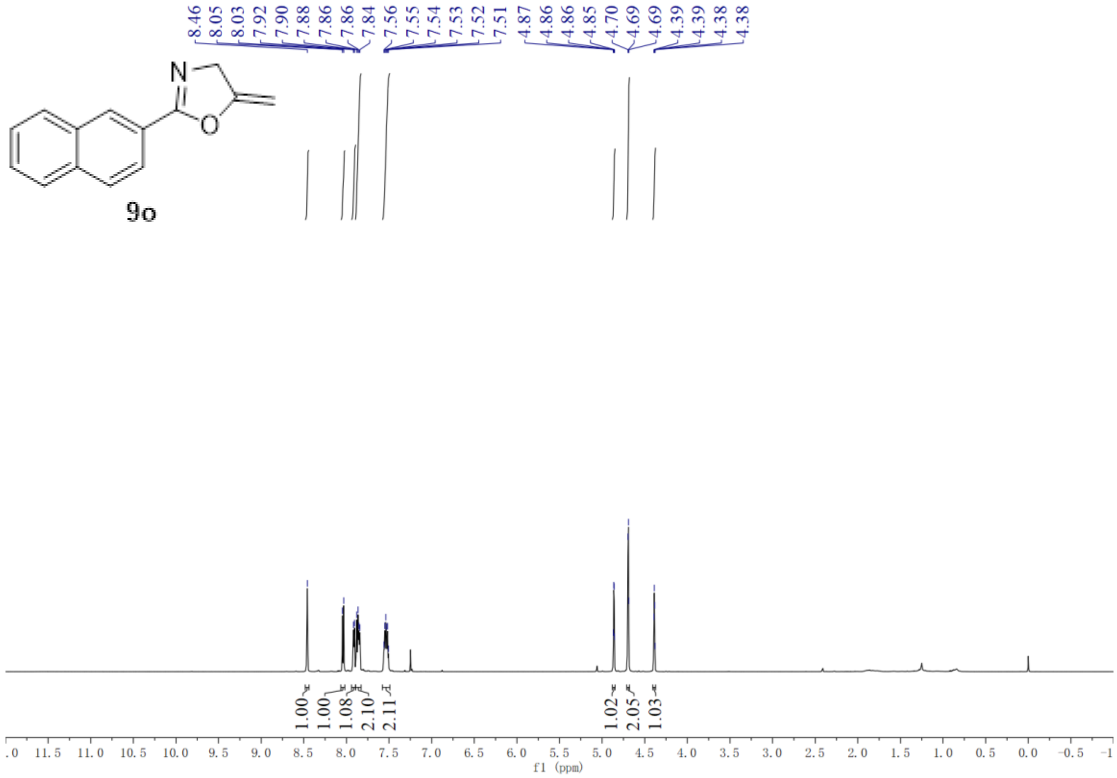


**Supplementary Figure S96.** $^{1}$H NMR (600 MHz, $CDCl_3$) Spectrum of Compound **9o**

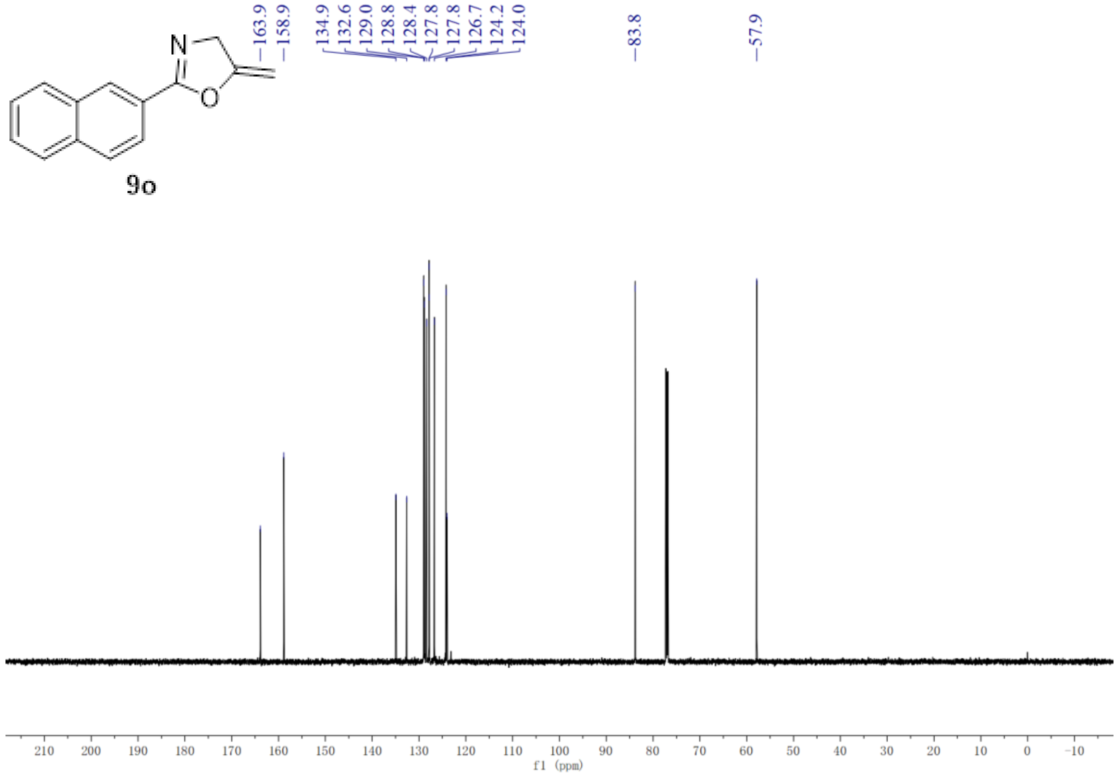


**Supplementary Figure S97.** $^{13}$C NMR (150 MHz, $CDCl_3$) Spectrum of Compound **9o**

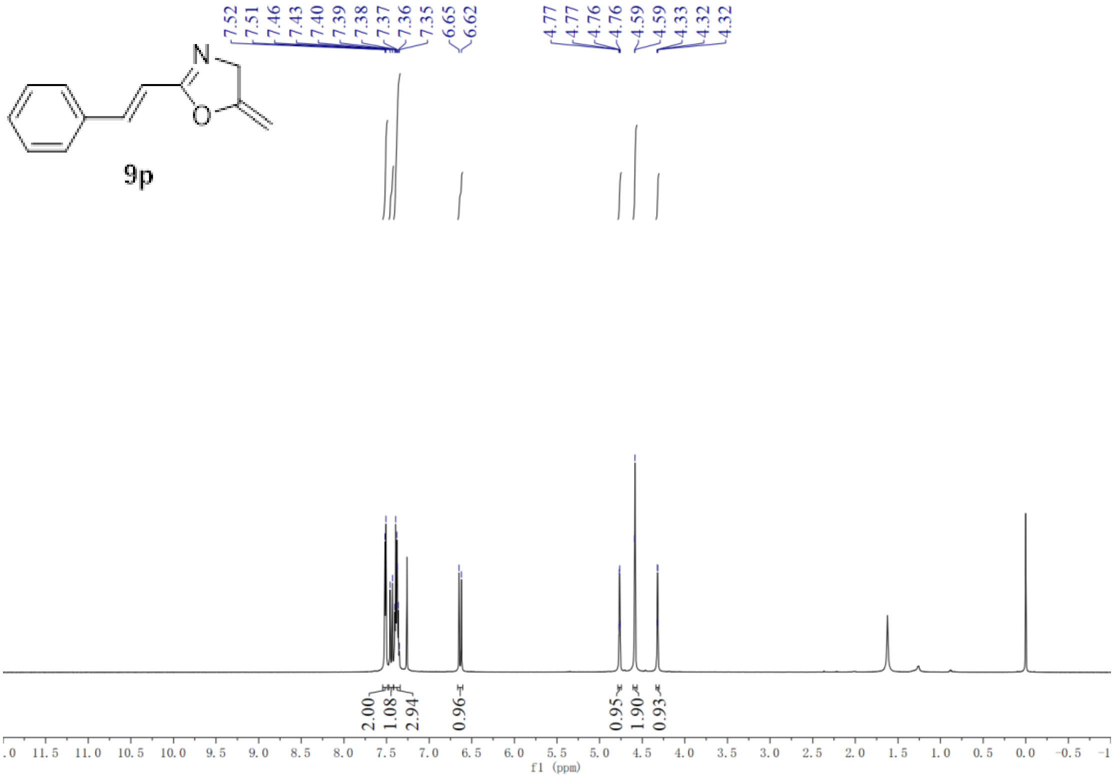


**Supplementary Figure S98.** $^{1}$H NMR (600 MHz, $CDCl_3$) Spectrum of Compound **9p**

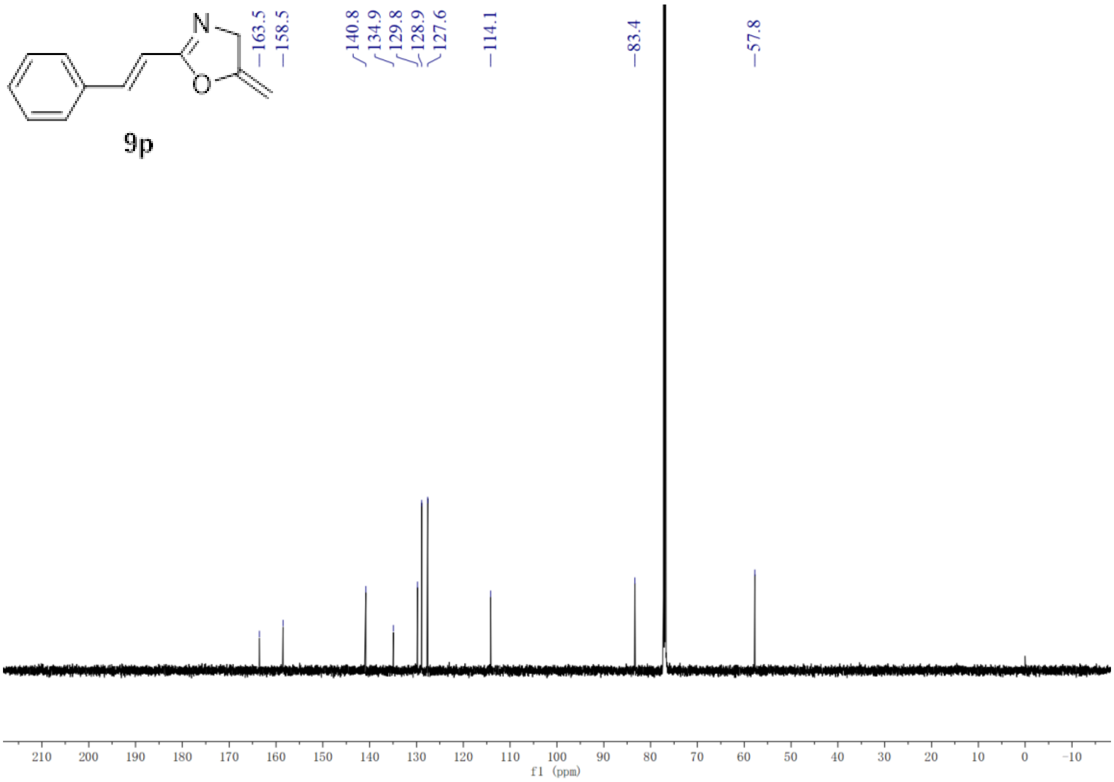


**Supplementary Figure S99.** $^{13}$C NMR (150 MHz, $CDCl_3$) Spectrum of Compound **9p**

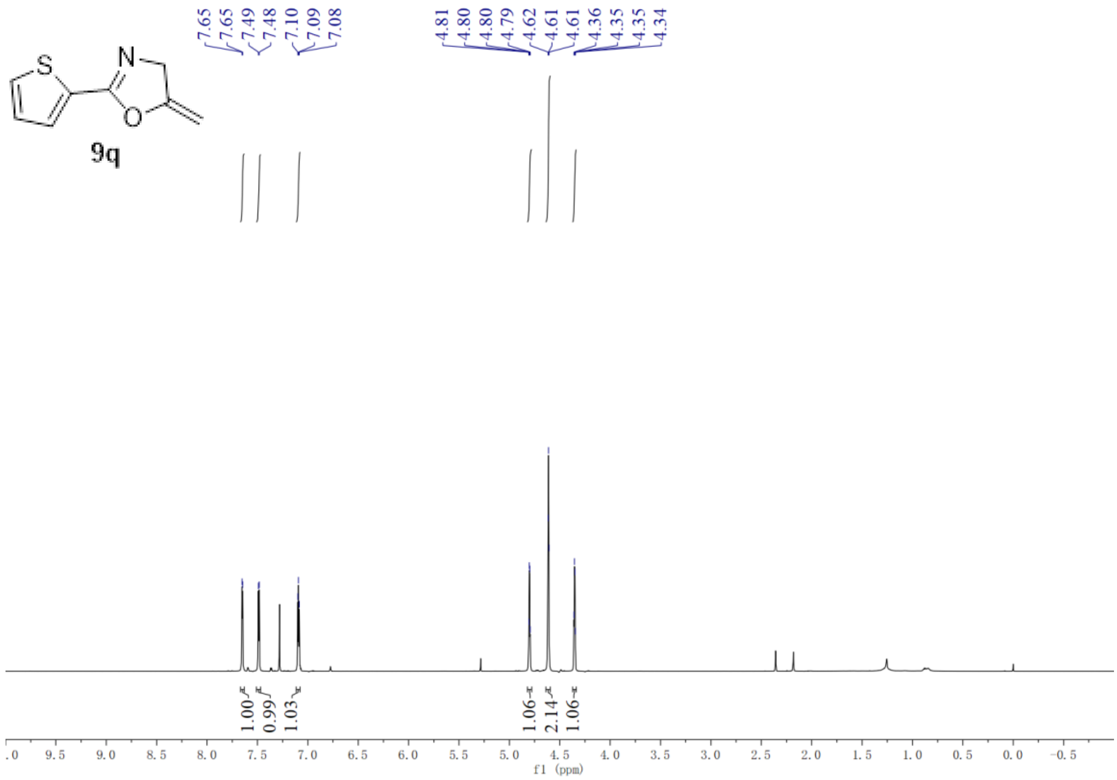


**Supplementary Figure S100.** $^{1}$H NMR (600 MHz, $CDCl_3$) Spectrum of Compound **9q**

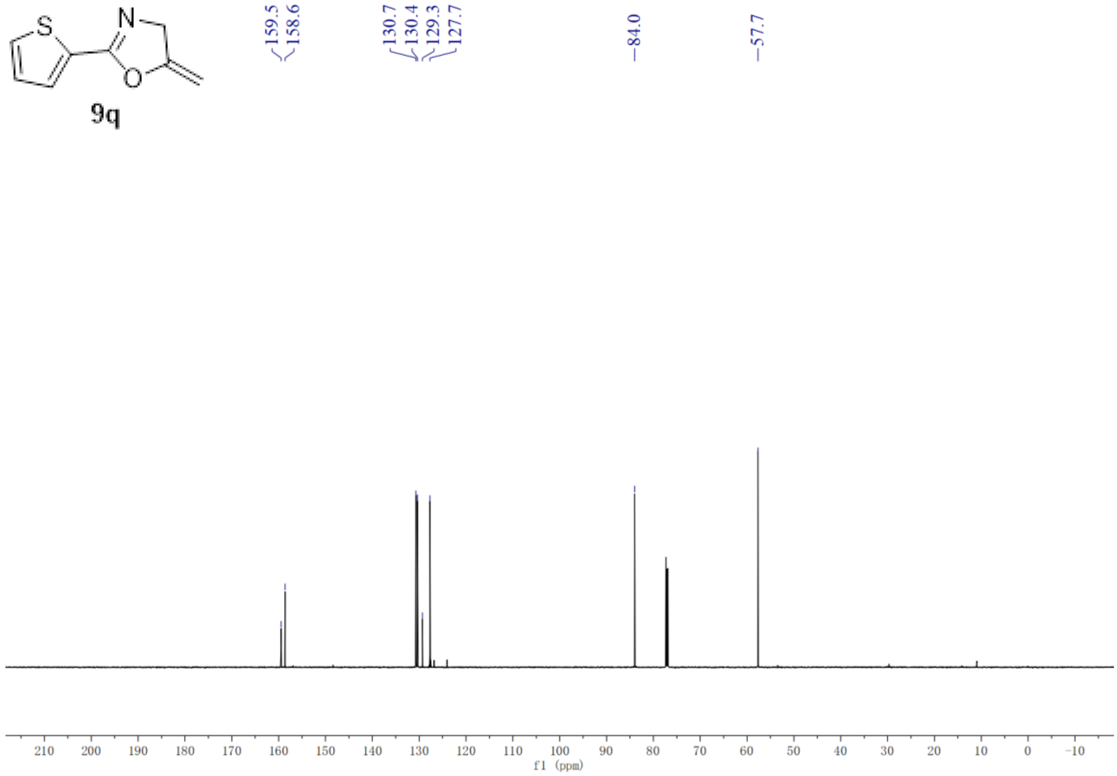


**Supplementary Figure S101.** $^{13}$C NMR (150 MHz, $CDCl_3$) Spectrum of Compound **9q**

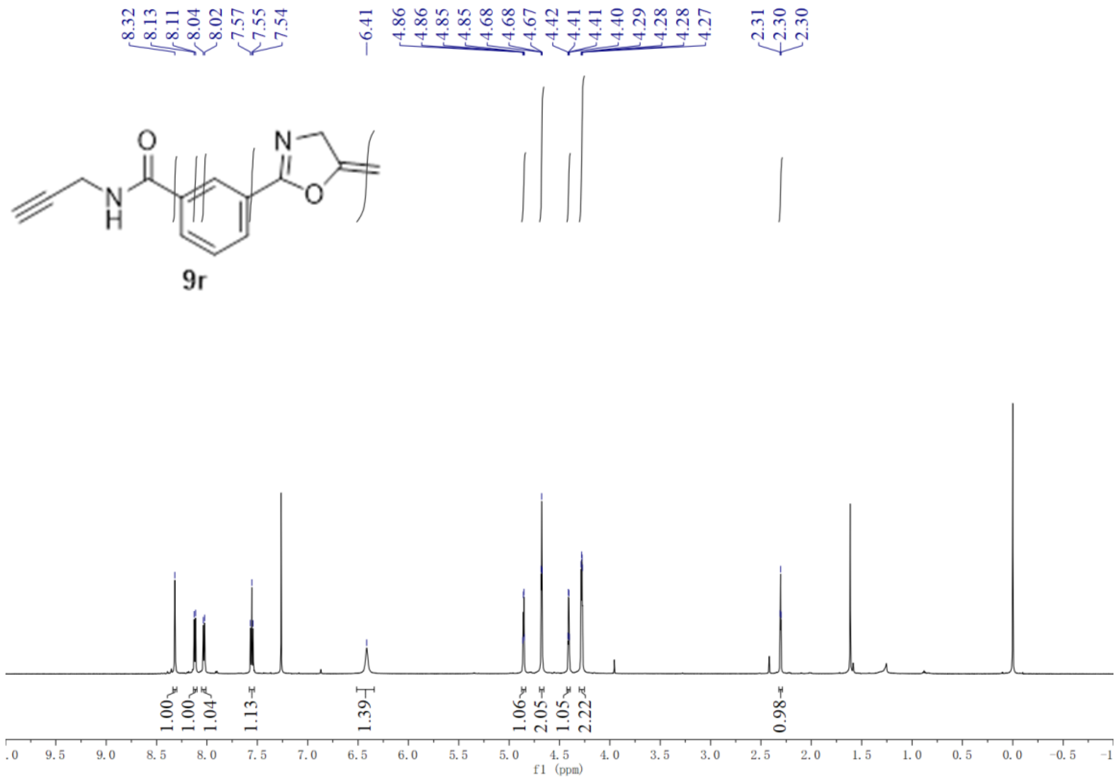

**Supplementary Figure S102.** $^{1}$H NMR (600 MHz, $CDCl_3$) Spectrum of Compound **9r**

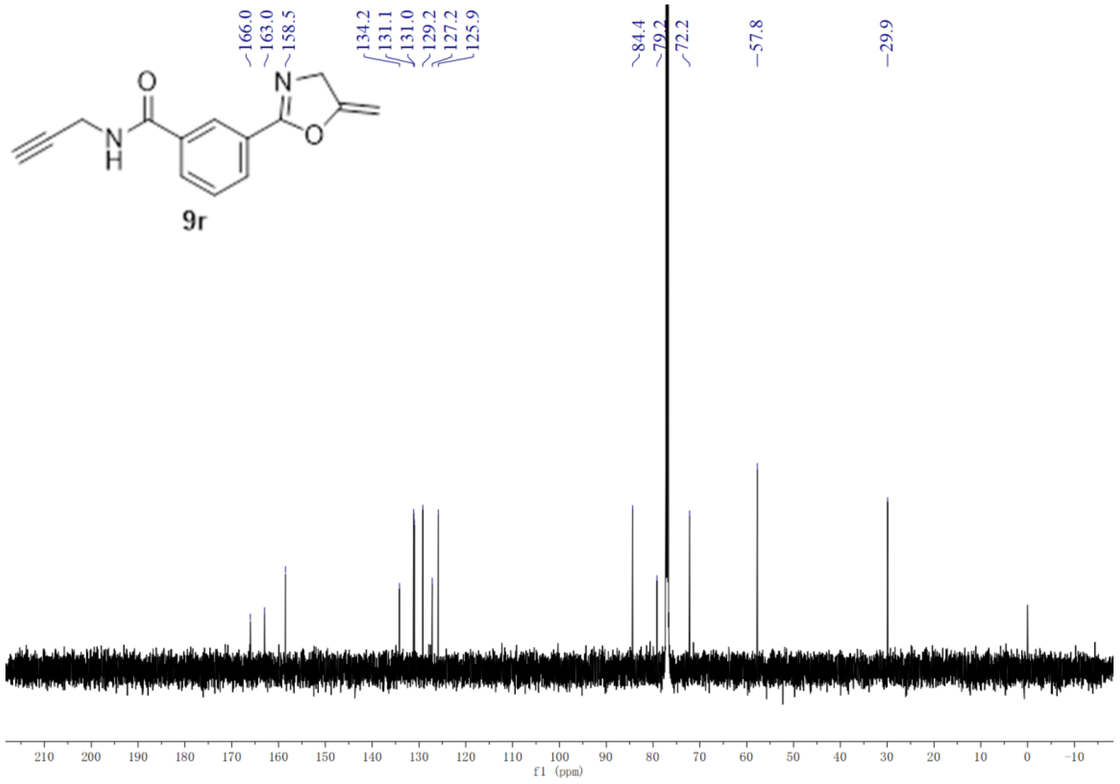

**Supplementary Figure S103.** $^{13}$C NMR (150 MHz, $CDCl_3$) Spectrum of Compound **9r**

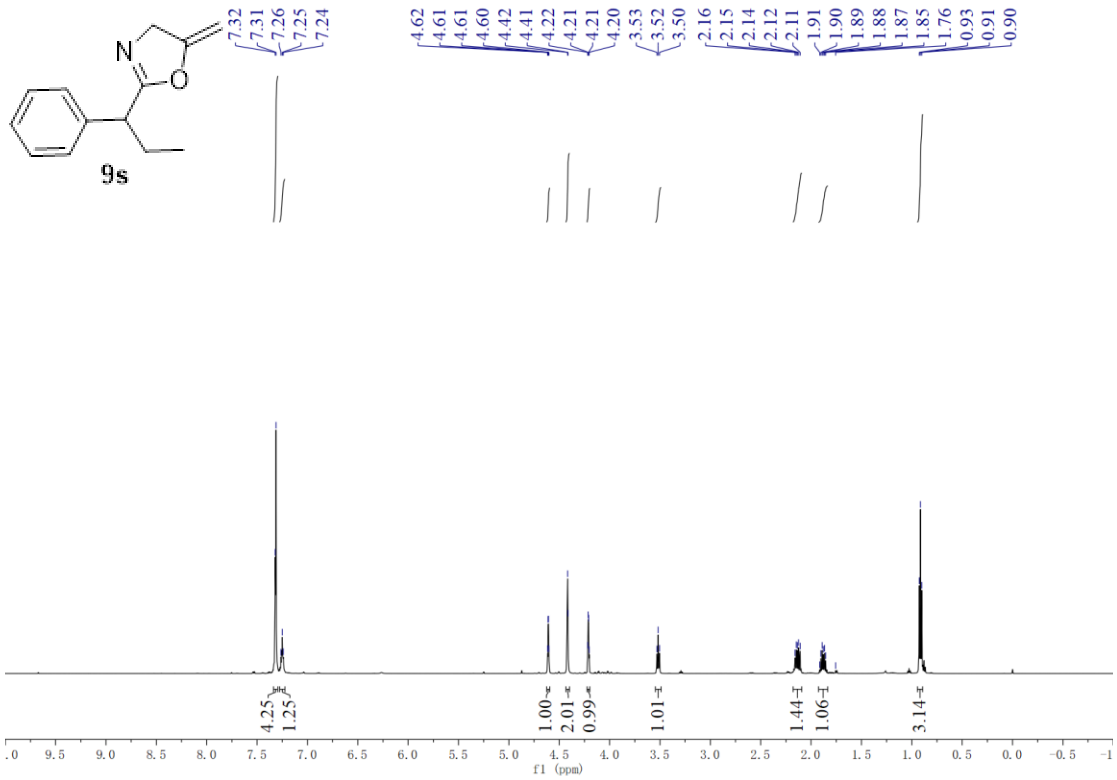

**Supplementary Figure S104.** $^{1}$H NMR (600 MHz, $CDCl_3$) Spectrum of Compound **9s**

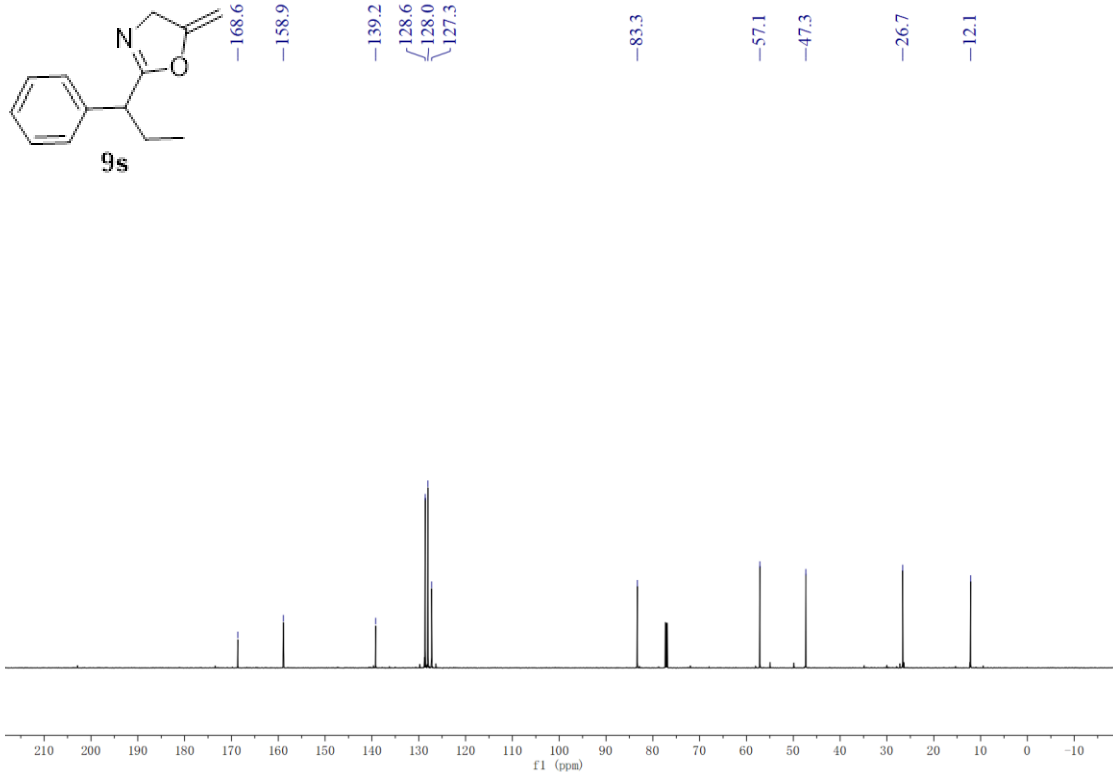

**Supplementary Figure S105.** $^{13}$C NMR (150 MHz, $CDCl_3$) Spectrum of Compound **9s**

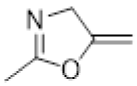

9t

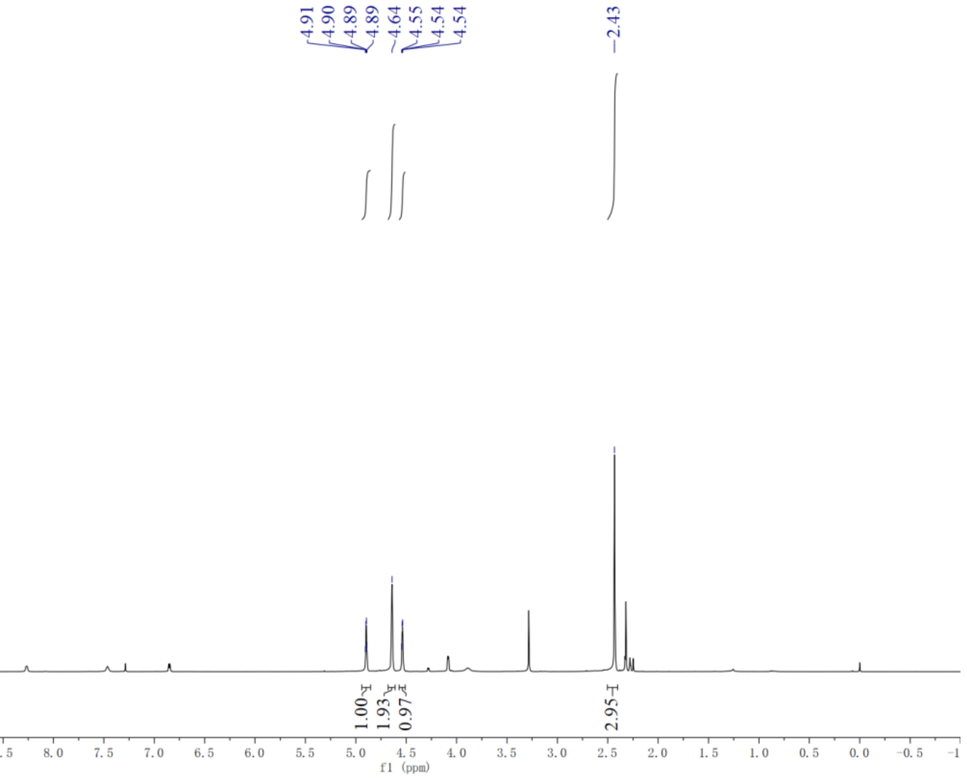


**Supplementary Figure S106.** $^{1}H$ NMR (600 MHz, $CDCl_3$) Spectrum of Compound **9t**

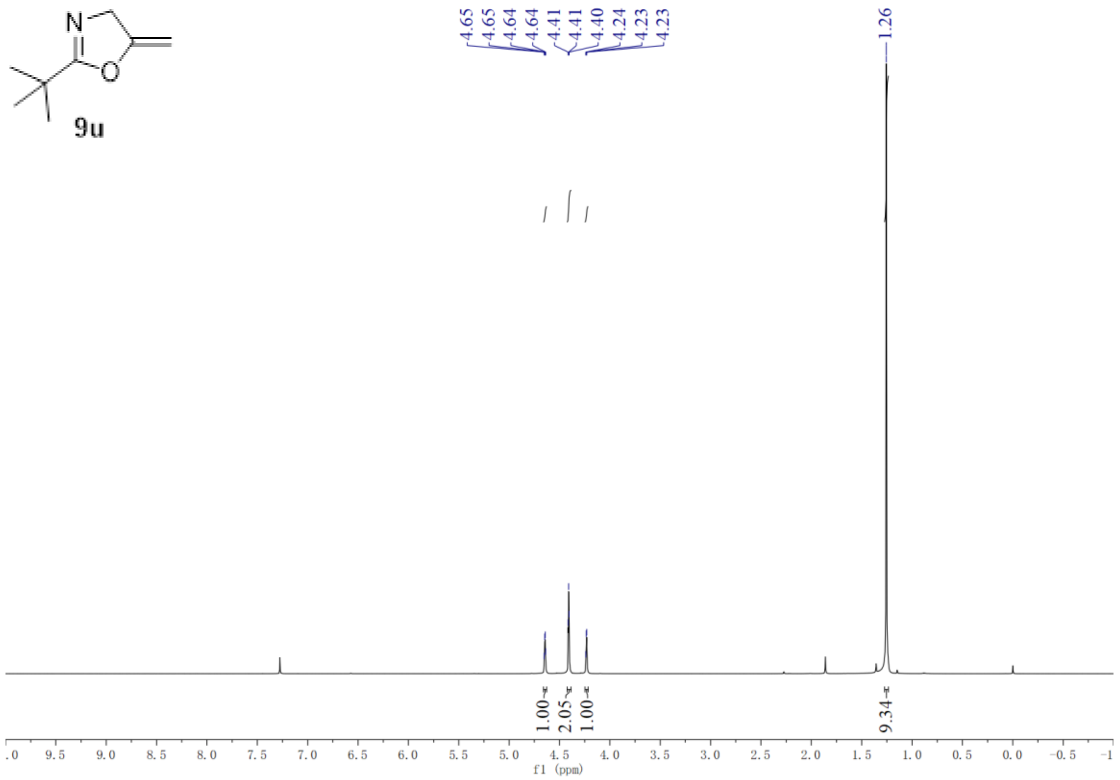


**Supplementary Figure S107.** $^{1}H$ NMR (600 MHz, $CDCl_3$) Spectrum of Compound **9u**

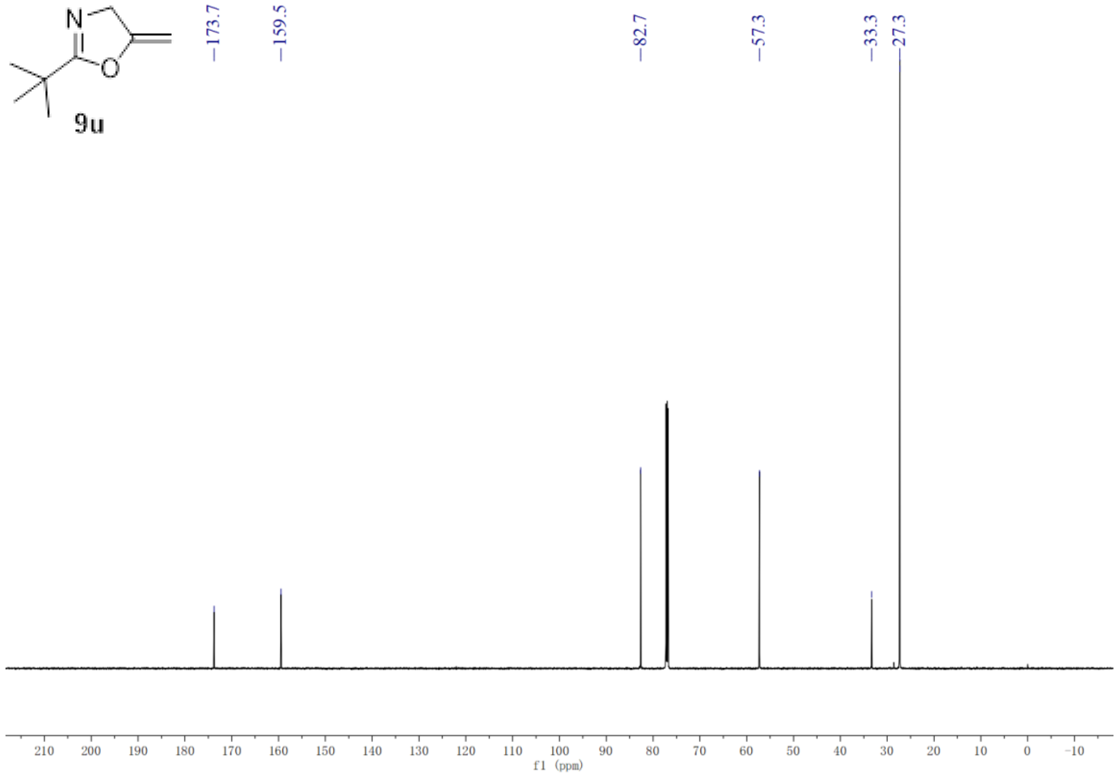


**Supplementary Figure S108.** $^{13}C$ NMR (150 MHz, $CDCl_3$) Spectrum of Compound **9u**

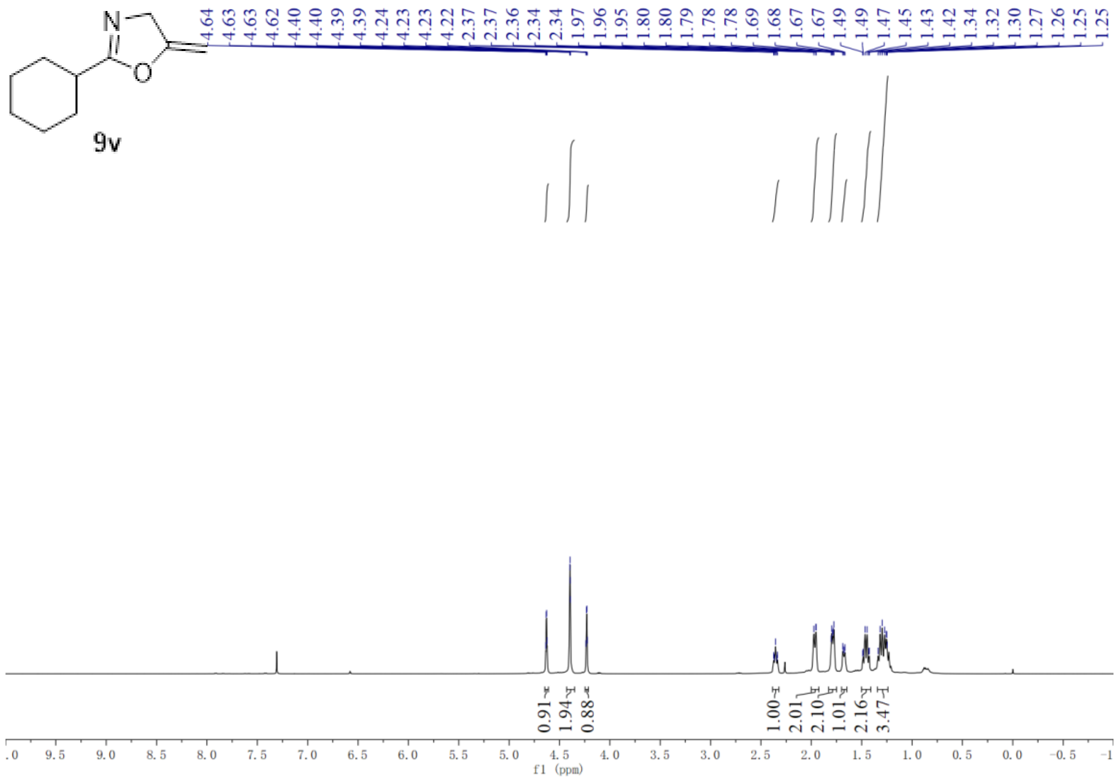

**Supplementary Figure S109.** $^{1}H$ NMR (600 MHz, $CDCl_3$) Spectrum of Compound **9v**

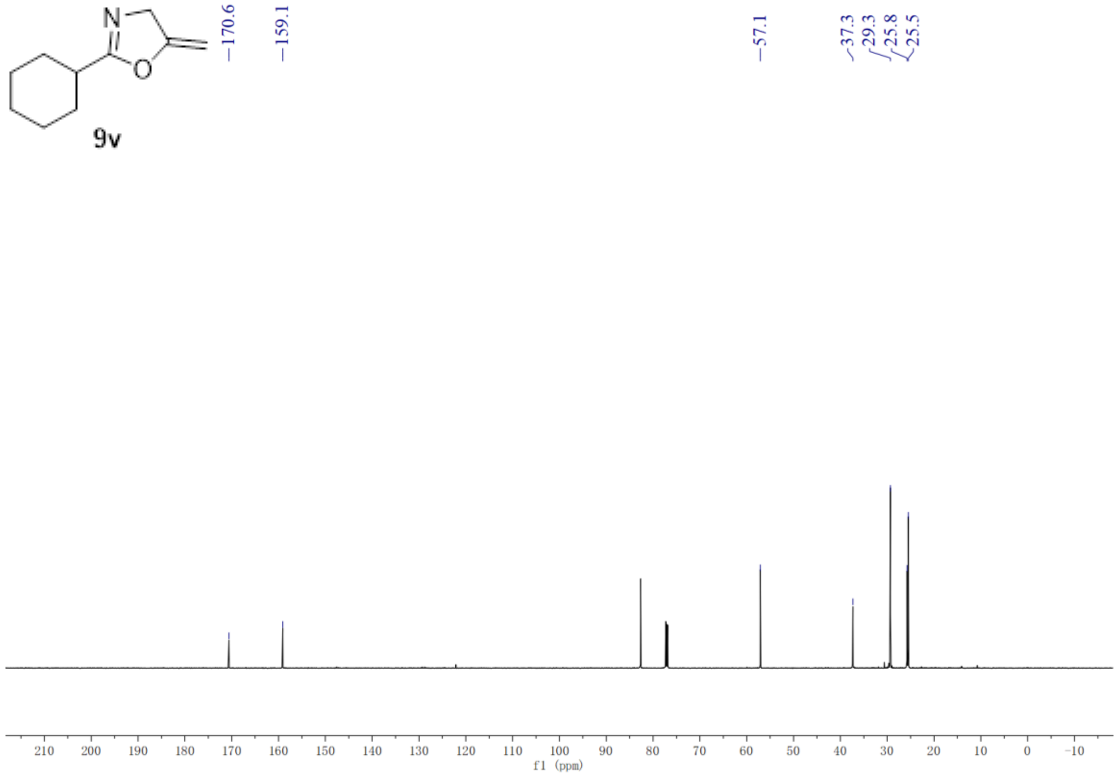

**Supplementary Figure S110.** $^{13}C$ NMR (150 MHz, $CDCl_3$) Spectrum of Compound **9v**

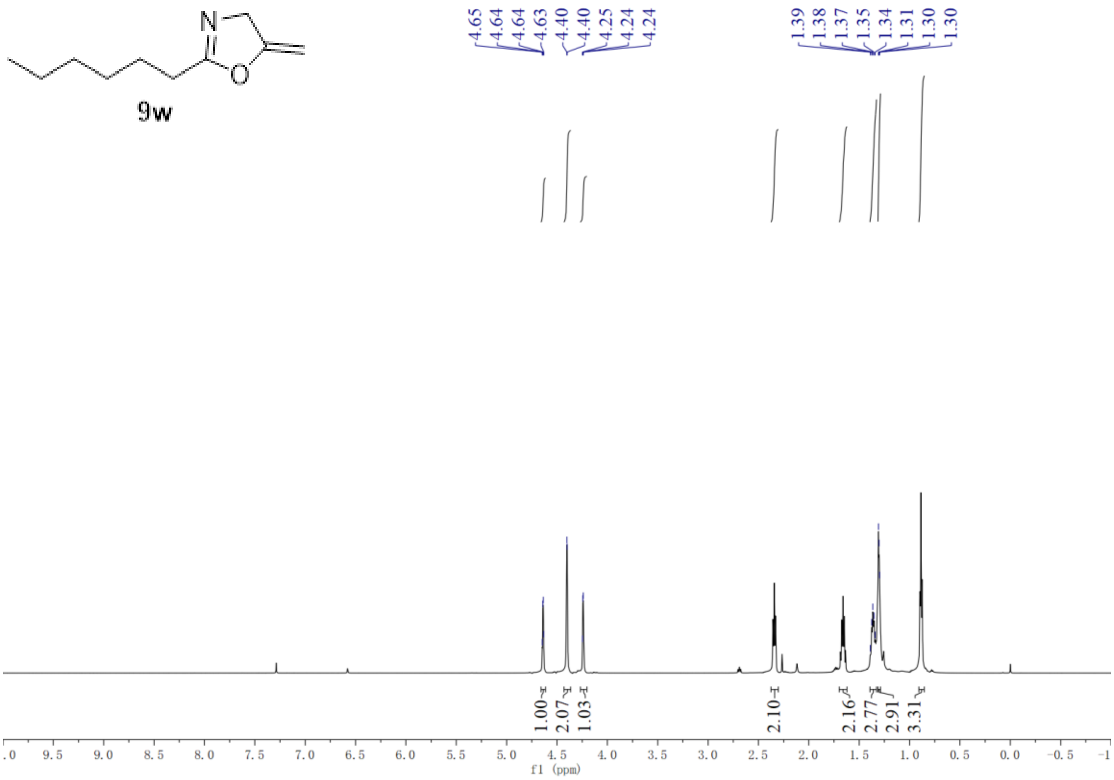


**Supplementary Figure S111.** $^{1}H$ NMR (600 MHz, $CDCl_3$) Spectrum of Compound **9w**

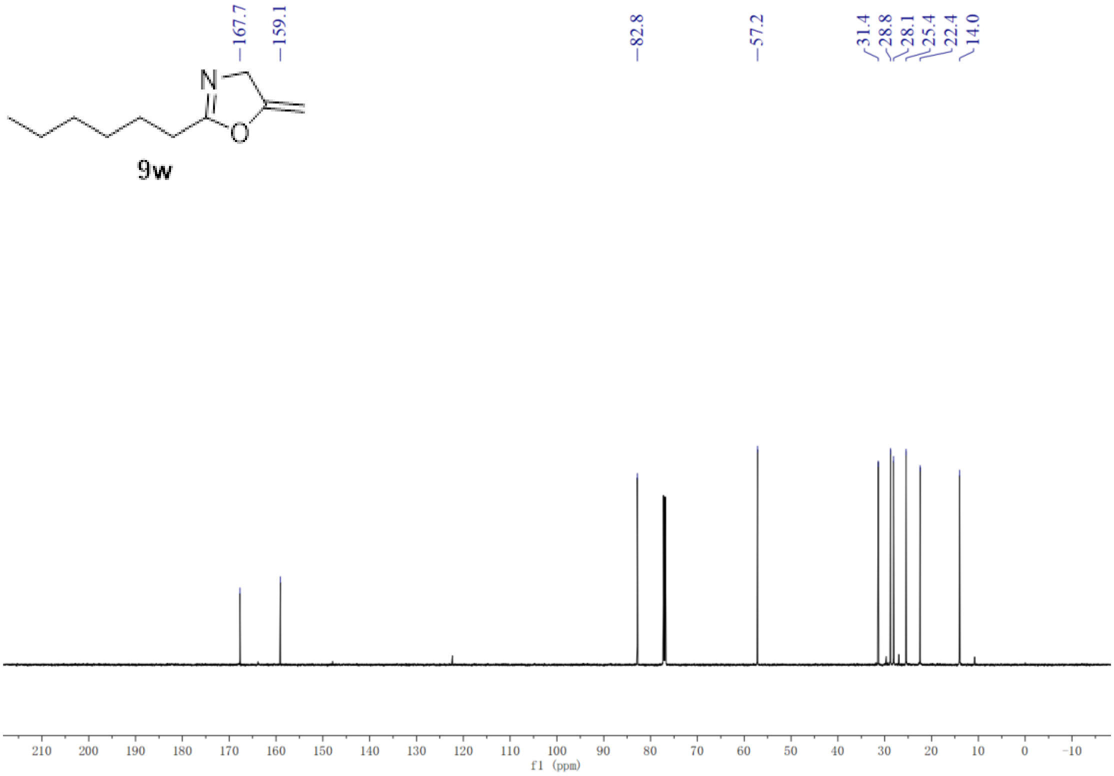


**Supplementary Figure S112.** $^{13}C$ NMR (150 MHz, $CDCl_3$) Spectrum of Compound **9w**

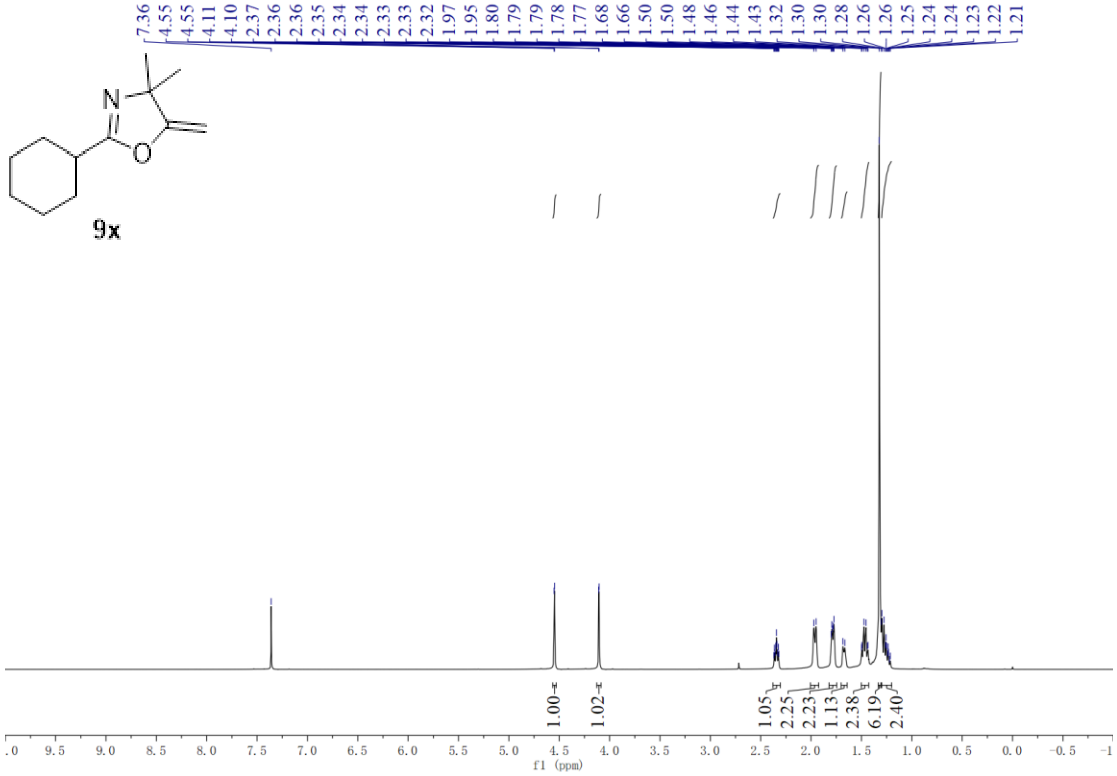


**Supplementary Figure S113.** $^{1}$H NMR (600 MHz, $CDCl_3$) Spectrum of Compound **9x**

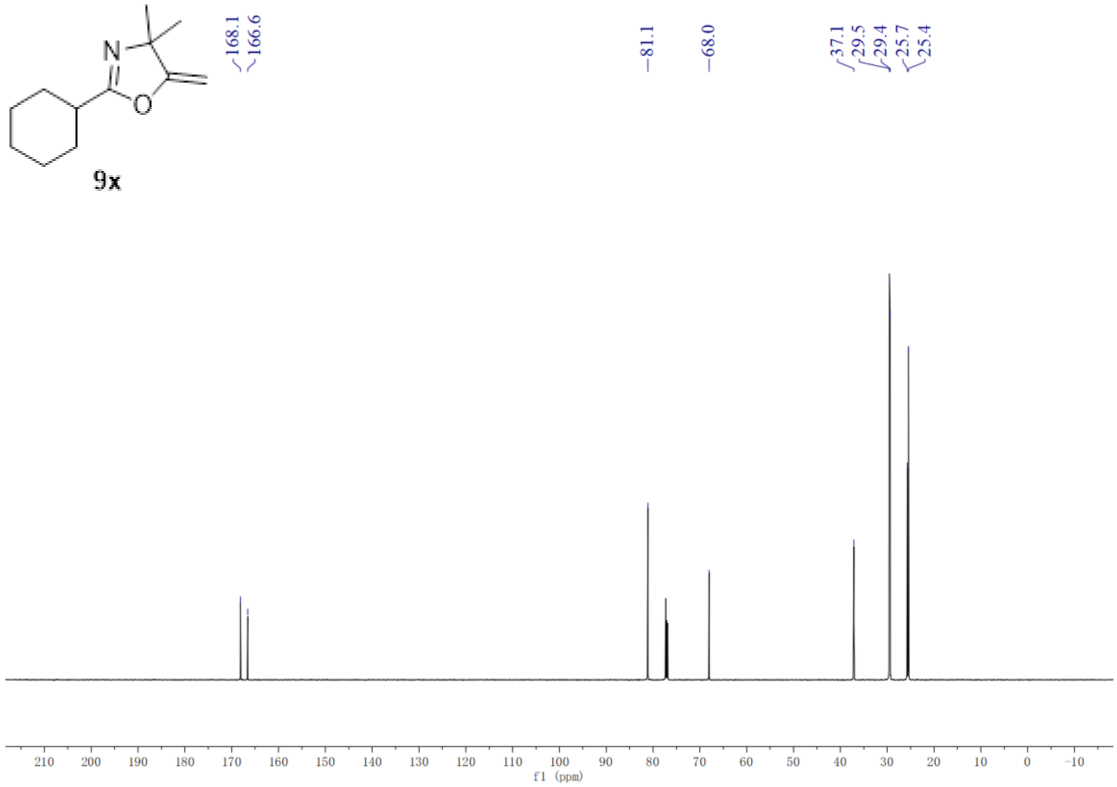


**Supplementary Figure S114.** $^{13}$C NMR (150 MHz, $CDCl_3$) Spectrum of Compound **9x**

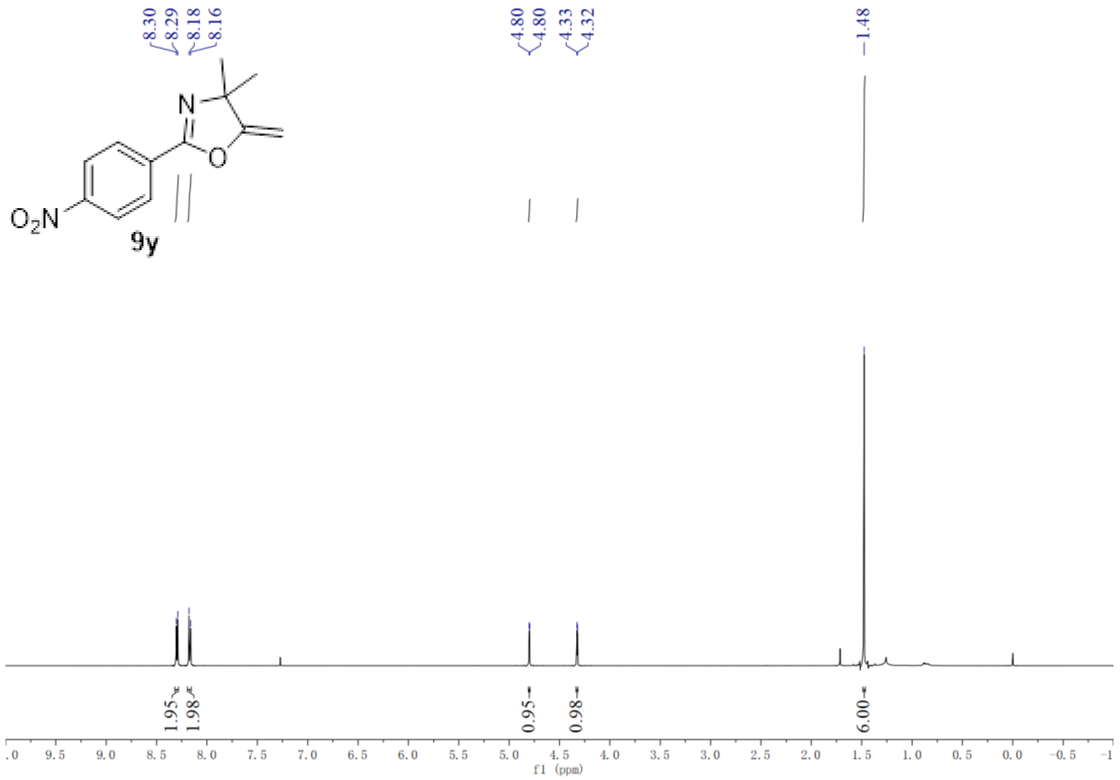


**Supplementary Figure S115.** $^{1}$H NMR (600 MHz, $CDCl_3$) Spectrum of Compound **9y**

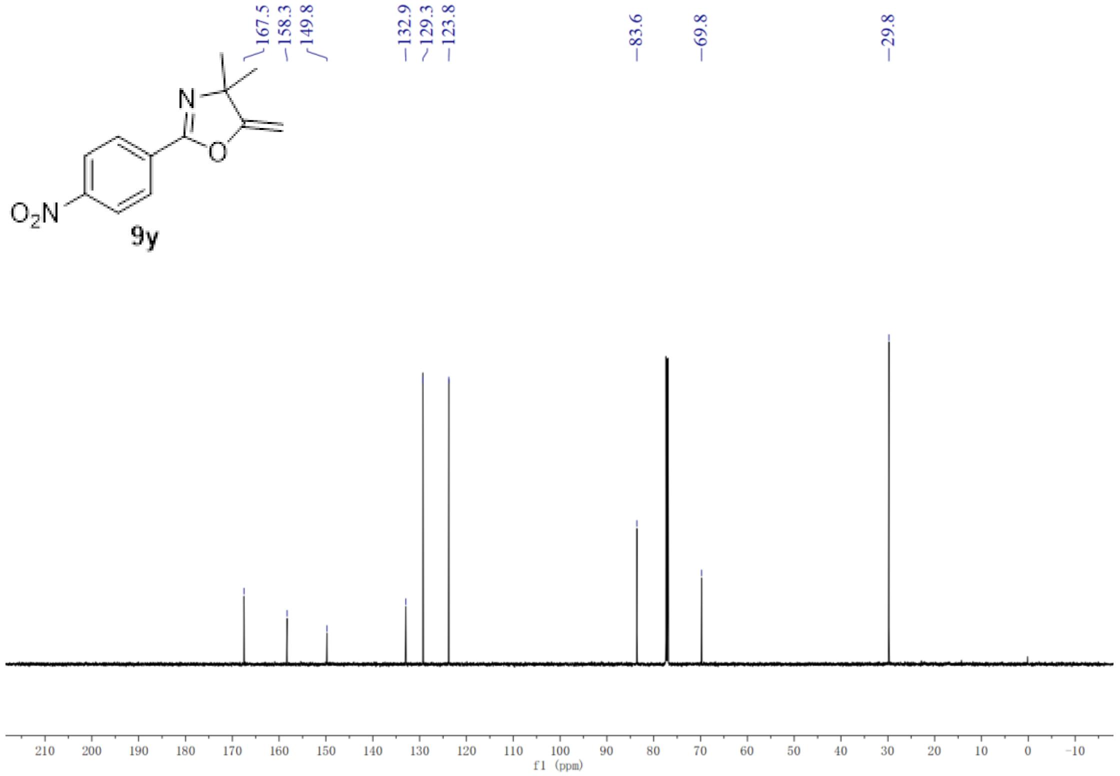


**Supplementary Figure S116.** $^{13}$C NMR (150 MHz, $CDCl_3$) Spectrum of Compound **9y**

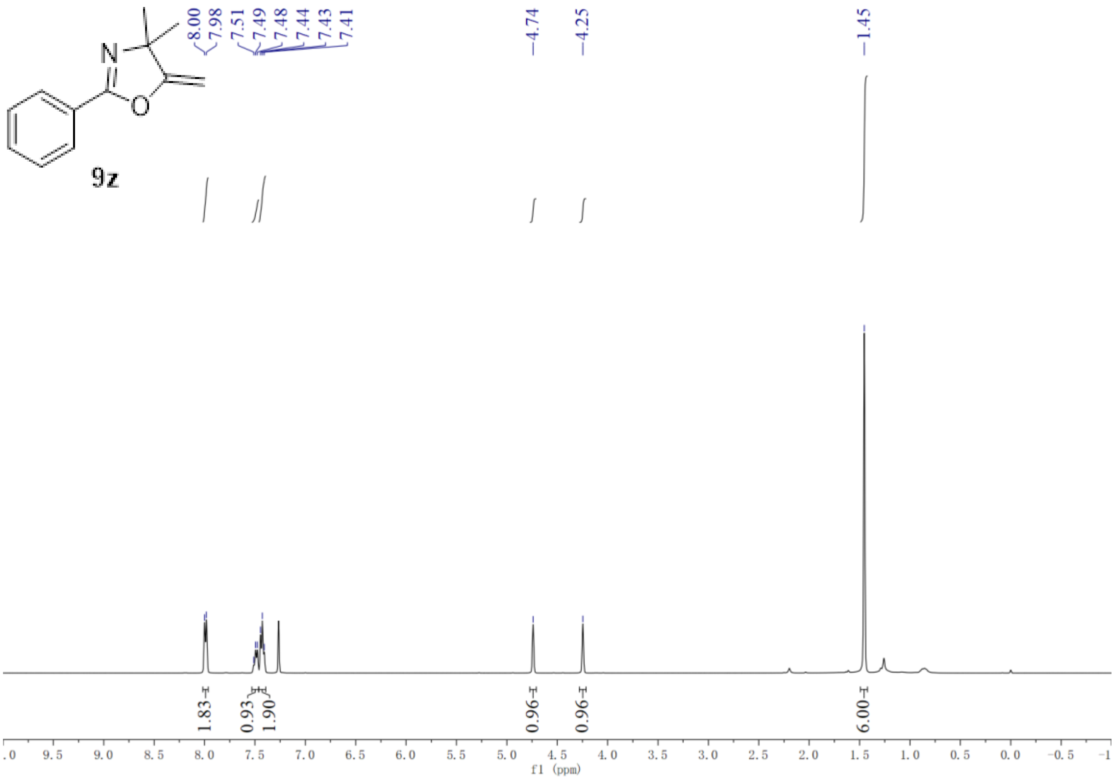


**Supplementary Figure S117.** $^{1}H$ NMR (400 MHz, $CDCl_3$) Spectrum of Compound **9z**

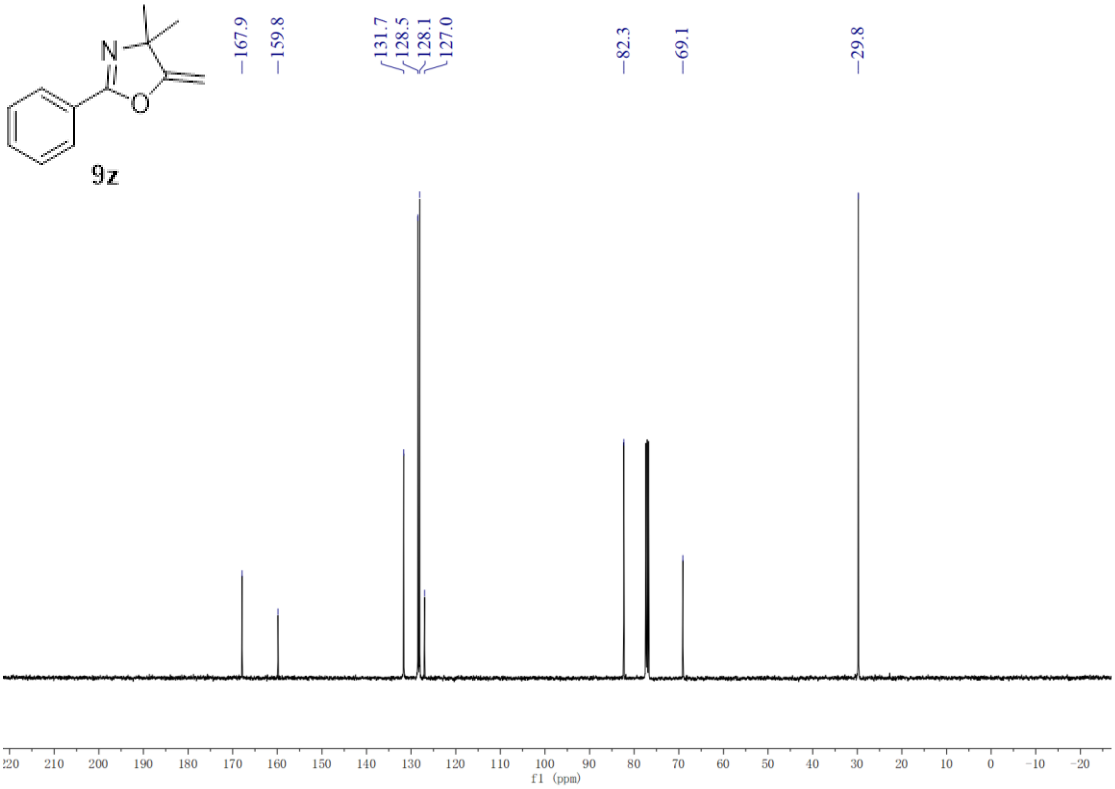


**Supplementary Figure S118.** $^{13}C$ NMR (100 MHz, $CDCl_3$) Spectrum of Compound **9z**

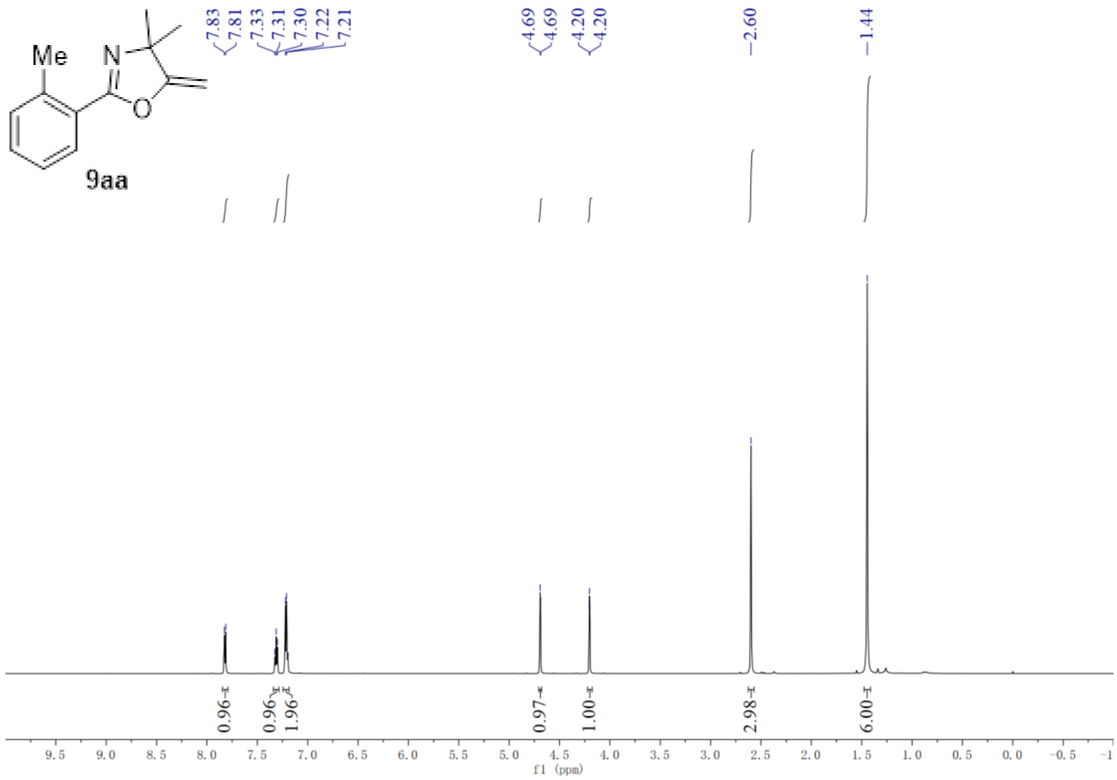


**Supplementary Figure S119.** $^{1}H$ NMR (600 MHz, $CDCl_3$) Spectrum of Compound **9aa**

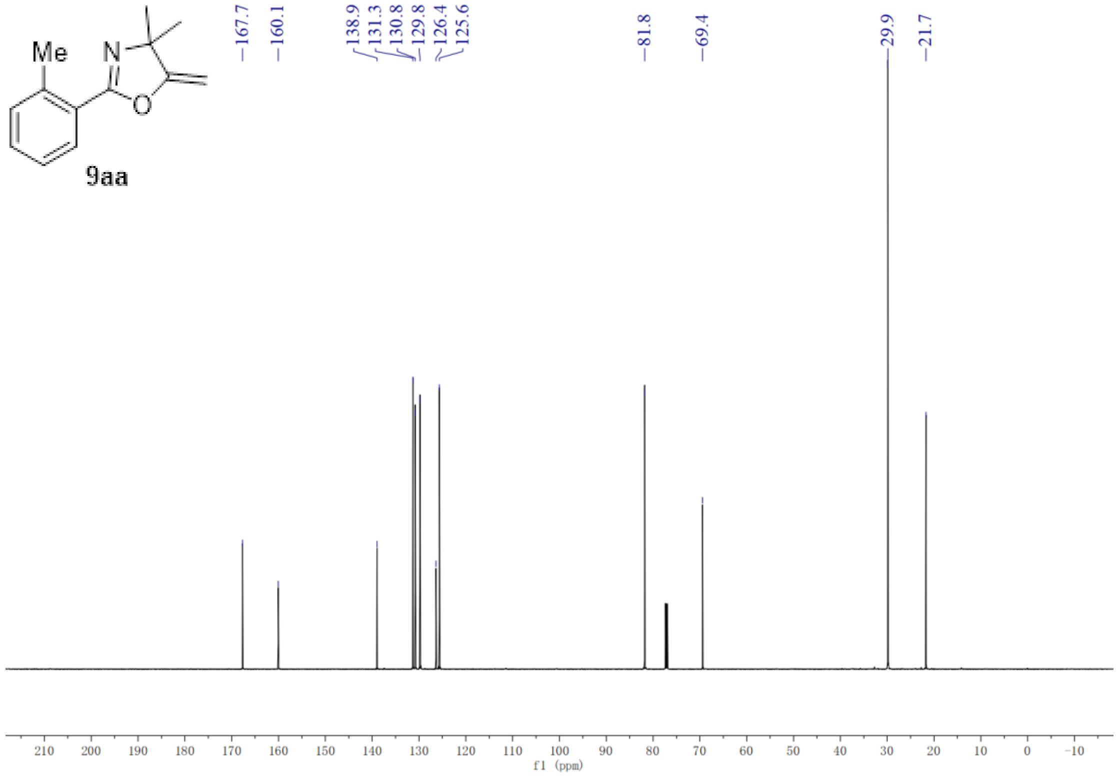


**Supplementary Figure S120.** $^{13}C$ NMR (150 MHz, $CDCl_3$) Spectrum of Compound **9aa**

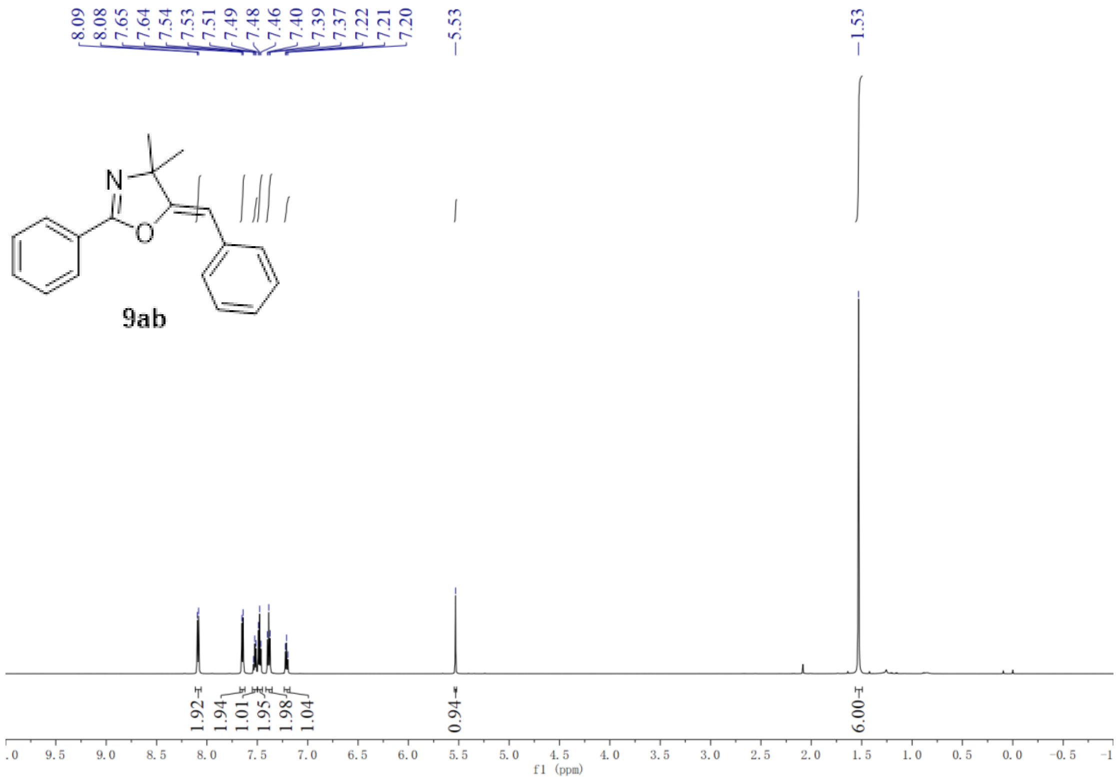


**Supplementary Figure S121.** $^{1}H$ NMR (600 MHz, $CDCl_3$) Spectrum of Compound **9ab**

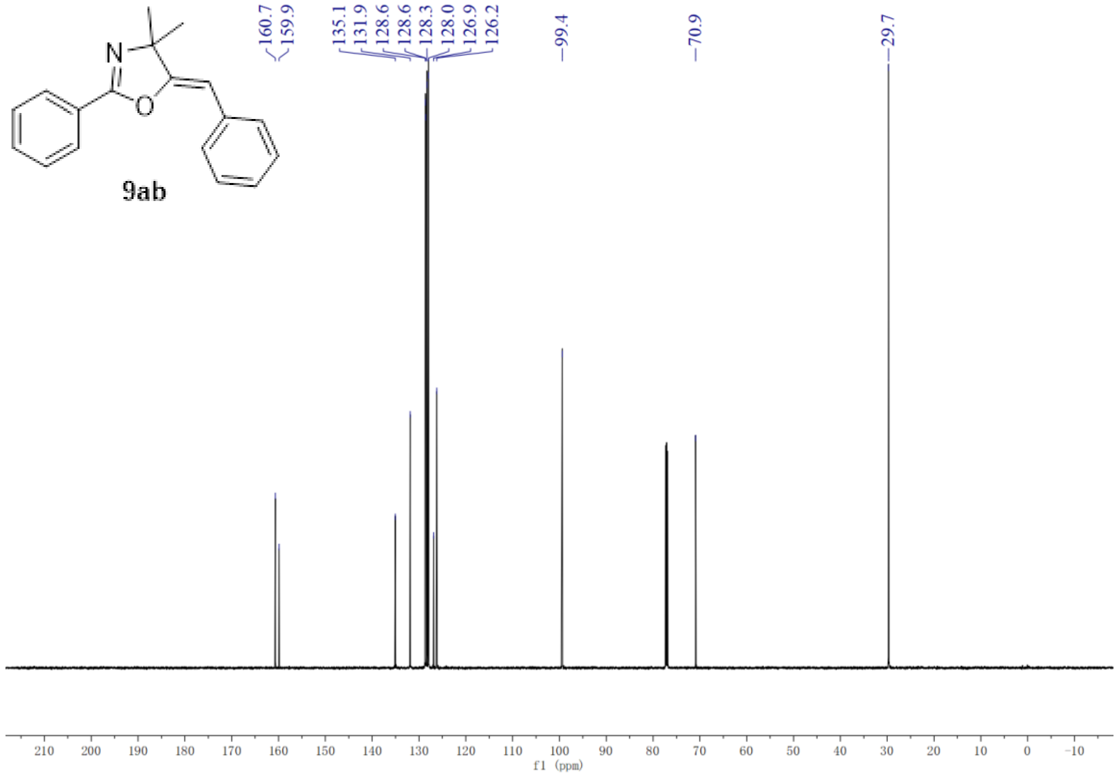


**Supplementary Figure S122.** $^{13}C$ NMR (150 MHz, $CDCl_3$) Spectrum of Compound **9ab**

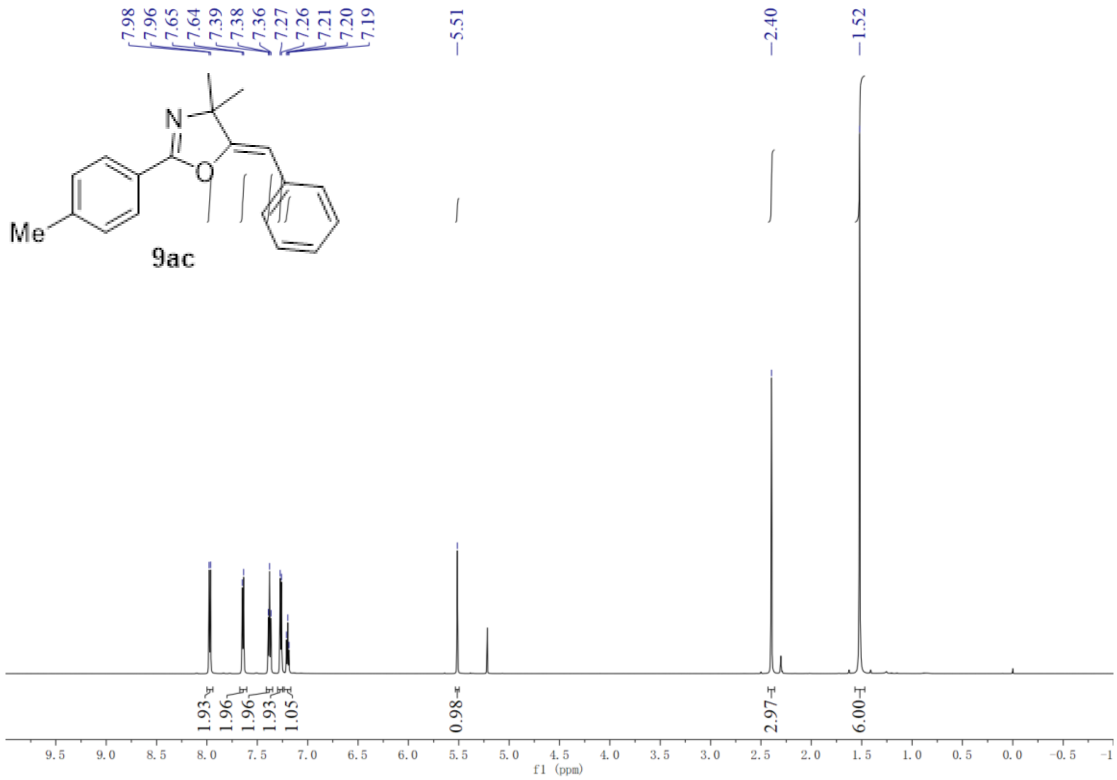

**Supplementary Figure S123.** $^{1}$H NMR (600 MHz, $CDCl_3$) Spectrum of Compound **9ac**

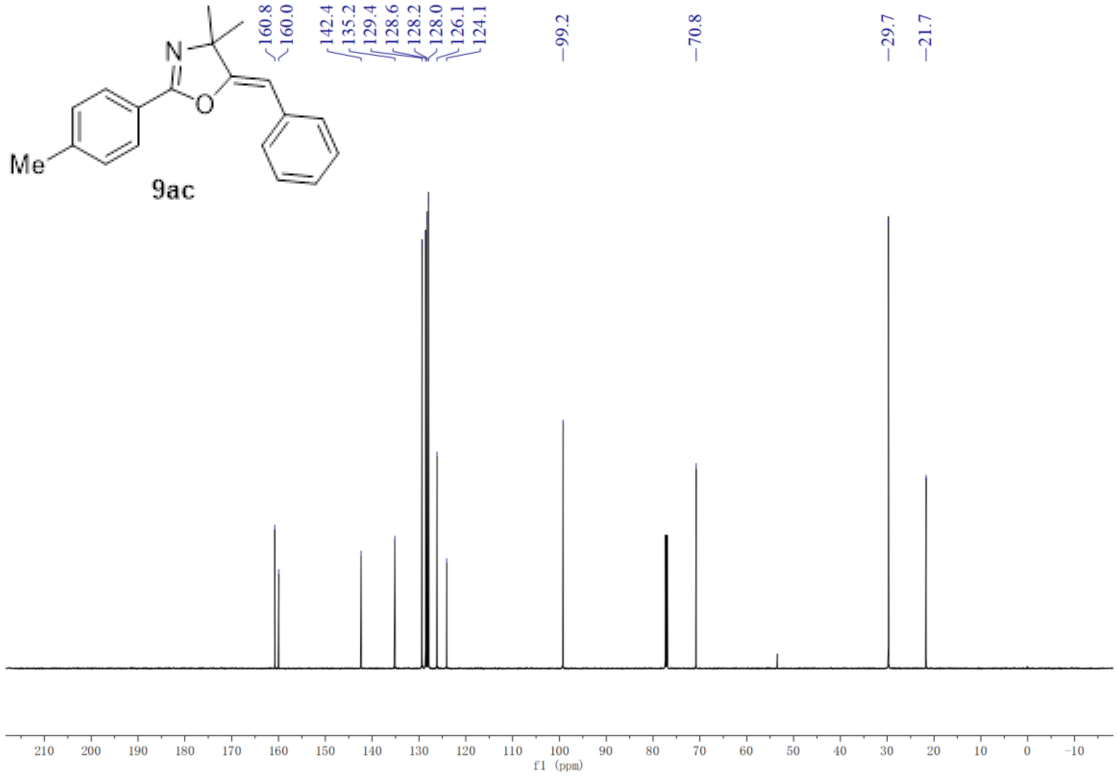

**Supplementary Figure S124.** $^{13}$C NMR (150 MHz, $CDCl_3$) Spectrum of Compound **9ac**

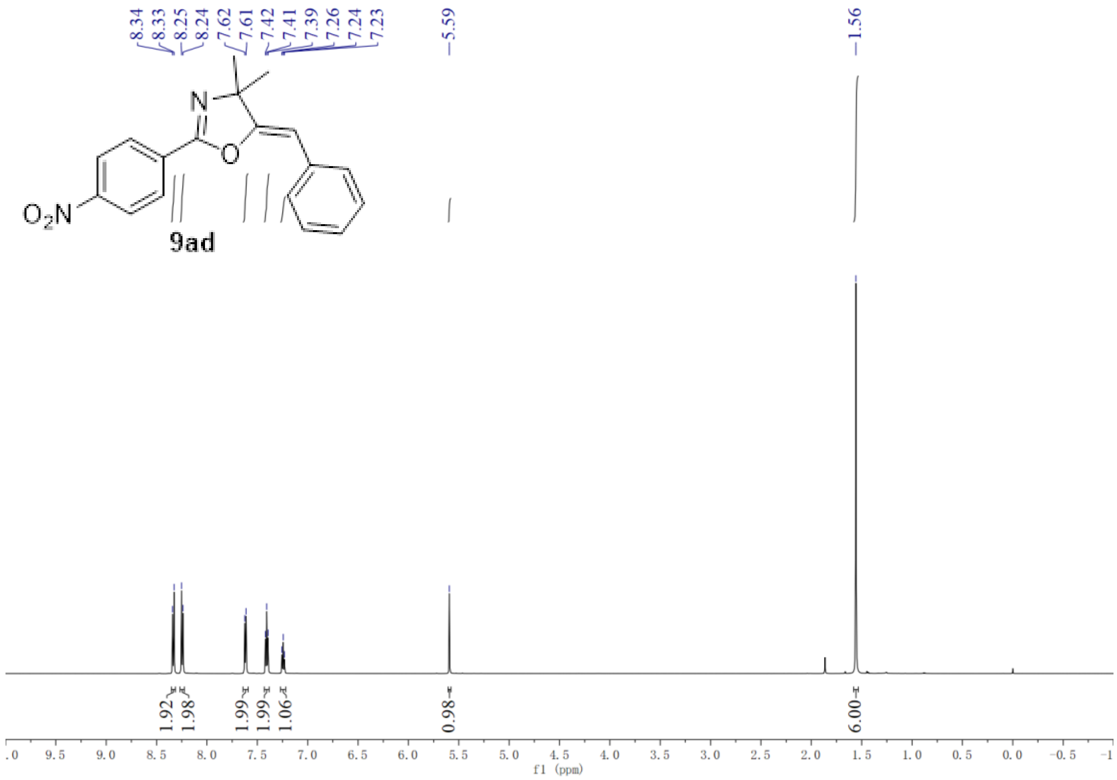

**Supplementary Figure S125.** $^{1}H$ NMR (600 MHz, $CDCl_3$) Spectrum of Compound **9ad**

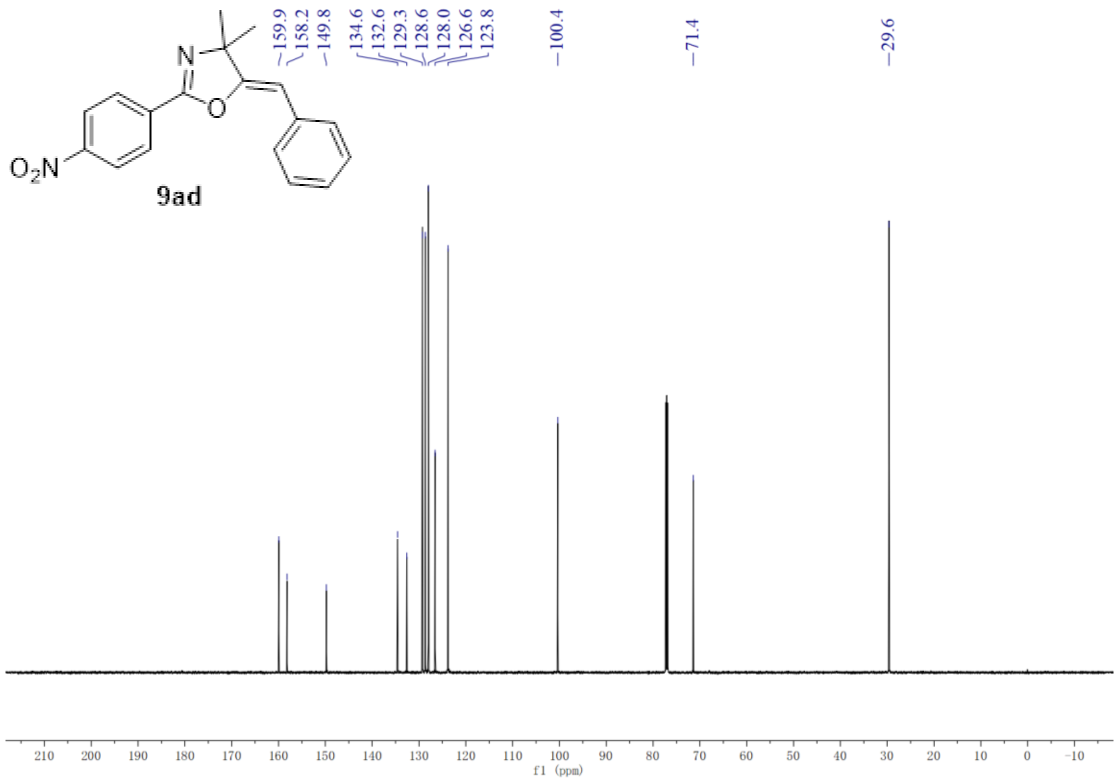

**Supplementary Figure S126.** $^{13}C$ NMR (150 MHz, $CDCl_3$) Spectrum of Compound **9ad**

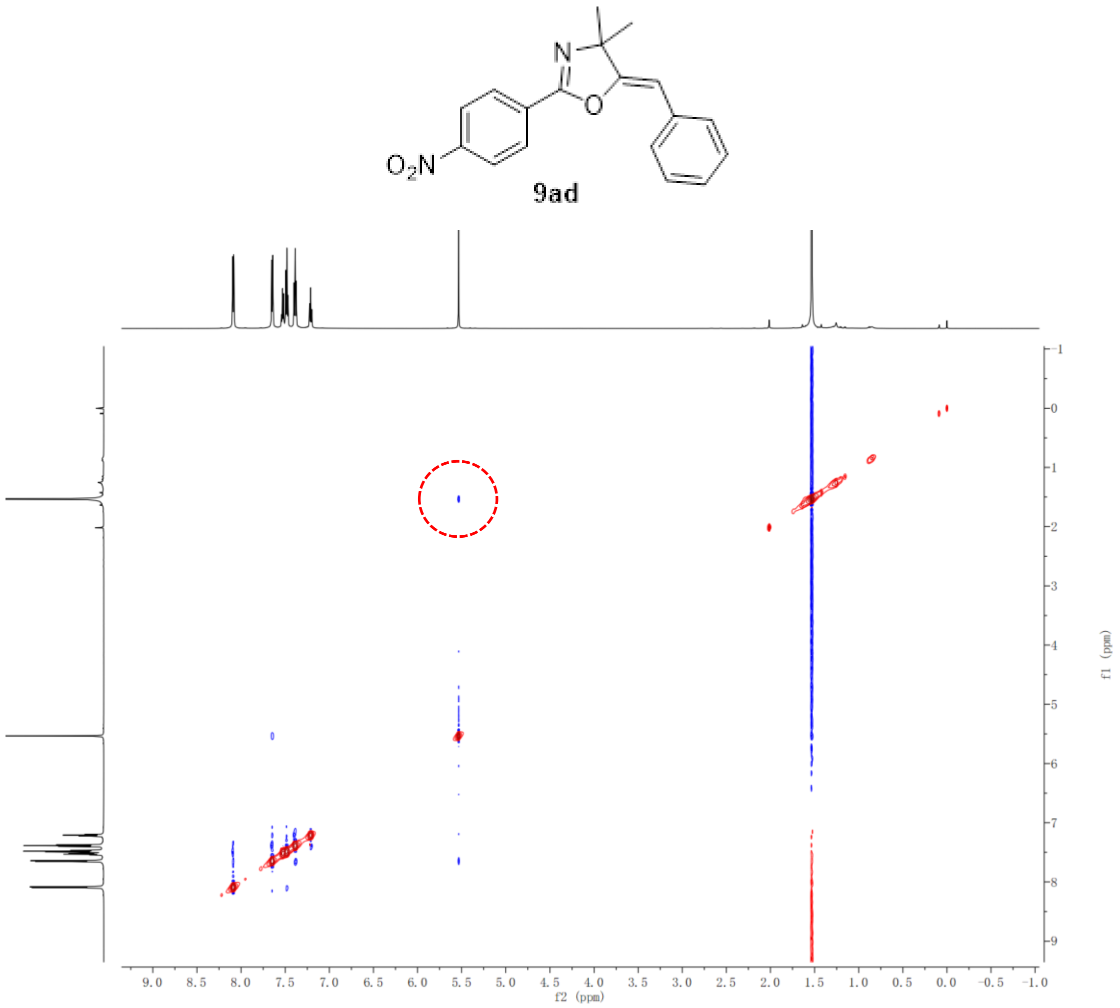


**Supplementary Figure S127.** NOESY Spectrum of Compound **9ad**

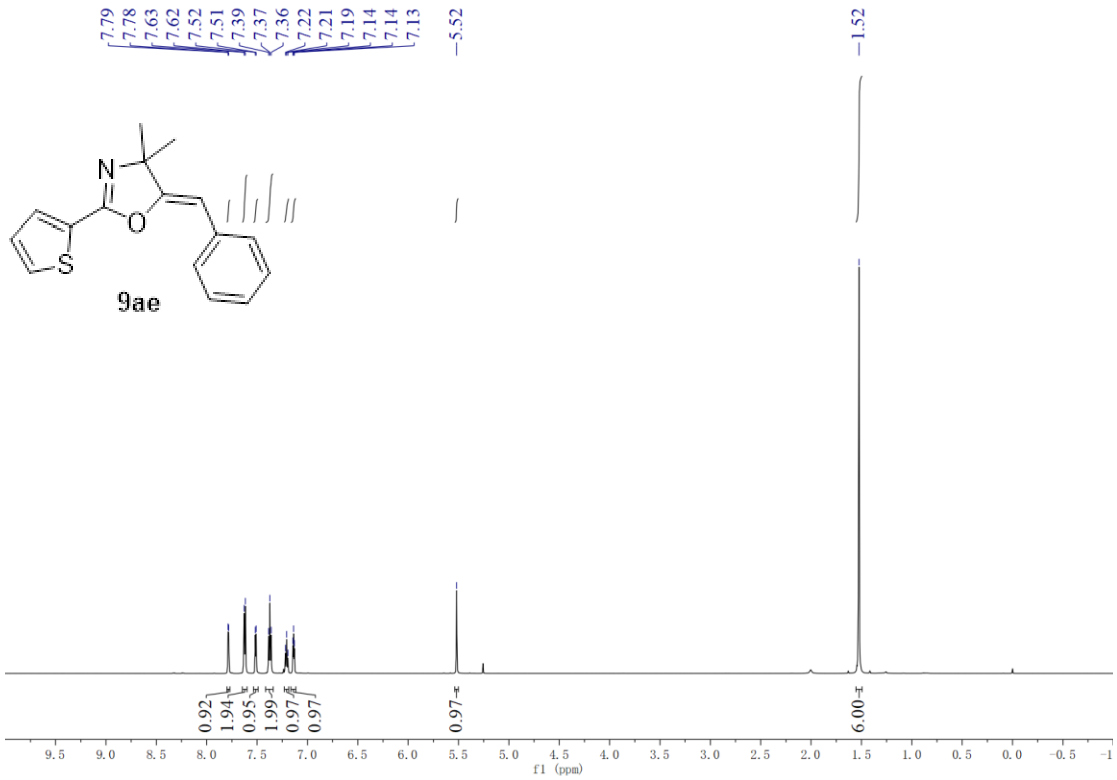


**Supplementary Figure S128.** $^{1}$H NMR (600 MHz, $CDCl_3$) Spectrum of Compound **9ae**

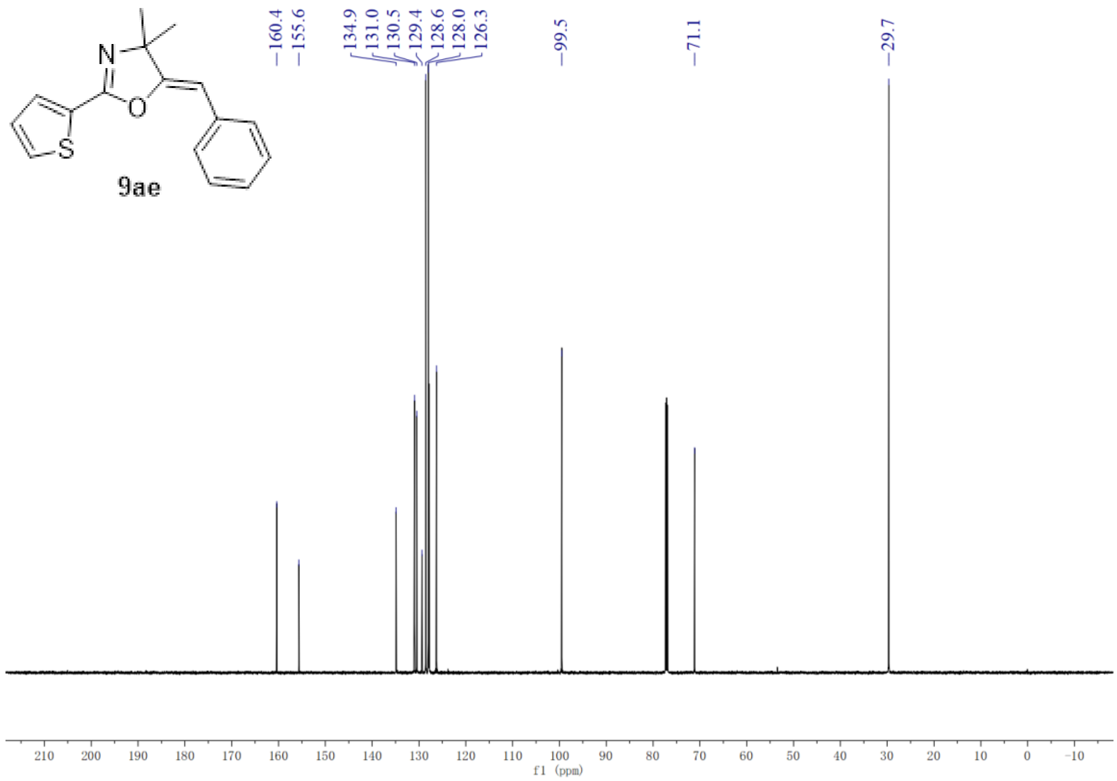


**Supplementary Figure S129.** $^{13}$C NMR (150 MHz, $CDCl_3$) Spectrum of Compound **9ae**

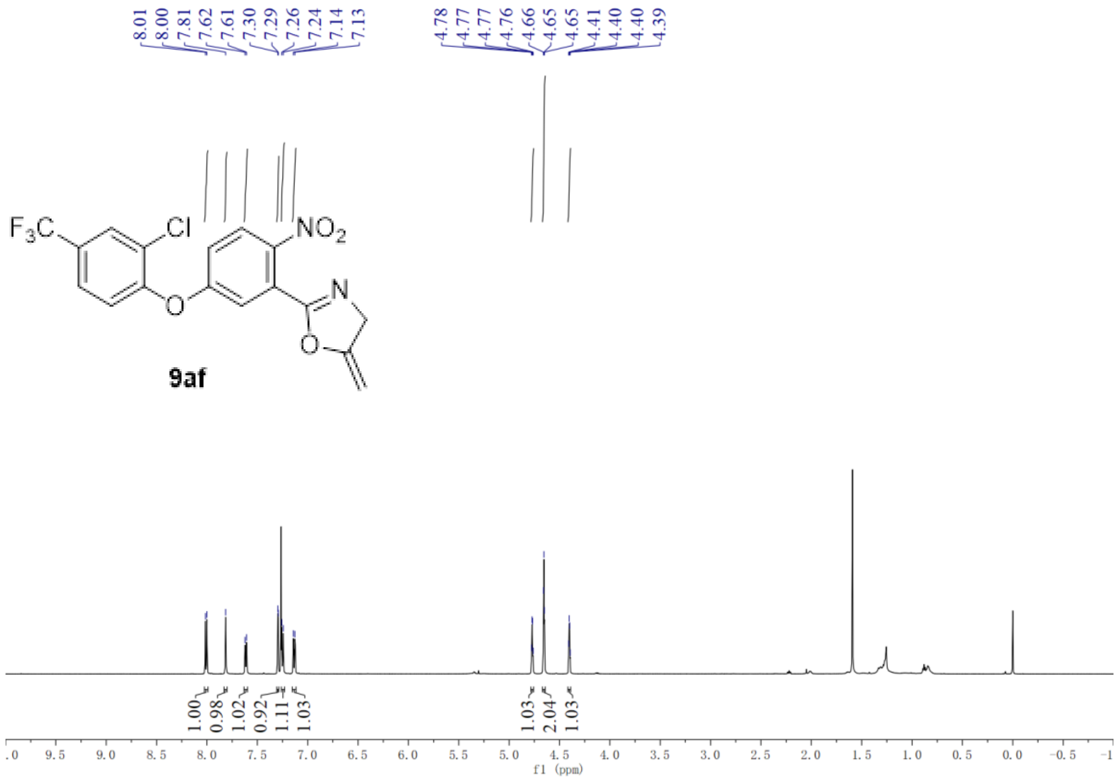

**Supplementary Figure S130.** $^{1}$H NMR (600 MHz, $CDCl_3$) Spectrum of Compound **9af**

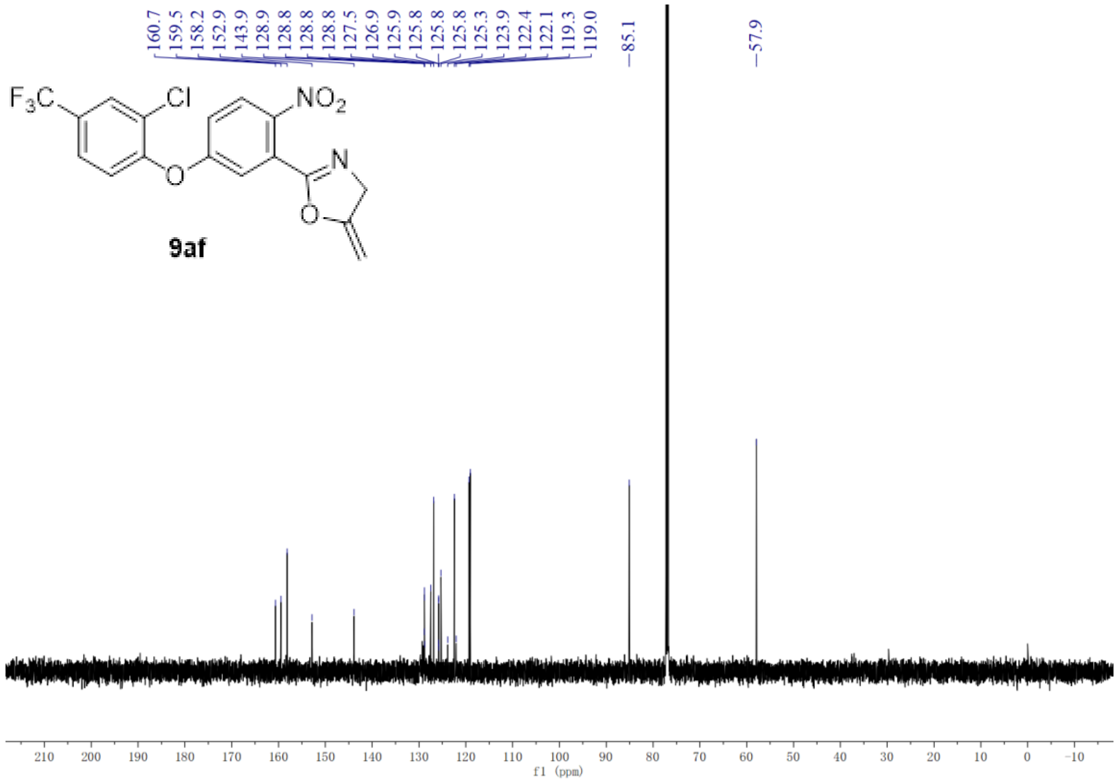

**Supplementary Figure S131.** $^{13}$C NMR (150 MHz, $CDCl_3$) Spectrum of Compound **9af**

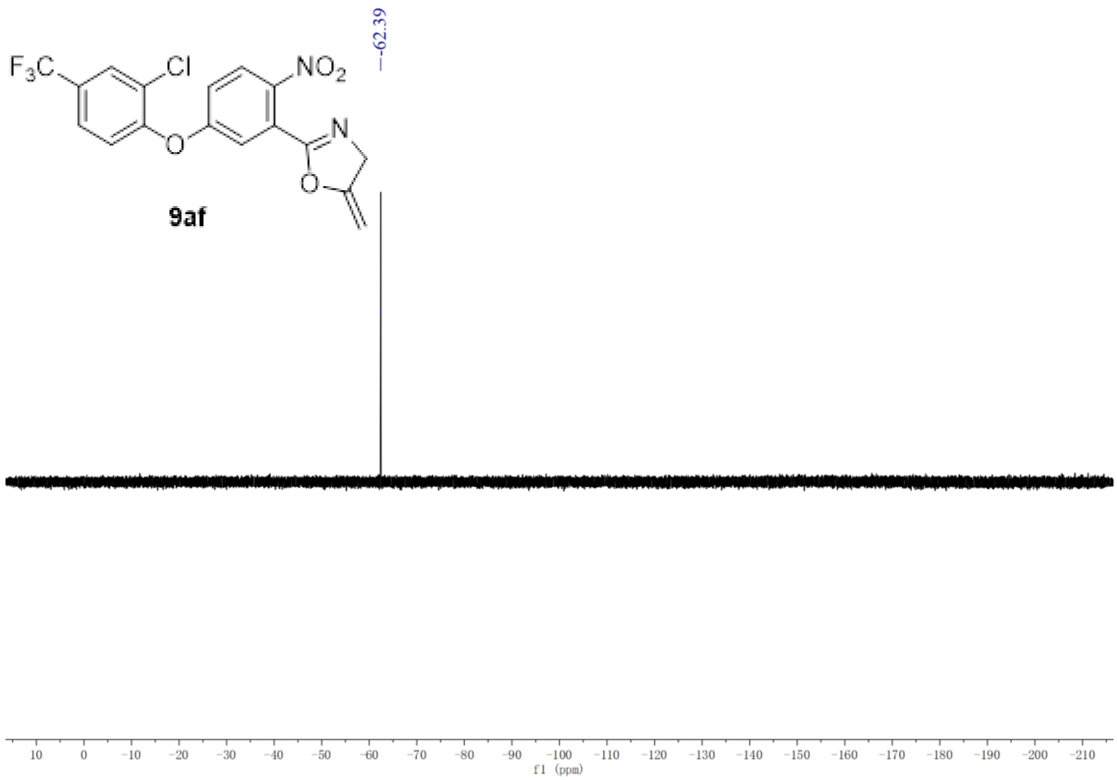


**Supplementary Figure S132.** $^{19}$F NMR (565 MHz, $CDCl_3$) Spectrum of Compound **9af**

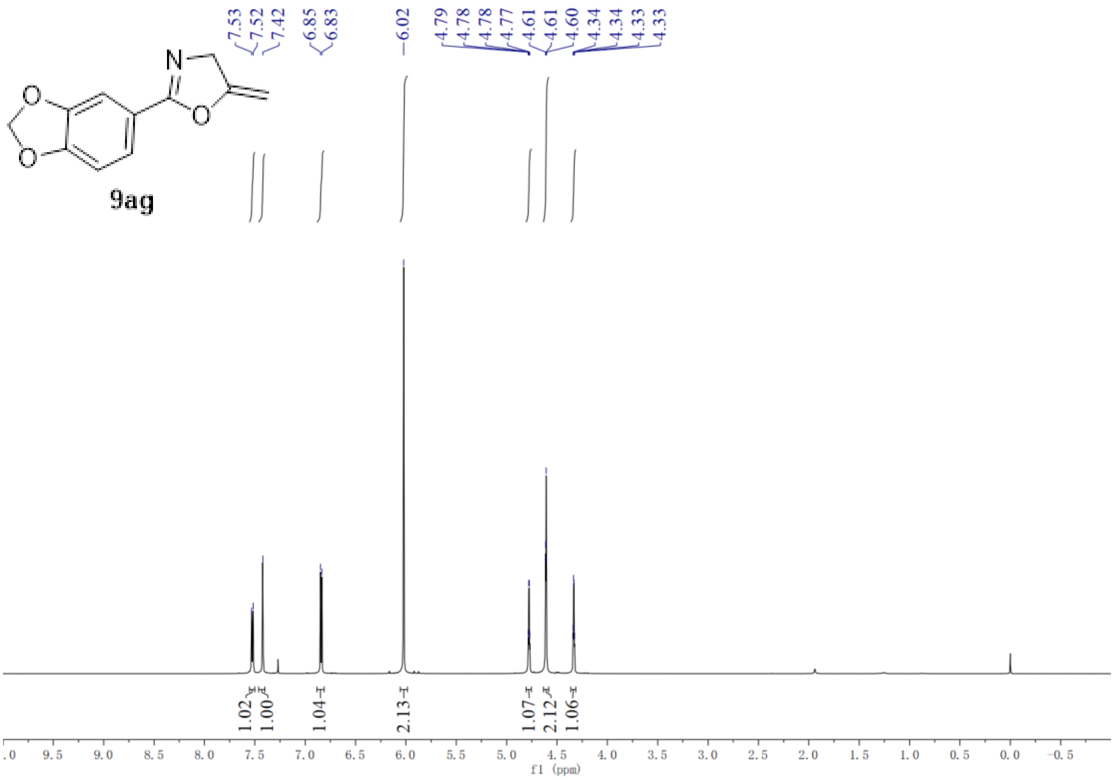


**Supplementary Figure S133.** $^{1}$H NMR (600 MHz, $CDCl_3$) Spectrum of Compound **9ag**

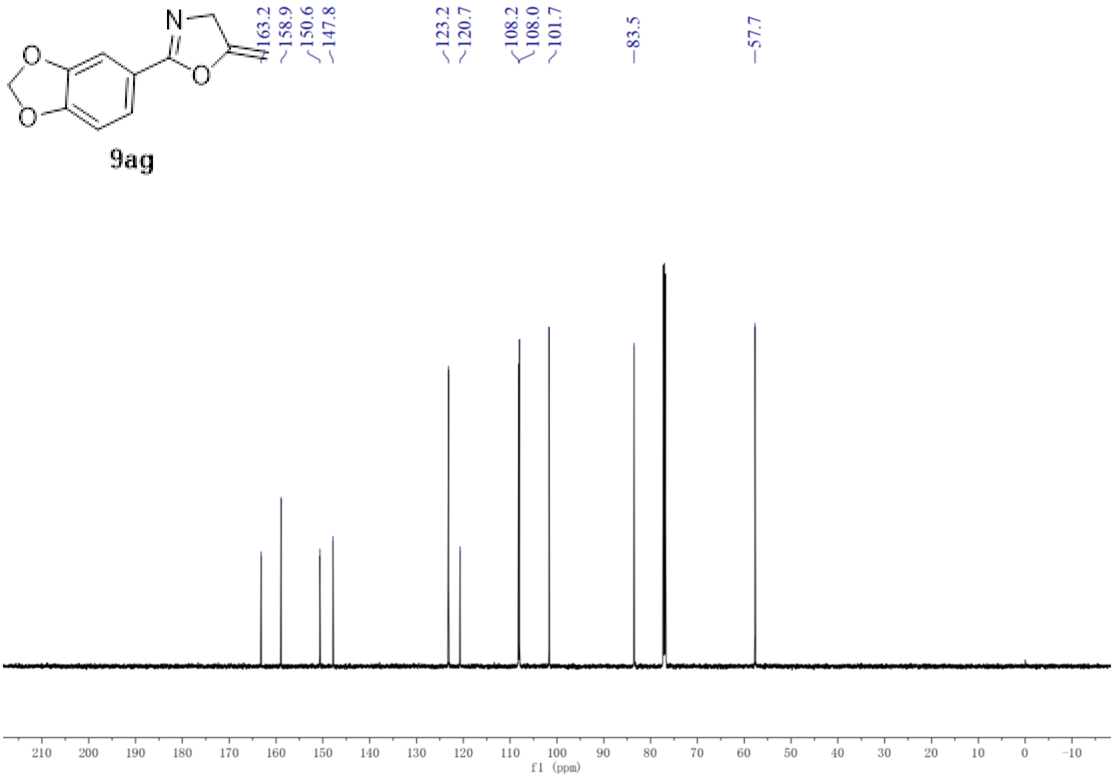


**Supplementary Figure S134.** $^{13}$C NMR (150 MHz, $CDCl_3$) Spectrum of Compound **9ag**

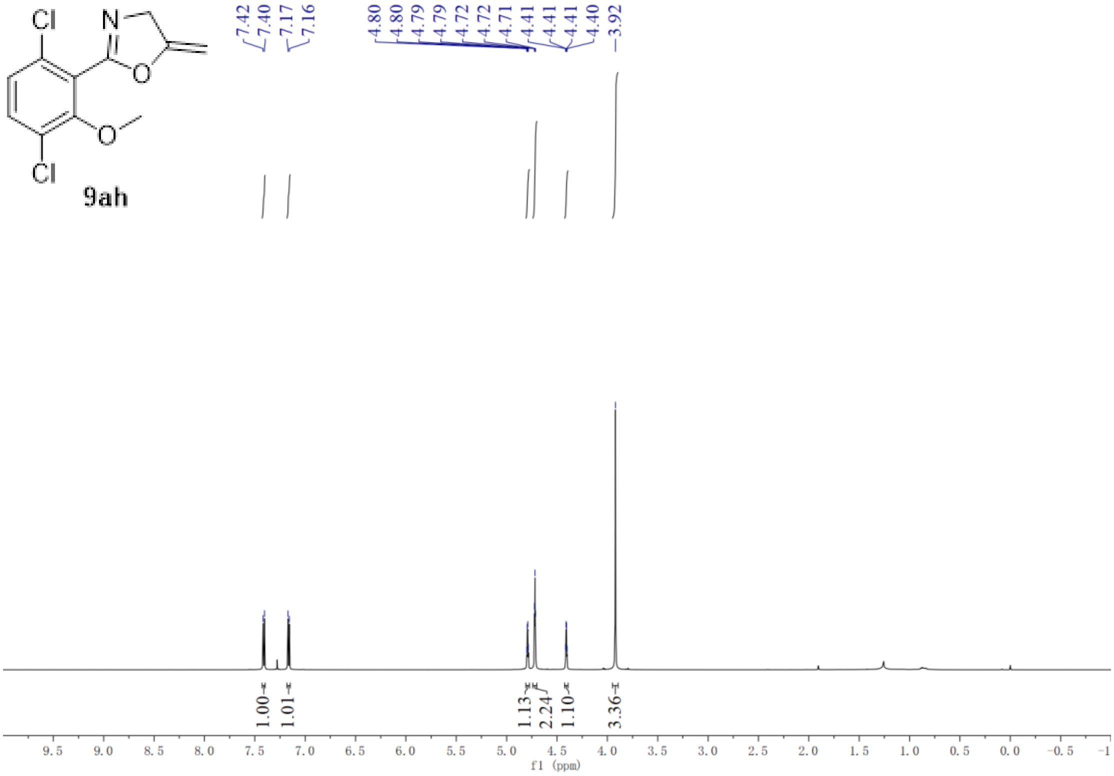


**Supplementary Figure S135.** $^{1}$H NMR (600 MHz, $CDCl_3$) Spectrum of Compound **9ah**

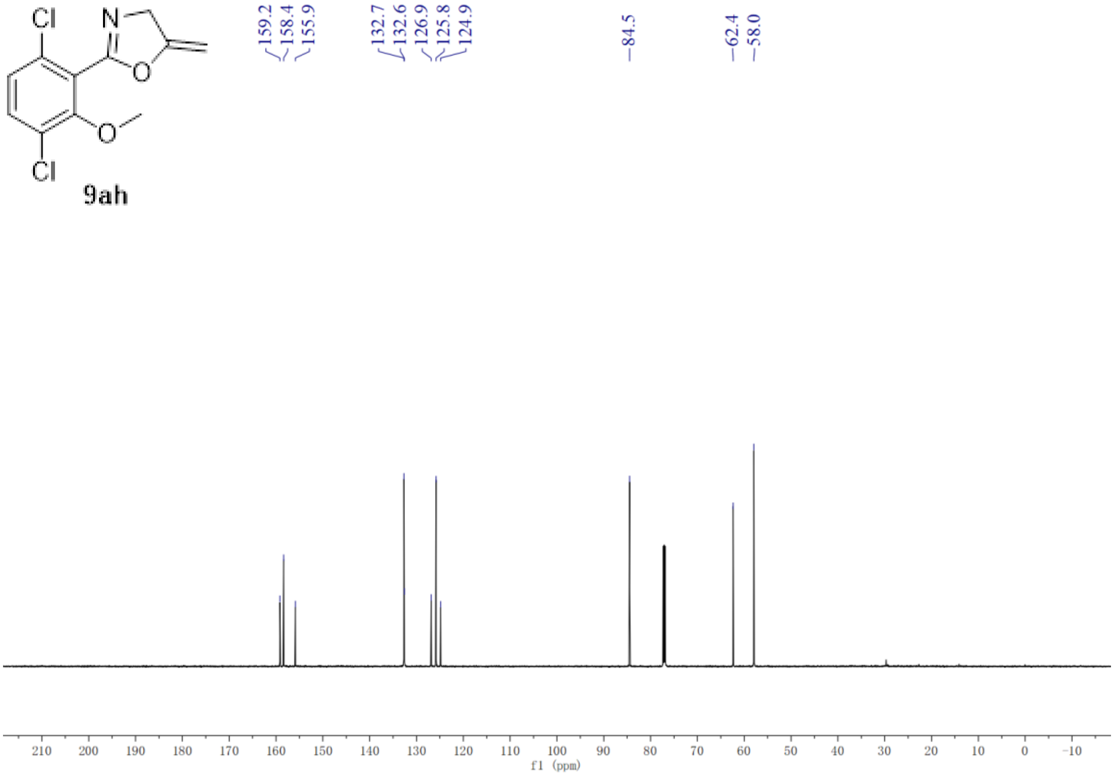


**Supplementary Figure S136.** $^{13}$C NMR (150 MHz, $CDCl_3$) Spectrum of Compound **9ah**

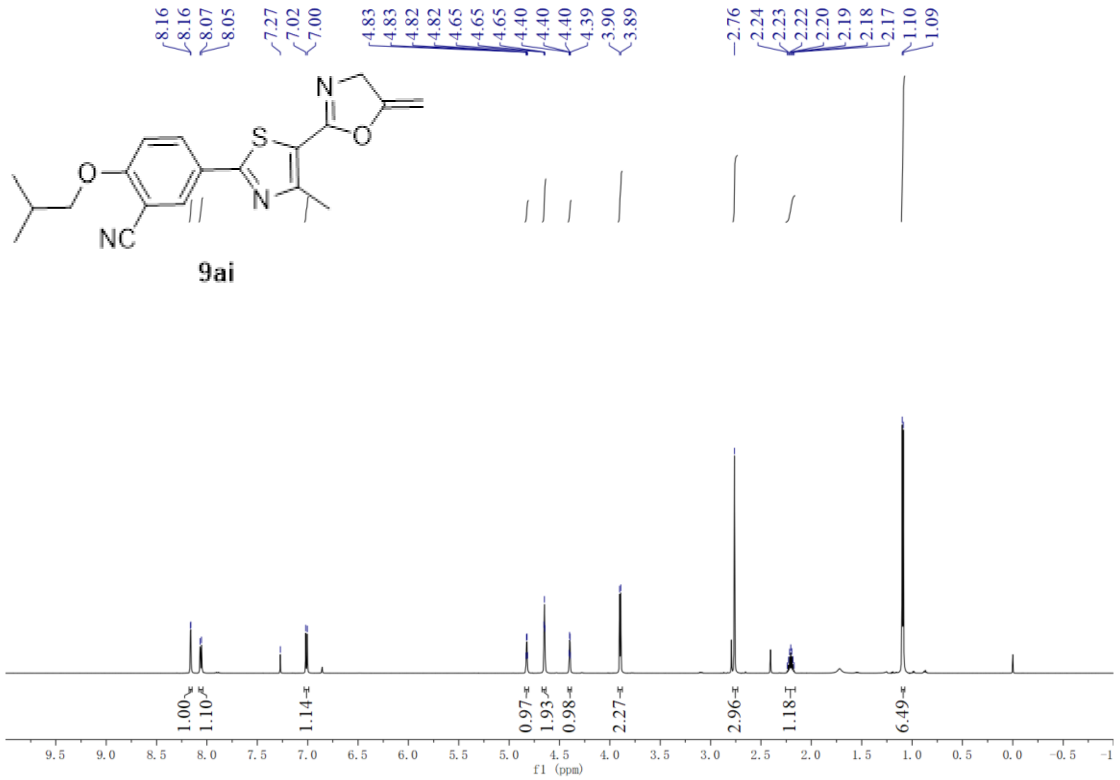


**Supplementary Figure S137.** $^{1}H$ NMR (600 MHz, $CDCl_3$) Spectrum of Compound **9ai**

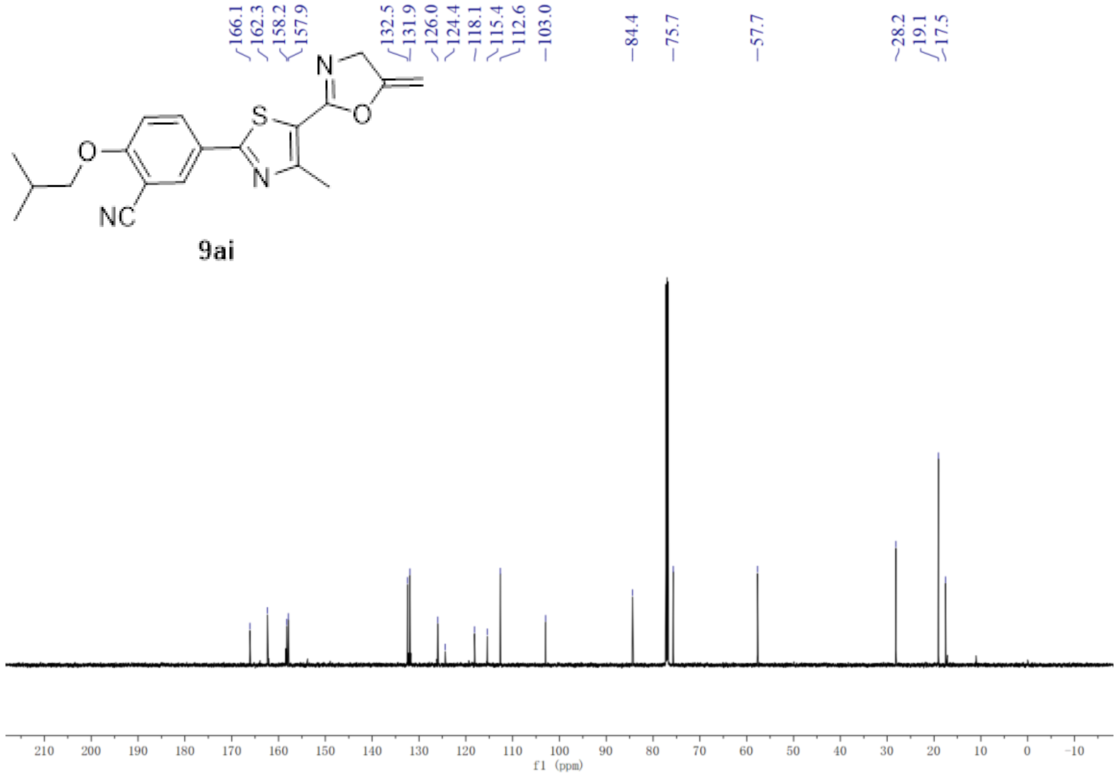


**Supplementary Figure S138.** $^{13}C$ NMR (150 MHz, $CDCl_3$) Spectrum of Compound **9ai**

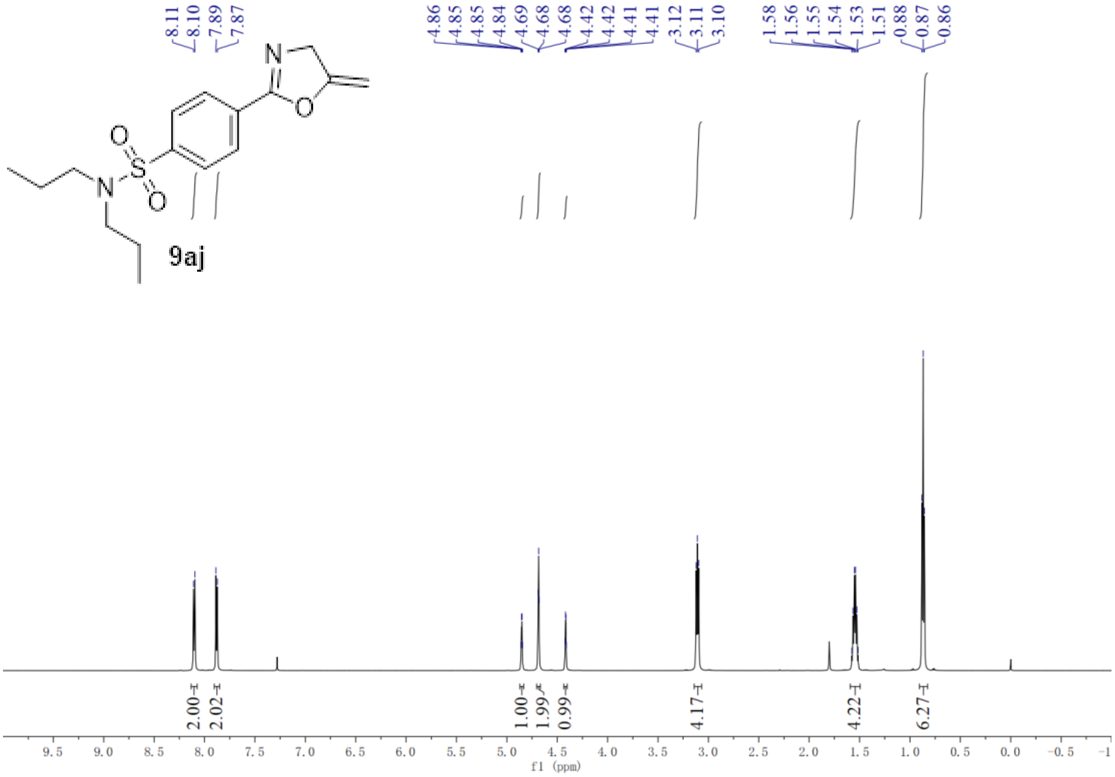


**Supplementary Figure S139.** $^{1}H$ NMR (600 MHz, $CDCl_3$) Spectrum of Compound **9aj**

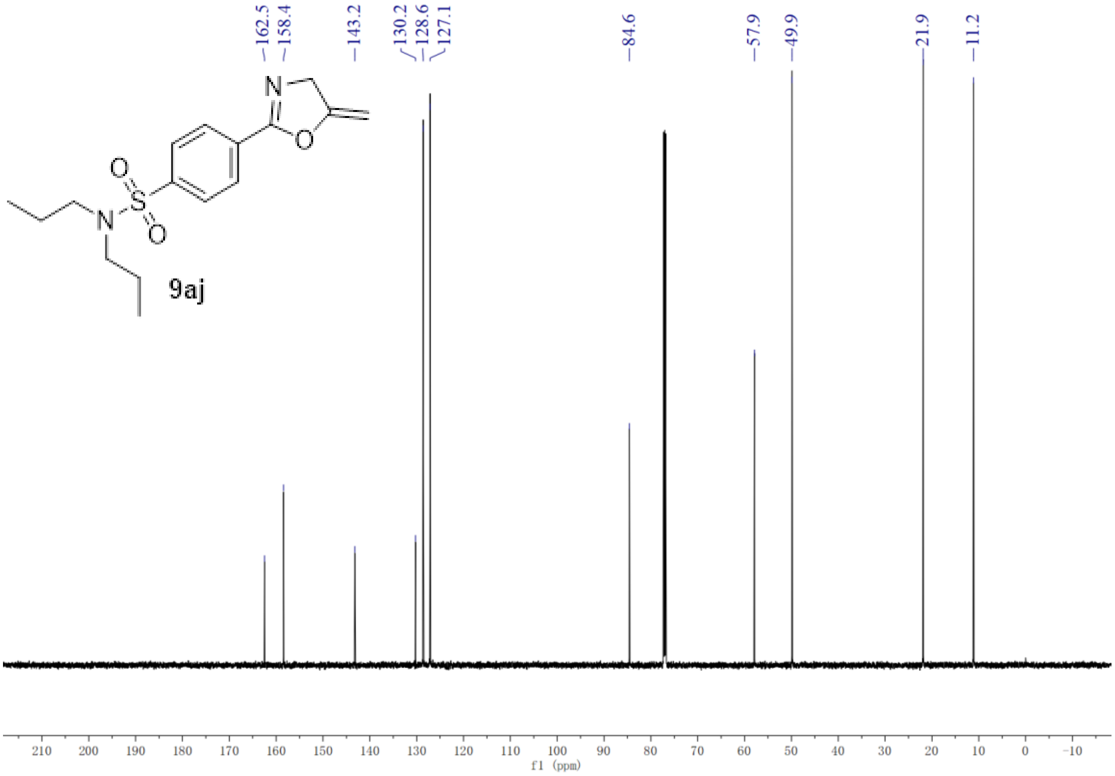


**Supplementary Figure S140.** $^{13}C$ NMR (150 MHz, $CDCl_3$) Spectrum of Compound **9aj**

**Supplementary Note 3: Computational Details**

All calculations were carried out with the Gaussian 09 programs.[s1] The geometries of all the species were fully optimized by using density functional theory (DFT)[s2] and the B3LYP method[s3] with the 6-31G(d) basis set for all atoms except for Ag. The Lanl2dz [s4] basis set was used for Ag (BSI). Vibrational frequency calculations carried out at the B3LYP/6-31G (d, p) level of theory were used to characterize all of the stationary points as either minima (the number of imaginary frequencies (NIMAG=0) or transition states (NIMAG=1)). The relative energies were corrected for the vibrational zero-point energies (ZPE, not scaled). The convergence criteria were used as default and the vibrational analysis was also carried out at standard conditions (1 atm., 298.15 K). The solvent effect was taken into account by single-point calculations with integral equation formalism polarizable continuum model (IEFPCM) in DCM at the M06[s6]/6-311++G (d, p) (for Ag, Lanl2tz(f),[s7] (BSII) was used). The radii and nonelectrostatic terms were taken from Truhlar and co-workers' universal solvation model (SMD).[s8] Unless stated otherwise, all the relative free energies discussed in the main text are calculated in solution ($\Delta G_{sol}$), which were obtained by M06/SMD/6-311++G (d, p) //B3LYP/6-31G (d) (Lanl2dz).

(s1) M. J. Frisch, G. W. Trucks, H. B. Schlegel, G. E. Scuseria,M. A. Robb, J. R. Cheeseman, G. Scalmani, V. Barone, B. Mennucci, G. A. Petersson, H. Nakatsuji, M. Caricato, X. Li, H. P. Hratchian,A. F. Izmaylov, J. Bloino, G. Zheng, J. L. Sonnenberg, M. Hada,M. Ehara, K. Toyota, R. Fukuda, J. Hasegawa, M. Ishida, T. Nakajima,Y. Honda, O. Kitao, H. Nakai, T. Vreven, J. A. Montgomery, Jr.,J. E. Peralta, F. Ogliaro, M. Bearpark, J. J. Heyd, E. Brothers,K. N. Kudin, V. N. Staroverov, T. Keith, R. Kobayashi, J. Normand,K. Raghavachari, A. Rendell, J. C. Burant, S. S. Iyengar, J. Tomasi,M. Cossi, N. Rega, J. M. Millam, M. Klene, J. E. Knox, J. B. Cross,V. Bakken, C. Adamo, J. Jaramillo, R. Gomperts, R. E. Stratmann,O. Yazyev, A. J. Austin, R. Cammi, C. Pomelli, J. W. Ochterski,R. L. Martin, K. Morokuma, V. G. Zakrzewski, G. A. Voth,P. Salvador, J. J. Dannenberg, S. Dapprich, A. D. Daniels,O. Farkas, J. B. Foresman, J. V. Ortiz, J. Cioslowski,and D. J. Fox, Gaussian 09, D.01, Gaussian, Inc., Wallingford CT, **2013**.

(s2) Parr, R. G.; Yang, W. *Density-functional Theory of Atoms and Molecules*; Oxford University Press: New York, **1989**.

(s3) (a) A. D. Becke, *J. Chem. Phys.* **1993**, *98*, 5648–5652;(b) C. Lee, W. Yang, R. G. Parr *Phys. Rev. B* **1988**, *37*, 785–789.

(s4) (a) W. R. Wadt and P. J. Hay, *J. Chem. Phys.*, **1985**, *82*, 284-298; (b) P. J. Hay and W. R. Wadt, *J. Chem. Phys.*, **1985**, *82*, 299-310.

(s5) W. Ehlers, M. Böhme, S. Dapprich, A. Gobbi, A. Höllwarth, V. Jonas, K. F. Köhler, R. Stegmann, A.Veldkamp and G. Frenking *Chem. Phys. Lett.*, **1993**, *208*, 111-114.

(s6) (a) Y. Zhao, N. E. Schultz, D. G. Truhlar, *J. Chem. Theory. Comput.* **2006**, *2*, 364-382. (b) Y. Zhao, D. G. Truhlar, *J. Phys. Chem. A.* **2006**, *110*, 13126-13130.

(s7) L. E. Roy, P. J. Hay and R. L. Martin, *J. Chem. Theory Comput.*, **2008**, *4*, 1029-1103.

(s8) (a) Y. Zhao, D. G. Truhlar, *J. Phys. Chem. Lett.* **2008**, *112*, 1095-1099; (b) D. Jacquemin, E. A. Perpète, I. Ciofini, C. Adamo, R. Valero, Y. Zhao, D. Truhlar, *J. Chem. Theory Comput.* **2010**, *6*, 2071-2085.

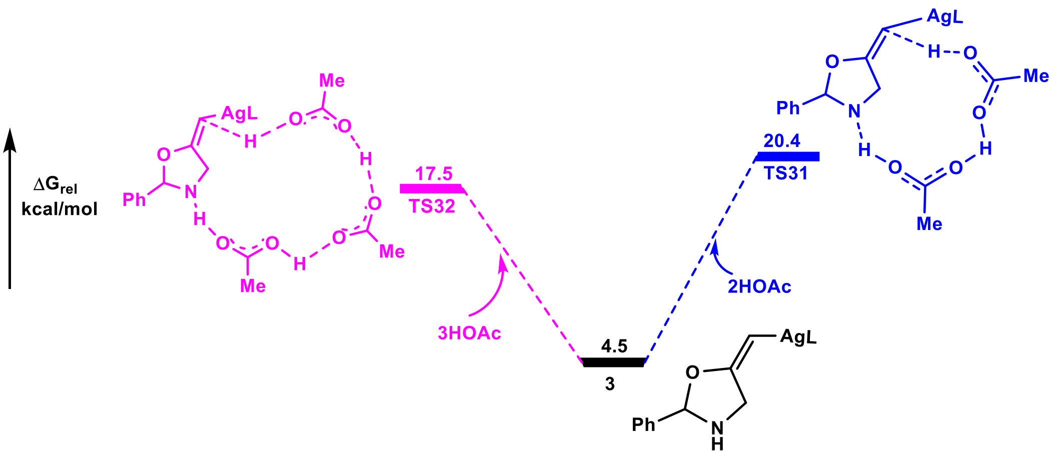


**Supplementary Figure S141.** Energy profile calculated of two and three-molecule HOAc-assisted 1,4-H shift. Relative energies are in kcal/mol.

**The Cartesian coordinates of the stationary points**

$L_2Ag$

| | | | |
|---|---|---|---|
| 47 | -0.000036000 | 0.000083000 | -0.810879000 |
| 16 | -2.724577000 | 1.562186000 | -2.044190000 |
| 16 | 2.724190000 | -1.562560000 | -2.043701000 |
| 7 | -3.131535000 | -0.186907000 | -0.329501000 |
| 7 | 3.131582000 | 0.186883000 | -0.329473000 |
| 6 | 2.894488000 | 1.216602000 | 0.675475000 |
| 6 | -2.894224000 | -1.216284000 | 0.675731000 |
| 6 | -4.429377000 | 0.236095000 | -0.697860000 |
| 6 | -2.092647000 | 0.405996000 | -0.946205000 |
| 6 | 2.092538000 | -0.406000000 | -0.945928000 |
| 6 | -2.780849000 | -0.821921000 | 2.024445000 |
| 6 | -4.390197000 | 1.211846000 | -1.648694000 |
| 6 | 2.781040000 | 0.822618000 | 2.024287000 |
| 6 | -5.540347000 | 1.907029000 | -2.328498000 |
| 1 | -6.026832000 | 1.199256000 | -3.015937000 |
| 1 | -5.144941000 | 2.712626000 | -2.957149000 |
| 6 | 2.378650000 | 3.159105000 | 2.576068000 |
| 1 | 2.175131000 | 3.921870000 | 3.322278000 |
| 6 | -2.779770000 | -2.555656000 | 0.253002000 |
| 6 | 2.780076000 | 2.555856000 | 0.252339000 |
| 6 | 2.514156000 | 1.827930000 | 2.962486000 |
| 1 | 2.416404000 | 1.563702000 | 4.011080000 |
| 6 | -2.513944000 | -1.826956000 | 2.962933000 |
| 1 | -2.416277000 | -1.562420000 | 4.011458000 |
| 6 | -6.597894000 | 2.490316000 | -1.370653000 |
| 1 | -6.106141000 | 3.164006000 | -0.656309000 |

| | | | |
|---|---|---|---|
| 1 | -7.272534000 | 3.112656000 | -1.970969000 |
| 6 | 2.515637000 | 3.516360000 | 1.237390000 |
| 1 | 2.420275000 | 4.559266000 | 0.950365000 |
| 6 | 2.938909000 | -0.624734000 | 2.486186000 |
| 1 | 3.245361000 | -1.229454000 | 1.626561000 |
| 6 | -2.939227000 | -2.988731000 | -1.202334000 |
| 1 | -3.168202000 | -2.103166000 | -1.803951000 |
| 6 | -2.378331000 | -3.158233000 | 2.576898000 |
| 1 | -2.174801000 | -3.920773000 | 3.323335000 |
| 6 | 4.429320000 | -0.236401000 | -0.697872000 |
| 6 | -2.515279000 | -3.515871000 | 1.238319000 |
| 1 | -2.419858000 | -4.558849000 | 0.951575000 |
| 6 | -2.938862000 | 0.625538000 | 2.485961000 |
| 1 | -3.245386000 | 1.230009000 | 1.626187000 |
| 6 | -5.664040000 | -0.384073000 | -0.096062000 |
| 1 | -5.364066000 | -1.055229000 | 0.712815000 |
| 1 | -6.146004000 | -1.018507000 | -0.854495000 |
| 6 | 4.037773000 | -0.766304000 | 3.558573000 |
| 1 | 4.997012000 | -0.366627000 | 3.212410000 |
| 1 | 4.181315000 | -1.822570000 | 3.812311000 |
| 1 | 3.772304000 | -0.240190000 | 4.482178000 |
| 6 | 5.664161000 | 0.383549000 | -0.096226000 |
| 1 | 5.364384000 | 1.055022000 | 0.712459000 |
| 1 | 6.146367000 | 1.017601000 | -0.854821000 |
| 6 | 4.389885000 | -1.212323000 | -1.648523000 |
| 6 | 1.604846000 | -1.203585000 | 2.997324000 |
| 1 | 1.245164000 | -0.652656000 | 3.873963000 |
| 1 | 1.734626000 | -2.251541000 | 3.290681000 |
| 1 | 0.824804000 | -1.159928000 | 2.229761000 |
| 6 | -7.441205000 | 1.451894000 | -0.614762000 |
| 1 | -7.914734000 | 0.772447000 | -1.339293000 |
| 1 | -8.263116000 | 1.980271000 | -0.116229000 |
| 6 | -1.634243000 | -3.591182000 | -1.759442000 |
| 1 | -0.801952000 | -2.881863000 | -1.679383000 |
| 1 | -1.758219000 | -3.852928000 | -2.816270000 |
| 1 | -1.350822000 | -4.502298000 | -1.220657000 |
| 6 | 2.939567000 | 2.988498000 | -1.203120000 |
| 1 | 3.168427000 | 2.102738000 | -1.804489000 |
| 6 | -6.699421000 | 0.623396000 | 0.444955000 |
| 1 | -7.441174000 | 0.048053000 | 1.012147000 |
| 1 | -6.210858000 | 1.294305000 | 1.163934000 |
| 6 | 5.539889000 | -1.907811000 | -2.328268000 |
| 1 | 6.026562000 | -1.200181000 | -3.015722000 |
| 1 | 5.144315000 | -2.713345000 | -2.956893000 |

| | | | |
|---|---|---|---|
| 6 | 6.597268000 | -2.491285000 | -1.370346000 |
| 1 | 7.271777000 | -3.113841000 | -1.970586000 |
| 1 | 6.105330000 | -3.164796000 | -0.655961000 |
| 6 | 1.634704000 | 3.590974000 | -1.760456000 |
| 1 | 0.802291000 | 2.881820000 | -1.680210000 |
| 1 | 1.758773000 | 3.852356000 | -2.817363000 |
| 1 | 1.351390000 | 4.502317000 | -1.222003000 |
| 6 | -4.114363000 | -3.972619000 | -1.373322000 |
| 1 | -3.940795000 | -4.907058000 | -0.828232000 |
| 1 | -4.244488000 | -4.225319000 | -2.431483000 |
| 1 | -5.054274000 | -3.544302000 | -1.008182000 |
| 6 | -1.604826000 | 1.204609000 | 2.996952000 |
| 1 | -1.245133000 | 0.653915000 | 3.873734000 |
| 1 | -1.734665000 | 2.252636000 | 3.290031000 |
| 1 | -0.824778000 | 1.160802000 | 2.229407000 |
| 6 | -4.037689000 | 0.767327000 | 3.558346000 |
| 1 | -4.996917000 | 0.367499000 | 3.212328000 |
| 1 | -4.181277000 | 1.823657000 | 3.811789000 |
| 1 | -3.772163000 | 0.241493000 | 4.482097000 |
| 6 | 7.440812000 | -1.453003000 | -0.614523000 |
| 1 | 8.262550000 | -1.981545000 | -0.115882000 |
| 1 | 7.914570000 | -0.773769000 | -1.339104000 |
| 6 | 4.114859000 | 3.972187000 | -1.374322000 |
| 1 | 3.941393000 | 4.906740000 | -0.829392000 |
| 1 | 4.245022000 | 4.224676000 | -2.432529000 |
| 1 | 5.054711000 | 3.543820000 | -1.009093000 |
| 6 | 6.699200000 | -0.624154000 | 0.445047000 |
| 1 | 7.441084000 | -0.048955000 | 1.012213000 |
| 1 | 6.210382000 | -1.294818000 | 1.164077000 |

Zero-point correction= 0.862529 (Hartree/Particle)
Thermal correction to Energy= 0.910351
Thermal correction to Enthalpy= 0.911296
Thermal correction to Gibbs Free Energy= 0.778442
Sum of electronic and zero-point Energies= -2607.415116
Sum of electronic and thermal Energies= -2607.367293
Sum of electronic and thermal Enthalpies= -2607.366349
Sum of electronic and thermal Free Energies= -2607.499203

TS1（1 imaginary frequencies, -51.8 i $cm^{-1}$）

| | | | |
|---|---|---|---|
| 47 | -0.317126000 | -0.818399000 | -0.031059000 |
| 16 | -1.766820000 | 2.151048000 | 0.876855000 |
| 16 | 1.702530000 | 1.208563000 | -1.930503000 |
| 7 | -3.235949000 | 0.385520000 | -0.065766000 |
| 7 | 3.130548000 | 0.416945000 | -0.068796000 |
| 6 | 3.768720000 | -0.462213000 | 0.909690000 |

| | | | |
|---|---|---|---|
| 6 | -3.679925000 | -0.868856000 | -0.664601000 |
| 6 | -4.123679000 | 1.450431000 | 0.217653000 |
| 6 | -1.934728000 | 0.575566000 | 0.221088000 |
| 6 | 2.129308000 | -0.057551000 | -0.845303000 |
| 6 | -4.027654000 | -1.939207000 | 0.184144000 |
| 6 | -3.470340000 | 2.523018000 | 0.746165000 |
| 6 | 4.888309000 | -1.220327000 | 0.503480000 |
| 6 | -4.029671000 | 3.862572000 | 1.148023000 |
| 1 | -4.302776000 | 4.420376000 | 0.239858000 |
| 1 | -3.237293000 | 4.445719000 | 1.630775000 |
| 6 | 4.959938000 | -2.161804000 | 2.748640000 |
| 1 | 5.425344000 | -2.829400000 | 3.468203000 |
| 6 | -3.731565000 | -0.954549000 | -2.070937000 |
| 6 | 3.244469000 | -0.519342000 | 2.217623000 |
| 6 | 5.463005000 | -2.074014000 | 1.454280000 |
| 1 | 6.322569000 | -2.675434000 | 1.174501000 |
| 6 | -4.437195000 | -3.131209000 | -0.427330000 |
| 1 | -4.713065000 | -3.977941000 | 0.193670000 |
| 6 | -5.248552000 | 3.798482000 | 2.088296000 |
| 1 | -4.988016000 | 3.209410000 | 2.977752000 |
| 1 | -5.445152000 | 4.819404000 | 2.437660000 |
| 6 | 3.866551000 | -1.387201000 | 3.124341000 |
| 1 | 3.489077000 | -1.454901000 | 4.140150000 |
| 6 | 5.512943000 | -1.132577000 | -0.888403000 |
| 1 | 4.934373000 | -0.418803000 | -1.484321000 |
| 6 | -3.350588000 | 0.201760000 | -2.992621000 |
| 1 | -3.120802000 | 1.076407000 | -2.375485000 |
| 6 | -4.495169000 | -3.250651000 | -1.813003000 |
| 1 | -4.817059000 | -4.185264000 | -2.263499000 |
| 6 | 3.561352000 | 1.747573000 | -0.283670000 |
| 6 | -4.145244000 | -2.173370000 | -2.622927000 |
| 1 | -4.195490000 | -2.278307000 | -3.702488000 |
| 6 | -3.971657000 | -1.855299000 | 1.707377000 |
| 1 | -3.626421000 | -0.854354000 | 1.986210000 |
| 6 | -5.598285000 | 1.352527000 | -0.078336000 |
| 1 | -5.830456000 | 0.327526000 | -0.378978000 |
| 1 | -5.828544000 | 1.984329000 | -0.948941000 |
| 6 | 6.962329000 | -0.607635000 | -0.814013000 |
| 1 | 7.025771000 | 0.350699000 | -0.288352000 |
| 1 | 7.365871000 | -0.470592000 | -1.823566000 |
| 1 | 7.615719000 | -1.314846000 | -0.290984000 |
| 6 | 4.638386000 | 2.383867000 | 0.557380000 |
| 1 | 5.098543000 | 1.614372000 | 1.182856000 |
| 1 | 4.166699000 | 3.096055000 | 1.250827000 |

| | | | |
|---|---|---|---|
| 6 | 2.861433000 | 2.345244000 | -1.290823000 |
| 6 | 5.474789000 | -2.485479000 | -1.627237000 |
| 1 | 6.073215000 | -3.240308000 | -1.104104000 |
| 1 | 5.894958000 | -2.373205000 | -2.633248000 |
| 1 | 4.454123000 | -2.864763000 | -1.720705000 |
| 6 | -6.534355000 | 3.246654000 | 1.454476000 |
| 1 | -6.778991000 | 3.835610000 | 0.557800000 |
| 1 | -7.358575000 | 3.414900000 | 2.158456000 |
| 6 | -2.079600000 | -0.127542000 | -3.801347000 |
| 1 | -1.236871000 | -0.357174000 | -3.138769000 |
| 1 | -1.796450000 | 0.724148000 | -4.430230000 |
| 1 | -2.234636000 | -0.991306000 | -4.457656000 |
| 6 | 2.060273000 | 0.322695000 | 2.686012000 |
| 1 | 1.657148000 | 0.862409000 | 1.821942000 |
| 6 | -6.520538000 | 1.753714000 | 1.091183000 |
| 1 | -7.539403000 | 1.460224000 | 0.810699000 |
| 1 | -6.259719000 | 1.160234000 | 1.977481000 |
| 6 | 2.960782000 | 3.758320000 | -1.804131000 |
| 1 | 2.528765000 | 4.438756000 | -1.054858000 |
| 1 | 2.328807000 | 3.852972000 | -2.694431000 |
| 6 | 4.385533000 | 4.230490000 | -2.149446000 |
| 1 | 4.293678000 | 5.194628000 | -2.664382000 |
| 1 | 4.833289000 | 3.533815000 | -2.870554000 |
| 6 | 0.924794000 | -0.553917000 | 3.251486000 |
| 1 | 0.619344000 | -1.329202000 | 2.538494000 |
| 1 | 0.049997000 | 0.062964000 | 3.486907000 |
| 1 | 1.228172000 | -1.060617000 | 4.174277000 |
| 6 | -4.510753000 | 0.595809000 | -3.928104000 |
| 1 | -4.766826000 | -0.214763000 | -4.619104000 |
| 1 | -4.230745000 | 1.466817000 | -4.531072000 |
| 1 | -5.414881000 | 0.849239000 | -3.363718000 |
| 6 | -2.962286000 | -2.866515000 | 2.288617000 |
| 1 | -3.285419000 | -3.898569000 | 2.110966000 |
| 1 | -2.873393000 | -2.729857000 | 3.372426000 |
| 1 | -1.968892000 | -2.750215000 | 1.842367000 |
| 6 | -5.364590000 | -2.054840000 | 2.338677000 |
| 1 | -6.089812000 | -1.324661000 | 1.962954000 |
| 1 | -5.305282000 | -1.944975000 | 3.427346000 |
| 1 | -5.760566000 | -3.054541000 | 2.128133000 |
| 6 | 5.321513000 | 4.408448000 | -0.945340000 |
| 1 | 6.230801000 | 4.914164000 | -1.292748000 |
| 1 | 4.855015000 | 5.091235000 | -0.219332000 |
| 6 | 2.495771000 | 1.375097000 | 3.726209000 |
| 1 | 2.889736000 | 0.897981000 | 4.630525000 |

| | | | |
|---|---|---|---|
| 1 | 1.642085000 | 1.995913000 | 4.021613000 |
| 1 | 3.276024000 | 2.035368000 | 3.333174000 |
| 6 | 5.744046000 | 3.115523000 | -0.231029000 |
| 1 | 6.536479000 | 3.362798000 | 0.485906000 |
| 1 | 6.188892000 | 2.424608000 | -0.958347000 |
| 6 | 1.195189000 | -3.533953000 | -0.675928000 |
| 8 | 0.315654000 | -3.015531000 | 0.024615000 |
| 8 | 2.056461000 | -2.841440000 | -1.390573000 |
| 1 | 1.980153000 | -1.839341000 | -1.188747000 |
| 6 | 1.367252000 | -5.024849000 | -0.778451000 |
| 1 | 2.338825000 | -5.309056000 | -0.359956000 |
| 1 | 0.568737000 | -5.533563000 | -0.238277000 |
| 1 | 1.365930000 | -5.326959000 | -1.830529000 |

Zero-point correction= 0.923692 (Hartree/Particle)
Thermal correction to Energy= 0.977219
Thermal correction to Enthalpy= 0.978163
Thermal correction to Gibbs Free Energy= 0.829064
Sum of electronic and zero-point Energies= -2836.423018
Sum of electronic and thermal Energies= -2836.369491
Sum of electronic and thermal Enthalpies= -2836.368547
Sum of electronic and thermal Free Energies= -2836.517646

1

| | | | |
|---|---|---|---|
| 47 | 1.704558000 | -0.919579000 | -0.310354000 |
| 16 | 2.970815000 | 2.171582000 | -0.879918000 |
| 16 | -3.249400000 | 2.363550000 | 0.113607000 |
| 7 | 4.487881000 | 0.452229000 | 0.074948000 |
| 7 | -4.858617000 | 0.455234000 | -0.137721000 |
| 6 | -5.343546000 | -0.915277000 | -0.292230000 |
| 6 | 4.978709000 | -0.813460000 | 0.608656000 |
| 6 | 5.305604000 | 1.600687000 | -0.038489000 |
| 6 | 3.207138000 | 0.566002000 | -0.323318000 |
| 6 | -3.554921000 | 0.704200000 | -0.060647000 |
| 6 | 5.511854000 | -1.761932000 | -0.286946000 |
| 6 | 4.618907000 | 2.658574000 | -0.552898000 |
| 6 | -5.594119000 | -1.662687000 | 0.874555000 |
| 6 | 5.108741000 | 4.059441000 | -0.808882000 |
| 1 | 5.257445000 | 4.568185000 | 0.155365000 |
| 1 | 4.321802000 | 4.620043000 | -1.326008000 |
| 6 | -6.224565000 | -3.506364000 | -0.582131000 |
| 1 | -6.572605000 | -4.528870000 | -0.696517000 |
| 6 | 4.885263000 | -1.033671000 | 1.997433000 |
| 6 | -5.502519000 | -1.416814000 | -1.597782000 |
| 6 | -6.043711000 | -2.976501000 | 0.693419000 |
| 1 | -6.247088000 | -3.594534000 | 1.561800000 |

| | | | |
|---|---|---|---|
| 6 | 5.955640000 | -2.974028000 | 0.257139000 |
| 1 | 6.367785000 | -3.732597000 | -0.401312000 |
| 6 | 6.409089000 | 4.146120000 | -1.631675000 |
| 1 | 6.276073000 | 3.601152000 | -2.575713000 |
| 1 | 6.557869000 | 5.199753000 | -1.898375000 |
| 6 | -5.953595000 | -2.737913000 | -1.711638000 |
| 1 | -6.087219000 | -3.172098000 | -2.697122000 |
| 6 | -5.334948000 | -1.115590000 | 2.275812000 |
| 1 | -5.233031000 | -0.026193000 | 2.205953000 |
| 6 | 4.295716000 | -0.010691000 | 2.964978000 |
| 1 | 4.050211000 | 0.895866000 | 2.402446000 |
| 6 | 5.875339000 | -3.224621000 | 1.624139000 |
| 1 | 6.225897000 | -4.172474000 | 2.022547000 |
| 6 | -5.688182000 | 1.589508000 | -0.061132000 |
| 6 | 5.346845000 | -2.263742000 | 2.481994000 |
| 1 | 5.288727000 | -2.472125000 | 3.546082000 |
| 6 | 5.605145000 | -1.528595000 | -1.792741000 |
| 1 | 5.286467000 | -0.502580000 | -2.003793000 |
| 6 | 6.747380000 | 1.592290000 | 0.399907000 |
| 1 | 7.029167000 | 0.570494000 | 0.666306000 |
| 1 | 6.841064000 | 2.184063000 | 1.322627000 |
| 6 | -6.495439000 | -1.392486000 | 3.249127000 |
| 1 | -7.447836000 | -1.012606000 | 2.862803000 |
| 1 | -6.298924000 | -0.905057000 | 4.210147000 |
| 1 | -6.616785000 | -2.462676000 | 3.447678000 |
| 6 | -7.186544000 | 1.474703000 | -0.144875000 |
| 1 | -7.455382000 | 0.414844000 | -0.135775000 |
| 1 | -7.516881000 | 1.866583000 | -1.117815000 |
| 6 | -4.957033000 | 2.735775000 | 0.077485000 |
| 6 | -4.002747000 | -1.668443000 | 2.828434000 |
| 1 | -4.056294000 | -2.756098000 | 2.952655000 |
| 1 | -3.786803000 | -1.228436000 | 3.808581000 |
| 1 | -3.161869000 | -1.451515000 | 2.160246000 |
| 6 | 7.671799000 | 3.645832000 | -0.913294000 |
| 1 | 7.784155000 | 4.186268000 | 0.038630000 |
| 1 | 8.541236000 | 3.923355000 | -1.522030000 |
| 6 | 2.982791000 | -0.526744000 | 3.587837000 |
| 1 | 2.245877000 | -0.773845000 | 2.815180000 |
| 1 | 2.548440000 | 0.236373000 | 4.244103000 |
| 1 | 3.152817000 | -1.427485000 | 4.188570000 |
| 6 | -5.146475000 | -0.608586000 | -2.842452000 |
| 1 | -5.039292000 | 0.443074000 | -2.552000000 |
| 6 | 7.743273000 | 2.134269000 | -0.645493000 |
| 1 | 8.751263000 | 1.898328000 | -0.282920000 |

| | | | |
|---|---|---|---|
| 1 | 7.615371000 | 1.584151000 | -1.587313000 |
| 6 | -5.452469000 | 4.154527000 | 0.171451000 |
| 1 | -5.839605000 | 4.458618000 | -0.811977000 |
| 1 | -4.602557000 | 4.814537000 | 0.377510000 |
| 6 | -6.539916000 | 4.382132000 | 1.240458000 |
| 1 | -6.682106000 | 5.465633000 | 1.330112000 |
| 1 | -6.168614000 | 4.035169000 | 2.213787000 |
| 6 | -3.785606000 | -1.070185000 | -3.408753000 |
| 1 | -2.988610000 | -1.003734000 | -2.659807000 |
| 1 | -3.503386000 | -0.452577000 | -4.268910000 |
| 1 | -3.839253000 | -2.111611000 | -3.746157000 |
| 6 | 5.303817000 | 0.392270000 | 4.059383000 |
| 1 | 5.563051000 | -0.455567000 | 4.703238000 |
| 1 | 4.875318000 | 1.172461000 | 4.698635000 |
| 1 | 6.233952000 | 0.778865000 | 3.628118000 |
| 6 | 4.654759000 | -2.468874000 | -2.561718000 |
| 1 | 4.928268000 | -3.519638000 | -2.413141000 |
| 1 | 4.701009000 | -2.258954000 | -3.636489000 |
| 1 | 3.616612000 | -2.344624000 | -2.233770000 |
| 6 | 7.051481000 | -1.672804000 | -2.306669000 |
| 1 | 7.737613000 | -0.999803000 | -1.780402000 |
| 1 | 7.096995000 | -1.436777000 | -3.375795000 |
| 1 | 7.425411000 | -2.694661000 | -2.179352000 |
| 6 | -7.901615000 | 3.737966000 | 0.937905000 |
| 1 | -8.624664000 | 4.118128000 | 1.669569000 |
| 1 | -8.256388000 | 4.084273000 | -0.044198000 |
| 6 | -6.244152000 | -0.662645000 | -3.920977000 |
| 1 | -6.360511000 | -1.669453000 | -4.336070000 |
| 1 | -5.983131000 | 0.002243000 | -4.751375000 |
| 1 | -7.216195000 | -0.348161000 | -3.525111000 |
| 6 | -7.950302000 | 2.202221000 | 0.980996000 |
| 1 | -8.998774000 | 1.890107000 | 0.908198000 |
| 1 | -7.587683000 | 1.845214000 | 1.954225000 |
| 6 | -0.892033000 | -2.223467000 | -0.286901000 |
| 8 | 0.341607000 | -2.546676000 | -0.342400000 |
| 8 | -1.313304000 | -1.042381000 | -0.197659000 |
| 1 | -2.771898000 | -0.077235000 | -0.108034000 |
| 6 | -1.864713000 | -3.396828000 | -0.335405000 |
| 1 | -1.696445000 | -3.973074000 | -1.251118000 |
| 1 | -1.664928000 | -4.070528000 | 0.504511000 |
| 1 | -2.904143000 | -3.065323000 | -0.298099000 |

Zero-point correction= 0.925065 (Hartree/Particle)

Thermal correction to Energy= 0.979352

Thermal correction to Enthalpy= 0.980296

Thermal correction to Gibbs Free Energy= 0.826471
Sum of electronic and zero-point Energies= -2836.447012
Sum of electronic and thermal Energies= -2836.392725
Sum of electronic and thermal Enthalpies= -2836.391781
Sum of electronic and thermal Free Energies= -2836.545606

2

| | | | |
|---|---|---|---|
| 6 | 4.352020000 | -0.220298000 | 0.735921000 |
| 8 | 3.265600000 | 0.277429000 | 1.106470000 |
| 7 | 4.589035000 | -1.541766000 | 0.899815000 |
| 6 | 3.594807000 | -2.433557000 | 1.494594000 |
| 1 | 3.304969000 | -2.047902000 | 2.479576000 |
| 6 | 2.398616000 | -2.600809000 | 0.658743000 |
| 1 | 4.073872000 | -3.404672000 | 1.651930000 |
| 6 | 1.403700000 | -2.739400000 | -0.016547000 |
| 1 | 0.542781000 | -2.930483000 | -0.621760000 |
| 6 | 5.425189000 | 0.616913000 | 0.137131000 |
| 6 | 6.428176000 | 0.083247000 | -0.689453000 |
| 6 | 7.409934000 | 0.915388000 | -1.223301000 |
| 6 | 7.401371000 | 2.282221000 | -0.933589000 |
| 6 | 6.400990000 | 2.819880000 | -0.119255000 |
| 6 | 5.411735000 | 1.993987000 | 0.408345000 |
| 1 | 5.507576000 | -1.910037000 | 0.699232000 |
| 1 | 6.428699000 | -0.970188000 | -0.957802000 |
| 1 | 8.175738000 | 0.499342000 | -1.870826000 |
| 1 | 8.170101000 | 2.927970000 | -1.347838000 |
| 1 | 6.392823000 | 3.882475000 | 0.103963000 |
| 1 | 4.629023000 | 2.397796000 | 1.041556000 |
| 47 | 1.086241000 | 0.110041000 | 0.588900000 |
| 16 | -1.208689000 | 2.552423000 | 0.655700000 |
| 7 | -2.007181000 | 0.340176000 | -0.143009000 |
| 6 | -2.027554000 | -1.064978000 | -0.529616000 |
| 6 | -0.873098000 | 0.909590000 | 0.306056000 |
| 6 | -2.334937000 | -2.029177000 | 0.452077000 |
| 6 | -2.042128000 | -3.733365000 | -1.262998000 |
| 1 | -2.051824000 | -4.780695000 | -1.551535000 |
| 6 | -1.737878000 | -1.393753000 | -1.869496000 |
| 6 | -2.332234000 | -3.371910000 | 0.050961000 |
| 1 | -2.563185000 | -4.144360000 | 0.777963000 |
| 6 | -1.746748000 | -2.753886000 | -2.209923000 |
| 1 | -1.529431000 | -3.047649000 | -3.232484000 |
| 6 | -2.651012000 | -1.672123000 | 1.902331000 |
| 1 | -2.687534000 | -0.581417000 | 1.988548000 |
| 6 | -3.153143000 | 1.166060000 | -0.225693000 |
| 6 | -4.027210000 | -2.211166000 | 2.341265000 |

| | | | |
|---|---|---|---|
| 1 | -4.829267000 | -1.857018000 | 1.684653000 |
| 1 | -4.251307000 | -1.878953000 | 3.360848000 |
| 1 | -4.054140000 | -3.306368000 | 2.339420000 |
| 6 | -4.473567000 | 0.645997000 | -0.733750000 |
| 1 | -4.398914000 | -0.435077000 | -0.876492000 |
| 1 | -4.661315000 | 1.072092000 | -1.730353000 |
| 6 | -2.885812000 | 2.439676000 | 0.180045000 |
| 6 | -1.542252000 | -2.168170000 | 2.852534000 |
| 1 | -1.465436000 | -3.261408000 | 2.838872000 |
| 1 | -1.759291000 | -1.860530000 | 3.881489000 |
| 1 | -0.564427000 | -1.760565000 | 2.572460000 |
| 6 | -1.420825000 | -0.347094000 | -2.935055000 |
| 1 | -1.533157000 | 0.645289000 | -2.487099000 |
| 6 | -3.811558000 | 3.626951000 | 0.219050000 |
| 1 | -4.022695000 | 3.946407000 | -0.812255000 |
| 1 | -3.290357000 | 4.466270000 | 0.692693000 |
| 6 | -5.142308000 | 3.380016000 | 0.956009000 |
| 1 | -5.636374000 | 4.352255000 | 1.071134000 |
| 1 | -4.931898000 | 3.015291000 | 1.970126000 |
| 6 | 0.038115000 | -0.464157000 | -3.420030000 |
| 1 | 0.745752000 | -0.351797000 | -2.590098000 |
| 1 | 0.256245000 | 0.315583000 | -4.158075000 |
| 1 | 0.225864000 | -1.434219000 | -3.894340000 |
| 6 | -6.113072000 | 2.423919000 | 0.246435000 |
| 1 | -7.076369000 | 2.468366000 | 0.769004000 |
| 1 | -6.305013000 | 2.792704000 | -0.772194000 |
| 6 | -2.402019000 | -0.426091000 | -4.122035000 |
| 1 | -2.308072000 | -1.373881000 | -4.663427000 |
| 1 | -2.196617000 | 0.381255000 | -4.833351000 |
| 1 | -3.442437000 | -0.332356000 | -3.792206000 |
| 6 | -5.681395000 | 0.950601000 | 0.176893000 |
| 1 | -6.525909000 | 0.364099000 | -0.204813000 |
| 1 | -5.475998000 | 0.576296000 | 1.188705000 |

Zero-point correction= 0.597642 (Hartree/Particle)
Thermal correction to Energy= 0.634650
Thermal correction to Enthalpy= 0.635594
Thermal correction to Gibbs Free Energy= 0.523176
Sum of electronic and zero-point Energies= -1892.743887
Sum of electronic and thermal Energies= -1892.706879
Sum of electronic and thermal Enthalpies= -1892.705935
Sum of electronic and thermal Free Energies= -1892.818353

TS2（1 imaginary frequencies, -90.3 i $cm^{-1}$）

| | | | |
|---|---|---|---|
| 6 | 5.905379000 | -0.242896000 | 0.494306000 |
| 8 | 5.366943000 | 0.819016000 | 0.845786000 |

| | | | |
|---|---|---|---|
| 7 | 5.175661000 | -1.387988000 | 0.630462000 |
| 6 | 3.886739000 | -1.255125000 | 1.288117000 |
| 1 | 3.997831000 | -1.294783000 | 2.381360000 |
| 6 | 3.245410000 | 0.022464000 | 0.918120000 |
| 1 | 3.240964000 | -2.085154000 | 0.984389000 |
| 6 | 2.399454000 | 0.897087000 | 0.647903000 |
| 1 | 2.379589000 | 1.961093000 | 0.471829000 |
| 6 | 7.273011000 | -0.309422000 | -0.075206000 |
| 6 | 8.012803000 | -1.501215000 | -0.138573000 |
| 6 | 9.293162000 | -1.499243000 | -0.687669000 |
| 6 | 9.842375000 | -0.310344000 | -1.175337000 |
| 6 | 9.114406000 | 0.880516000 | -1.103997000 |
| 6 | 7.836390000 | 0.884157000 | -0.551813000 |
| 1 | 5.484597000 | -2.282144000 | 0.280714000 |
| 1 | 7.616703000 | -2.427432000 | 0.269887000 |
| 1 | 9.865226000 | -2.421244000 | -0.727046000 |
| 1 | 10.840591000 | -0.311620000 | -1.603404000 |
| 1 | 9.544570000 | 1.805206000 | -1.476677000 |
| 1 | 7.258617000 | 1.799535000 | -0.483056000 |
| 47 | 0.414775000 | -0.028193000 | 0.551921000 |
| 16 | -1.990738000 | -2.441782000 | 0.865025000 |
| 7 | -2.679841000 | -0.178039000 | 0.107358000 |
| 6 | -2.609046000 | 1.229205000 | -0.272627000 |
| 6 | -1.561053000 | -0.833136000 | 0.465105000 |
| 6 | -2.375209000 | 1.550054000 | -1.625466000 |
| 6 | -2.407981000 | 3.895667000 | -0.975237000 |
| 1 | -2.328220000 | 4.943264000 | -1.251112000 |
| 6 | -2.759784000 | 2.201750000 | 0.736847000 |
| 6 | -2.274977000 | 2.909344000 | -1.949372000 |
| 1 | -2.091727000 | 3.198422000 | -2.979731000 |
| 6 | -2.650084000 | 3.543632000 | 0.350167000 |
| 1 | -2.757375000 | 4.322838000 | 1.098696000 |
| 6 | -2.224731000 | 0.495328000 | -2.718968000 |
| 1 | -2.416994000 | -0.488385000 | -2.277989000 |
| 6 | -3.889498000 | -0.907862000 | 0.136418000 |
| 6 | -3.250039000 | 0.695355000 | -3.852570000 |
| 1 | -4.277111000 | 0.701198000 | -3.471549000 |
| 1 | -3.164184000 | -0.114644000 | -4.585360000 |
| 1 | -3.087313000 | 1.638938000 | -4.384717000 |
| 6 | -5.207000000 | -0.271976000 | -0.226115000 |
| 1 | -5.018928000 | 0.730301000 | -0.619692000 |
| 1 | -5.796712000 | -0.132877000 | 0.691976000 |
| 6 | -3.690477000 | -2.194710000 | 0.541498000 |
| 6 | -0.787982000 | 0.471033000 | -3.278607000 |

| | | | |
|---|---|---|---|
| 1 | -0.525740000 | 1.425516000 | -3.748760000 |
| 1 | -0.687274000 | -0.314232000 | -4.036140000 |
| 1 | -0.054386000 | 0.275073000 | -2.487204000 |
| 6 | -3.021905000 | 1.853268000 | 2.200106000 |
| 1 | -3.131699000 | 0.767094000 | 2.283712000 |
| 6 | -4.708250000 | -3.286983000 | 0.739809000 |
| 1 | -5.311366000 | -3.051946000 | 1.629304000 |
| 1 | -4.184341000 | -4.221235000 | 0.970549000 |
| 6 | -5.645762000 | -3.519240000 | -0.461346000 |
| 1 | -6.210299000 | -4.437562000 | -0.259871000 |
| 1 | -5.043098000 | -3.714687000 | -1.358238000 |
| 6 | -1.832612000 | 2.258824000 | 3.093960000 |
| 1 | -0.904638000 | 1.772369000 | 2.769507000 |
| 1 | -2.021368000 | 1.968078000 | 4.133316000 |
| 1 | -1.667382000 | 3.341903000 | 3.074256000 |
| 6 | -6.645730000 | -2.386382000 | -0.738440000 |
| 1 | -7.362603000 | -2.743080000 | -1.488049000 |
| 1 | -7.232706000 | -2.190925000 | 0.171265000 |
| 6 | -4.332912000 | 2.483262000 | 2.711743000 |
| 1 | -4.282708000 | 3.577603000 | 2.713742000 |
| 1 | -4.529421000 | 2.160212000 | 3.739966000 |
| 1 | -5.188642000 | 2.190311000 | 2.093628000 |
| 6 | -6.047916000 | -1.065443000 | -1.247266000 |
| 1 | -6.873058000 | -0.413147000 | -1.557843000 |
| 1 | -5.445374000 | -1.252040000 | -2.146311000 |

Zero-point correction= 0.596757 (Hartree/Particle)
Thermal correction to Energy= 0.632999
Thermal correction to Enthalpy= 0.633943
Thermal correction to Gibbs Free Energy= 0.522043
Sum of electronic and zero-point Energies= -1892.725326
Sum of electronic and thermal Energies= -1892.689084
Sum of electronic and thermal Enthalpies= -1892.688140
Sum of electronic and thermal Free Energies= -1892.800040

TS22（1 imaginary frequencies, -233.6 i $cm^{-1}$）

| | | | |
|---|---|---|---|
| 6 | -4.919293000 | -0.487735000 | -0.463503000 |
| 8 | -3.694934000 | -0.499567000 | -0.734247000 |
| 7 | -5.560843000 | -1.672793000 | -0.578833000 |
| 6 | -4.735932000 | -2.764702000 | -1.071524000 |
| 1 | -4.820046000 | -2.849118000 | -2.164115000 |
| 6 | -3.324302000 | -2.536146000 | -0.688707000 |
| 1 | -5.064265000 | -3.711565000 | -0.628288000 |
| 6 | -2.128056000 | -2.792915000 | -0.399954000 |
| 1 | -1.657864000 | -3.749666000 | -0.205243000 |
| 6 | -5.641272000 | 0.722854000 | -0.024223000 |

| | | | |
|---|---|---|---|
| 6 | -7.036494000 | 0.837730000 | -0.139417000 |
| 6 | -7.677093000 | 1.996129000 | 0.293338000 |
| 6 | -6.931749000 | 3.042533000 | 0.843625000 |
| 6 | -5.542300000 | 2.935619000 | 0.951391000 |
| 6 | -4.895538000 | 1.783182000 | 0.513765000 |
| 1 | -6.511945000 | -1.818218000 | -0.274113000 |
| 1 | -7.623738000 | 0.047441000 | -0.599446000 |
| 1 | -8.754383000 | 2.086399000 | 0.194295000 |
| 1 | -7.434145000 | 3.943964000 | 1.182006000 |
| 1 | -4.964945000 | 3.751425000 | 1.375612000 |
| 1 | -3.817733000 | 1.685860000 | 0.588560000 |
| 47 | -0.732879000 | -1.128578000 | -0.304067000 |
| 16 | 0.355215000 | 2.086303000 | -0.586245000 |
| 7 | 2.011374000 | 0.366780000 | 0.098036000 |
| 6 | 2.592456000 | -0.920795000 | 0.462285000 |
| 6 | 0.707374000 | 0.448520000 | -0.224121000 |
| 6 | 3.134163000 | -1.726962000 | -0.559151000 |
| 6 | 3.626331000 | -3.393131000 | 1.145690000 |
| 1 | 4.031048000 | -4.364979000 | 1.413614000 |
| 6 | 2.565933000 | -1.308091000 | 1.817233000 |
| 6 | 3.647363000 | -2.974651000 | -0.182363000 |
| 1 | 4.069547000 | -3.626925000 | -0.940763000 |
| 6 | 3.094300000 | -2.566663000 | 2.132003000 |
| 1 | 3.087588000 | -2.902880000 | 3.164506000 |
| 6 | 3.170322000 | -1.302304000 | -2.025397000 |
| 1 | 2.809530000 | -0.270833000 | -2.094263000 |
| 6 | 2.759588000 | 1.565912000 | 0.072377000 |
| 6 | 4.602989000 | -1.321347000 | -2.594511000 |
| 1 | 5.281236000 | -0.696227000 | -2.003341000 |
| 1 | 4.603555000 | -0.944445000 | -3.623304000 |
| 1 | 5.018487000 | -2.334771000 | -2.615909000 |
| 6 | 4.223465000 | 1.594389000 | 0.429024000 |
| 1 | 4.576460000 | 0.567308000 | 0.553993000 |
| 1 | 4.340948000 | 2.078414000 | 1.409864000 |
| 6 | 1.988807000 | 2.631337000 | -0.285735000 |
| 6 | 2.229763000 | -2.173055000 | -2.882718000 |
| 1 | 2.538609000 | -3.224559000 | -2.870035000 |
| 1 | 2.240279000 | -1.833124000 | -3.924412000 |
| 1 | 1.196776000 | -2.121545000 | -2.519067000 |
| 6 | 1.985127000 | -0.430982000 | 2.923893000 |
| 1 | 1.685758000 | 0.526357000 | 2.485117000 |
| 6 | 2.384009000 | 4.079927000 | -0.402457000 |
| 1 | 2.558450000 | 4.483015000 | 0.606210000 |
| 1 | 1.538246000 | 4.645386000 | -0.809495000 |

6 3.627959000 4.340455000 -1.274025000
1 3.702613000 5.424925000 -1.419887000
1 3.470334000 3.902681000 -2.268689000
6 0.719272000 -1.066226000 3.533470000
1 -0.049062000 -1.234615000 2.769456000
1 0.296895000 -0.409687000 4.302527000
1 0.942573000 -2.031078000 4.002656000
6 4.955095000 3.838552000 -0.684807000
1 5.771122000 4.232850000 -1.302741000
1 5.095800000 4.275826000 0.315083000
6 3.028141000 -0.124914000 4.016983000
1 3.345086000 -1.033811000 4.540125000
1 2.603394000 0.554642000 4.764198000
1 3.923580000 0.347251000 3.598054000
6 5.123573000 2.313439000 -0.596979000
1 6.162326000 2.099948000 -0.317003000
1 4.971508000 1.865556000 -1.588063000
Zero-point correction= 0.596870 (Hartree/Particle)
Thermal correction to Energy= 0.632816
Thermal correction to Enthalpy= 0.633761
Thermal correction to Gibbs Free Energy= 0.523077
Sum of electronic and zero-point Energies= -1892.720603
Sum of electronic and thermal Energies= -1892.684656
Sum of electronic and thermal Enthalpies= -1892.683712
Sum of electronic and thermal Free Energies= -1892.794396
3
6 -5.838311000 -0.629070000 -0.050120000
8 -4.930943000 0.278457000 0.151610000
7 -5.316356000 -1.812303000 -0.343991000
6 -3.846053000 -1.773607000 -0.358655000
1 -3.466422000 -2.043551000 -1.350452000
6 -3.576023000 -0.322959000 0.000495000
1 -3.438598000 -2.475340000 0.377287000
6 -2.486035000 0.395009000 0.175131000
1 -2.664556000 1.440820000 0.429578000
6 -7.250975000 -0.301704000 0.059162000
6 -8.246622000 -1.234921000 -0.286465000
6 -9.587900000 -0.892405000 -0.163375000
6 -9.944971000 0.377352000 0.303634000
6 -8.959898000 1.308594000 0.644679000
6 -7.614818000 0.976296000 0.522978000
1 -5.863580000 -2.647702000 -0.493887000
1 -7.987535000 -2.219976000 -0.664925000
1 -10.355341000 -1.610633000 -0.433382000

| | | | |
|---|---|---|---|
| 1 | -10.994181000 | 0.640544000 | 0.398840000 |
| 1 | -9.240965000 | 2.292881000 | 1.005215000 |
| 1 | -6.842658000 | 1.690566000 | 0.786132000 |
| 47 | -0.482218000 | -0.248209000 | 0.025936000 |
| 16 | 2.008342000 | -2.556071000 | -0.174498000 |
| 7 | 2.678053000 | -0.166824000 | -0.136744000 |
| 6 | 2.597497000 | 1.289012000 | -0.102734000 |
| 6 | 1.551766000 | -0.903830000 | -0.103437000 |
| 6 | 2.591811000 | 1.934907000 | 1.149935000 |
| 6 | 2.386333000 | 4.046454000 | -0.043070000 |
| 1 | 2.302609000 | 5.129322000 | -0.019430000 |
| 6 | 2.510736000 | 1.982695000 | -1.326409000 |
| 6 | 2.481355000 | 3.331338000 | 1.147850000 |
| 1 | 2.469645000 | 3.864861000 | 2.093591000 |
| 6 | 2.402554000 | 3.377735000 | -1.264252000 |
| 1 | 2.329081000 | 3.946753000 | -2.186134000 |
| 6 | 2.689480000 | 1.184065000 | 2.475509000 |
| 1 | 2.848755000 | 0.122614000 | 2.259605000 |
| 6 | 3.907184000 | -0.861735000 | -0.218443000 |
| 6 | 3.887932000 | 1.662431000 | 3.319081000 |
| 1 | 4.830230000 | 1.579810000 | 2.766267000 |
| 1 | 3.973227000 | 1.057231000 | 4.228553000 |
| 1 | 3.774645000 | 2.707202000 | 3.628795000 |
| 6 | 5.227219000 | -0.136751000 | -0.276794000 |
| 1 | 5.052210000 | 0.930425000 | -0.117124000 |
| 1 | 5.639095000 | -0.228428000 | -1.292766000 |
| 6 | 3.720620000 | -2.210711000 | -0.253638000 |
| 6 | 1.376902000 | 1.294279000 | 3.277363000 |
| 1 | 1.159263000 | 2.335201000 | 3.542762000 |
| 1 | 1.450273000 | 0.719681000 | 4.207816000 |
| 1 | 0.525924000 | 0.908638000 | 2.704218000 |
| 6 | 2.516233000 | 1.282132000 | -2.683037000 |
| 1 | 2.677813000 | 0.211899000 | -2.518651000 |
| 6 | 4.755361000 | -3.298705000 | -0.368070000 |
| 1 | 5.182319000 | -3.279986000 | -1.381863000 |
| 1 | 4.259011000 | -4.270639000 | -0.269401000 |
| 6 | 5.896790000 | -3.210744000 | 0.663466000 |
| 1 | 6.473342000 | -4.141286000 | 0.593884000 |
| 1 | 5.468805000 | -3.184005000 | 1.674418000 |
| 6 | 1.154958000 | 1.432447000 | -3.391191000 |
| 1 | 0.340814000 | 1.034648000 | -2.774534000 |
| 1 | 1.159704000 | 0.890351000 | -4.343759000 |
| 1 | 0.931376000 | 2.483808000 | -3.605201000 |
| 6 | 6.860491000 | -2.029048000 | 0.472361000 |

| | | | |
|---|---|---|---|
| 1 | 7.712803000 | -2.173738000 | 1.147607000 |
| 1 | 7.273970000 | -2.060607000 | -0.546829000 |
| 6 | 3.664061000 | 1.782314000 | -3.582333000 |
| 1 | 3.544363000 | 2.839921000 | -3.842255000 |
| 1 | 3.684762000 | 1.213362000 | -4.518582000 |
| 1 | 4.637973000 | 1.668010000 | -3.093591000 |
| 6 | 6.279114000 | -0.631125000 | 0.737463000 |
| 1 | 7.104912000 | 0.090699000 | 0.722150000 |
| 1 | 5.852625000 | -0.592650000 | 1.748790000 |

Zero-point correction= 0.600191 (Hartree/Particle)
Thermal correction to Energy= 0.635816
Thermal correction to Enthalpy= 0.636761
Thermal correction to Gibbs Free Energy= 0.526727
Sum of electronic and zero-point Energies= -1892.737945
Sum of electronic and thermal Energies= -1892.702320
Sum of electronic and thermal Enthalpies= -1892.701376
Sum of electronic and thermal Free Energies= -1892.811410

4

| | | | |
|---|---|---|---|
| 6 | 1.633653000 | -4.153765000 | 0.264751000 |
| 8 | 0.668514000 | -3.908377000 | -0.606621000 |
| 7 | 1.715965000 | -3.274865000 | 1.226110000 |
| 6 | 0.720270000 | -2.217679000 | 1.032683000 |
| 1 | 1.236112000 | -1.265373000 | 0.860996000 |
| 6 | -0.059422000 | -2.714385000 | -0.169965000 |
| 1 | 0.095046000 | -2.113245000 | 1.925957000 |
| 6 | -1.156482000 | -2.316806000 | -0.793253000 |
| 1 | -1.436976000 | -2.940098000 | -1.644996000 |
| 6 | 2.471473000 | -5.337289000 | 0.074659000 |
| 6 | 3.512917000 | -5.634993000 | 0.972666000 |
| 6 | 4.286196000 | -6.774437000 | 0.766888000 |
| 6 | 4.031983000 | -7.615539000 | -0.320422000 |
| 6 | 2.997931000 | -7.318678000 | -1.212576000 |
| 6 | 2.216872000 | -6.183340000 | -1.018783000 |
| 1 | 2.507349000 | -3.196107000 | 1.980456000 |
| 1 | 3.716494000 | -4.983675000 | 1.818038000 |
| 1 | 5.088636000 | -7.008456000 | 1.460065000 |
| 1 | 4.638617000 | -8.504131000 | -0.471256000 |
| 1 | 2.799095000 | -7.973094000 | -2.055947000 |
| 1 | 1.409184000 | -5.945579000 | -1.702085000 |
| 47 | -2.456035000 | -0.756546000 | -0.266836000 |
| 16 | -3.443302000 | 2.196893000 | 1.155304000 |
| 7 | -5.174483000 | 0.840336000 | 0.009432000 |
| 6 | -5.814275000 | -0.237683000 | -0.735776000 |
| 6 | -3.853738000 | 0.785263000 | 0.267318000 |

| | | | |
|---|---|---|---|
| 6 | -6.321674000 | -1.341295000 | -0.020966000 |
| 6 | -6.976809000 | -2.307517000 | -2.156486000 |
| 1 | -7.431068000 | -3.121486000 | -2.714417000 |
| 6 | -5.881012000 | -0.135054000 | -2.139455000 |
| 6 | -6.901778000 | -2.374503000 | -0.767883000 |
| 1 | -7.297645000 | -3.244598000 | -0.252786000 |
| 6 | -6.473850000 | -1.198484000 | -2.831553000 |
| 1 | -6.539903000 | -1.157155000 | -3.914678000 |
| 6 | -6.250756000 | -1.454093000 | 1.499895000 |
| 1 | -5.838656000 | -0.520323000 | 1.895853000 |
| 6 | -5.887709000 | 1.966112000 | 0.485327000 |
| 6 | -7.647034000 | -1.639340000 | 2.126750000 |
| 1 | -8.328620000 | -0.828763000 | 1.845310000 |
| 1 | -7.569988000 | -1.654610000 | 3.219752000 |
| 1 | -8.107577000 | -2.583662000 | 1.816407000 |
| 6 | -7.363420000 | 2.133864000 | 0.228881000 |
| 1 | -7.749265000 | 1.217546000 | -0.225325000 |
| 1 | -7.506320000 | 2.927559000 | -0.519542000 |
| 6 | -5.073627000 | 2.832686000 | 1.149101000 |
| 6 | -5.301223000 | -2.590166000 | 1.930672000 |
| 1 | -5.664619000 | -3.564628000 | 1.584804000 |
| 1 | -5.227403000 | -2.628854000 | 3.023640000 |
| 1 | -4.294801000 | -2.443505000 | 1.523324000 |
| 6 | -5.333097000 | 1.061337000 | -2.913953000 |
| 1 | -4.961548000 | 1.797554000 | -2.193896000 |
| 6 | -5.423110000 | 4.154525000 | 1.780790000 |
| 1 | -5.622126000 | 4.889451000 | 0.986251000 |
| 1 | -4.547358000 | 4.529239000 | 2.322783000 |
| 6 | -6.626281000 | 4.112802000 | 2.742487000 |
| 1 | -6.665128000 | 5.079350000 | 3.260037000 |
| 1 | -6.445937000 | 3.353045000 | 3.514642000 |
| 6 | -4.139640000 | 0.652623000 | -3.800449000 |
| 1 | -3.337643000 | 0.205269000 | -3.202505000 |
| 1 | -3.734255000 | 1.528979000 | -4.319436000 |
| 1 | -4.438760000 | -0.077740000 | -4.560799000 |
| 6 | -7.988276000 | 3.865984000 | 2.075792000 |
| 1 | -8.769640000 | 4.037113000 | 2.826659000 |
| 1 | -8.150634000 | 4.622389000 | 1.293248000 |
| 6 | -6.430158000 | 1.752070000 | -3.747776000 |
| 1 | -6.823527000 | 1.089427000 | -4.526658000 |
| 1 | -6.024867000 | 2.641041000 | -4.244273000 |
| 1 | -7.273188000 | 2.066820000 | -3.122576000 |
| 6 | -8.203956000 | 2.466857000 | 1.478125000 |
| 1 | -9.258524000 | 2.376604000 | 1.189995000 |

| | | | |
|---|---|---|---|
| 1 | -8.026205000 | 1.703979000 | 2.247790000 |
| 16 | 7.379127000 | 1.133439000 | 0.928366000 |
| 7 | 5.152127000 | 1.602537000 | -0.125334000 |
| 6 | 3.720738000 | 1.510876000 | -0.410893000 |
| 6 | 5.720994000 | 0.804213000 | 0.774028000 |
| 6 | 2.840566000 | 2.277585000 | 0.375723000 |
| 6 | 1.025163000 | 1.354727000 | -0.955518000 |
| 1 | -0.037409000 | 1.287773000 | -1.170220000 |
| 6 | 3.301737000 | 0.646677000 | -1.439353000 |
| 6 | 1.477706000 | 2.182632000 | 0.069133000 |
| 1 | 0.761015000 | 2.753910000 | 0.650818000 |
| 6 | 1.925824000 | 0.590336000 | -1.693236000 |
| 1 | 1.551348000 | -0.074530000 | -2.464796000 |
| 6 | 3.306413000 | 3.114197000 | 1.563205000 |
| 1 | 4.398018000 | 3.201315000 | 1.519204000 |
| 6 | 6.020703000 | 2.522608000 | -0.742371000 |
| 6 | 2.744795000 | 4.547562000 | 1.544204000 |
| 1 | 2.995386000 | 5.069055000 | 0.613321000 |
| 1 | 3.160269000 | 5.122797000 | 2.378883000 |
| 1 | 1.654714000 | 4.559962000 | 1.651416000 |
| 6 | 5.533420000 | 3.481499000 | -1.794967000 |
| 1 | 4.445565000 | 3.398705000 | -1.864014000 |
| 1 | 5.927675000 | 3.163361000 | -2.771014000 |
| 6 | 7.298377000 | 2.398587000 | -0.275045000 |
| 6 | 2.955543000 | 2.390567000 | 2.882016000 |
| 1 | 1.868928000 | 2.343720000 | 3.020016000 |
| 1 | 3.379355000 | 2.929127000 | 3.737363000 |
| 1 | 3.333401000 | 1.362473000 | 2.887954000 |
| 6 | 4.261024000 | -0.257429000 | -2.206815000 |
| 1 | 5.287533000 | 0.037927000 | -1.961138000 |
| 6 | 8.522787000 | 3.173255000 | -0.685487000 |
| 1 | 8.809640000 | 2.867370000 | -1.702222000 |
| 1 | 9.358581000 | 2.886703000 | -0.037697000 |
| 6 | 8.352560000 | 4.705307000 | -0.642462000 |
| 1 | 9.347212000 | 5.144629000 | -0.783627000 |
| 1 | 8.019736000 | 5.003972000 | 0.360238000 |
| 6 | 4.083757000 | -1.721183000 | -1.747497000 |
| 1 | 4.176892000 | -1.810132000 | -0.659746000 |
| 1 | 4.835716000 | -2.362387000 | -2.221553000 |
| 1 | 3.094433000 | -2.098764000 | -2.032666000 |
| 6 | 7.409319000 | 5.290747000 | -1.705220000 |
| 1 | 7.512963000 | 6.382358000 | -1.683730000 |
| 1 | 7.748384000 | 4.975309000 | -2.703194000 |
| 6 | 4.109540000 | -0.123657000 | -3.733366000 |

| | | | |
|---|---|---|---|
| 1 | 3.122592000 | -0.453345000 | -4.075317000 |
| 1 | 4.855659000 | -0.746329000 | -4.239107000 |
| 1 | 4.249603000 | 0.911603000 | -4.064746000 |
| 6 | 5.917538000 | 4.954825000 | -1.549103000 |
| 1 | 5.353340000 | 5.556942000 | -2.271231000 |
| 1 | 5.569060000 | 5.259037000 | -0.552936000 |
| 6 | 4.286921000 | -2.023921000 | 2.833872000 |
| 8 | 3.599649000 | -3.076377000 | 3.001610000 |
| 8 | 4.059700000 | -1.153427000 | 1.942167000 |
| 1 | 5.153356000 | 0.023331000 | 1.337799000 |
| 6 | 5.471717000 | -1.815135000 | 3.776798000 |
| 1 | 5.436117000 | -0.810988000 | 4.213474000 |
| 1 | 5.478296000 | -2.560344000 | 4.574174000 |
| 1 | 6.409892000 | -1.896816000 | 3.212509000 |

Zero-point correction= 1.093009 (Hartree/Particle)
Thermal correction to Energy= 1.158341
Thermal correction to Enthalpy= 1.159285
Thermal correction to Gibbs Free Energy= 0.978546
Sum of electronic and zero-point Energies= -3352.679184
Sum of electronic and thermal Energies= -3352.613853
Sum of electronic and thermal Enthalpies= -3352.612909
Sum of electronic and thermal Free Energies= -3352.793647
TS3（1 imaginary frequencies, -1273.6 i $cm^{-1}$）

| | | | |
|---|---|---|---|
| 6 | 1.804045000 | -3.725375000 | 0.165960000 |
| 8 | 1.318153000 | -2.818660000 | 1.094578000 |
| 7 | 1.742885000 | -3.346582000 | -1.056412000 |
| 6 | 1.152919000 | -2.008291000 | -1.060508000 |
| 1 | 0.234789000 | -1.999850000 | -1.659590000 |
| 6 | 0.909141000 | -1.692214000 | 0.399571000 |
| 1 | 1.858391000 | -1.300519000 | -1.517679000 |
| 6 | 0.517895000 | -0.544985000 | 1.006969000 |
| 1 | 0.568780000 | -0.592745000 | 2.098954000 |
| 6 | 2.324676000 | -4.985115000 | 0.698727000 |
| 6 | 2.900449000 | -5.924335000 | -0.174257000 |
| 6 | 3.392199000 | -7.125405000 | 0.326798000 |
| 6 | 3.312843000 | -7.401221000 | 1.695730000 |
| 6 | 2.742868000 | -6.469260000 | 2.565727000 |
| 6 | 2.250348000 | -5.262362000 | 2.073144000 |
| 1 | 2.871745000 | -3.588587000 | -2.434256000 |
| 1 | 2.967458000 | -5.703333000 | -1.234183000 |
| 1 | 3.838759000 | -7.847982000 | -0.349762000 |
| 1 | 3.696816000 | -8.340904000 | 2.082578000 |
| 1 | 2.682458000 | -6.681668000 | 3.629036000 |
| 1 | 1.808671000 | -4.535103000 | 2.745288000 |

| | | | |
|---|---|---|---|
| 47 | -1.412847000 | 0.242356000 | 0.387843000 |
| 16 | -3.699473000 | 2.693161000 | -0.227598000 |
| 7 | -4.501006000 | 0.343904000 | -0.271878000 |
| 6 | -4.506347000 | -1.113359000 | -0.216634000 |
| 6 | -3.352669000 | 1.020098000 | -0.081144000 |
| 6 | -4.698315000 | -1.740329000 | 1.030694000 |
| 6 | -4.447687000 | -3.876431000 | -0.109275000 |
| 1 | -4.422449000 | -4.961604000 | -0.066494000 |
| 6 | -4.303112000 | -1.827878000 | -1.414384000 |
| 6 | -4.660687000 | -3.140560000 | 1.053322000 |
| 1 | -4.799406000 | -3.660596000 | 1.996344000 |
| 6 | -4.274509000 | -3.225416000 | -1.328110000 |
| 1 | -4.115010000 | -3.810954000 | -2.228470000 |
| 6 | -4.930313000 | -0.967700000 | 2.326655000 |
| 1 | -4.985925000 | 0.099255000 | 2.087404000 |
| 6 | -5.664606000 | 1.103575000 | -0.533509000 |
| 6 | -6.265371000 | -1.356122000 | 2.992991000 |
| 1 | -7.113786000 | -1.207889000 | 2.315791000 |
| 1 | -6.432772000 | -0.743811000 | 3.886128000 |
| 1 | -6.270448000 | -2.405645000 | 3.307365000 |
| 6 | -7.000385000 | 0.449208000 | -0.776513000 |
| 1 | -6.908889000 | -0.622932000 | -0.584087000 |
| 1 | -7.254888000 | 0.546404000 | -1.842328000 |
| 6 | -5.399328000 | 2.439961000 | -0.549807000 |
| 6 | -3.754981000 | -1.154672000 | 3.307104000 |
| 1 | -3.652740000 | -2.202271000 | 3.612402000 |
| 1 | -3.915408000 | -0.556202000 | 4.211067000 |
| 1 | -2.805833000 | -0.843608000 | 2.855546000 |
| 6 | -4.104839000 | -1.145768000 | -2.765913000 |
| 1 | -4.248878000 | -0.068499000 | -2.633905000 |
| 6 | -6.345013000 | 3.580620000 | -0.818966000 |
| 1 | -6.621540000 | 3.570941000 | -1.883793000 |
| 1 | -5.815023000 | 4.525546000 | -0.654925000 |
| 6 | -7.626531000 | 3.568330000 | 0.036247000 |
| 1 | -8.134050000 | 4.527826000 | -0.121508000 |
| 1 | -7.351864000 | 3.532092000 | 1.098819000 |
| 6 | -2.668206000 | -1.349235000 | -3.288056000 |
| 1 | -1.931112000 | -0.957120000 | -2.577281000 |
| 1 | -2.530987000 | -0.827597000 | -4.242100000 |
| 1 | -2.449507000 | -2.410662000 | -3.452121000 |
| 6 | -8.616439000 | 2.438441000 | -0.287231000 |
| 1 | -9.549333000 | 2.639416000 | 0.253776000 |
| 1 | -8.873295000 | 2.477887000 | -1.356419000 |
| 6 | -5.139445000 | -1.619009000 | -3.806115000 |

| | | | |
|---|---|---|---|
| 1 | -5.026249000 | -2.684229000 | -4.035652000 |
| 1 | -5.012629000 | -1.064865000 | -4.742804000 |
| 1 | -6.164948000 | -1.461771000 | -3.454176000 |
| 6 | -8.159474000 | 1.015094000 | 0.069772000 |
| 1 | -9.012882000 | 0.338670000 | -0.061317000 |
| 1 | -7.887338000 | 0.968917000 | 1.132715000 |
| 16 | 1.600220000 | 2.162727000 | -1.721072000 |
| 7 | 3.085955000 | 2.210428000 | 0.268705000 |
| 6 | 3.621708000 | 1.933874000 | 1.598422000 |
| 6 | 1.990989000 | 1.570520000 | -0.162888000 |
| 6 | 4.576918000 | 0.909482000 | 1.748403000 |
| 6 | 4.632779000 | 1.439688000 | 4.124748000 |
| 1 | 5.031699000 | 1.246215000 | 5.116560000 |
| 6 | 3.152576000 | 2.713758000 | 2.674945000 |
| 6 | 5.073167000 | 0.687162000 | 3.040167000 |
| 1 | 5.812218000 | -0.092762000 | 3.196000000 |
| 6 | 3.679089000 | 2.438002000 | 3.942620000 |
| 1 | 3.337751000 | 3.013268000 | 4.797970000 |
| 6 | 5.073194000 | 0.047705000 | 0.591079000 |
| 1 | 4.510839000 | 0.306188000 | -0.310822000 |
| 6 | 3.648846000 | 3.181484000 | -0.595697000 |
| 6 | 6.564736000 | 0.298784000 | 0.292782000 |
| 1 | 6.764409000 | 1.351821000 | 0.066348000 |
| 1 | 6.878001000 | -0.298517000 | -0.570561000 |
| 1 | 7.195941000 | 0.017394000 | 1.143749000 |
| 6 | 4.860765000 | 3.984879000 | -0.200427000 |
| 1 | 5.277558000 | 3.562422000 | 0.717696000 |
| 1 | 4.542906000 | 5.007180000 | 0.053737000 |
| 6 | 2.938286000 | 3.289838000 | -1.753630000 |
| 6 | 4.823911000 | -1.449438000 | 0.859561000 |
| 1 | 5.469505000 | -1.829351000 | 1.659973000 |
| 1 | 5.029186000 | -2.024889000 | -0.047821000 |
| 1 | 3.786299000 | -1.633434000 | 1.154777000 |
| 6 | 2.096020000 | 3.804066000 | 2.512817000 |
| 1 | 1.911078000 | 3.950975000 | 1.443690000 |
| 6 | 3.158280000 | 4.226018000 | -2.913098000 |
| 1 | 2.875638000 | 5.243922000 | -2.605230000 |
| 1 | 2.469090000 | 3.954224000 | -3.720523000 |
| 6 | 4.594882000 | 4.248471000 | -3.467698000 |
| 1 | 4.575897000 | 4.829448000 | -4.397989000 |
| 1 | 4.892805000 | 3.228000000 | -3.742091000 |
| 6 | 0.758783000 | 3.372201000 | 3.149445000 |
| 1 | 0.391584000 | 2.438211000 | 2.709195000 |
| 1 | -0.002746000 | 4.146298000 | 2.998324000 |

| | | | |
|---|---|---|---|
| 1 | 0.866294000 | 3.214096000 | 4.228743000 |
| 6 | 5.643853000 | 4.865401000 | -2.530585000 |
| 1 | 6.573029000 | 4.995643000 | -3.098988000 |
| 1 | 5.322208000 | 5.877140000 | -2.241025000 |
| 6 | 2.564295000 | 5.157911000 | 3.080423000 |
| 1 | 2.710218000 | 5.116572000 | 4.165347000 |
| 1 | 1.813276000 | 5.930243000 | 2.879704000 |
| 1 | 3.510146000 | 5.477519000 | 2.629355000 |
| 6 | 5.972365000 | 4.055289000 | -1.267290000 |
| 1 | 6.847918000 | 4.508633000 | -0.786608000 |
| 1 | 6.266204000 | 3.036190000 | -1.550549000 |
| 6 | 4.255532000 | -2.535261000 | -3.242004000 |
| 8 | 3.563073000 | -3.676221000 | -3.151381000 |
| 8 | 4.023257000 | -1.543482000 | -2.566665000 |
| 1 | 1.181107000 | 0.532519000 | 0.447180000 |
| 6 | 5.350972000 | -2.620328000 | -4.279079000 |
| 1 | 6.046322000 | -3.426728000 | -4.023191000 |
| 1 | 4.921587000 | -2.863715000 | -5.256614000 |
| 1 | 5.886071000 | -1.671749000 | -4.333798000 |

Zero-point correction= 1.088850 (Hartree/Particle)
Thermal correction to Energy= 1.154026
Thermal correction to Enthalpy= 1.154970
Thermal correction to Gibbs Free Energy= 0.976400
Sum of electronic and zero-point Energies= -3352.655838
Sum of electronic and thermal Energies= -3352.590662
Sum of electronic and thermal Enthalpies= -3352.589718
Sum of electronic and thermal Free Energies= -3352.768288

P1

| | | | |
|---|---|---|---|
| 6 | 0.620163000 | -0.343267000 | -0.000039000 |
| 8 | 1.421681000 | 0.785188000 | 0.000243000 |
| 7 | 1.210429000 | -1.475362000 | -0.000247000 |
| 6 | 2.647367000 | -1.190488000 | -0.000093000 |
| 1 | 3.123320000 | -1.638544000 | -0.882171000 |
| 6 | 2.735681000 | 0.326946000 | 0.000067000 |
| 1 | 3.123142000 | -1.638745000 | 0.881985000 |
| 6 | 3.770548000 | 1.161751000 | 0.000040000 |
| 1 | 3.630638000 | 2.236786000 | 0.000170000 |
| 6 | -0.828226000 | -0.102780000 | -0.000004000 |
| 6 | -1.703779000 | -1.200284000 | 0.000130000 |
| 6 | -3.079695000 | -0.992053000 | 0.000133000 |
| 6 | -3.593708000 | 0.308927000 | -0.000001000 |
| 6 | -2.725118000 | 1.402006000 | -0.000133000 |
| 6 | -1.345037000 | 1.200688000 | -0.000128000 |

| | | | |
|---|---|---|---|
| 1 | -1.286795000 | -2.201955000 | 0.000223000 |
| 1 | -3.754129000 | -1.843901000 | 0.000240000 |
| 1 | -4.668664000 | 0.468492000 | 0.000002000 |
| 1 | -3.121508000 | 2.413576000 | -0.000237000 |
| 1 | -0.665252000 | 2.045604000 | -0.000226000 |
| 1 | 4.783619000 | 0.776038000 | -0.000017000 |

Zero-point correction= 0.167594 (Hartree/Particle)
Thermal correction to Energy= 0.177084
Thermal correction to Enthalpy= 0.178028
Thermal correction to Gibbs Free Energy= 0.131978
Sum of electronic and zero-point Energies= -516.255994
Sum of electronic and thermal Energies= -516.246504
Sum of electronic and thermal Enthalpies= -516.245560
Sum of electronic and thermal Free Energies= -516.291609

TS31（1 imaginary frequencies, -739.9 i $cm^{-1}$）

| | | | |
|---|---|---|---|
| 6 | 4.794965000 | -1.175023000 | -0.717954000 |
| 8 | 3.682724000 | -1.779634000 | -0.280970000 |
| 7 | 4.545905000 | -0.012757000 | -1.269867000 |
| 6 | 3.102628000 | 0.249327000 | -1.262838000 |
| 1 | 2.697942000 | 0.140465000 | -2.277195000 |
| 6 | 2.632796000 | -0.814018000 | -0.314099000 |
| 1 | 2.905397000 | 1.244297000 | -0.858856000 |
| 6 | 1.609780000 | -0.830311000 | 0.549740000 |
| 1 | 1.576374000 | -1.678950000 | 1.233199000 |
| 6 | 6.077916000 | -1.819792000 | -0.536683000 |
| 6 | 7.255751000 | -1.204998000 | -1.008733000 |
| 6 | 8.477556000 | -1.840917000 | -0.824157000 |
| 6 | 8.534354000 | -3.082609000 | -0.181051000 |
| 6 | 7.367055000 | -3.693217000 | 0.287933000 |
| 6 | 6.138169000 | -3.066396000 | 0.116042000 |
| 1 | 5.234447000 | 0.772904000 | -1.339209000 |
| 1 | 7.218080000 | -0.238000000 | -1.500440000 |
| 1 | 9.387780000 | -1.371357000 | -1.183203000 |
| 1 | 9.492701000 | -3.575050000 | -0.044472000 |
| 1 | 7.417291000 | -4.654998000 | 0.788289000 |
| 1 | 5.227529000 | -3.528988000 | 0.479787000 |
| 47 | -0.417433000 | -0.314330000 | -0.209809000 |
| 16 | -2.703018000 | 0.779671000 | -2.496101000 |
| 7 | -3.542241000 | -0.151468000 | -0.350996000 |
| 6 | -3.556263000 | -0.769638000 | 0.970980000 |
| 6 | -2.369887000 | 0.083485000 | -0.967112000 |
| 6 | -3.488998000 | 0.061419000 | 2.107923000 |
| 6 | -3.512843000 | -1.958543000 | 3.467344000 |
| 1 | -3.494586000 | -2.425197000 | 4.448138000 |

| | | | |
|---|---|---|---|
| 6 | -3.613989000 | -2.176619000 | 1.045877000 |
| 6 | -3.467016000 | -0.571588000 | 3.357862000 |
| 1 | -3.413068000 | 0.033667000 | 4.257540000 |
| 6 | -3.587606000 | -2.749365000 | 2.323498000 |
| 1 | -3.626863000 | -3.829922000 | 2.423043000 |
| 6 | -3.454810000 | 1.586154000 | 2.034205000 |
| 1 | -3.468136000 | 1.882162000 | 0.979929000 |
| 6 | -4.718677000 | 0.201549000 | -1.050220000 |
| 6 | -4.701541000 | 2.203167000 | 2.701884000 |
| 1 | -5.630207000 | 1.820145000 | 2.264701000 |
| 1 | -4.693573000 | 3.292422000 | 2.582731000 |
| 1 | -4.727410000 | 1.988139000 | 3.776028000 |
| 6 | -6.087676000 | -0.035868000 | -0.466575000 |
| 1 | -5.977093000 | -0.356104000 | 0.572783000 |
| 1 | -6.557432000 | -0.878158000 | -0.995917000 |
| 6 | -4.436192000 | 0.734013000 | -2.273206000 |
| 6 | -2.167542000 | 2.164245000 | 2.656061000 |
| 1 | -2.104705000 | 1.935879000 | 3.726197000 |
| 1 | -2.162725000 | 3.255421000 | 2.552063000 |
| 1 | -1.260811000 | 1.776938000 | 2.179737000 |
| 6 | -3.692381000 | -3.075955000 | -0.185791000 |
| 1 | -3.770265000 | -2.441452000 | -1.074567000 |
| 6 | -5.390339000 | 1.200789000 | -3.341050000 |
| 1 | -5.878201000 | 0.322406000 | -3.789063000 |
| 1 | -4.815721000 | 1.668935000 | -4.148099000 |
| 6 | -6.470120000 | 2.187294000 | -2.856000000 |
| 1 | -6.978203000 | 2.577558000 | -3.746156000 |
| 1 | -5.984725000 | 3.045449000 | -2.372438000 |
| 6 | -2.412375000 | -3.921098000 | -0.345003000 |
| 1 | -1.522458000 | -3.285087000 | -0.422696000 |
| 1 | -2.471662000 | -4.534229000 | -1.251383000 |
| 1 | -2.269438000 | -4.595640000 | 0.506825000 |
| 6 | -7.527372000 | 1.587574000 | -1.917158000 |
| 1 | -8.327169000 | 2.327479000 | -1.790220000 |
| 1 | -7.994870000 | 0.718708000 | -2.404179000 |
| 6 | -4.944033000 | -3.975589000 | -0.158523000 |
| 1 | -4.914362000 | -4.686190000 | 0.674828000 |
| 1 | -5.010995000 | -4.556554000 | -1.085066000 |
| 1 | -5.861804000 | -3.385584000 | -0.059865000 |
| 6 | -7.034617000 | 1.180653000 | -0.519681000 |
| 1 | -7.909662000 | 0.931807000 | 0.093014000 |
| 1 | -6.550446000 | 2.038396000 | -0.034441000 |
| 6 | 6.412218000 | 3.069595000 | -0.465473000 |
| 8 | 6.246983000 | 2.076602000 | -1.184588000 |

| | | | |
|---|---|---|---|
| 8 | 5.619712000 | 3.439791000 | 0.514317000 |
| 1 | 4.811553000 | 2.838640000 | 0.669774000 |
| 6 | 7.586326000 | 3.997820000 | -0.651920000 |
| 1 | 8.223320000 | 3.643269000 | -1.462412000 |
| 1 | 7.224853000 | 5.006759000 | -0.876666000 |
| 1 | 8.159913000 | 4.061882000 | 0.278439000 |
| 6 | 2.506910000 | 2.246673000 | 1.706881000 |
| 8 | 3.494050000 | 1.991572000 | 0.963279000 |
| 8 | 1.441769000 | 1.536564000 | 1.736905000 |
| 1 | 1.458668000 | 0.342361000 | 1.047051000 |
| 6 | 2.561248000 | 3.431612000 | 2.654498000 |
| 1 | 3.045726000 | 3.115292000 | 3.586434000 |
| 1 | 3.157450000 | 4.241627000 | 2.226199000 |
| 1 | 1.557281000 | 3.784696000 | 2.897908000 |

Zero-point correction= 0.722266 (Hartree/Particle)
Thermal correction to Energy= 0.768819
Thermal correction to Enthalpy= 0.769763
Thermal correction to Gibbs Free Energy= 0.633102
Sum of electronic and zero-point Energies= -2350.800969
Sum of electronic and thermal Energies= -2350.754417
Sum of electronic and thermal Enthalpies= -2350.753472
Sum of electronic and thermal Free Energies= -2350.890134

TS32(1 imaginary frequencies, -1023.9 i $cm^{-1}$)

| | | | |
|---|---|---|---|
| 6 | 4.238621000 | -1.758364000 | 0.046487000 |
| 8 | 3.093983000 | -2.058480000 | 0.662110000 |
| 7 | 4.229886000 | -0.545520000 | -0.444953000 |
| 6 | 2.952013000 | 0.121836000 | -0.176132000 |
| 1 | 2.456700000 | 0.377606000 | -1.119301000 |
| 6 | 2.221196000 | -0.932140000 | 0.618758000 |
| 1 | 3.089584000 | 1.035673000 | 0.415543000 |
| 6 | 1.062633000 | -0.923333000 | 1.290949000 |
| 1 | 0.864796000 | -1.836159000 | 1.856073000 |
| 6 | 5.291668000 | -2.756381000 | 0.002116000 |
| 6 | 6.462084000 | -2.540581000 | -0.751993000 |
| 6 | 7.451367000 | -3.517915000 | -0.767147000 |
| 6 | 7.286081000 | -4.702169000 | -0.041489000 |
| 6 | 6.123426000 | -4.918022000 | 0.704555000 |
| 6 | 5.124492000 | -3.951591000 | 0.729058000 |
| 1 | 5.016083000 | -0.079572000 | -0.938650000 |
| 1 | 6.593837000 | -1.628787000 | -1.326147000 |
| 1 | 8.353991000 | -3.357758000 | -1.348420000 |
| 1 | 8.064813000 | -5.459109000 | -0.058534000 |
| 1 | 5.997903000 | -5.837780000 | 1.267001000 |

| | | | |
|---|---|---|---|
| 1 | 4.221473000 | -4.109001000 | 1.307919000 |
| 47 | -0.771894000 | -0.280396000 | 0.208457000 |
| 16 | -2.579928000 | 1.159234000 | -2.297823000 |
| 7 | -3.809128000 | -0.125194000 | -0.562493000 |
| 6 | -4.077205000 | -0.957792000 | 0.605696000 |
| 6 | -2.544426000 | 0.219354000 | -0.865507000 |
| 6 | -4.300153000 | -0.331499000 | 1.848849000 |
| 6 | -4.526732000 | -2.552745000 | 2.820405000 |
| 1 | -4.702292000 | -3.178625000 | 3.690753000 |
| 6 | -4.082116000 | -2.357656000 | 0.436111000 |
| 6 | -4.523723000 | -1.166971000 | 2.951283000 |
| 1 | -4.697264000 | -0.722084000 | 3.926244000 |
| 6 | -4.309753000 | -3.138621000 | 1.575901000 |
| 1 | -4.317767000 | -4.220742000 | 1.486187000 |
| 6 | -4.322296000 | 1.183849000 | 2.036062000 |
| 1 | -4.115726000 | 1.654922000 | 1.069228000 |
| 6 | -4.826706000 | 0.328629000 | -1.432966000 |
| 6 | -5.713004000 | 1.666847000 | 2.498666000 |
| 1 | -6.504179000 | 1.358918000 | 1.806455000 |
| 1 | -5.728774000 | 2.760311000 | 2.568433000 |
| 1 | -5.964529000 | 1.268552000 | 3.487980000 |
| 6 | -6.276049000 | -0.016022000 | -1.204628000 |
| 1 | -6.372792000 | -0.506397000 | -0.232481000 |
| 1 | -6.585452000 | -0.760682000 | -1.952907000 |
| 6 | -4.317336000 | 1.059730000 | -2.464539000 |
| 6 | -3.234418000 | 1.658910000 | 3.020362000 |
| 1 | -3.401983000 | 1.253035000 | 4.024615000 |
| 1 | -3.259761000 | 2.752108000 | 3.100247000 |
| 1 | -2.227513000 | 1.365643000 | 2.706465000 |
| 6 | -3.842686000 | -3.036413000 | -0.910646000 |
| 1 | -3.744506000 | -2.261156000 | -1.677322000 |
| 6 | -5.046103000 | 1.688925000 | -3.622775000 |
| 1 | -5.390374000 | 0.893610000 | -4.300495000 |
| 1 | -4.336335000 | 2.290179000 | -4.201973000 |
| 6 | -6.245601000 | 2.571542000 | -3.227507000 |
| 1 | -6.572306000 | 3.101105000 | -4.130730000 |
| 1 | -5.910043000 | 3.340758000 | -2.519294000 |
| 6 | -2.525206000 | -3.837877000 | -0.905895000 |
| 1 | -1.669467000 | -3.194274000 | -0.669772000 |
| 1 | -2.350900000 | -4.289426000 | -1.889178000 |
| 1 | -2.551817000 | -4.645774000 | -0.165888000 |
| 6 | -7.450092000 | 1.815880000 | -2.645910000 |
| 1 | -8.288946000 | 2.519053000 | -2.574087000 |
| 1 | -7.768570000 | 1.038110000 | -3.356146000 |

| | | | |
|---|---|---|---|
| 6 | -5.029033000 | -3.932683000 | -1.318285000 |
| 1 | -5.159258000 | -4.771164000 | -0.625291000 |
| 1 | -4.859700000 | -4.352376000 | -2.316181000 |
| 1 | -5.969252000 | -3.370824000 | -1.340750000 |
| 6 | -7.244141000 | 1.183528000 | -1.260801000 |
| 1 | -8.216341000 | 0.827797000 | -0.898437000 |
| 1 | -6.910387000 | 1.951471000 | -0.550699000 |
| 6 | 6.803898000 | 1.505029000 | -2.408486000 |
| 8 | 6.289893000 | 0.474406000 | -1.966049000 |
| 8 | 6.489464000 | 2.717299000 | -2.001790000 |
| 1 | 5.818368000 | 2.701718000 | -1.243859000 |
| 6 | 7.866799000 | 1.492711000 | -3.476050000 |
| 1 | 8.011727000 | 0.480423000 | -3.853860000 |
| 1 | 7.583749000 | 2.164262000 | -4.292322000 |
| 1 | 8.807292000 | 1.869673000 | -3.059302000 |
| 6 | 1.328586000 | 2.163315000 | 2.705558000 |
| 8 | 2.400625000 | 2.122918000 | 2.052270000 |
| 8 | 0.454605000 | 1.222766000 | 2.685342000 |
| 1 | 0.767871000 | 0.213721000 | 1.918311000 |
| 6 | 1.021697000 | 3.343263000 | 3.609124000 |
| 1 | -0.054963000 | 3.516860000 | 3.667794000 |
| 1 | 1.379032000 | 3.113847000 | 4.620307000 |
| 1 | 1.534428000 | 4.244066000 | 3.263233000 |
| 6 | 4.926682000 | 3.815600000 | 0.790556000 |
| 8 | 4.818820000 | 2.837224000 | 0.035417000 |
| 8 | 4.156220000 | 4.003396000 | 1.832950000 |
| 1 | 3.461686000 | 3.256800000 | 1.932838000 |
| 6 | 5.946566000 | 4.911728000 | 0.599081000 |
| 1 | 5.428904000 | 5.851273000 | 0.376857000 |
| 1 | 6.503341000 | 5.063725000 | 1.528654000 |
| 1 | 6.633125000 | 4.674680000 | -0.214244000 |

Zero-point correction= 0.785924 (Hartree/Particle)
Thermal correction to Energy= 0.838687
Thermal correction to Enthalpy= 0.839631
Thermal correction to Gibbs Free Energy= 0.686385
Sum of electronic and zero-point Energies= -2579.847992
Sum of electronic and thermal Energies= -2579.795229
Sum of electronic and thermal Enthalpies= -2579.794285
Sum of electronic and thermal Free Energies= -2579.947531

Re

| | | | |
|---|---|---|---|
| 6 | 0.362759000 | 0.855278000 | 0.121086000 |
| 8 | 0.507450000 | 2.055424000 | 0.340868000 |
| 7 | 1.440645000 | 0.030796000 | -0.071335000 |

| | | | |
|---|---|---|---|
| 6 | 2.787122000 | 0.597718000 | -0.095262000 |
| 1 | 2.892251000 | 1.250787000 | 0.778159000 |
| 6 | 3.800734000 | -0.456409000 | -0.085021000 |
| 1 | 2.922837000 | 1.244446000 | -0.975267000 |
| 6 | 4.634927000 | -1.328535000 | -0.093759000 |
| 1 | 5.378649000 | -2.092751000 | -0.090522000 |
| 6 | -0.992538000 | 0.210482000 | 0.054477000 |
| 6 | -1.203113000 | -1.164732000 | 0.231011000 |
| 6 | -2.491960000 | -1.694270000 | 0.159712000 |
| 6 | -3.580264000 | -0.855247000 | -0.088709000 |
| 6 | -3.377822000 | 0.517861000 | -0.252949000 |
| 6 | -2.092031000 | 1.048420000 | -0.175381000 |
| 1 | 1.312169000 | -0.879340000 | -0.488787000 |
| 1 | -0.370494000 | -1.823999000 | 0.461767000 |
| 1 | -2.646499000 | -2.759662000 | 0.307021000 |
| 1 | -4.583529000 | -1.268943000 | -0.145860000 |
| 1 | -4.223531000 | 1.174372000 | -0.438643000 |
| 1 | -1.912851000 | 2.112737000 | -0.286706000 |

Zero-point correction= 0.165329 (Hartree/Particle)
Thermal correction to Energy= 0.176400
Thermal correction to Enthalpy= 0.177344
Thermal correction to Gibbs Free Energy= 0.126727
Sum of electronic and zero-point Energies= -516.220832
Sum of electronic and thermal Energies= -516.209760
Sum of electronic and thermal Enthalpies= -516.208816
Sum of electronic and thermal Free Energies= -516.259434

HOAc

| | | | |
|---|---|---|---|
| 6 | 0.092406000 | 0.125505000 | -0.000093000 |
| 8 | 0.645374000 | 1.202143000 | 0.000018000 |
| 8 | 0.779355000 | -1.046313000 | -0.000004000 |
| 1 | 1.724298000 | -0.802328000 | 0.000161000 |
| 6 | -1.397853000 | -0.110157000 | -0.000008000 |
| 1 | -1.685528000 | -0.693524000 | -0.881119000 |
| 1 | -1.685687000 | -0.690785000 | 0.882883000 |
| 1 | -1.918233000 | 0.847909000 | -0.001433000 |

Zero-point correction= 0.062028 (Hartree/Particle)
Thermal correction to Energy= 0.066596
Thermal correction to Enthalpy= 0.067540
Thermal correction to Gibbs Free Energy= 0.034769
Sum of electronic and zero-point Energies= -229.015581
Sum of electronic and thermal Energies= -229.011014
Sum of electronic and thermal Enthalpies= -229.010070
Sum of electronic and thermal Free Energies= -229.042841

**Computational Methods.** For frontier orbital calculations, all of the calculations were performed using Gaussian 09 suite of programs. All of the geometry optimizations were performed at the B3LYP level of theory in the gas phase and the 6-311++G(d,p) basis set (*Beilstein J. Org. Chem.* **2015**, *11*, 2727). For geometry optimizations, we employed the X-ray structures of 3-(2,6-diisopropylphenyl)-4,5-dimethylthiazol-3-ium, 3-(2,6-diisopropylphenyl)-4,5,6,7-tetrahydrobenzo[*d*]thiazol-3-ium, 3-(2,6-diisopropyl phenyl)-5,6,7,8-tetrahydro-4*H*-cyclohepta[*d*]thiazol-3-ium, 3-mesityl-4,5-dimethyl thiazol-3-ium or their metal complexes as the starting geometry and performed full optimization. The absence of imaginary frequencies was used to characterize the structures as minima on the potential energy surface. All of the optimized geometries were verified as minima (no imaginary frequencies). Energetic parameters were calculated under standard conditions (298.15 K and 1 atm). Structural representations were generated using CYLview software (Legault, C. Y. CYLview version 1.0 BETA, University of Sherbrooke). All other representations were generated using GaussView (GaussView, version 5, Dennington, R.; Keith, T.; Millam, J. Semichem Inc., Shawnee Mission, KS, 2009) or ChemCraft software (Andrienko, G. L. ChemCraft version b562a, https://www.chemcraftprog.com).

**Supplementary Table S7. HOMO and LUMO Energy Levels of Thiazol-2-ylidene Ligands Calculated at the B3LYP 6-311++g(d,p) Level**[a,b]

| entry | carbene | orbital | E [au] | E [eV] |
|---|---|---|---|---|
| 1 | **$^{Me}$IPrS (3a)** | LUMO+3 | -0.0134 | -0.36 |
| 2 | **$^{Me}$IPrS (3a)** | HOMO | -0.2232 | -6.07 |
| 3 | **$^{Me}$IPrS (3a)** | HOMO-1 | -0.2416 | -6.57 |
| 4 | **$^{6}$IPrS (3b)** | LUMO+3 | -0.0123 | -0.33 |
| 5 | **$^{6}$IPrS (3b)** | HOMO | -0.2203 | -5.99 |
| 6 | **$^{6}$IPrS (3b)** | HOMO-1 | -0.2367 | -6.44 |
| 7 | **$^{7}$IPrS (3c)** | LUMO+3 | -0.0107 | -0.29 |
| 8 | **$^{7}$IPrS (3c)** | HOMO | -0.2225 | -6.05 |
| 9 | **$^{7}$IPrS (3c)** | HOMO-1 | -0.2385 | -6.49 |
| 10 | **$^{Me}$IMesS (3d)** | LUMO+3 | -0.0134 | -0.36 |
| 11 | **$^{Me}$IMesS (3d)** | HOMO | -0.2211 | -6.02 |
| 12 | **$^{Me}$IMesS (3d)** | HOMO-1 | -0.2391 | -6.51 |

[a]See, Falivene, L.; Cavallo, L. *Coord. Chem. Rev.* **2017**, *344*, 101-114. [b]LUMO+3 due to required orbital symmetry.

**Supplementary Figure S142. Graphical Representation of Frontier Orbitals of Thiazol-2-ylidene Ligands, B3LYP 6-311++g(d,p) Level. See Supplementary Table S7 for Energy Levels.**

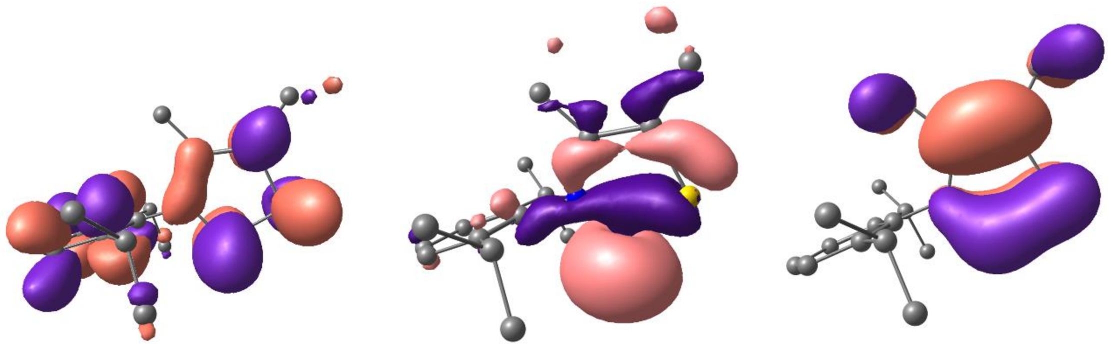

**LUMO+3 $^{Me}$IPrS (3a)** **HOMO $^{Me}$IPrS (3a)** **HOMO–1 $^{Me}$IPrS (3a)**

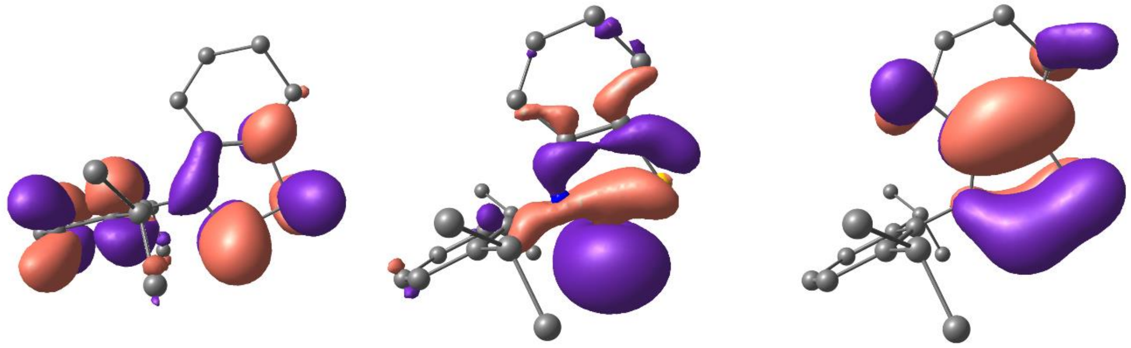

**LUMO+3 $^{6}$IPrS (3b)** **HOMO $^{6}$IPrS (3b)** **HOMO–1 $^{6}$IPrS (3b)**

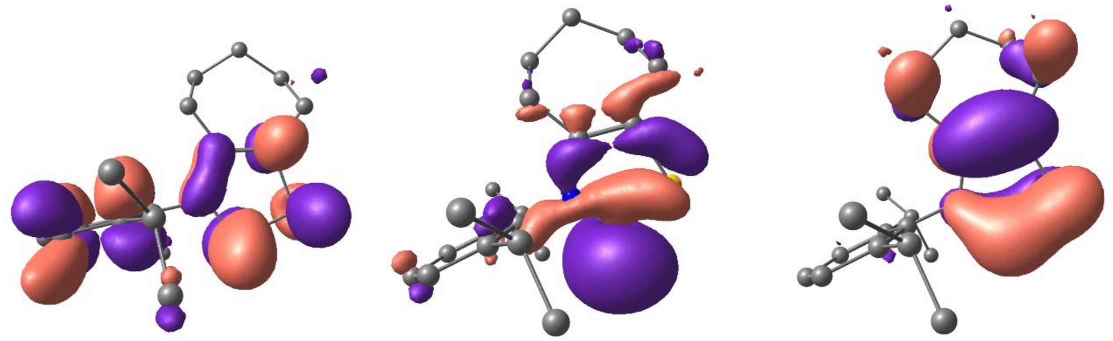

**LUMO+3 $^{7}$IPrS (3c)** **HOMO $^{7}$IPrS (3c)** **HOMO–1 $^{7}$IPrS (3c)**

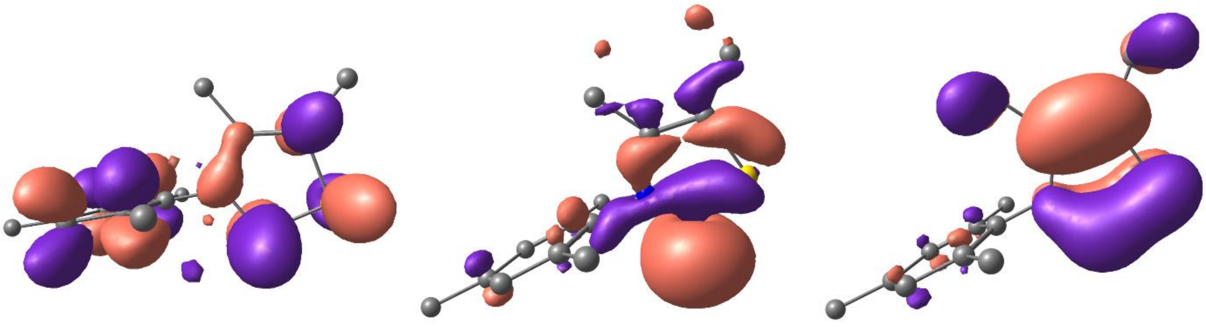

**LUMO+3 $^{Me}$IMesS (3d)** **HOMO $^{Me}$IMesS (3d)** **HOMO–1 $^{Me}$IMesS (3d)**

$^{Me}$IPrS

Energy: -1114.779025 au

Sum of electronic and thermal Energies: -1114.400187 au

Geometry:

| | | | |
|---|---|---|---|
| S | 2.88010300 | -0.00001500 | -1.34882600 |
| N | 0.67926600 | -0.00000300 | -0.25935400 |
| C | 1.15923000 | -0.00000700 | -1.51993500 |
| C | 2.87285100 | -0.00001200 | 0.41613500 |
| C | 1.58534100 | -0.00000600 | 0.83696900 |
| C | 4.13801900 | -0.00001700 | 1.21696900 |
| H | 4.74733000 | 0.88151500 | 0.99617600 |
| H | 4.74731900 | -0.88155800 | 0.99618200 |
| H | 3.93040400 | -0.00001200 | 2.28877300 |
| C | 1.06764200 | -0.00000200 | 2.24208300 |
| H | 0.44962200 | -0.87961600 | 2.44225900 |
| H | 0.44963300 | 0.87962000 | 2.44225800 |
| H | 1.89447900 | -0.00000700 | 2.95241100 |
| C | -0.75851700 | 0.00000300 | -0.04820400 |
| C | -1.42749300 | -1.23304800 | 0.04036800 |
| C | -2.81308600 | -1.20485700 | 0.22616100 |
| H | -3.36348700 | -2.13580400 | 0.29233500 |
| C | -3.49969900 | 0.00001700 | 0.32056500 |
| H | -4.57485400 | 0.00002200 | 0.46327500 |
| C | -2.81307400 | 1.20488400 | 0.22616500 |
| H | -3.36346500 | 2.13583600 | 0.29234100 |
| C | -1.42748100 | 1.23306200 | 0.04037100 |
| C | -1.12553900 | 3.61817500 | 0.90886400 |
| H | -1.02920300 | 3.24656200 | 1.93291200 |
| H | -2.16203700 | 3.93532800 | 0.76467900 |
| H | -0.49844000 | 4.50900500 | 0.81238000 |
| C | -0.69589200 | 2.56118600 | -0.12275800 |
| H | 0.37001800 | 2.37749300 | 0.02971100 |
| C | -0.86159600 | 3.08970600 | -1.56181500 |
| H | -1.91365100 | 3.29677700 | -1.78137800 |
| H | -0.49705100 | 2.35982000 | -2.28745000 |
| H | -0.29897400 | 4.01899000 | -1.69426100 |
| C | -0.86164700 | -3.08971100 | -1.56181100 |
| H | -0.49710700 | -2.35983200 | -2.28745600 |
| H | -1.91370600 | -3.29677600 | -1.78135800 |
| H | -0.29903300 | -4.01899900 | -1.69425800 |
| C | -0.69592000 | -2.56118100 | -0.12276100 |
| H | 0.36999400 | -2.37749700 | 0.02969400 |
| C | -1.12556500 | -3.61815600 | 0.90887700 |

| | | | |
|---|---|---|---|
| H | -2.16206900 | -3.93529600 | 0.76471100 |
| H | -1.02920600 | -3.24653700 | 1.93292000 |
| H | -0.49847900 | -4.50899600 | 0.81238900 |

$^{6}$IPrS

Energy: -1192.218940 au

Sum of electronic and thermal Energies: -1191.801864 au

Geometry:

| | | | |
|---|---|---|---|
| N | -0.27154700 | 0.02621800 | 0.62826800 |
| C | -0.44318900 | 0.05325300 | 1.96799700 |
| S | -2.16018300 | 0.11066300 | 2.20901900 |
| C | -2.56001000 | 0.09339800 | 0.49594200 |
| C | -3.93360300 | 0.11776600 | -0.11106600 |
| C | -3.86551900 | -0.28425400 | -1.59649600 |
| H | -4.81332400 | -0.04329600 | -2.08635800 |
| H | -3.73715300 | -1.37044700 | -1.67034900 |
| C | -2.70239400 | 0.41298600 | -2.31561000 |
| C | -1.33888500 | 0.02637200 | -1.71276200 |
| H | -1.04615000 | -0.97496600 | -2.05340900 |
| H | -0.55885600 | 0.70454100 | -2.07058600 |
| C | -1.40986800 | 0.05170500 | -0.21258300 |
| C | 1.07131100 | -0.03025900 | 0.07751400 |
| C | 1.64692600 | -1.28867000 | -0.16795800 |
| C | 2.94618700 | -1.31574600 | -0.68433100 |
| H | 3.42517100 | -2.26802100 | -0.87886600 |
| C | 3.63873200 | -0.13932200 | -0.94562700 |
| H | 4.64639900 | -0.18209300 | -1.34454200 |
| C | 3.04552100 | 1.09188300 | -0.69164100 |
| H | 3.60104000 | 2.00015900 | -0.89285700 |
| C | 1.74875300 | 1.17532000 | -0.17529900 |
| C | 0.92384900 | -2.58753100 | 0.17206900 |
| H | -0.13646800 | -2.35808700 | 0.29752600 |
| C | 1.03034100 | -3.64500900 | -0.93993300 |
| H | 0.68931600 | -3.25368000 | -1.90250100 |
| H | 0.41344800 | -4.51276800 | -0.68952000 |
| H | 2.05534400 | -4.00331600 | -1.06922700 |
| C | 1.42712100 | -3.14348300 | 1.51929300 |
| H | 2.49041300 | -3.39685400 | 1.46376000 |
| H | 0.87682500 | -4.05064300 | 1.78742100 |
| H | 1.29092700 | -2.41007400 | 2.31671000 |
| C | 1.13268900 | 2.53131700 | 0.15212100 |
| H | 0.05602000 | 2.39013200 | 0.26938600 |
| C | 1.66936400 | 3.05187000 | 1.50058500 |
| H | 1.46614200 | 2.33692100 | 2.30038600 |

| | | | |
|---|---|---|---|
| H | 1.19400400 | 4.00316700 | 1.75894100 |
| H | 2.75042100 | 3.21595800 | 1.45223000 |
| C | 1.33423600 | 3.57048200 | -0.96399800 |
| H | 2.38587400 | 3.84442100 | -1.08540800 |
| H | 0.78728100 | 4.48646200 | -0.72306100 |
| H | 0.97078100 | 3.20347600 | -1.92795500 |
| H | -4.36369800 | 1.12261600 | -0.01321000 |
| H | -4.60386800 | -0.55550600 | 0.43233900 |
| H | -2.83342100 | 1.49883400 | -2.24133500 |
| H | -2.71210100 | 0.16648300 | -3.38099300 |

[7]IPrS

Energy: -1231.538709 au

Sum of electronic and thermal Energies: -1231.091830 au

Geometry:

| | | | |
|---|---|---|---|
| S | 1.72020600 | 0.75365400 | 2.38389200 |
| N | 0.02549900 | 0.22095400 | 0.68930100 |
| C | -1.24994300 | -0.10409800 | 0.07319500 |
| C | 2.27286600 | 0.67832000 | 0.71517600 |
| C | -2.42134400 | 3.00462400 | 1.04504600 |
| H | -2.10825700 | 4.04011500 | 1.21051500 |
| H | -2.20117100 | 2.43018700 | 1.94724100 |
| H | -3.50490000 | 2.99963600 | 0.89098700 |
| C | 1.21521300 | 0.37401400 | -0.07492800 |
| C | 0.07111400 | 0.38212900 | 2.02982900 |
| C | -2.07584700 | 0.94676400 | -0.36228900 |
| C | -3.69949400 | -0.71878900 | -1.05748800 |
| H | -4.66047500 | -0.95969200 | -1.49887500 |
| C | -1.62197700 | -1.45508500 | -0.03926300 |
| C | -2.86488900 | -1.73804700 | -0.61418600 |
| H | -3.18738600 | -2.76805500 | -0.71086600 |
| C | 3.75137300 | -0.22350500 | -2.00905400 |
| H | 3.82981300 | 0.76085900 | -2.49037100 |
| H | 4.38965600 | -0.89506700 | -2.59370000 |
| C | 3.69585600 | 0.94552300 | 0.30850400 |
| H | 4.30394600 | 1.05385300 | 1.21089700 |
| H | 3.75063500 | 1.91455600 | -0.20657400 |
| C | 2.30374000 | -0.72355500 | -2.12404300 |
| H | 2.08493400 | -0.89005600 | -3.18444000 |
| H | 2.21172400 | -1.69883100 | -1.63244500 |
| C | 1.22291500 | 0.22512600 | -1.57314600 |
| H | 1.35246800 | 1.21485800 | -2.03211400 |
| H | 0.24329400 | -0.12913700 | -1.89802700 |
| C | -0.74538800 | -2.58306000 | 0.49382400 |

| | | | |
|---|---|---|---|
| H | 0.25378500 | -2.17778400 | 0.66602400 |
| C | -1.27372000 | -3.07418900 | 1.85641600 |
| H | -2.27807200 | -3.49750000 | 1.75639700 |
| H | -1.31755500 | -2.25183300 | 2.57331200 |
| H | -0.61841800 | -3.85117700 | 2.26208000 |
| C | -1.68930500 | 2.40985500 | -0.17454600 |
| H | -0.62039800 | 2.44920000 | 0.04466000 |
| C | -0.59787500 | -3.74904000 | -0.49884900 |
| H | 0.11246600 | -4.48355300 | -0.10853500 |
| H | -0.23133300 | -3.40688700 | -1.47064000 |
| H | -1.54490900 | -4.27042300 | -0.66341300 |
| C | -1.92485600 | 3.25993600 | -1.43482300 |
| H | -2.98735900 | 3.34813300 | -1.67786400 |
| H | -1.41624400 | 2.83745500 | -2.30578100 |
| H | -1.54329200 | 4.27298400 | -1.27819800 |
| C | -3.30831500 | 0.60891400 | -0.92961500 |
| H | -3.97383200 | 1.39353100 | -1.26983500 |
| C | 4.32681200 | -0.13449100 | -0.58811700 |
| H | 4.23898100 | -1.10826400 | -0.09253700 |
| H | 5.39848000 | 0.07969000 | -0.66514400 |

$^{Me}$IMesS

Energy: -996.815021 au

Sum of electronic and thermal Energies: -996.525623 au

Geometry:

| | | | |
|---|---|---|---|
| N | 0.76784300 | -0.00000200 | -0.36182700 |
| C | 1.30281000 | -0.00000800 | -1.59946800 |
| S | 3.01418200 | -0.00000600 | -1.35146100 |
| C | 2.92808200 | 0.00000200 | 0.41178600 |
| C | 1.62308900 | 0.00000400 | 0.77421000 |
| C | 4.15590300 | 0.00000700 | 1.26875000 |
| H | 4.77465400 | 0.88154700 | 1.07589800 |
| H | 4.77465300 | -0.88153600 | 1.07590800 |
| H | 3.90018800 | 0.00001300 | 2.33019700 |
| C | 1.04033900 | 0.00000800 | 2.15373800 |
| H | 0.41309000 | -0.87932300 | 2.32588300 |
| H | 0.41308900 | 0.87934000 | 2.32587800 |
| H | 1.83299600 | 0.00001000 | 2.90207600 |
| C | -0.67458000 | -0.00000100 | -0.21530000 |
| C | -1.35169500 | -1.22430700 | -0.15906700 |
| C | -2.74083900 | -1.19868800 | -0.01475900 |
| H | -3.27907100 | -2.14089900 | 0.02420400 |
| C | -3.45229600 | 0.00000100 | 0.06671800 |
| C | -2.74083700 | 1.19868900 | -0.01476600 |

| | | | |
|---|---|---|---|
| H | -3.27906900 | 2.14090100 | 0.02419200 |
| C | -1.35169400 | 1.22430500 | -0.15907400 |
| C | -0.61260200 | -2.53253800 | -0.29049500 |
| H | -0.10907200 | -2.59522500 | -1.25927200 |
| H | -1.30072600 | -3.37486000 | -0.20322000 |
| H | 0.16029400 | -2.64593900 | 0.47556700 |
| C | -4.95048400 | 0.00000300 | 0.25439400 |
| H | -5.40831000 | -0.88437400 | -0.19466800 |
| H | -5.40831700 | 0.88434000 | -0.19474100 |
| H | -5.21228700 | 0.00004600 | 1.31829700 |
| C | -0.61260000 | 2.53253500 | -0.29051100 |
| H | 0.16029500 | 2.64594100 | 0.47555200 |
| H | -1.30072300 | 3.37485800 | -0.20324300 |
| H | -0.10906900 | 2.59521500 | -1.25928800 |

## Supplementary References


1. (a) Ishii T, Ota K, Nagao K, Ohmiya H. N-Heterocyclic Carbene-Catalyzed Radical Relay Enabling Vicinal Alkylacylation of Alkenes. *J Am Chem Soc* **141**, 14073-14077 (2019). (b) Ishii T, Kakeno Y, Nagao K, Ohmiya H. N-Heterocyclic Carbene-Catalyzed Decarboxylative Alkylation of Aldehydes. *J Am Chem Soc* **141**, 3854-3858 (2019).
2. Herszman JD, Berger M, Waldvogel SR. Fluorocyclization of N-Propargylamides to Oxazoles by Electrochemically Generated $ArIF_2$. *Org Lett* **21**, 7893-7896 (2019).
3. Nalivela KS, Rudolph M, Baeissa ES, Alhogbi BG, Mkhalid IAI, Hashmi ASK. Sequential Au/Cu Catalysis: A Two Catalyst One-Pot Protocol for the Enantioselective Synthesis of Oxazole α-Hydroxy Esters via Intramolecular Cyclization/Intermolecular Alder-Ene Reaction. *Adv Synth Catal* **360**, 2183-2190 (2018).
4. Lukin A, Vedekhina T, Tovpeko D, Zhurilo N, Krasavin M. Zn-catalyzed hydrohydrazination of propargylamides with $BocNHNH_2$: a novel entry into the 1,2,4-triazine core. *RSC Adv* **6**, 57956-57959 (2016).
5. Safrygin A, Dar'in D, Lukin A, Bakholdina A, Sapegin A, Krasavin M. $Zn(OTf)_2$-catalyzed, microwave-promoted synthesis of 2-substituted 5-methyloxazoles from propargylic amides. *Tetrahedron Lett* **60**, 777-779 (2019).
6. Wong VHL, Vummaleti SVC, Cavallo L, White AJP, Nolan SP, Hii KK. Synthesis, structure and catalytic activity of NHC-AgI carboxylate complexes. *Chem - Eur J* **22**, 13320-13327 (2016).
7. Deprez-Poulain R*, et al.* Catalytic site inhibition of insulin-degrading enzyme by a small molecule induces glucose intolerance in mice. *Nat Commun* **6**, 8250 (2015).
8. Wong VHL, White AJP, Hor TS, Hii KK. Silver-Catalyzed Cyclization of Propargylic Amides to Oxazolines. *Adv Synth Catal* **357**, 3943-3948 (2015).
9. Wang Y, Jiang M, Liu J-T. Copper-catalyzed stereoselective oxytrifluoromethylation of propargyl amides for the construction of oxazolines. *Org Chem Front* **2**, 542-547 (2015).
10. Wang W*, et al.* 3-Silaazetidine: An Unexplored yet Versatile Organosilane Species for Ring Expansion toward Silaazacycles. *J Am Chem Soc* **143**, 11141-11151 (2021).
11. Senadi GC, Hu W-P, Hsiao J-S, Vandavasi JK, Chen C-Y, Wang J-J. Facile, Selective, and Regiocontrolled Synthesis of Oxazolines and Oxazoles Mediated by $ZnI_2$ and $FeCl_3$. *Org Lett* **14**, 4478-4481 (2012).
12. Mali JK, Takale BS, Telvekar VN. Readily switchable one-pot 5-exo-dig cyclization using a palladium catalyst. *RSC Adv* **7**, 2231-2235 (2017).
13. Hashmi ASK, Blanco Jaimes MC, Schuster AM, Rominger F. From propargylic amides to functionalized oxazoles: Domino gold catalysis/oxidation by dioxygen. *J Org Chem* **77**, 6394-6408 (2012).
14. Weyrauch JP*, et al.* Cyclization of propargylic amides: mild access to oxazole derivatives. *Chem - Eur J* **16**, 956-963, S956/951-S956/958 (2010).
15. Doherty S*, et al.* Triaryl-like MONO-, BIS-, and TRISKITPHOS phosphines: synthesis, solution NMR studies, and a comparison in gold-catalyzed carbon-heteroatom bond forming 5-exo-dig and 6-endo-dig cyclizations. *Organometallics* **35**, 1265-1278 (2016).
16. Priante-Flores A, Salazar-Pereda V, Rheingold AL, Mendoza-Espinosa D.

Synthesis and characterization of a gold(I) bis(triazolylidene) complex featuring a large [$(TpMe_2)_2K$] anion. *New J Chem* **42**, 15533-15537 (2018).
17. Alhalib A, Moran WJ. CuI-catalyzed cycloisomerization of propargyl amides. *Org Biomol Chem* **12**, 795-800 (2014).
